# A resource database for protein kinase substrate sequence-preference motifs based on large-scale mass spectrometry data

**DOI:** 10.1186/s12964-023-01436-2

**Published:** 2024-02-19

**Authors:** Brian G. Poll, Kirby T. Leo, Venky Deshpande, Nipun Jayatissa, Trairak Pisitkun, Euijung Park, Chin-Rang Yang, Viswanathan Raghuram, Mark A. Knepper

**Affiliations:** 1 Epithelial Systems Biology Laboratory, Systems Biology Center, Division of Intramural Research, National Heart, Lung, and Blood Institute, National Institutes of Health, 10 Center Drive, National Institutes of Health, Bethesda, MD 20892-1603 USA; 2 Center of Excellence in Systems Biology, Faculty of Medicine, Chulalongkorn University, Bangkok, 10330 Thailand

**Keywords:** Phosphorylation, Protein kinases, Kinase prediction

## Abstract

**Background:**

Protein phosphorylation is one of the most prevalent posttranslational modifications involved in molecular control of cellular processes, and is mediated by over 520 protein kinases in humans and other mammals. Identification of the protein kinases responsible for phosphorylation events is key to understanding signaling pathways. Unbiased phosphoproteomics experiments have generated a wealth of data that can be used to identify protein kinase targets and their preferred substrate sequences.

**Methods:**

This study utilized prior data from mass spectrometry-based studies identifying sites of protein phosphorylation after in vitro incubation of protein mixtures with recombinant protein kinases. *PTM-Logo* software was used with these data to generate position-dependent Shannon information matrices and sequence motif ‘logos’. Webpages were constructed for facile access to logos for each kinase and a new stand-alone application was written in *Python* that uses the position-dependent Shannon information matrices to identify kinases most likely to phosphorylate a particular phosphorylation site.

**Results:**

A database of kinase substrate target preference logos allows browsing, searching, or downloading target motif data for each protein kinase (https://esbl.nhlbi.nih.gov/Databases/Kinase_Logos/). These logos were combined with phylogenetic analysis of protein kinase catalytic sequences to reveal substrate preference patterns specific to particular groups of kinases (https://esbl.nhlbi.nih.gov/Databases/Kinase_Logos/KinaseTree.html). A stand-alone program, *KinasePredictor*, is provided (https://esbl.nhlbi.nih.gov/Databases/Kinase_Logos/KinasePredictor.html). It takes as input, amino-acid sequences surrounding a given phosphorylation site and generates a ranked list of protein kinases most likely to phosphorylate that site.

**Conclusions:**

This study provides three new resources for protein kinase characterization. It provides a tool for prediction of kinase-substrate interactions, which in combination with other types of data (co-localization, etc.), can predict which kinases are likely responsible for a given phosphorylation event in a given tissue.

Video Abstract

**Supplementary Information:**

The online version contains supplementary material available at 10.1186/s12964-023-01436-2.

## Background

Protein phosphorylation, mediated by protein kinases and reversed by phosphatases, is a key process in the regulation of cellular function. There are at least 520 protein kinases in mammalian genomes [1], and a large proportion of the human proteome is believed to be phosphorylated [2, 3]. This results in a staggering number of possible interactions between kinases and potential target proteins. Because of their key roles, protein kinases are the second most common target for drugs after G-protein coupled receptors [2]. A fundamental objective in biology is to identify what protein kinase is responsible for a particular phosphorylation event in a given protein [4].

Phosphorylation usually occurs on either serine (S), threonine (T), or tyrosine (Y) residues in a target protein. When predicting kinase-substrate interactions, the amino acid sequence of the target protein is a major factor, but other important factors must be taken into account. These include relative expression levels of substrates and kinases, relative localization of the substrate and kinases within the cell and the presence of other post-translational modifications including other phosphorylated residues. Nevertheless, a key determinant in the specification of kinases in signaling models is the sequence surrounding the phosphorylated amino acid in the substrate. Many tools have been introduced for kinase prediction based on target sequence including Phosida [5], PhosphoNet [6], Phosphomotif Finder [7], SCANSITE [8], and PhosphoSitePlus [9], and these are based largely on curated data sets from reductionist experiments. Thus, many protein kinases are not represented in these databases. Combinatorial peptide library screening methods have also been employed to identify kinase target sequences, markedly increasing the number of protein kinases with known target specificities [10, 11]. An alternative source of data for kinase substrate profiling is in vitro phosphorylation/phosphoproteomics, introduced by Douglass et al. in 2014 [12], in which mixtures of dephosphorylated proteins are incubated with recombinant protein kinases followed by identification of phosphorylated sites using mass spectrometry. Although the Douglass et al. study did this with a relatively low number of kinases, a later study by Sugiyama et al. [13] used the in vitro phosphorylation/phosphoproteomics technique to profile 384 recombinant human protein kinases, providing an extensive data set relevant to the identification of kinase substrate target preferences. The publication of this dataset was an important step forward, and the authors shared their data for the benefit of other investigators, although the data were not presented in a user-friendly fashion that would allow facile interrogation by potential users. Our objective was to curate this dataset, combined with that of Douglass et al. [12], and develop web resources to aid further in generating kinase-substrate predictions. Specifically, we have developed: 1) a web based listing of protein kinases allowing mouse-over display of sequence preference logos for each kinase along with “anti-logos” showing disfavored amino acids (https://esbl.nhlbi.nih.gov/Databases/Kinase_Logos/); 2) a dendrogram-based on kinase catalytic sequences mapped to kinase sequence preference logos (https://esbl.nhlbi.nih.gov/Databases/Kinase_Logos/KinaseTree.html); and 3) *KinasePredicto*r, a computational tool ranking kinases with regard to the degree of match of their sequence preferences to an inputted phosphopeptide sequence (https://esbl.nhlbi.nih.gov/Databases/Kinase_Logos/KinasePredictor.html).

## Methods

### Kinase logo generation

The data from this resource was curated from Sugiyama et al. [13]. Table S2 of Sugiyama et al. showed UniProt IDs for substrates that had the presence of site-determining ions and high localization probability (*P* > 0.75) based on PTM score. These sites were converted to amino acid sequences with the UniProt Retrieve/ID mapping tool (https://www.uniprot.org/uploadlists/). Amino acid sequences from the ID search with matching phosphorylation sites (i.e., an amino acid equivalent with the reported position) were reported as 13-amino-acid centralized sequences, correcting with “J” placeholders for sites with overhang positions at the ends of proteins. These substrate “Centralized AA Sequences” were united to their corresponding kinases in the original dataset.

For each protein kinase, the 13-amino-acid centralized sequences were input into *PTM-Logo*, described in Saethang et al. [14]. All kinase logos were generated from a minimum of 30 target amino acid sequences. The number of input sequences needed for kinase specificity can vary widely based on the strength of kinase-substrate interactions, but prior studies have suggested that preferences for specific kinases can be seen starting between 5 and 20 input sequences [9]. All kinase-target sequence datasets were analyzed using Chi-squared filtering alpha = 0.0001. The same analysis was done for Anti-logo (disfavored) residues. This analysis yielded 384 protein kinase sequence motifs, which were then compiled into an online web resource (https://esbl.nhlbi.nih.gov/Databases/Kinase_Logos/).

### Kinase alignment and phylogenetic tree

Protein kinases were aligned based on their catalytic subunits based on the classification of Manning et al. [1], and then converted into a phylogenetic tree using Interactive Tree of Life (iToL, https://itol.embl.de/) [15]. This was also compiled into an online resource. https://esbl.nhlbi.nih.gov/Databases/Kinase_Logos/KinaseTree.html. Each protein kinase represented on the phylogenetic tree is associated with its respective logo.

### KinasePredictor

The kinase prediction software (*KinasePredictor*) is made using the Python programming language (Python 3.8.5). The program and its associated data files are available at (https://esbl.nhlbi.nih.gov/Databases/Kinase_Logos/KinasePredictor.html). Using the sequence data from the Sugiyama et al. dataset, we generated a series of information content and probability matrices for each kinase, showing the likelihood of each amino acid appearing at each position relative to the modified residue. KinasePredictor takes a centralized 13 amino acid input sequence. For each kinase, KinasePredictor calculates a scalar based on the residue and position according to the following equation:

$${\boldsymbol{M}}_{\boldsymbol{s}}^{\boldsymbol{k}}=\sum \boldsymbol{x}\left(\boldsymbol{i},\boldsymbol{j}\right)\times {\boldsymbol{P}}_{\boldsymbol{j}}^{\boldsymbol{k}}$$

Where *k* is the index of the kinase, *j* is the position index of the input sequence *x*, *i* is the index for the amino acid and ${P}_j^k$ is the information content score for each kinase at that position and amino acid. These values are then totaled to give a scalar representation of the likelihood of each kinase to recognize the input sequence. The program then provides a ranked list of each of the kinases by these predictive values. Users can sort the results and save them as a csv export.

## Results

### Kinase substrate logo database

We generated target sequence preference motifs for each of the 384 protein kinases using the data from Sugiyama et al. [13]. These data originated from mass spectrometry based identification of phosphorylated peptides after incubation of dephosphorylated protein mixtures from HeLa cells with each of 384 recombinant protein kinases (Supplemental Spreadsheet 1). To generate logos for each kinase, centralized versions of these phosphopeptide sequences were input into *PTMLogo*, a program for generating sequence-based substrate preference logos utilizing position-specific information content [14]. The resulting database of kinase sequence motif logos is provided as a searchable and downloadable online web resource at https://esbl.nhlbi.nih.gov/Databases/Kinase_Logos/. *PTMLogo* also allows us to calculate disfavored amino acid residues for particular kinase substrate preference sequences, or “Anti-Logos”. These were also included in the online resource web page.

**Fig. 1:**
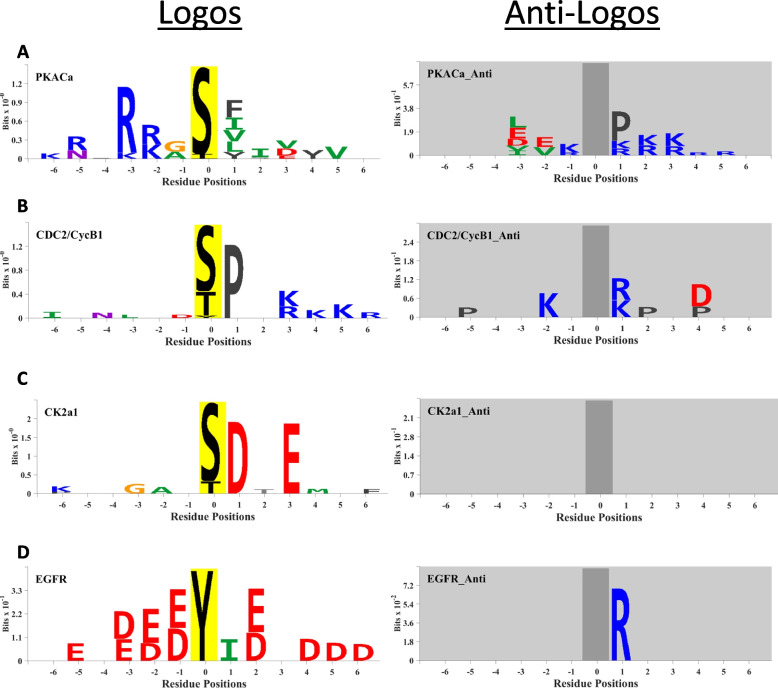
Examples of substrate preference logos (left) and anti-logos (right) for selected protein kinases. **A** PKACα (protein kinase cAMP-activated catalytic subunit alpha; gene symbol: Prkaca), **B** CDC2 (cyclin dependent kinase 1; gene symbol: Cdk1) coupled with Cyclin B1, **C** CK2a1 (casein kinase 2 alpha 1; gene symbol: Csnk2a1), and **D** EGFR (epidermal growth factor receptor, gene symbol: Egfr). Logos were generated in *PTM-Logo* using a Chi squared filtering α of 0.0001. Source: https://esbl.nhlbi.nih.gov/Databases/Kinase_Logos/). The colors of individual amino acids indicate different side chain properties: blue, basic; red, acidic; green, hydrophobic; black, aromatic; magenta, polar/uncharged; orange, no side chain

Selected examples of kinase logos and anti-logos are shown in Fig. 1. (A complete PDF file of all logos can be accessed in Data Supplement 1.) Fig. 1A shows the resulting logo (left) and anti-logo (right) for protein kinase A, i.e. cAMP-activated catalytic subunit alpha (Prkaca). Consistent with expectations, protein kinase A has a clear preference for basic amino acids (R/K) at the − 2, and − 3 positions, and more weakly at − 5, along with nonpolar amino acids at + 1. The anti-logo for protein kinase A is also distinctive, showing that proline is disfavored in the + 1 position along with basic residues downstream particularly at positions + 1,+ 2, and + 3. Figure 1B shows the logo/anti-logo for CDC2 (Cdk1), which demonstrates the classical proline in the + 1 position typical of many kinases of the Cyclin-dependent, Mitogen-activated, Glycogen synthase, and CDK-like kinase (CMGC) family. Like PKA, CDC2 also disfavors basic residues in + 1, but also aspartic acid (D) in + 3. Figure 1C shows the logo/anti-logo for an acidophilic protein kinase, casein kinase 2 alpha 1 (Csnk2a1) with the classical aspartic acid and glutamic acid moieties in position + 1 and + 3. Finally, Fig. 1D shows the logo/anti-logo for EGFR, a classical tyrosine kinase, and shows a general preference for acidic residues (D/E) at multiple positions surrounding the phosphorylation site. In addition, EGFR strongly disfavors the basic residue R in the + 1 position as shown on the anti-logo.

### Validation: comparison of logos between two kinase profiling studies

**Fig. 2:**
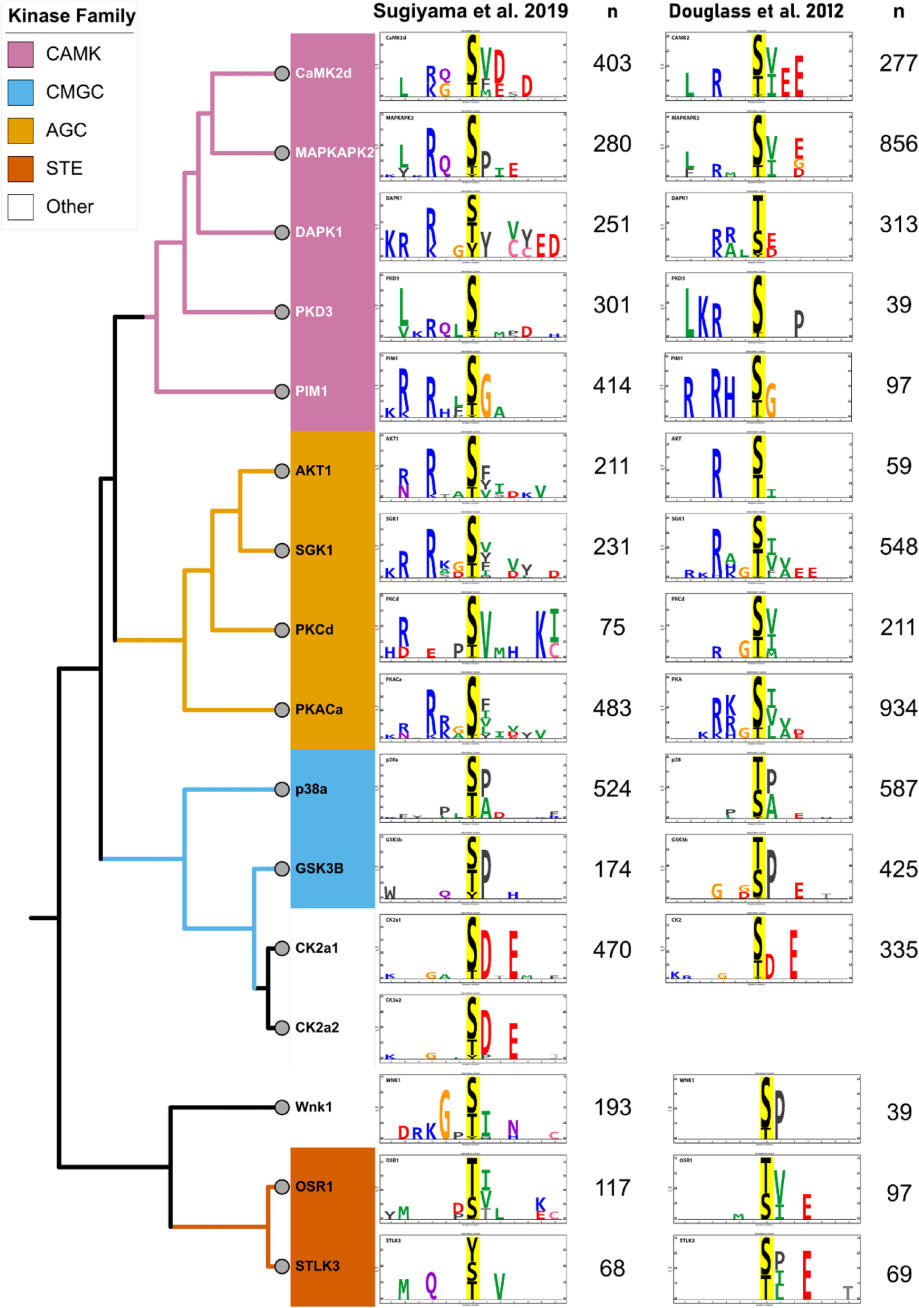
Comparison of substrate logos of representative kinases from two in vitro kinase profiling studies using MS-based methods, Sugiyama et al. 2019 [13] (left), and Douglass et al. 2012 [12] (right). Kinases are representatives from the indicated protein kinase families. Logos were generated in *PTM-Logo* (Chi squared filtering α = 0.0001) from “n” number of input peptide sequences for each kinase. Dendrograms were made using Interactive Tree of Life (iToL). The colors of individual amino acids indicate different side chain properties: blue, basic; red, acidic; green, hydrophobic; black, aromatic; magenta, polar/uncharged; orange, no side chain

The kinase profiling studies performed by Sugiyama et al. are in vitro phosphorylation studies using proteins from HeLa cells. To further validate the quality of the data, we compared representative kinases from the Sugiyama dataset with another kinase profiling dataset using a similar LC/MS technique performed using proteins isolated from a mixture of tissues from rats [12]. The comparison between these two studies is shown in Fig. 2. Kinases from multiple families are represented, including the CAMK kinases (CaMK2δ, DAPK1, MAPKAPK2, PKD3, and PIM1), AGC kinases (PKACα, AKT1, SGK, and PKCδ), CMGC kinases (p38α, and GSK3β), STE kinases (OSR1, and STLK3), and Others (CK2α1/2, and Wnk1). The sequence similarities between these kinases are shown in the context of a dendrogram, which is based on kinase catalytic subunit sequence alignment. Comparison between the two studies shows broad similarity of the substrate preferences, which largely conform to known substrate preferences within the different kinase families (e.g. proline in the + 1 position for the CMGC kinases, basophilic residues upstream of the phosphorylation site in the AGC kinases). However, as expected, this concordance seems to apply mainly to the high information content positions (large characters in logo) and the match is best with logos identified with larger numbers of phosphopeptides. In general, the identified substrate preferences appear to be independent of the tissue source, consistent with the idea that the preferences are properties of the specific kinases and not the population of target proteins. The similarity between the two studies highlights the robustness of the in vitro phosphorylation/phosphoproteomics technique.

**Fig. 3:**
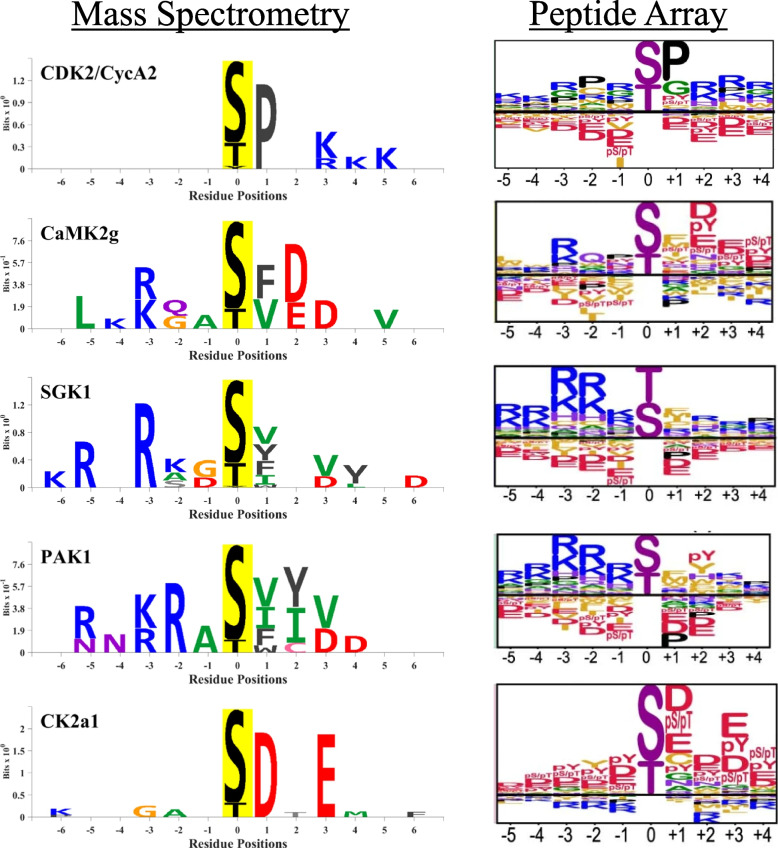
Comparison of substrate logos of representative kinases from two studies using different profiling methods: in vitro phosphorylation/phosphoproteomics technique (“Mass Spectrometry”) (left) and combinatorial peptide library screening method (“Peptide Array”) in which a library of target peptides is phosphorylated in vitro (right) [16]. Representative targets were chosen from different kinase families. In the logo images in the “Mass Spectrometry” column, the colors of individual amino acids indicate different side chain properties: blue, basic; red, acidic; green, hydrophobic; black, aromatic; magenta, polar/uncharged; orange, no side chain. The logo images in the “Peptide Array” column are from supplementary files from Johnson et al. [16] and use a similar format to what was used in the “Mass Spectrometry” column except that the anti-logos are given as downwardly directed stacks. These logos were copied directly from reference 14. (Reference 14 was licensed under a Creative Commons Attribution 4.0 International License, which permits use, sharing, adaptation, distribution and reproduction in any medium or format, as if appropriate credit is given to the original author(s) and the source. Creative Commons License: https://creativecommons.org/licenses/by/4.0/)

Figure 3 compares example logos from the in vitro phosphorylation/phosphoproteomics technique (“Mass Spectrometry”) to logos from combinatorial peptide library screening (“Peptide Array”) in which a library of target peptides are phosphorylated in in vitro arrays [16, 17]. In general, there is a high degree of concordance between the two methods. For example, logos from both methods for Cyclin-Dependent Kinase 2 (Cdk2) identified the known preference for proline in position + 1 and in addition a preference for basic amino acids in downstream positions, especially + 3. A comparison of logos from the two orthogonal methods can reveal preferences that may not be appreciated from one of the two methods alone. Similarly, logos from both data types for Calmodulin-Dependent Kinase 2 Gamma (Camk2g) identified the expected basic amino acids in position − 3, as well as concordant amino acids in position − 2 (Q), position + 1 (F) and position + 2 (D and E). Similar observations can be made with regard to the other three protein kinases displayed in Fig. 3. Thus, we conclude that comparison of logos derived from the two methods can in principle be used to generate sequence preference maps with a higher degree of confidence than with one method alone.

### Interactive phylogenetic kinase tree

**Fig. 4:**
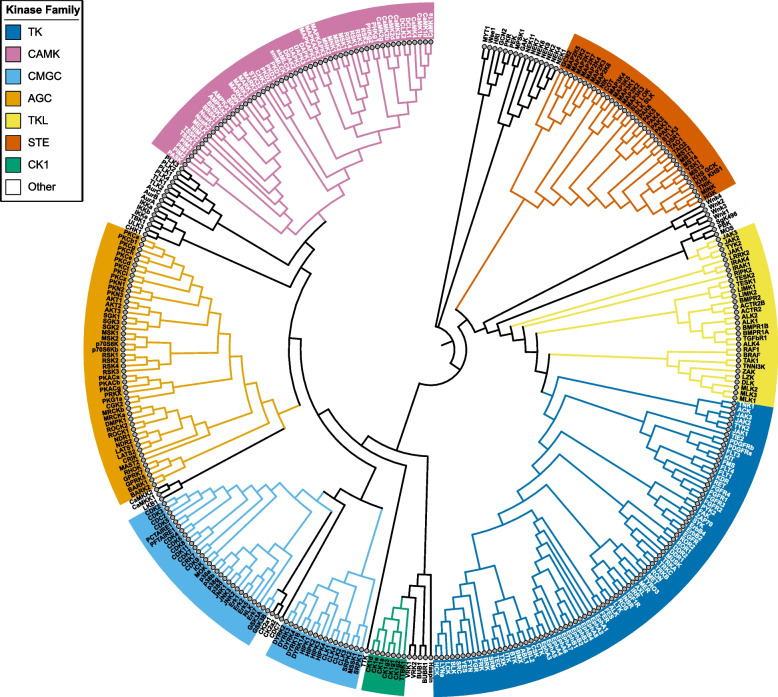
Phylogenetic tree of all protein kinases included in this database. The web version of this phylogenetic tree (https://esbl.nhlbi.nih.gov/Databases/Kinase_Logos/KinaseTree.html) has their respective kinase logos viewable as popups when the user hovers over each node. Kinase groups are AGC, PKA/PKG/PKC family; CAMK, calmodulin-kinase family; CK1, casein kinase family; CMGC, cyclin-dependent kinase/mitogen-activated kinase/glycogen-synthase kinase/CDK-like kinase family; STE, sterile family kinases; TKL, tyrosine-like kinases, and atypical kinases

To gain additional insight into the structural basis for kinase specificity, we cross-referenced the kinase sequence logos from our database onto a kinase phylogenetic tree (Fig. 4). We used an alignment of protein kinase catalytic subunits that has been previously published by Manning et al. [1]. After curating the alignment for the kinases present in the Sugiyama dataset [13], a phylogenetic tree interactive image was created using Interactive Tree of Life (iToL) [15]. This interactive phylogenetic tree is made available to users at https://esbl.nhlbi.nih.gov/Databases/Kinase_Logos/KinaseTree.html. Users can hover over each node of the phylogenetic tree to display the logo for each kinase. Organizing the data in this way allows easy visualization of how the sequence similarity of kinase catalytic regions can translate to similarity in substrate specificity.

### *KinasePredictor*

**Fig. 5:**
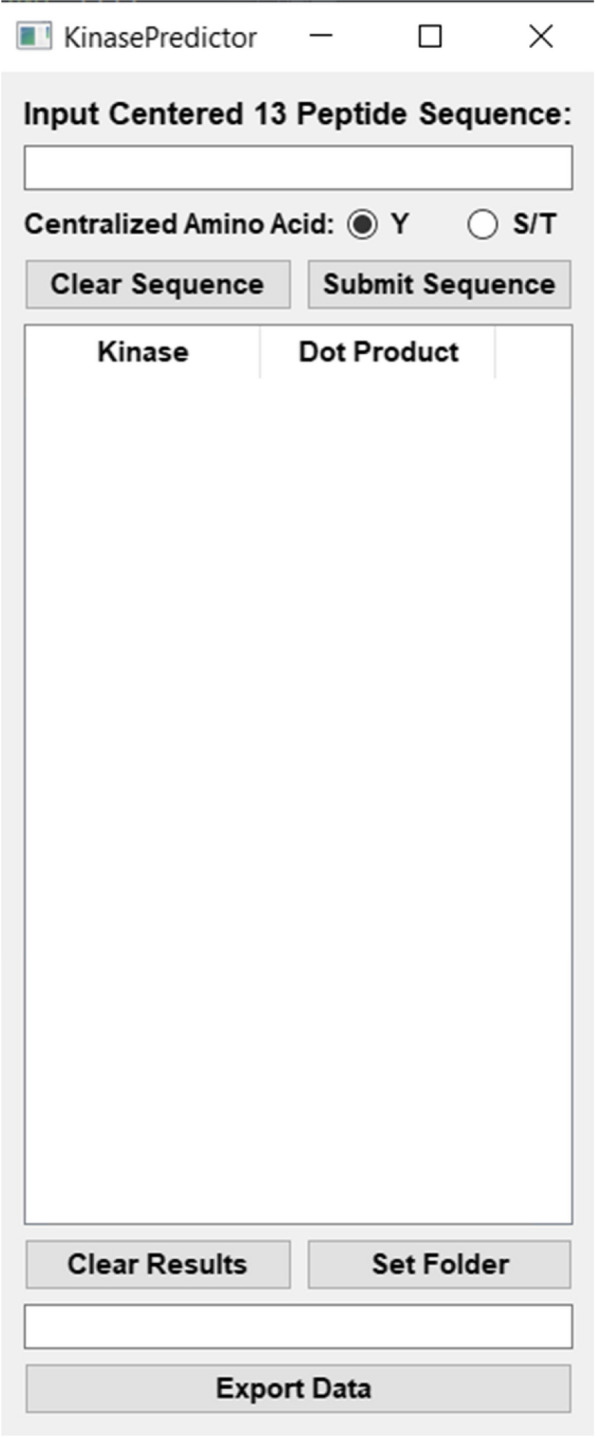
The *KinasePredictor* interface for inputting user phosphorylation sequences. Users input a centralized 13 amino-acid sequence in the top entry box, and the resulting ranked list of kinases are shown below with their respective dot-product scores. These ranked kinase results can then be saved and exported for further analysis

Using the Sugiyama data, we created an algorithm that can take any centralized 13-amino-acid sequence with a single phosphorylated site and rank-order all kinases with regard to degree-of-fit to their kinase target logos, called *KinasePredictor* (Fig. 5). *KinasePredictor* is available as a stand-alone program and is available for download at https://esbl.nhlbi.nih.gov/Databases/Kinase_Logos/KinasePredictor.html. The *Python* source code is also made available on the same page. For each kinase, *KinasePredictor* calculates a scalar product (“dot product”) from two matrices. The first matrix is a Boolean matrix (i.e. composed of 1’s and 0’s) of dimension 13 (positions) by 20 (amino acids) that represents the input 13-mer. The second matrix is an *information content matrix* of the same dimensions for each protein kinase derived through application of *PTM-Logo* from the mass spectrometry data from Sugiyama et al. [13] calculated as described by Leo et al. [18]. The dot product for each kinase is the sum of products of the corresponding elements of the two matrices over all positions. *KinasePredictor* outputs a rank-ordered list of the protein kinases and their associated dot product values. Thus, the program can give a ranked list identifying ‘best-fit’ kinases that could be responsible for a given phosphorylation event. A similar predictor, “Score Site” in the “Kinase Library” has been recently introduced (https://kinase-library.phosphosite.org/site) [16].

**Fig. 6:**
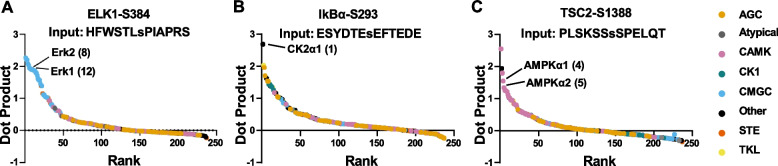
*KinasePredictor* validation using well-established phosphorylation sites. Three sequences with experimentally verified phosphorylation sites from PhosphoSitePlus were input into *KinasePredictor*, ELK1 at S384, a known target of Erk1/2 (**A**, left), IκB1α at S293, a known target of CK2α1 (**B**, mid), and TSC2 at S1388, a known target of AMPKα1/2 (**C**, right). For each sequence, a dot product was calculated for each kinase in the dataset, ranked, and then graphed with their dot product value. The lowest rank and highest dot product kinases represent those predicted most likely to phosphorylate the target site given the input sequence. The kinases known to target the site are indicated on each graph and their rank shown in parentheses. Kinases are color-coded by their family according to the legend on the right. Kinase groups are AGC, PKA/PKG/PKC family; CAMK, calmodulin-kinase family; CK1, casein kinase family; CMGC, cyclin-dependent kinase/mitogen-activated kinase/glycogen-synthase kinase/CDK-like kinase family; STE, sterile family kinases; TK, tyrosine kinases; TKL, tyrosine-like kinases

To validate the *KinasePredictor* algorithm, we input the centralized amino acid sequences for three well studied phosphorylation sites in which the protein kinase is known (Phosphosite Plus) [9] (Fig. 6). The centralized sequences were fed into *KinasePredictor* and a ranked list of predicted kinases was generated for each site, the results of which are shown graphically. The three sites shown here are for ELK1, which is phosphorylated by Erk1 and Erk2 at S384 (mouse numbering) [19] (Fig. 6A), I-κB1α, phosphorylated by CK2a1 at S293 [20] (Fig. 6B), and tuberin (TSC2), phosphorylated by AMPK at S1388 [21] (Fig. 6C). For ELK1 at S384, the known kinases Erk1 and Erk2 were ranked 8th and 12th, respectively among 237 possible S/T kinases. All of the surrounding kinases were from the CMGC family which have preference for proline (P) in position + 1 (light blue points) with the top ranked kinase being p38γ. For I-κB1α at S293, the known kinase, CK2α1, was ranked first. For TSC2 at S1388, known kinases AMPKα1 and AMPKα2 were ranked 4th and 5th, respectively. A closely related protein kinase BRSK1 was ranked first. Overall, these results illustrate the utility of *KinasePredictor* in predicting protein kinases that could be responsible for specific phosphorylation events. Although the tool does not distinguish strongly between top-ranked protein kinases, other types of data can be used to refine the prediction such as the relative abundances of the candidate kinases in a specific tissue and possible differences in kinases with regard to subcellular localization vis-à-vis the proposed substrate.

## Discussion

In this paper, we present three new tools for identifying candidate protein kinases corresponding to phosphorylation sites identified in cell signaling studies. Kinase preferences are represented as ‘logos’ that are specified by an information-theory based algorithm, *PTM-Logo*. These tools are provided in the form of online resources that allow visualization of kinase substrate preferences across the majority of the eukaryotic protein kinome, and allows user to interact with the data to aid in hypothesis generation and validation of their own experimental data.

*PTM-Logo* displays predicted amino acid preferences as single letter amino acid designators whose height is proportional to the position-specific information content while simultaneously filtering the output to remove noisy predictions. For all the logos in our resource, we used the same filtering parameter (Chi-square filtering α) for uniformity. We also chose a relatively non-stringent value of α (α = 0.0001) to avoid filtering out relevant predictive information. We have provided all of the tools and sequences used in supplementary files and web pages, allowing users to further refine the calculation for their kinase of interest if desired.

The basis of this resource was a large dataset published by Sugiyama et al. [13], which used protein mass spectrometry to identify phosphorylation events resulting from in vitro incubation of dephosphorylated proteins with individual recombinant protein kinases. The original paper provided the data, but did not provide a practical, user-friendly means of using the data for modeling of signaling systems. The Sugiyama data set was in part validated by comparison of findings using phosphorylation data from in vitro incubation with the same recombinant kinases in an earlier study by our laboratory [12] (Fig. 2). In general, the logos from the two studies were similar, and conformed to the general groupings of kinase target preferences, either for upstream basophilic residues (e.g PKA), downstream acidophilic residues (e.g. CK2a), or proline-directed kinases (e.g. p38α). The logos from the two studies were most similar for amino acid positions that show the highest information content. Consequently, we judged the Sugiyama data set to be appropriate for general use with one important caveat. Specifically, the quality of the logos appeared to be highly dependent on the number of phosphopeptide sequences identified for a given kinase in the Sugiyama data set and used for input into *PTM-Logo*. Consequently, we required at least *n* ≥ 30 input sequences, although the quality of the logos improved for kinases with an even greater number of input sequences as judged by the number of low information amino acids represented in the logo. Users should take note of the number of input sequences for each kinase when judging how to use the derived logos.

Our stand-alone prediction program, *KinasePredictor*, can create a ranked list of kinases that are likely to phosphorylate a given site. *KinasePredictor* only takes into account the match between position-specific amino acid preferences and observed phosphorylation sites to rank the kinases. Such preferences provide only part of the data needed for accurate predictions in model building. The output from *KinasePredictor* can be combined with other types of information, such as relative kinase abundances and localization inside the cell, using formalized Bayesian integration methods such as was carried out for two sites in aquaporin-2, Ser256 and Ser261 [22, 23] and more generally, for sites phosphorylated in response to vasopressin [18]. These Bayesian studies rank kinases based on sequential application of Bayes’ Theorem to calculate probabilities for each kinase in the mammalian kinome using transcriptomic data, proteomic data, existing literature data, and target sequence matches to provide a more precise ranking of kinases than could be achieved through sequence matching alone.

One important feature of the present paper is our mapping of kinase preference logos to the Manning phylogenetic tree based on sequence similarities of the catalytic domains of individual kinases (https://esbl.nhlbi.nih.gov/Databases/Kinase_Logos/KinaseTree.html). Although most protein kinases have a high degree of conserved structural similarity, changes in charge or hydrophobicity among the surface residues of the catalytic domain are key for kinase specificity [24, 25]. Other kinome tree visualization resources, such as KinMap [26], can be used for integration with biochemical and structural databases. However, our resource directly links to our logo database for direct comparison of substrate preferences. One thing that is obvious from examination of the full tree architecture is that related kinases have very similar target preferences, which makes it impossible to identify a specific kinase based on a single phosphorylation target sequence. For example, most CMGC kinases such as Erk1 or Erk2 display the classical proline-directed motif (P at + 1). On the other hand, the PKC subfamily of kinases have a largely ill-defined motif, despite high sequence similarity of their catalytic domains. These structural insights have implications for designing novel pharmacological tools targeting these kinases. Very few, if any, protein kinase inhibitors are specific to a single protein kinase as established by data viewable at the *Kinase Profiling Inhibitor Database* (https://www.kinase-screen.mrc.ac.uk/kinase-inhibitors). Resources are available at sites that curate kinase structural data in depth, such as *Kincore* [27].

### Comparison with other kinase prediction sites

As the identification of kinase-substrate interactions is a common goal, both in vivo and in vitro techniques have been previously developed to profile individual kinases. In addition to the method used in this study (mass spectrometry of phosphorylation resulting from in vitro incubation of mixtures of dephosphorylated proteins with recombinant kinases), additional approaches include target peptide library arrays [16, 17] and phosphoproteomic analysis of tissues after deletion or overexpression of individual kinases [28, 29], and phosphoproteomic analysis of tissues after incubation with kinase inhibitors thought to be relatively selective for a particular kinase. However, much of what we know about kinase-substrate preferences is based on reductionist studies. Each of these approaches have added valuable information and users of the tools introduced in this paper may benefit from consideration of data from all methodologies. Over the past few years, multiple resources have been established to map phosphorylation sites to individual kinases, such as *Phosida* [5], *PhosphoNet* [6], *Phosphomotif Finder* [7], *SCANSITE* [8], *PhosphoSitePlus* [9] and several others. These databases extract data from the literature, in most cases from reductionist experiments. However, many of these resources do not provide a comprehensive list of kinases and their motifs, and many of the predictions in these online databases are the result of experimental observations and not unbiased high throughput datasets. In addition to the *KinasePredictor* algorithm presented here, several computational resources have been developed to predict kinases based on target sequence preferences. Much of what we know about kinase-substrate preference is still reliant on targeted reductionist studies, and these tools are generally trained using existing databases that draw from this literature. Thus, many protein kinases are underrepresented in these databases.

During the preparation of this manuscript, similar tools have been described for analysis of sequence preference data from a different source, viz. combinatorial peptide library screening experiments [16]. As shown in Fig. 3, data from the in vitro phosphorylation/ phosphoproteomics technique and combinatorial peptide library screening method identify very similar sequence preferences, but may also provide complementary information. Consequently, we propose that the best predictions may be derived from use of both types of data and both sets of tools.

### Limitations

Our resources provide a user-friendly and unique set of kinase target preference logos calculated from in vitro kinase profiling studies. However, the data do have limitations that should be kept in mind when applied to modeling of signaling systems. First, in vitro phosphorylation may identify some phosphorylation sites that may not be observed in vivo due to lack of interaction or low substrate affinity. Thus, individual phosphorylation sites observed in the Sugiyama et al. dataset may not be *bonafide* targets of the relevant kinases in all tissues. For example, of the 20,669 phosphosites mapped to kinases by Sugiyama et al., only 4913 phosphosites match with those obtained by an ultra-deep phosphoproteomic study of HeLa cells reported by Sharma et al. [3]. As emphasized above, variables such as co-expression and co-localization must always be taken into account with any predictive analysis as carried out by Leo et al. [18] using Bayesian integration methods. Additionally, this dataset cannot account for phosphorylation sites that may require the binding of another protein or prior phosphorylation by another kinase in order to be favorable [24].

### Final overview

Here we present new resources to visualize and interact with the human kinome and curation of a large-scale dataset describing kinase-substrate preferences. This set of resources provides a user-friendly way to explore phosphorylation networks and aid in kinase-substrate predictions. This represents another tool to increase the accuracy of predicting kinases in modeling of signaling systems and for prediction of kinases involved in novel phosphorylation events.

### Supplementary Information

**Additional file 1.**

**Additional file 2.**

## Data Availability

The datasets generated and/or analysed during the current study are available online at the following web links: https://esbl.nhlbi.nih.gov/Databases/Kinase_Logos/ https://esbl.nhlbi.nih.gov/Databases/Kinase_Logos/KinaseTree.html https://esbl.nhlbi.nih.gov/Databases/Kinase_Logos/KinasePredictor.html
