# Supplementary material for: A resource database for protein kinase substrate sequence-preference motifs based on large-scale mass spectrometry data
Source: Cell Commun Signal. 2024 Feb 19;22:137. doi: 10.1186/s12964-023-01436-2 (PMC10875805; doi:10.1186/s12964-023-01436-2)
Supplement: Supplementary file 3 — Additional file 2. [file 12964_2023_1436_MOESM2_ESM.pdf]

Protein Kinase Sequence Motifs

Downloaded from https://esbl.nhlbi.nih.gov/Databases/Kinase_Logos/

Kinase data curated from Sugiyama et al. (PMID: 31324866). Sequence motifs for each protein kinase showing amino acid preferences flanking the post-translational modified site were identified by PTMLogo. All logos were generated from a minimum of 30 amino acid sequences, using Chi-squared filtering alpha = 0.0001. Exact number of reference sequences used for each kinase can be found at the link above. PTMLogo published by Saethang et al. 2019, PMID: 31318409.

Amino acids are color coded based on their properties. **L, I, M, V, A** - green. **K, R, H** – blue, **Q, N** - purple, **D, E** - red, **F, W, Y, P** - dark grey, **S, T** - light grey, **C** - pink, **G** - orange.

B represents the N-terminus and **J** represents the C-terminus.

Created by Brian Poll, Kirby Leo, and Mark A. Knepper in the Epithelial Systems Biology Laboratory at the National Heart, Lung and Blood Institute as part of its Kidney Systems Biology Project. Please contact Mark Knepper with any comments or questions.

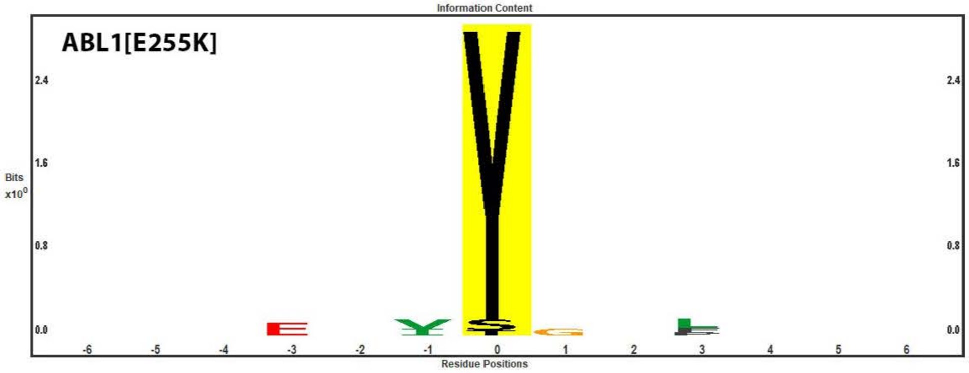
Information Content
ABL1[E255K]
Bits x10^0
2.4
1.6
0.8
0.0
-6 -5 -4 -3 -2 -1 0 1 2 3 4 5 6
Residue Positions

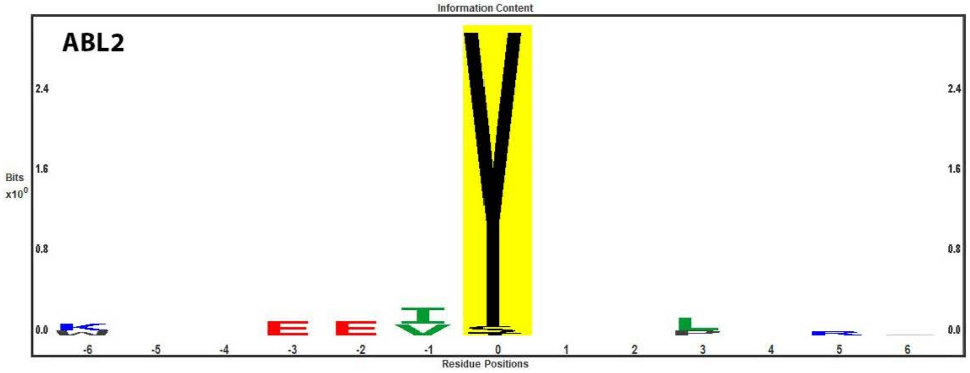
Information Content
ABL2
Bits x10^0
2.4
1.6
0.8
0.0
-6 -5 -4 -3 -2 -1 0 1 2 3 4 5 6
Residue Positions

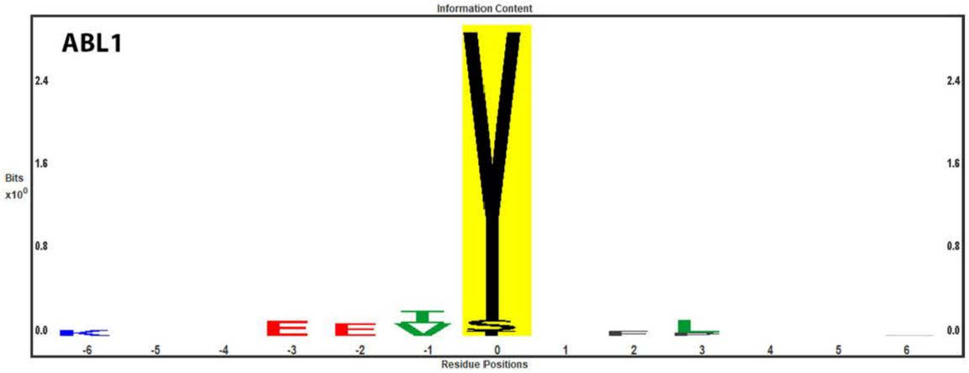
Information Content
ABL1
Bits x10^0
2.4
1.6
0.8
0.0
-6 -5 -4 -3 -2 -1 0 1 2 3 4 5 6
Residue Positions

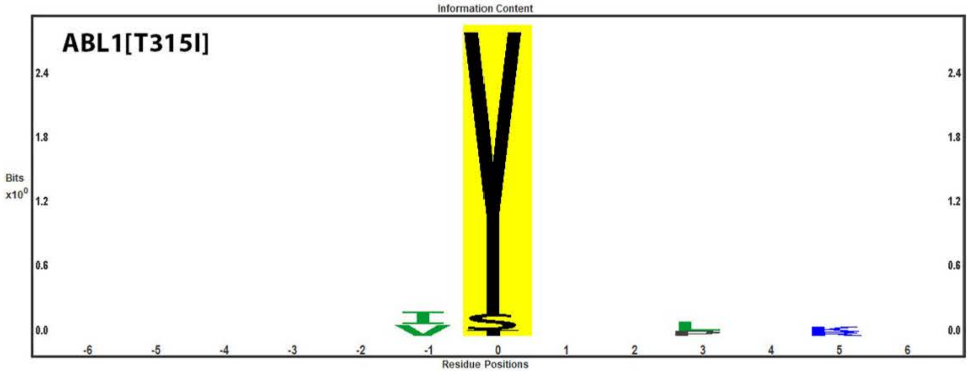
Information Content
ABL1[T315I]
Bits x10^0
2.4
1.8
1.2
0.6
0.0
-6 -5 -4 -3 -2 -1 0 1 2 3 4 5 6
Residue Positions

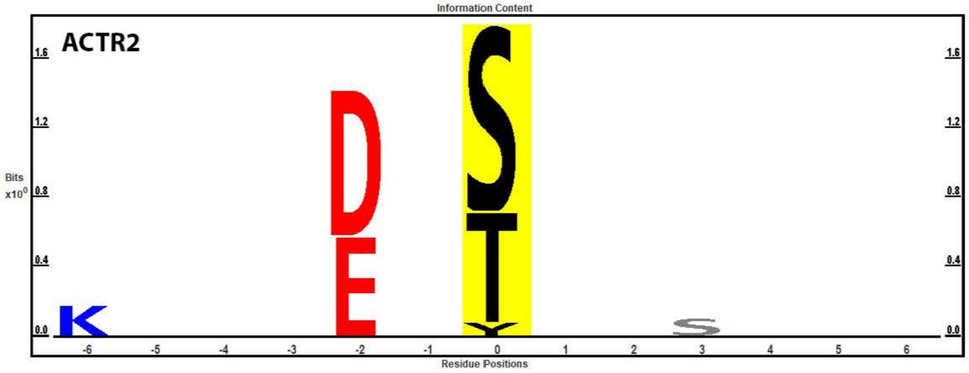
Information Content
ACTR2
Bits
x10^0
Residue Positions

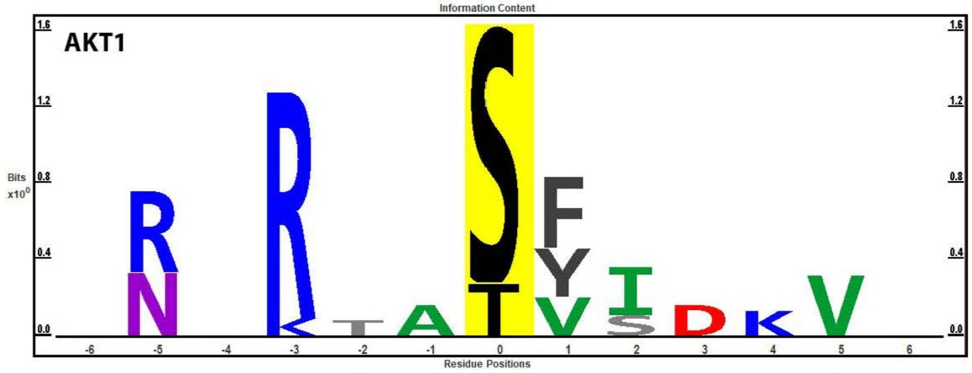
Information Content
AKT1
Bits
x10^0
Residue Positions

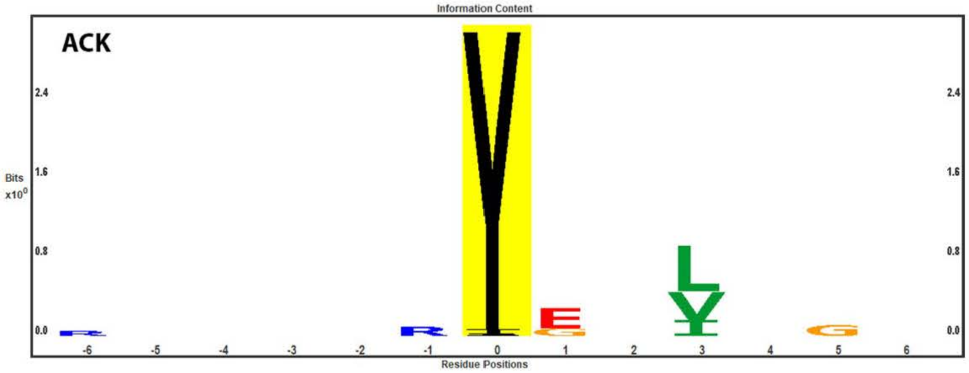
Information Content
ACK
Bits
x10^0
Residue Positions

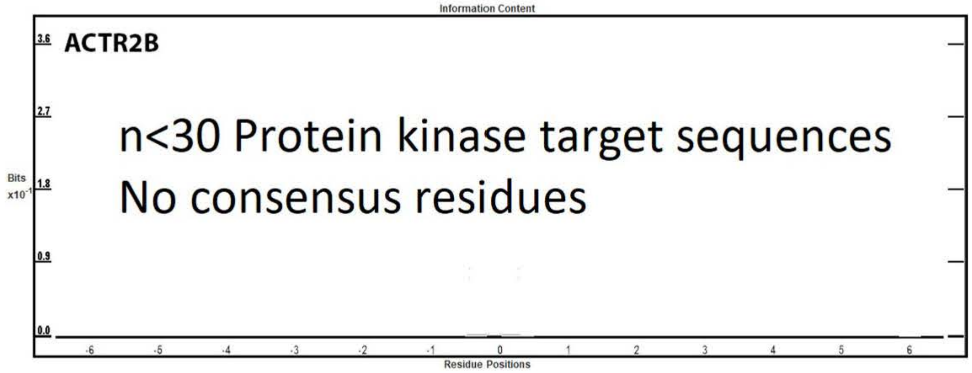
Information Content
ACTR2B
n<30 Protein kinase target sequences
No consensus residues
Bits
x10^-1
Residue Positions

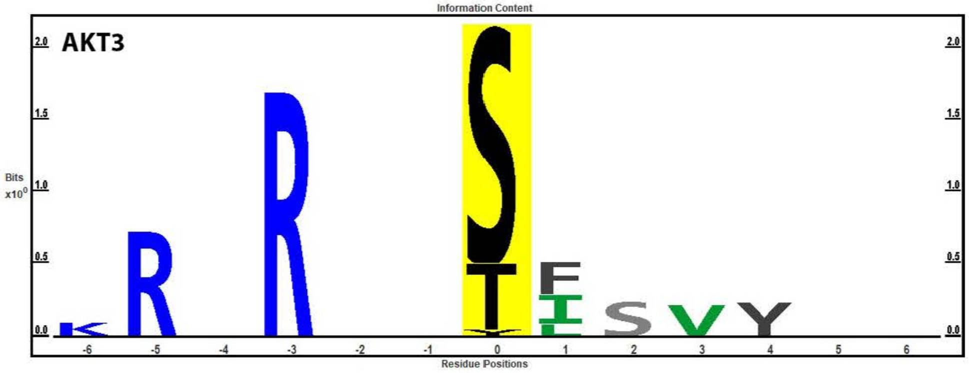
Information Content
AKT3
Bits
x10^0
0.0 0.5 1.0 1.5 2.0
-6 -5 -4 -3 -2 -1 0 1 2 3 4 5 6
Residue Positions

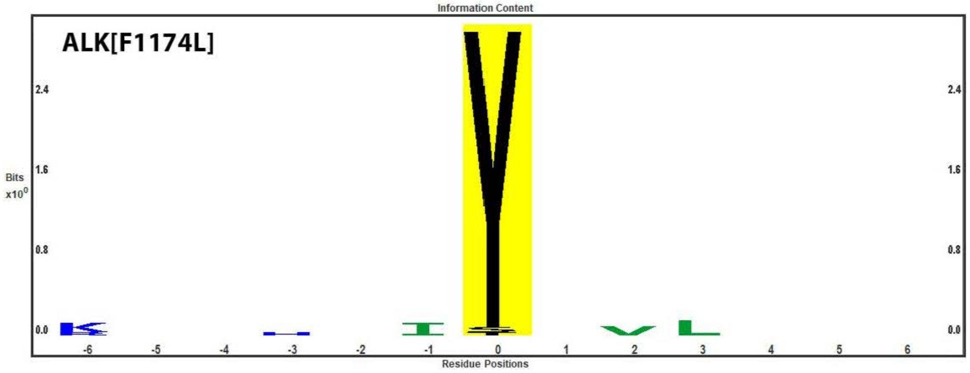
Information Content
ALK[F1174L]
Bits
x10^0
0.0 0.8 1.6 2.4
-6 -5 -4 -3 -2 -1 0 1 2 3 4 5 6
Residue Positions

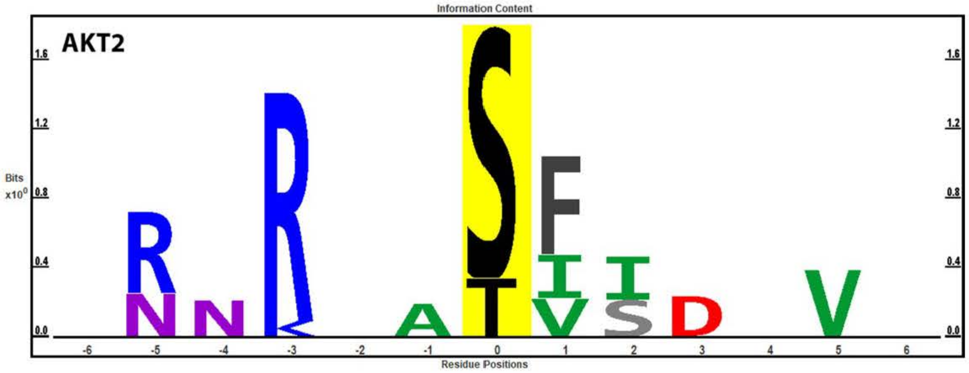
Information Content
AKT2
Bits
x10^0
0.0 0.4 0.8 1.2 1.6
-6 -5 -4 -3 -2 -1 0 1 2 3 4 5 6
Residue Positions

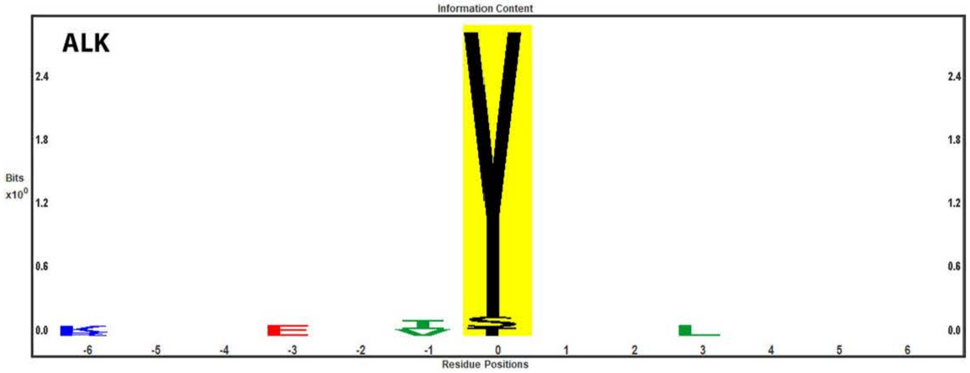
Information Content
ALK
Bits
x10^0
0.0 0.6 1.2 1.8 2.4
-6 -5 -4 -3 -2 -1 0 1 2 3 4 5 6
Residue Positions

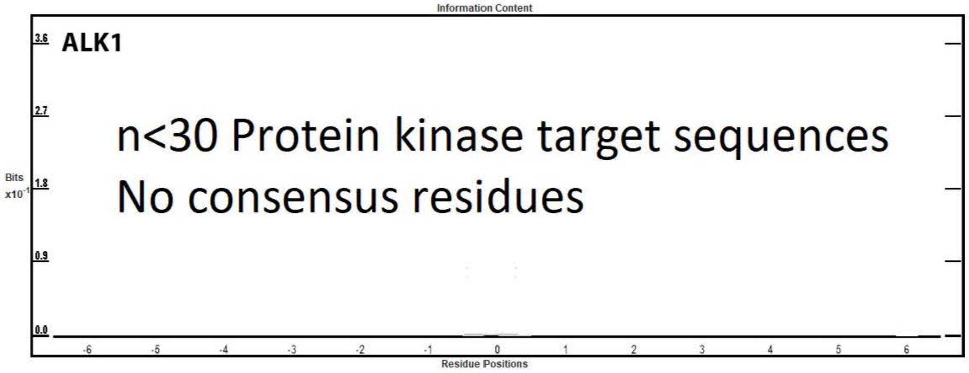
Information Content
ALK1
n<30 Protein kinase target sequences
No consensus residues
Bits
x10^-1
Residue Positions

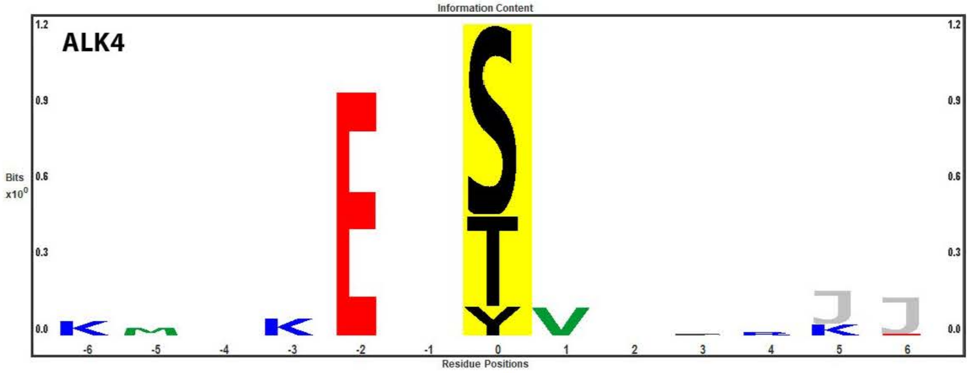
Information Content
ALK4
Bits
x10^0
Residue Positions

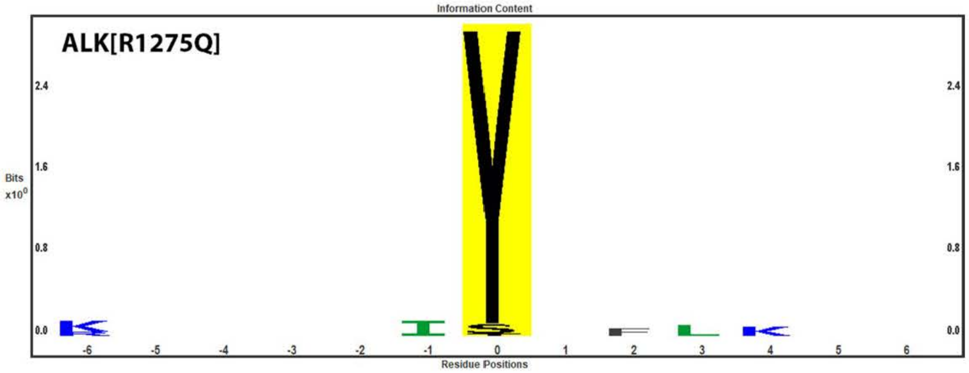
Information Content
ALK[R1275Q]
Bits
x10^0
Residue Positions

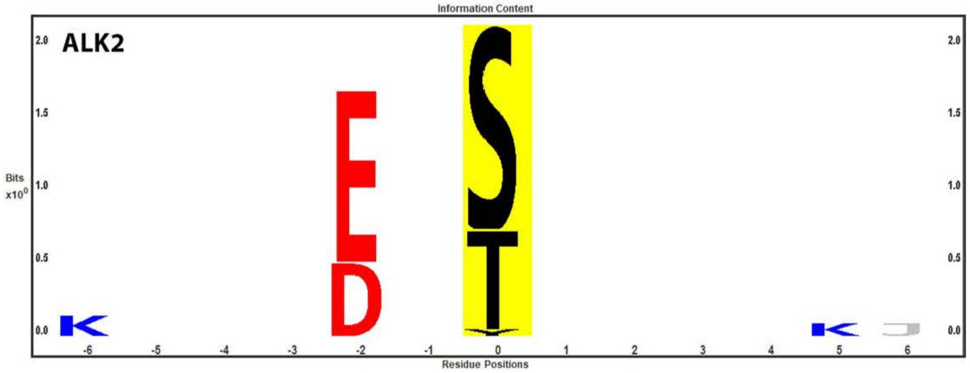
Information Content
ALK2
Bits
x10^0
Residue Positions

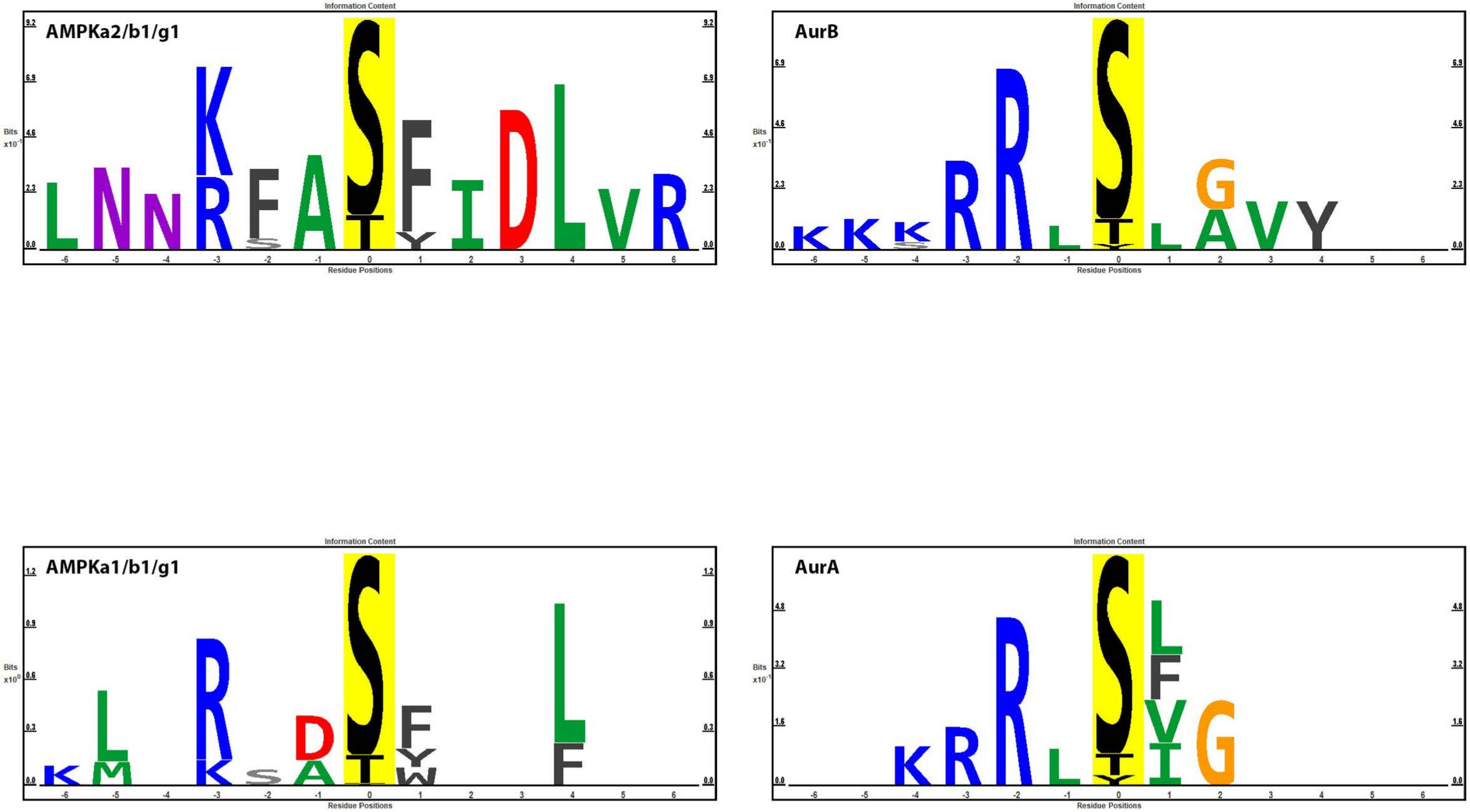
AMPKa2/b1/g1
AurB
AMPKa1/b1/g1
AurA
Information Content
Bits
Residue Positions

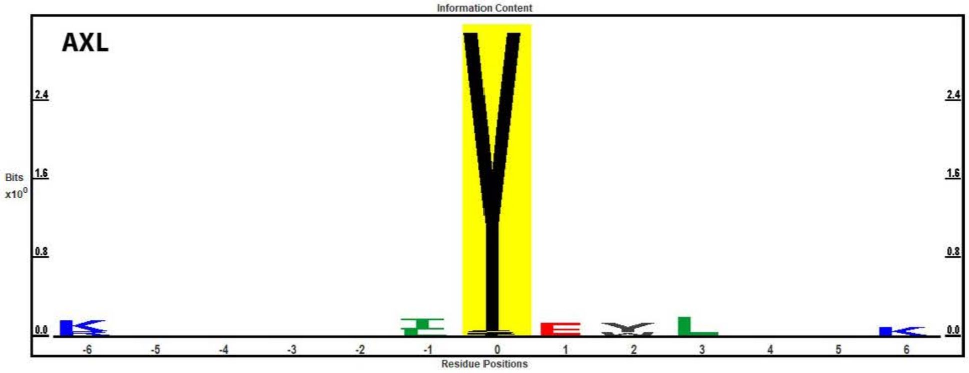
Information Content
AXL
Bits
x10^0
Residue Positions

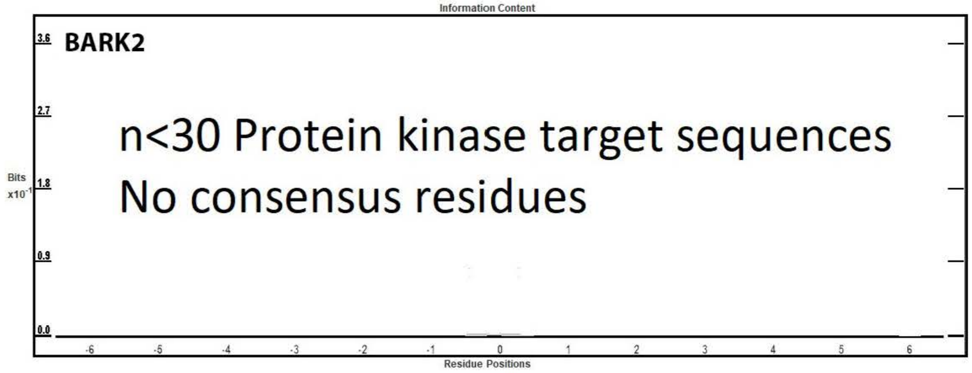
Information Content
BARK2
n<30 Protein kinase target sequences
No consensus residues
Bits
x10^-1
Residue Positions

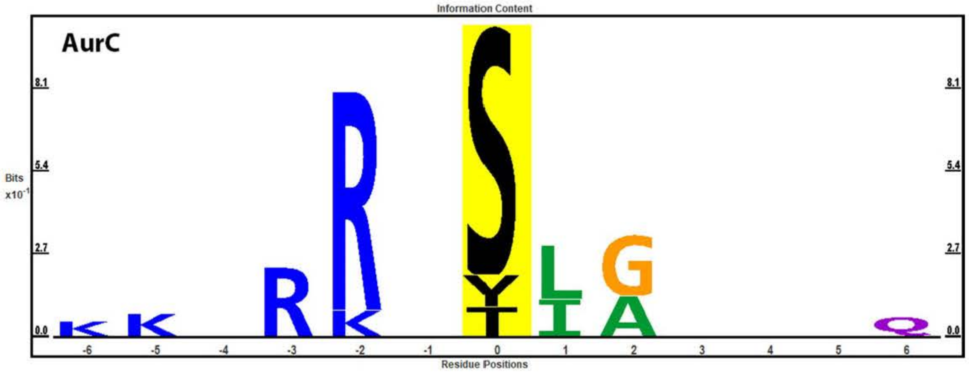
Information Content
AurC
Bits
x10^-1
Residue Positions

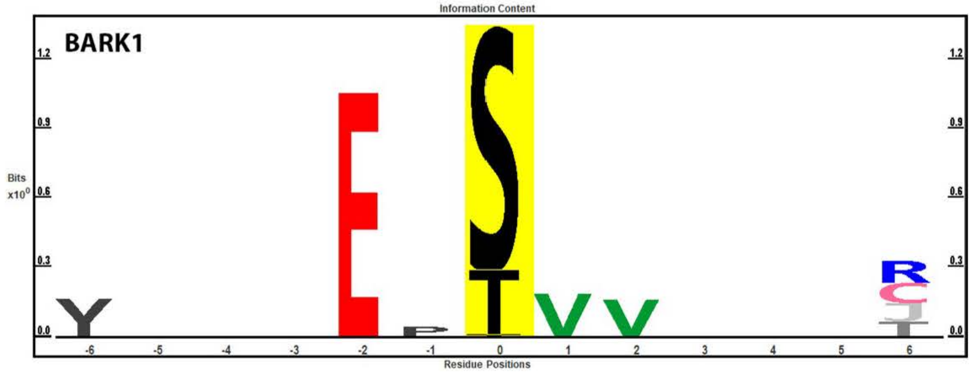
Information Content
BARK1
Bits
x10^0
Residue Positions

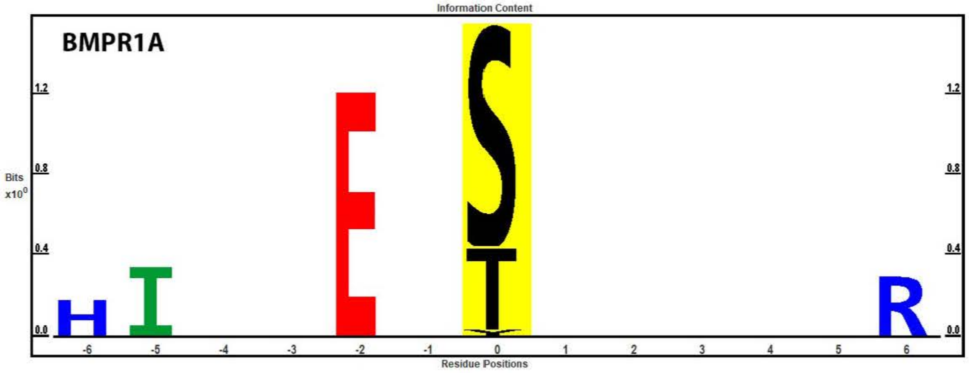
Information Content
BMPR1A
Bits
x10^0
Residue Positions

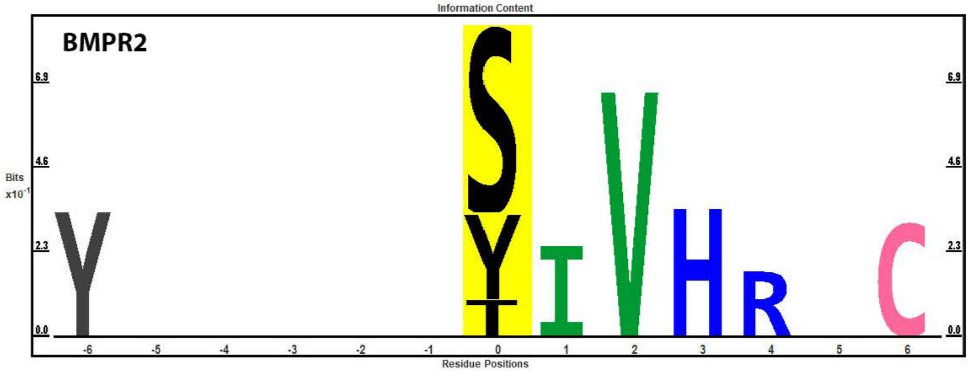
Information Content
BMPR2
Bits
x10^-1
Residue Positions

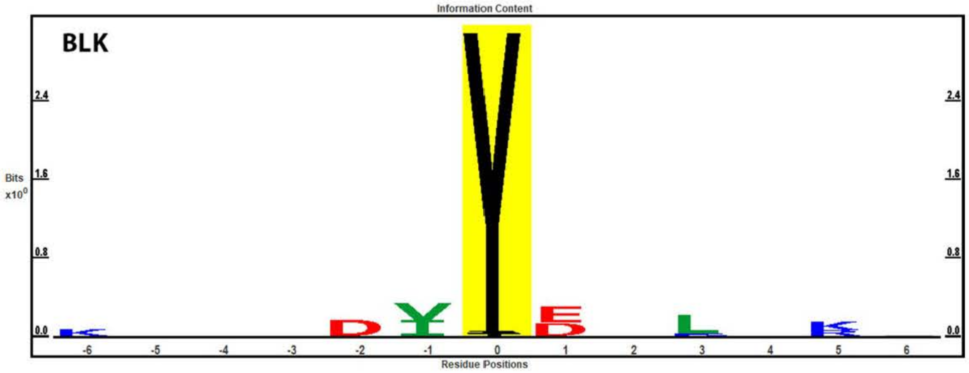
Information Content
BLK
Bits
x10^0
Residue Positions

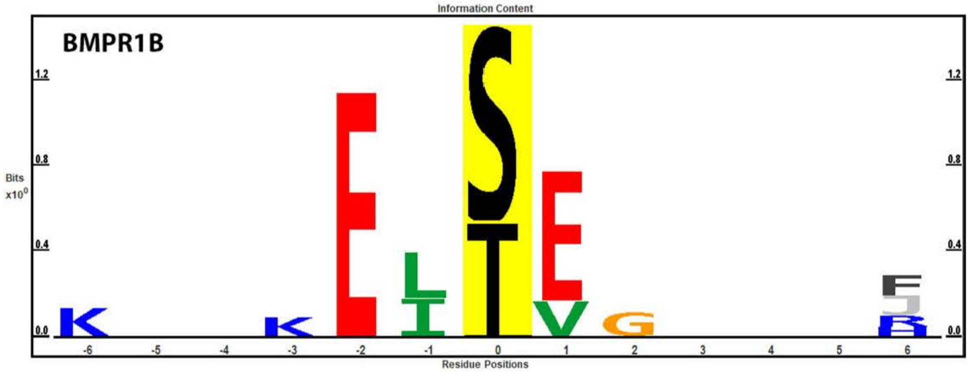
Information Content
BMPR1B
Bits
x10^0
Residue Positions

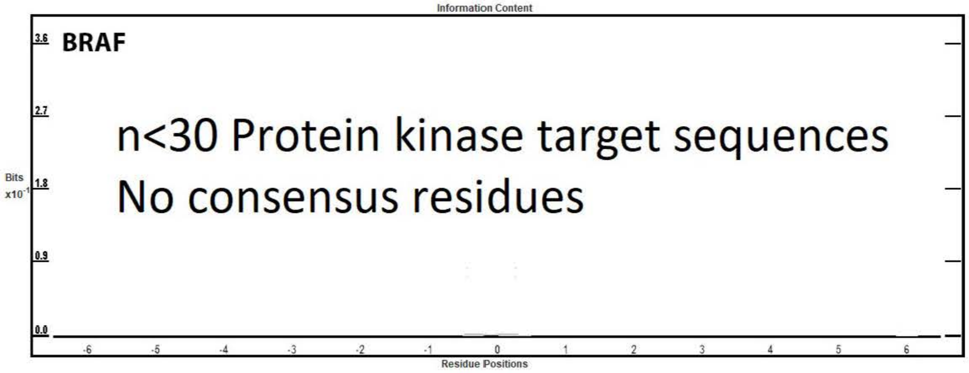
Information Content
BRAF
n<30 Protein kinase target sequences
No consensus residues
Bits
Residue Positions

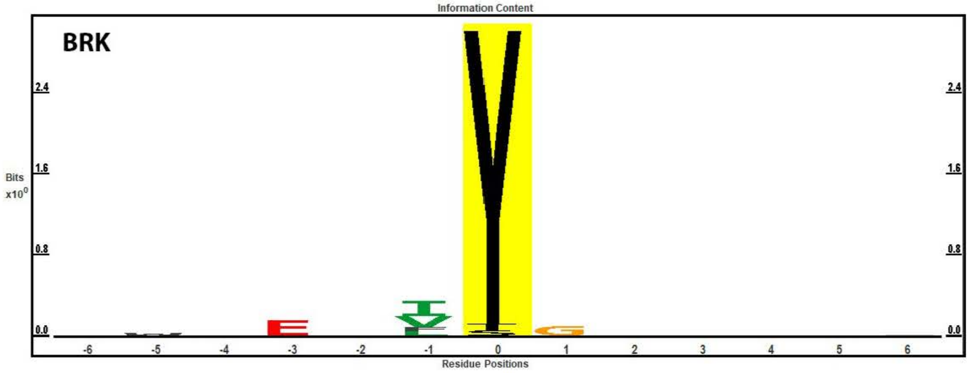
Information Content
BRK
Bits
Residue Positions

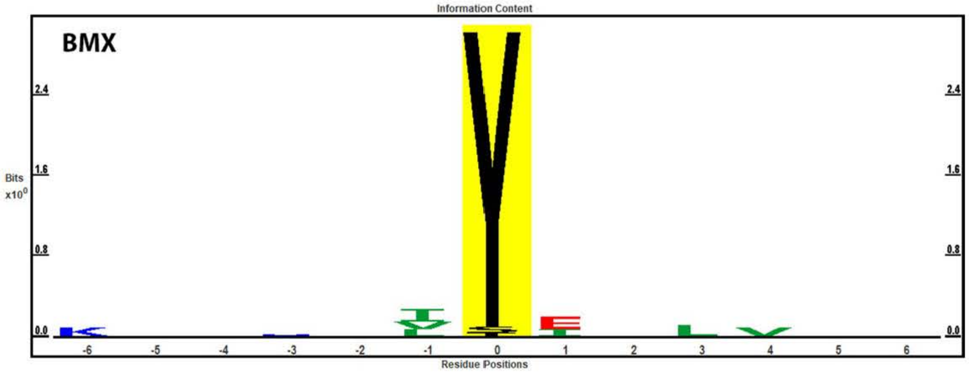
Information Content
BMX
Bits
Residue Positions

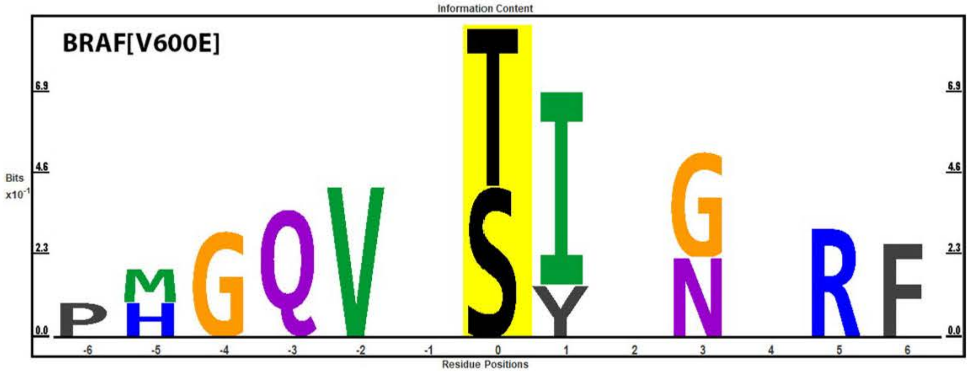
Information Content
BRAF[V600E]
Bits
Residue Positions

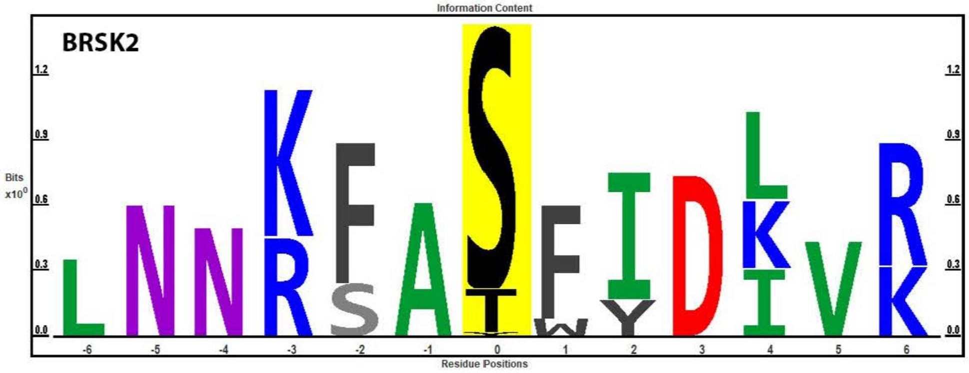

Information Content
BRSK2
Bits
x10^0
1.2
0.9
0.6
0.3
0.0
-6 -5 -4 -3 -2 -1 0 1 2 3 4 5 6
Residue Positions

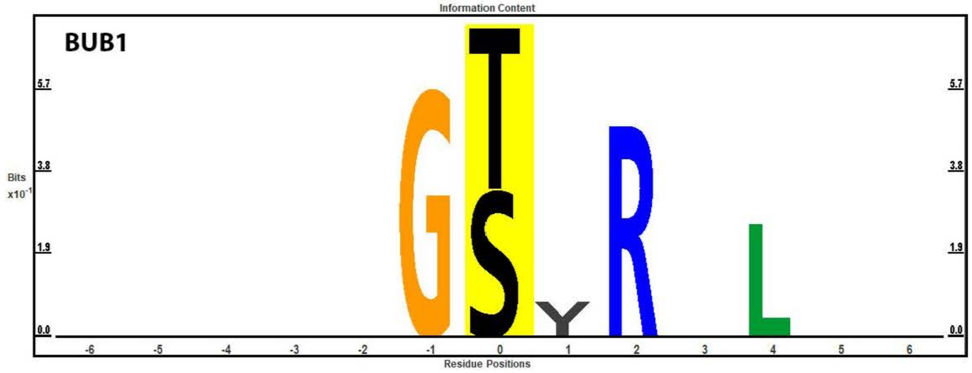

Information Content
BUB1
Bits
x10^-1
5.7
3.8
1.9
0.0
-6 -5 -4 -3 -2 -1 0 1 2 3 4 5 6
Residue Positions

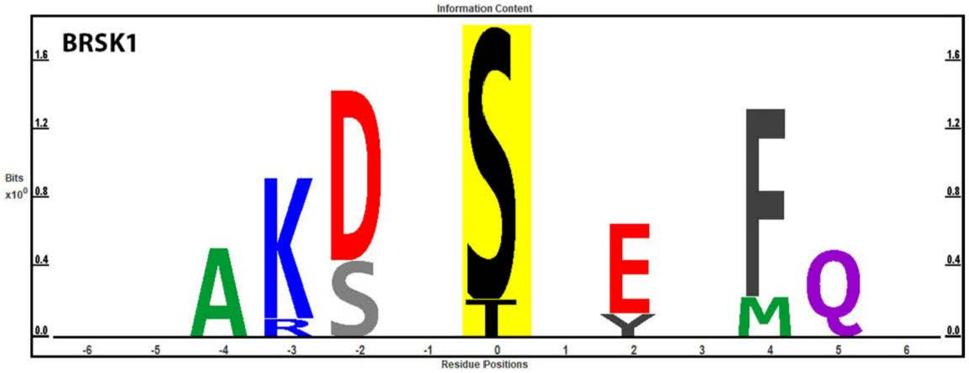

Information Content
BRSK1
Bits
x10^0
1.6
1.2
0.8
0.4
0.0
-6 -5 -4 -3 -2 -1 0 1 2 3 4 5 6
Residue Positions

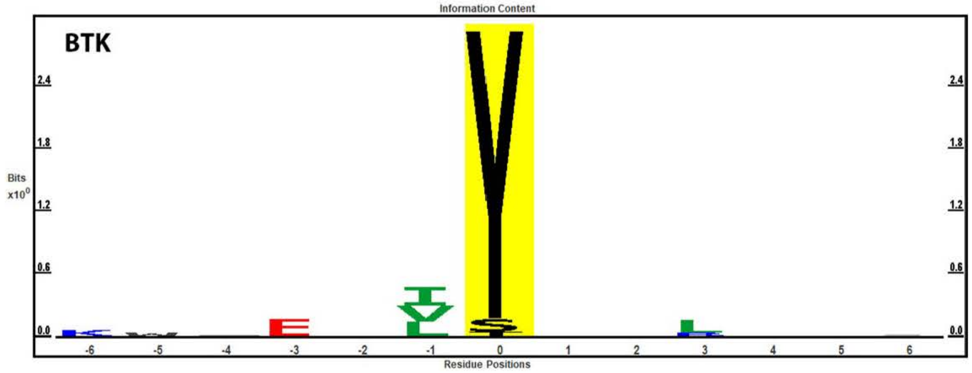

Information Content
BTK
Bits
x10^0
2.4
1.8
1.2
0.6
0.0
-6 -5 -4 -3 -2 -1 0 1 2 3 4 5 6
Residue Positions

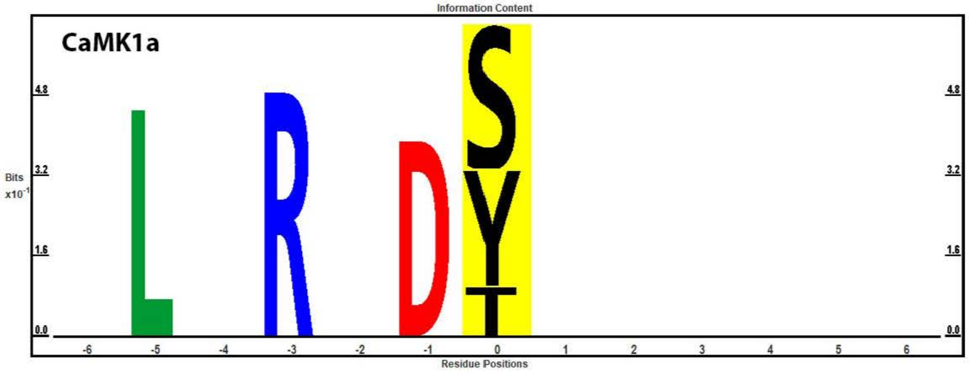
Information Content
CaMK1a
Bits
x10-1
4.8
3.2
1.6
0.0
-6 -5 -4 -3 -2 -1 0 1 2 3 4 5 6
Residue Positions

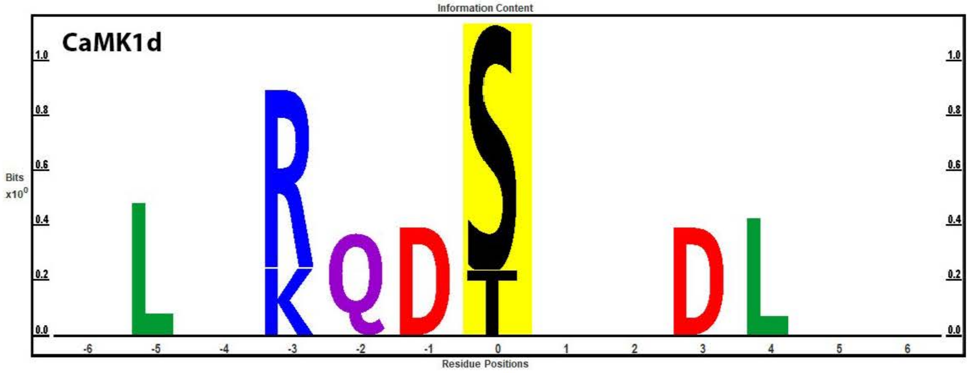
Information Content
CaMK1d
Bits
x10^0
1.0
0.8
0.6
0.4
0.2
0.0
-6 -5 -4 -3 -2 -1 0 1 2 3 4 5 6
Residue Positions

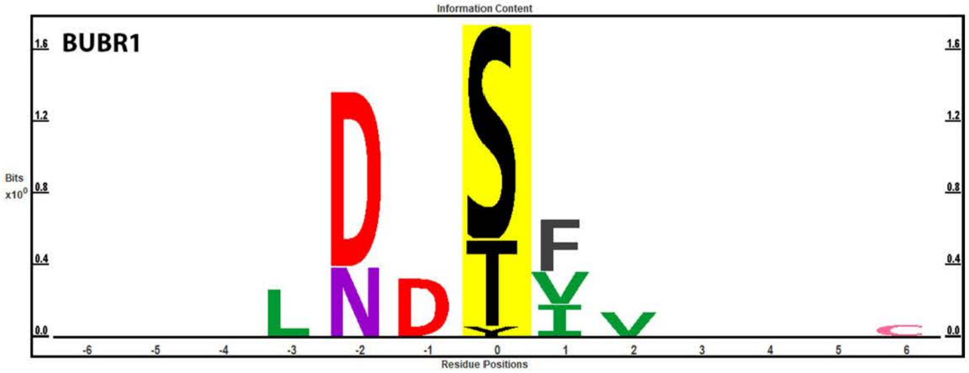
Information Content
BUBR1
Bits
x10^0
1.6
1.2
0.8
0.4
0.0
-6 -5 -4 -3 -2 -1 0 1 2 3 4 5 6
Residue Positions

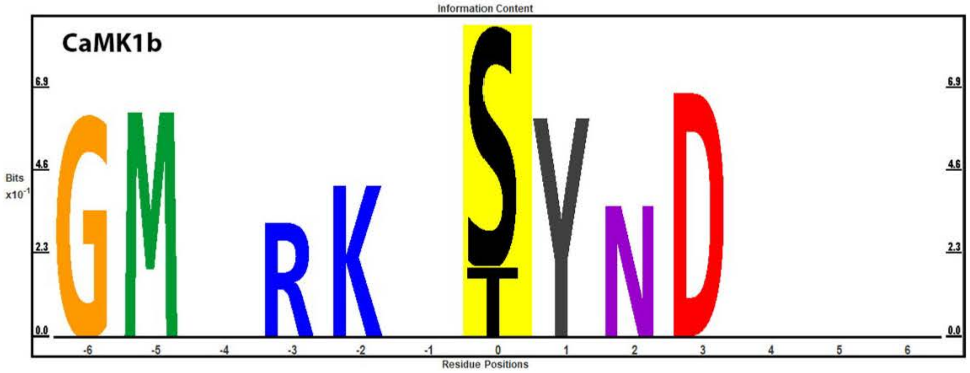
Information Content
CaMK1b
Bits
x10-1
6.9
4.6
2.3
0.0
-6 -5 -4 -3 -2 -1 0 1 2 3 4 5 6
Residue Positions

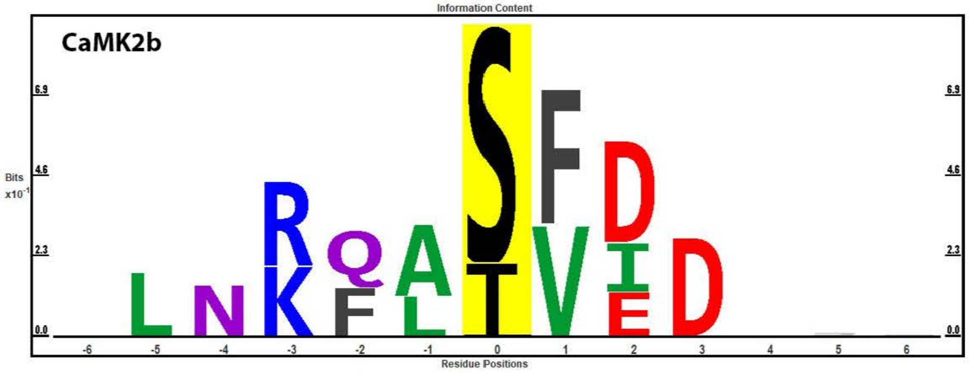
Information Content
CaMK2b
Bits
x10-1
Residue Positions

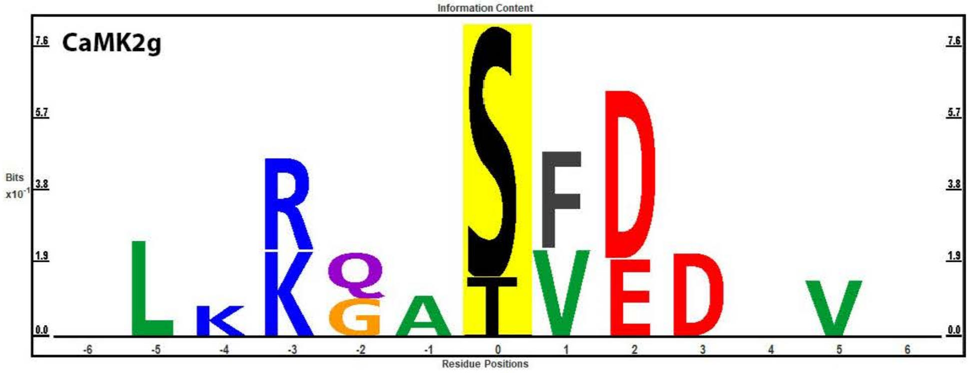
Information Content
CaMK2g
Bits
x10-1
Residue Positions

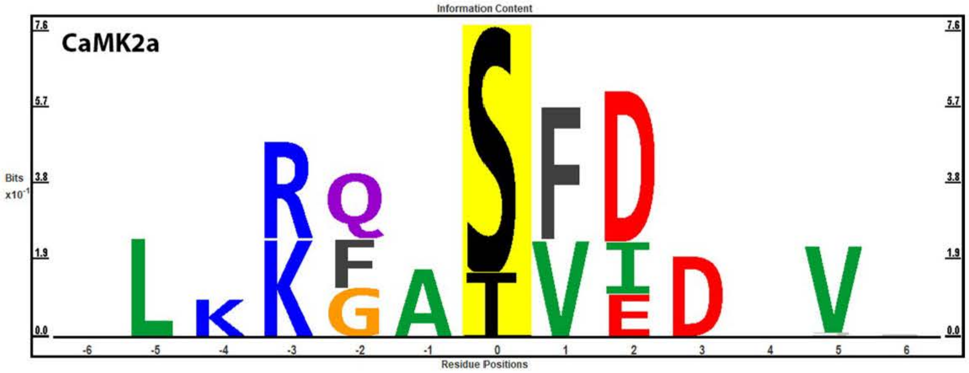
Information Content
CaMK2a
Bits
x10-1
Residue Positions

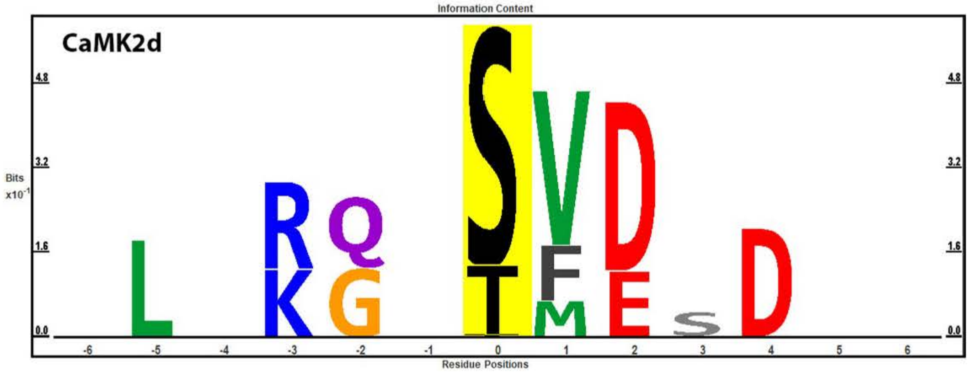
Information Content
CaMK2d
Bits
x10-1
Residue Positions

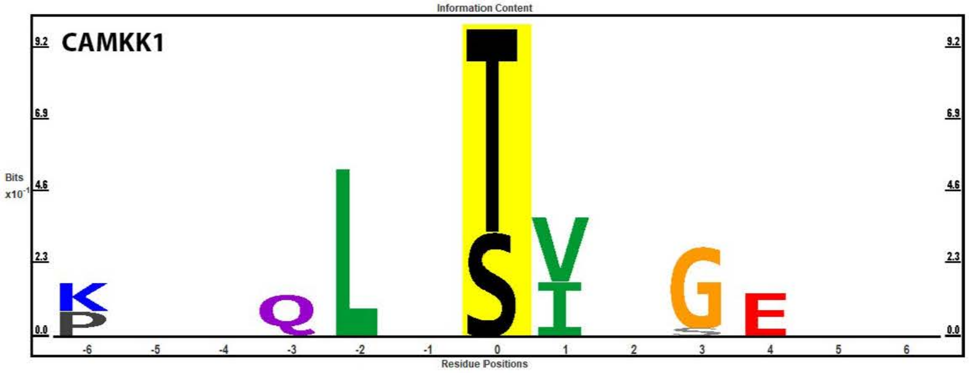
Information Content
CAMKK1
Bits
Residue Positions

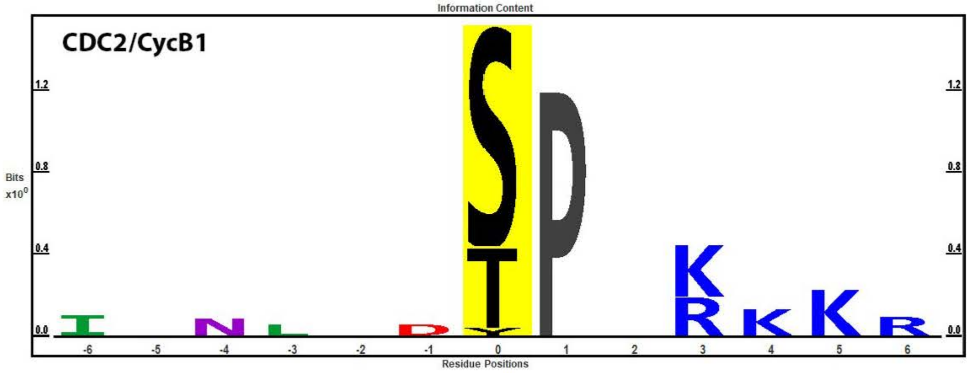
Information Content
CDC2/CycB1
Bits
Residue Positions

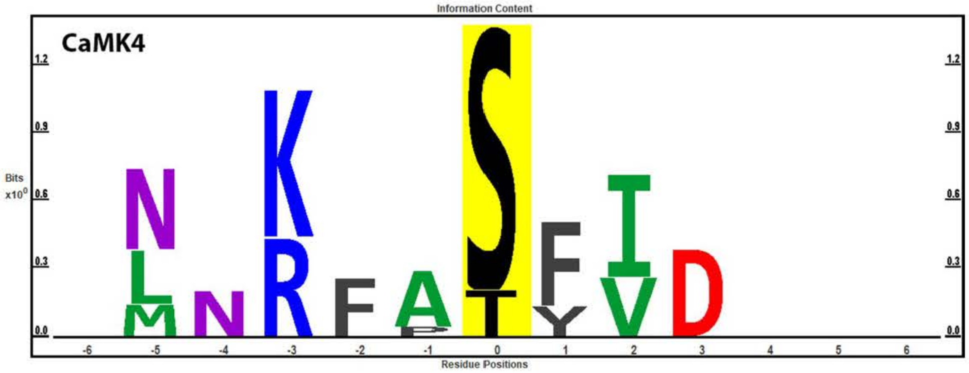
Information Content
CaMK4
Bits
Residue Positions

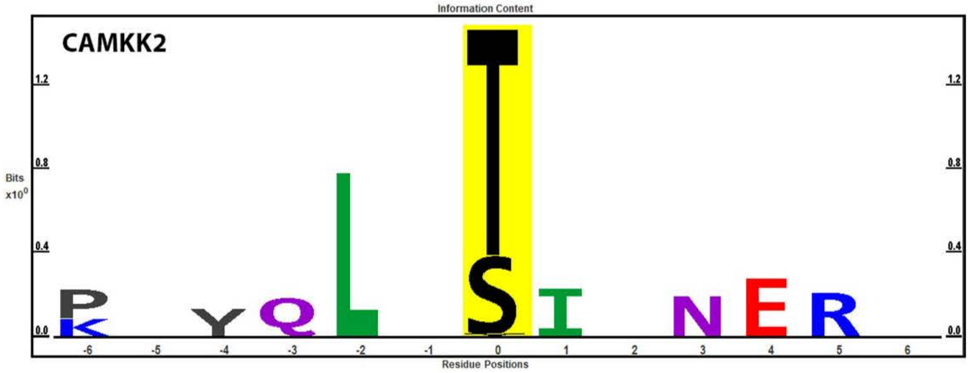
Information Content
CAMKK2
Bits
Residue Positions

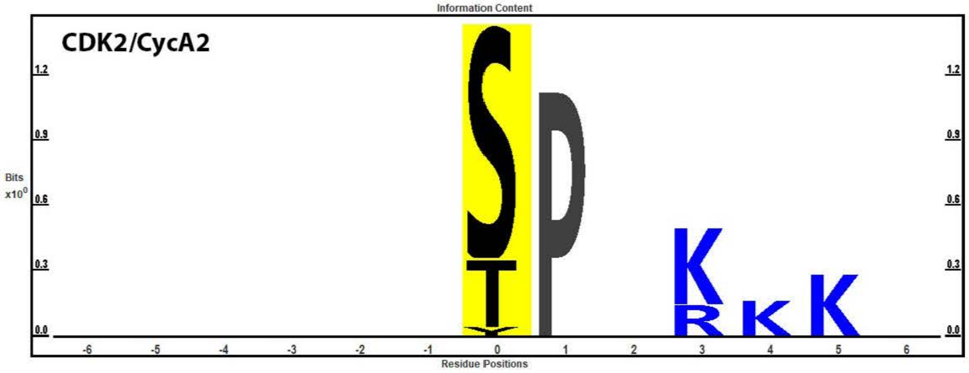
Information Content
CDK2/CycA2
Bits
x10^0
Residue Positions

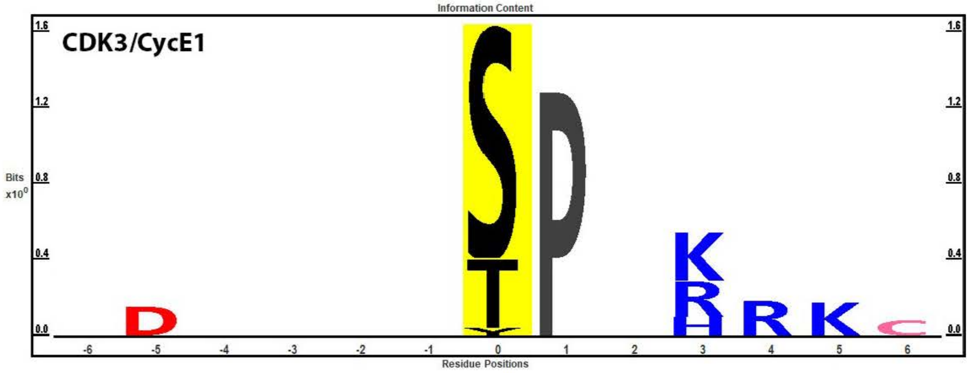
Information Content
CDK3/CycE1
Bits
x10^0
Residue Positions

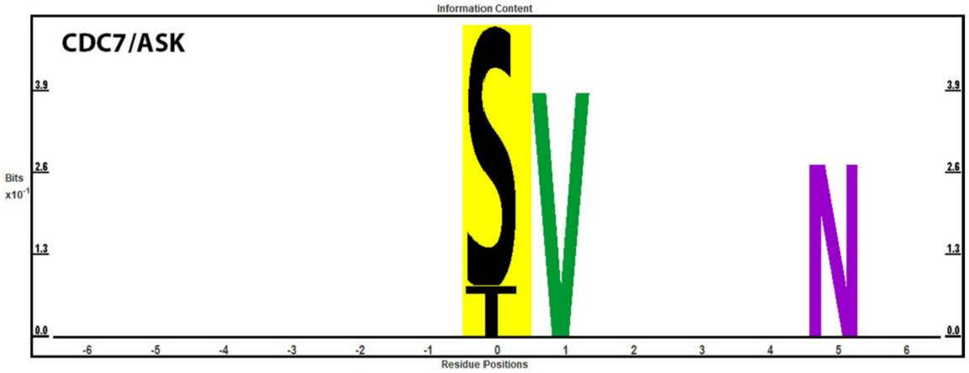
Information Content
CDC7/ASK
Bits
x10^-1
Residue Positions

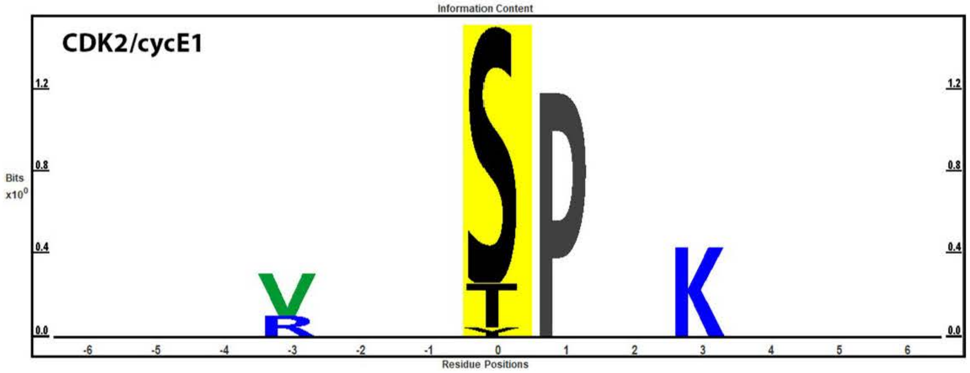
Information Content
CDK2/cycE1
Bits
x10^0
Residue Positions

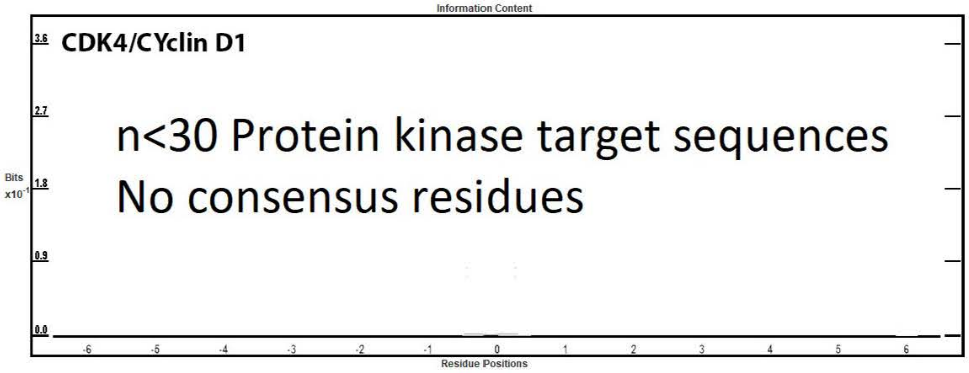
Information Content
CDK4/CYclin D1
n<30 Protein kinase target sequences
No consensus residues
Bits
Residue Positions

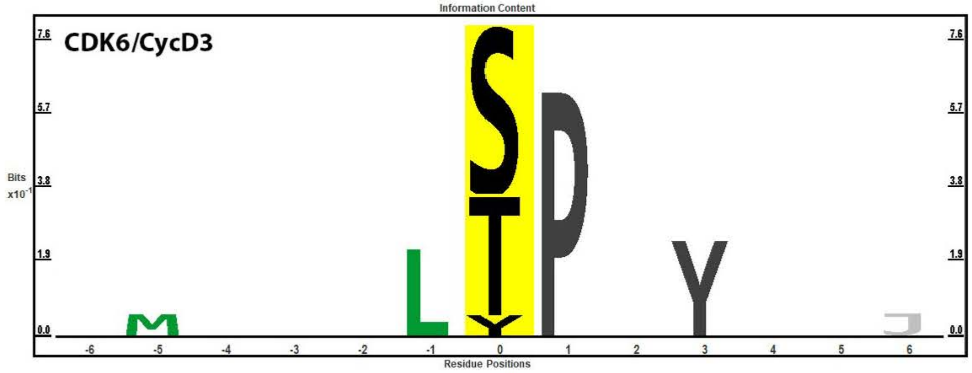
Information Content
CDK6/CycD3
Bits
Residue Positions

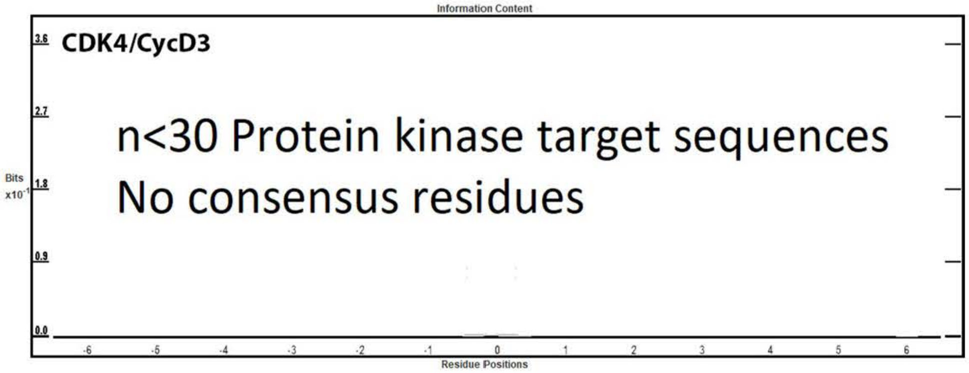
Information Content
CDK4/CycD3
n<30 Protein kinase target sequences
No consensus residues
Bits
Residue Positions

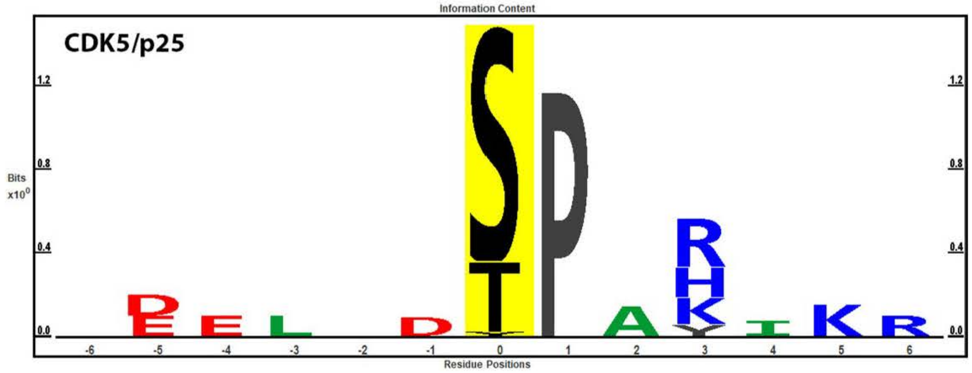
Information Content
CDK5/p25
Bits
Residue Positions

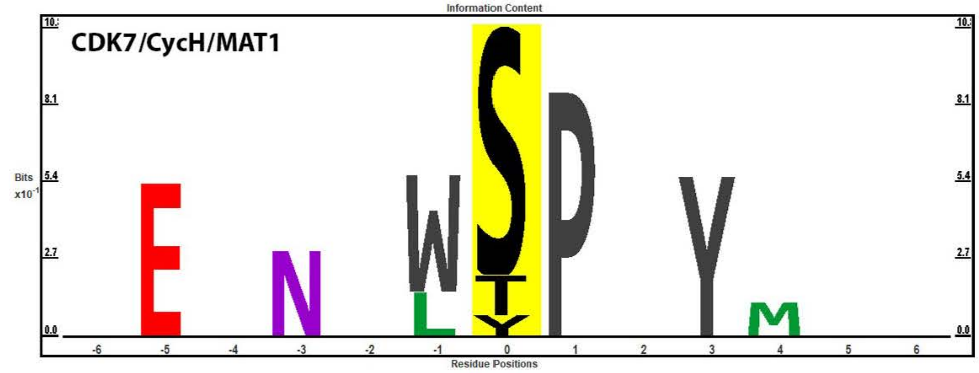
Information Content
CDK7/CycH/MAT1
Bits
x10-1
Residue Positions

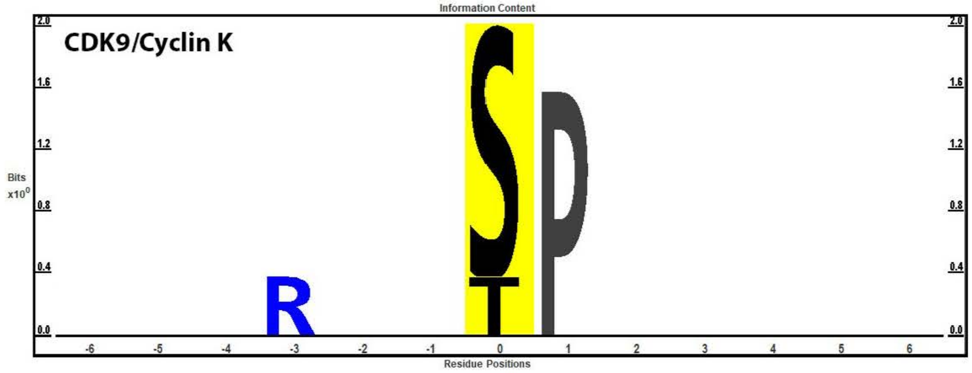
Information Content
CDK9/Cyclin K
Bits
x100
Residue Positions

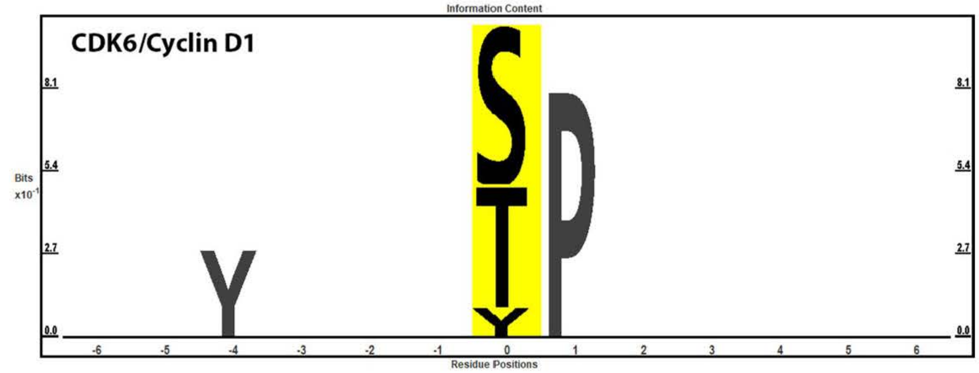
Information Content
CDK6/Cyclin D1
Bits
x10-1
Residue Positions

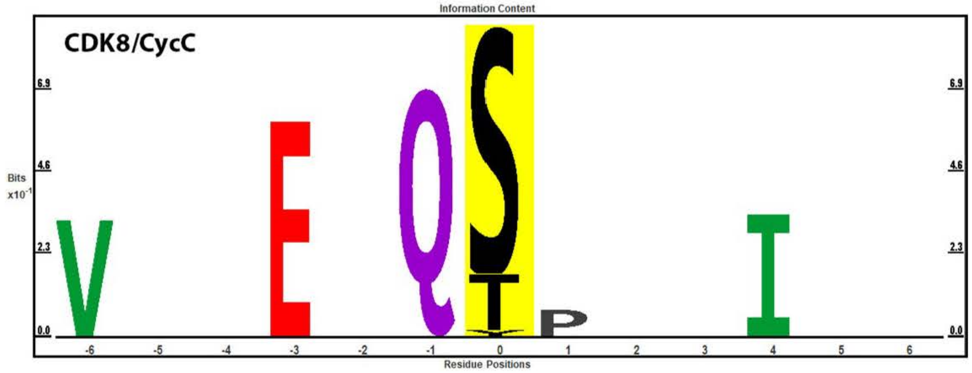
Information Content
CDK8/CycC
Bits
x10-1
Residue Positions

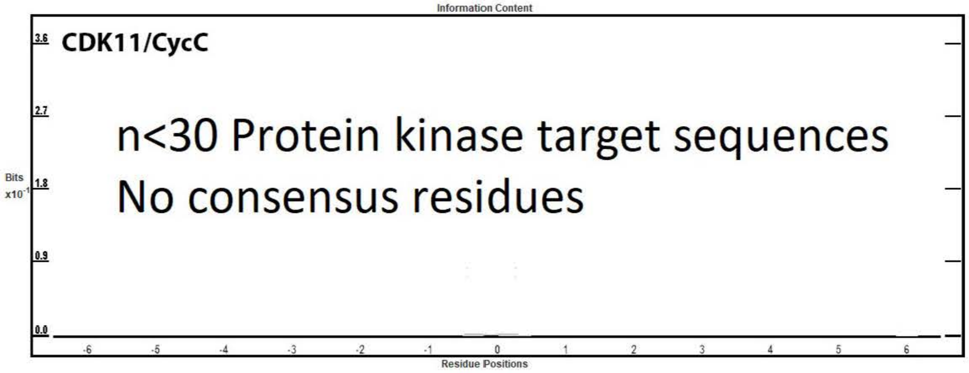
Information Content
CDK11/CycC
n<30 Protein kinase target sequences
No consensus residues
Bits x10-1
Residue Positions

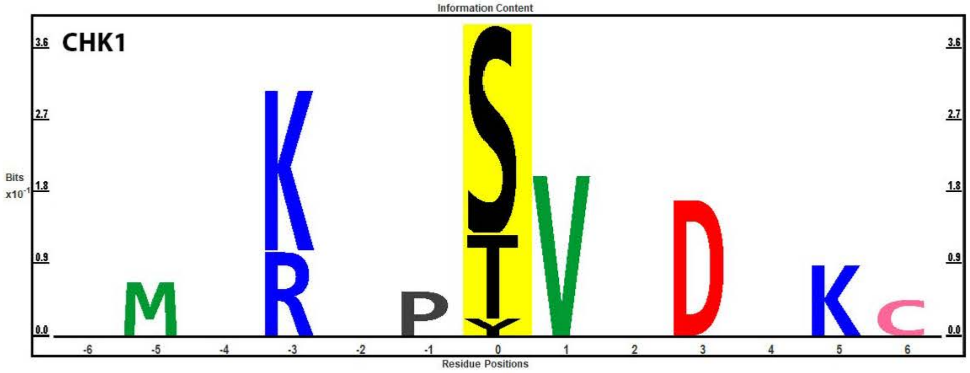
Information Content
CHK1
Bits x10-1
Residue Positions

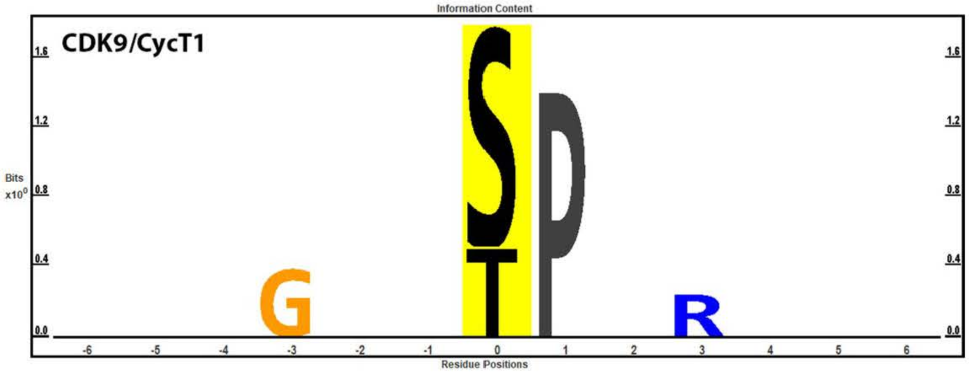
Information Content
CDK9/CycT1
Bits x10[0]
Residue Positions

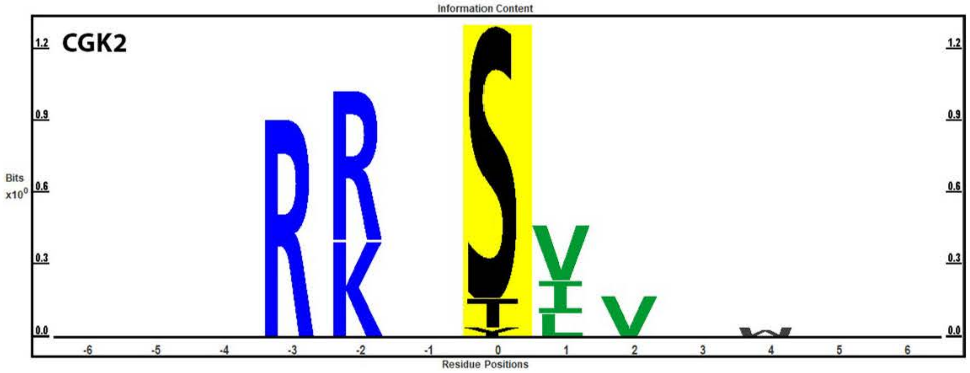
Information Content
CGK2
Bits x10[0]
Residue Positions

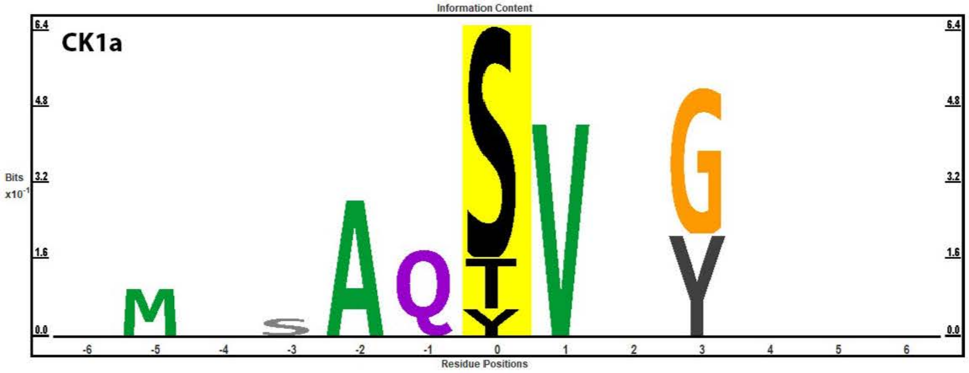
Information Content
CK1a
Bits
x10-1
Residue Positions

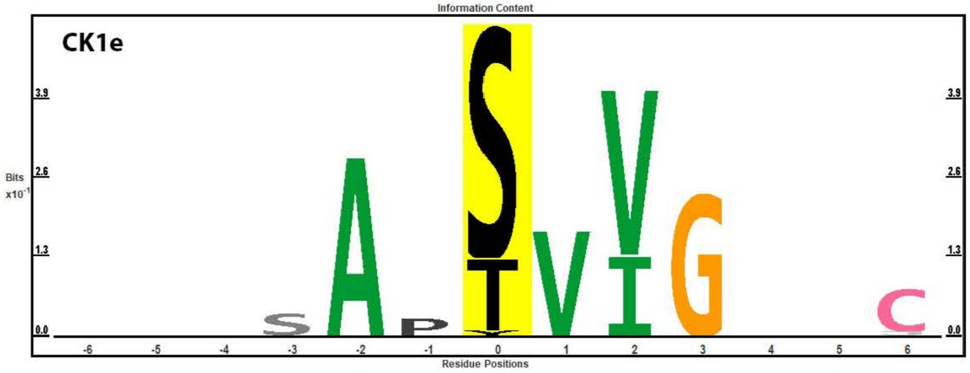
Information Content
CK1e
Bits
x10-1
Residue Positions

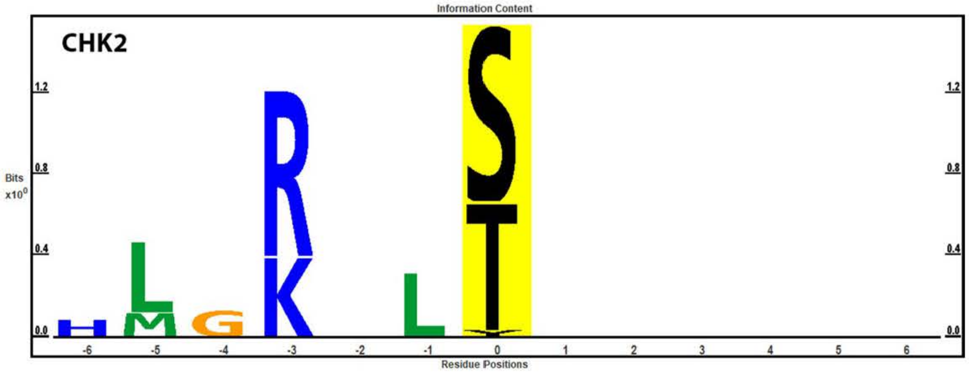
Information Content
CHK2
Bits
x100
Residue Positions

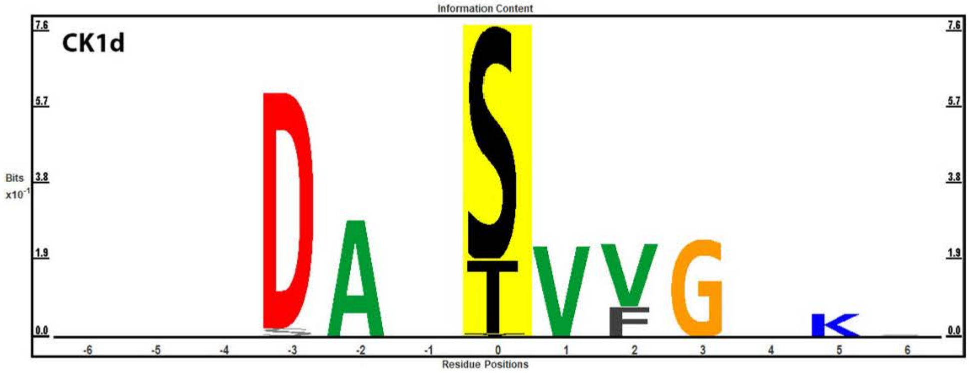
Information Content
CK1d
Bits
x10-1
Residue Positions

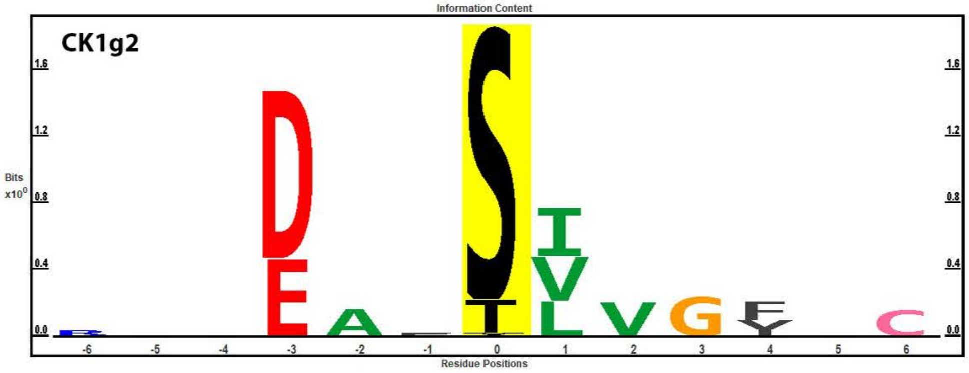
Information Content
CK1g2
Bits
x10^0
Residue Positions

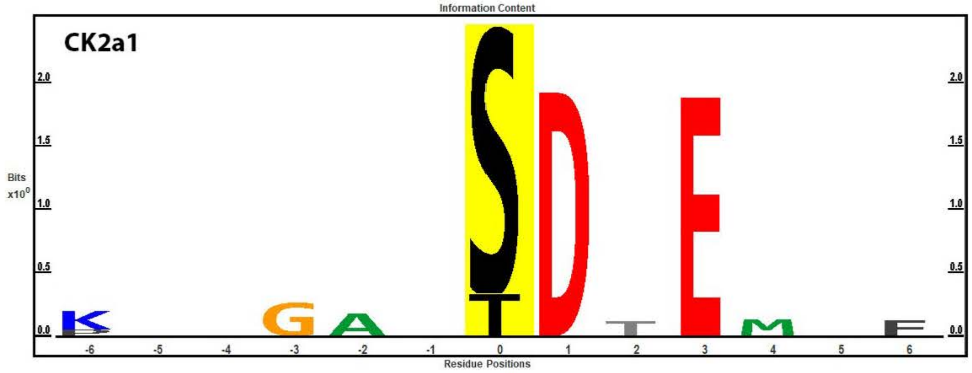
Information Content
CK2a1
Bits
x10^0
Residue Positions

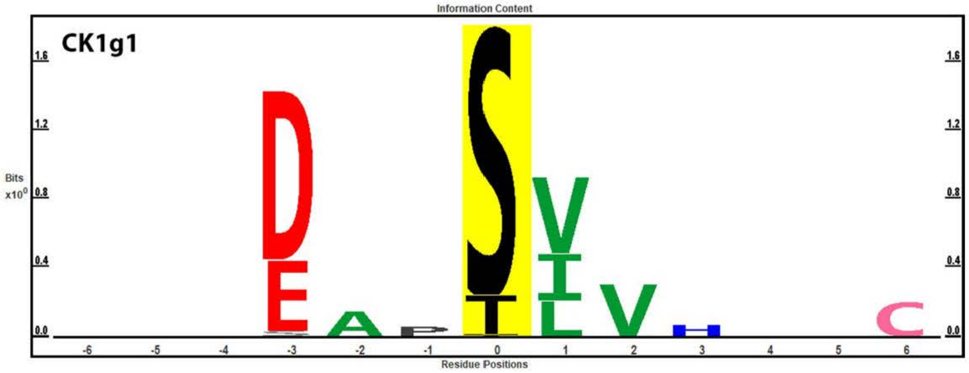
Information Content
CK1g1
Bits
x10^0
Residue Positions

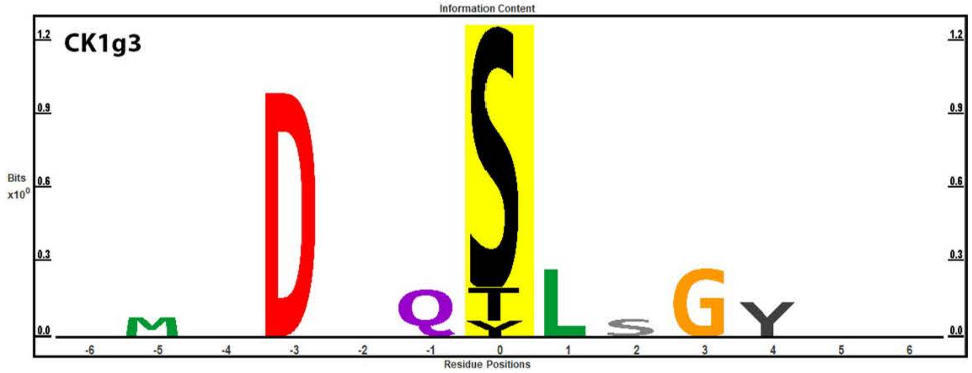
Information Content
CK1g3
Bits
x10^0
Residue Positions

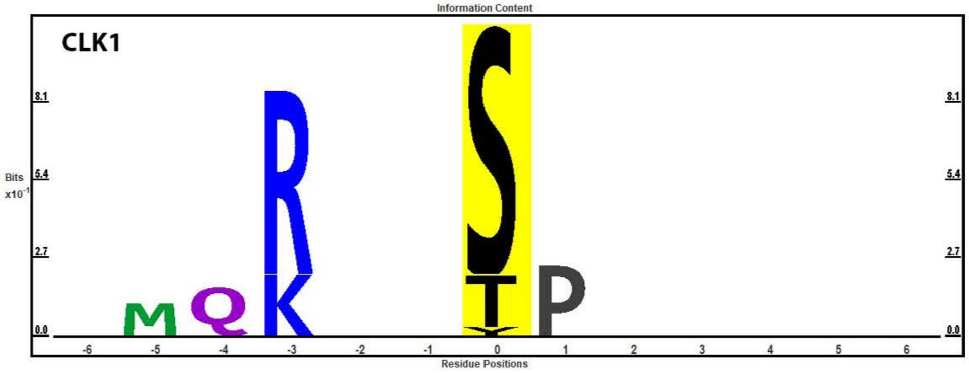
Information Content
CLK1
Bits
x10-1
8.1
5.4
2.7
0.0
-6 -5 -4 -3 -2 -1 0 1 2 3 4 5 6
Residue Positions

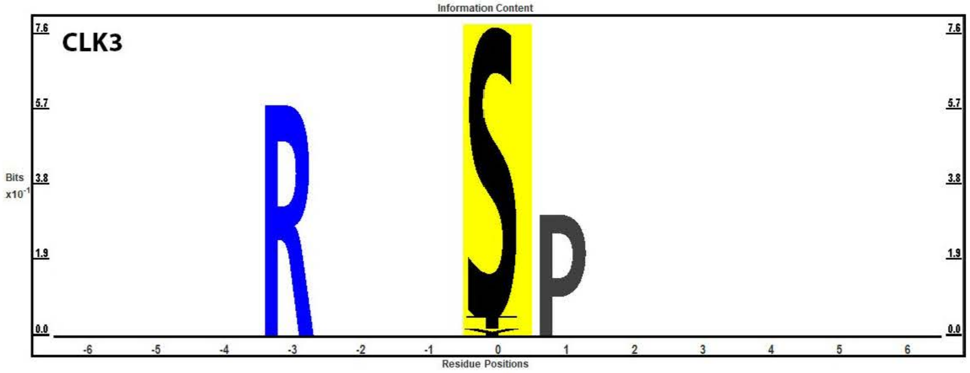
Information Content
CLK3
Bits
x10-1
7.6
5.7
3.8
1.9
0.0
-6 -5 -4 -3 -2 -1 0 1 2 3 4 5 6
Residue Positions

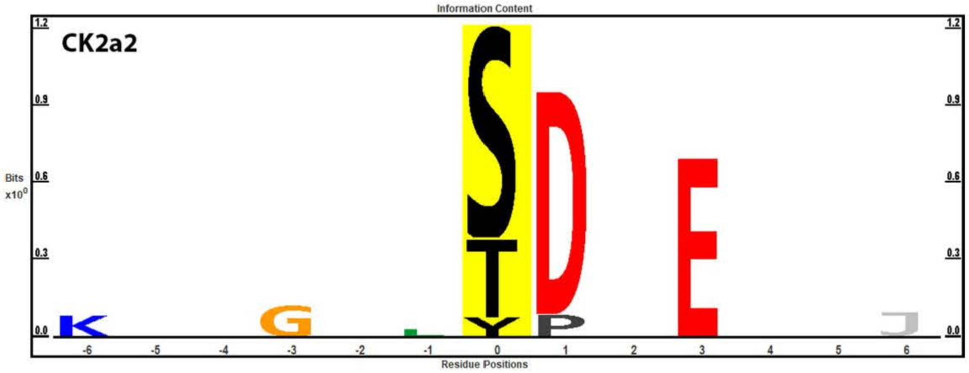
Information Content
CK2a2
Bits
x100
1.2
0.9
0.6
0.3
0.0
-6 -5 -4 -3 -2 -1 0 1 2 3 4 5 6
Residue Positions

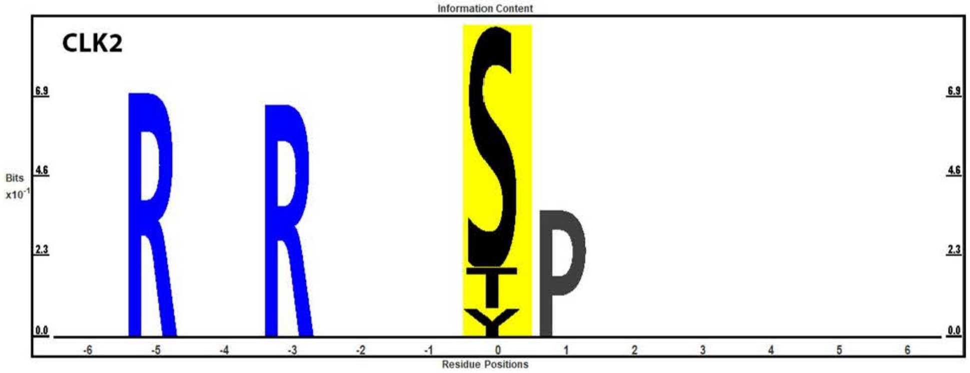
Information Content
CLK2
Bits
x10-1
6.9
4.6
2.3
0.0
-6 -5 -4 -3 -2 -1 0 1 2 3 4 5 6
Residue Positions

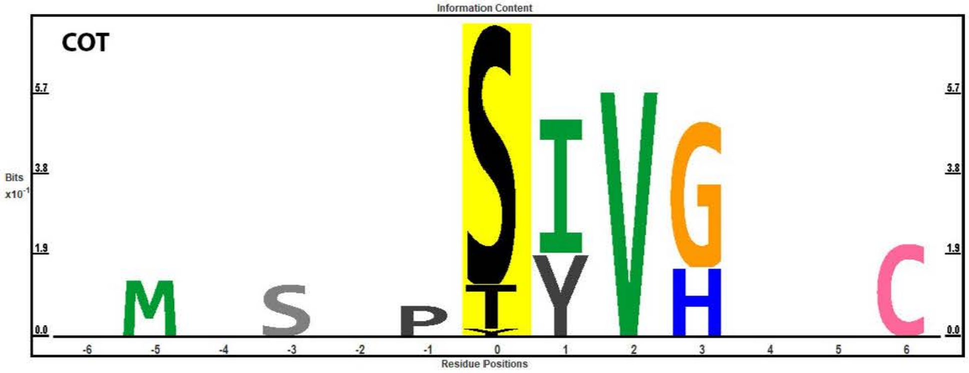
Information Content
COT
Bits x10⁻¹
5.7
3.8
1.9
0.0
-6 -5 -4 -3 -2 -1 0 1 2 3 4 5 6
Residue Positions

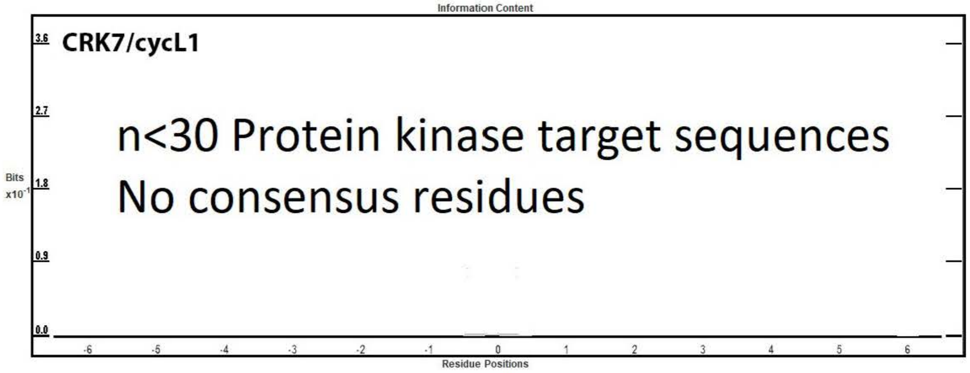
Information Content
CRK7/cycL1
n<30 Protein kinase target sequences
No consensus residues
Bits x10⁻¹
3.6
2.7
1.8
0.9
0.0
-6 -5 -4 -3 -2 -1 0 1 2 3 4 5 6
Residue Positions

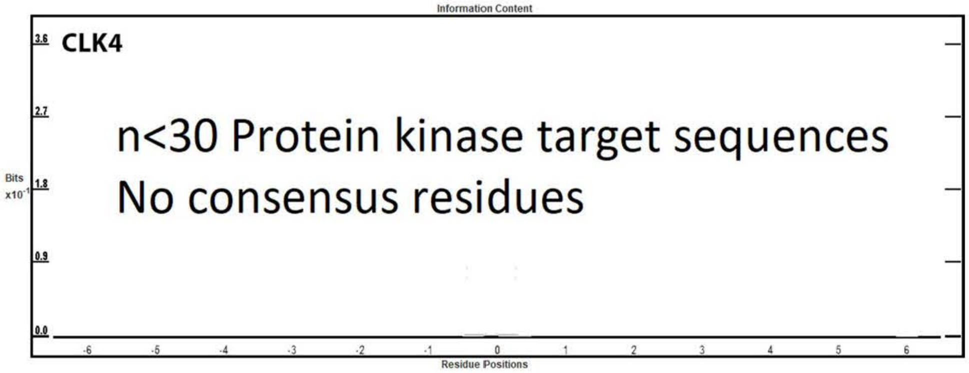
Information Content
CLK4
n<30 Protein kinase target sequences
No consensus residues
Bits x10⁻¹
3.6
2.7
1.8
0.9
0.0
-6 -5 -4 -3 -2 -1 0 1 2 3 4 5 6
Residue Positions

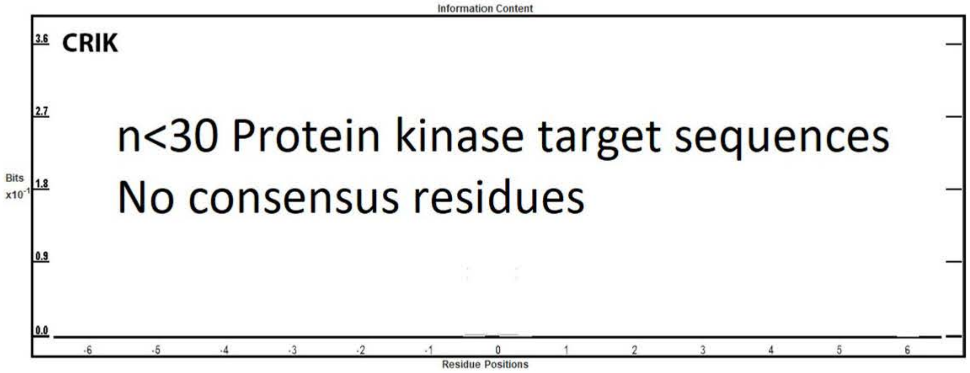
Information Content
CRIK
n<30 Protein kinase target sequences
No consensus residues
Bits x10⁻¹
3.6
2.7
1.8
0.9
0.0
-6 -5 -4 -3 -2 -1 0 1 2 3 4 5 6
Residue Positions

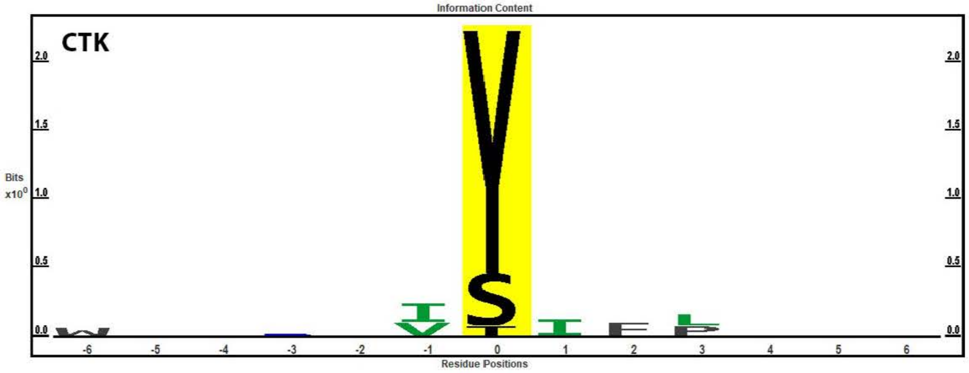
Information Content
CTK
Bits
x10^0
2.0
1.5
1.0
0.5
0.0
-6 -5 -4 -3 -2 -1 0 1 2 3 4 5 6
Residue Positions

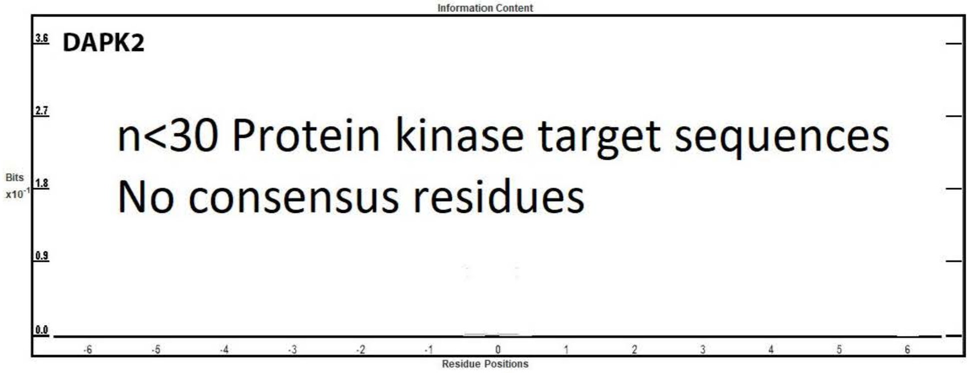
Information Content
DAPK2
n<30 Protein kinase target sequences
No consensus residues
Bits
x10^-1
3.6
2.7
1.8
0.9
0.0
-6 -5 -4 -3 -2 -1 0 1 2 3 4 5 6
Residue Positions

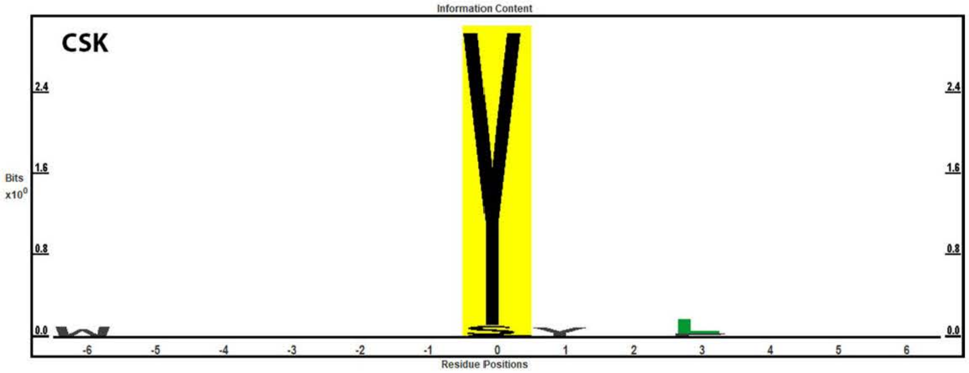
Information Content
CSK
Bits
x10^0
2.4
1.6
0.8
0.0
-6 -5 -4 -3 -2 -1 0 1 2 3 4 5 6
Residue Positions

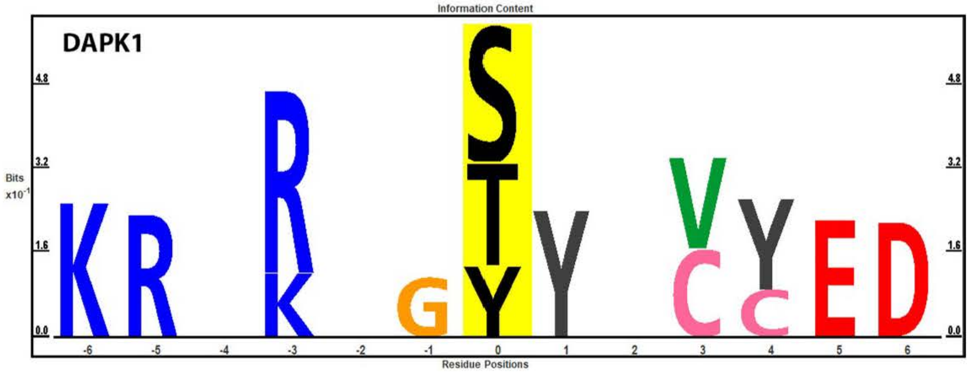
Information Content
DAPK1
Bits
x10^-1
4.8
3.2
1.6
0.0
K R R K G S T Y Y V C Y C E D
-6 -5 -4 -3 -2 -1 0 1 2 3 4 5 6
Residue Positions

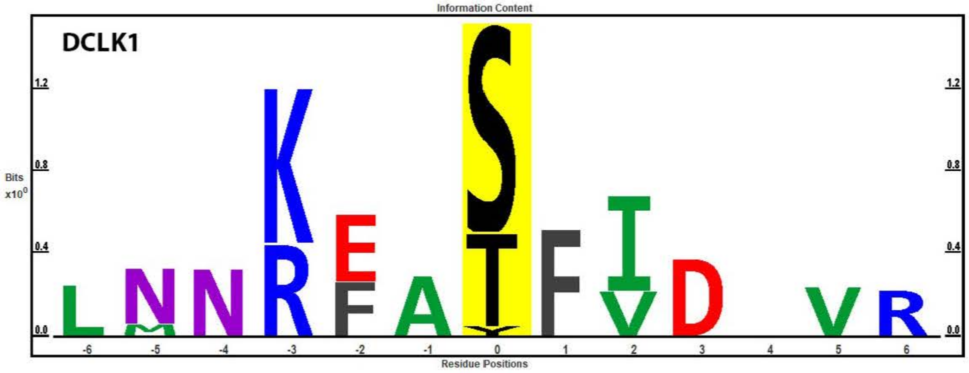
Information Content
DCLK1
Bits
x10^0
Residue Positions

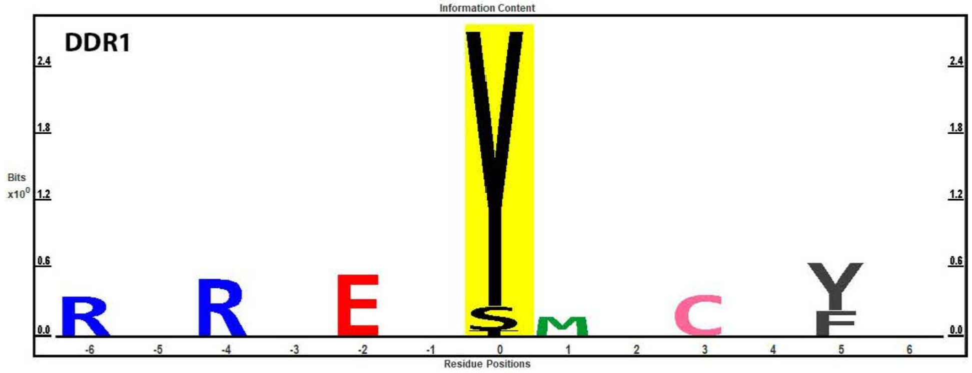
Information Content
DDR1
Bits
x10^0
Residue Positions

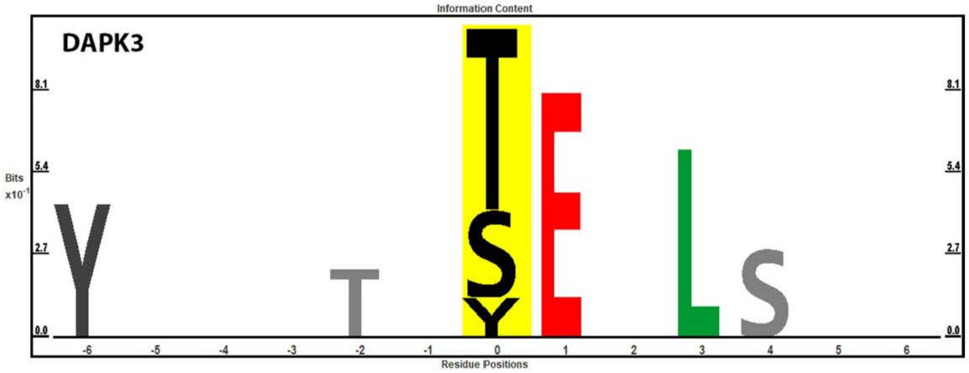
Information Content
DAPK3
Bits
x10^-1
Residue Positions

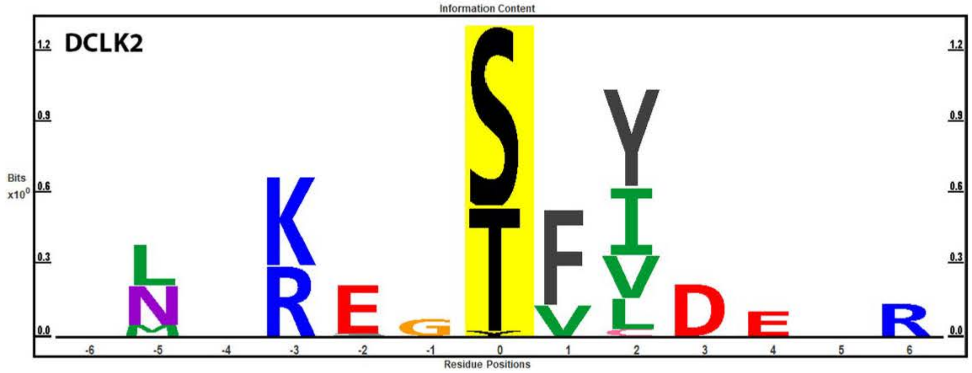
Information Content
DCLK2
Bits
x10^0
Residue Positions

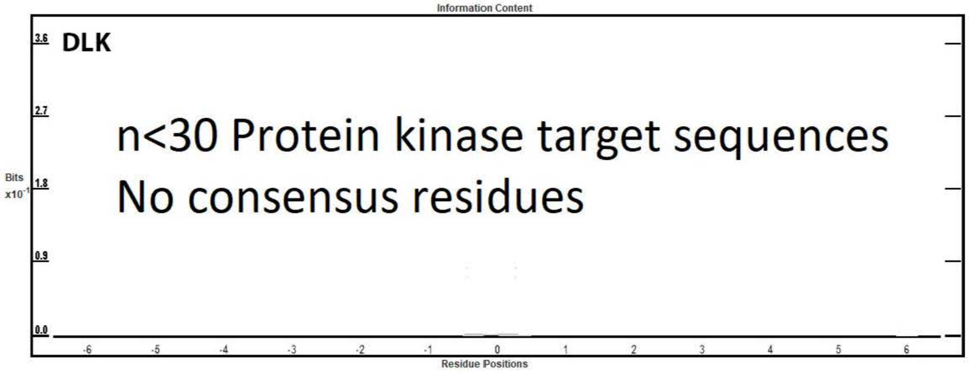
Information Content
DLK
n<30 Protein kinase target sequences
No consensus residues
Bits
Residue Positions

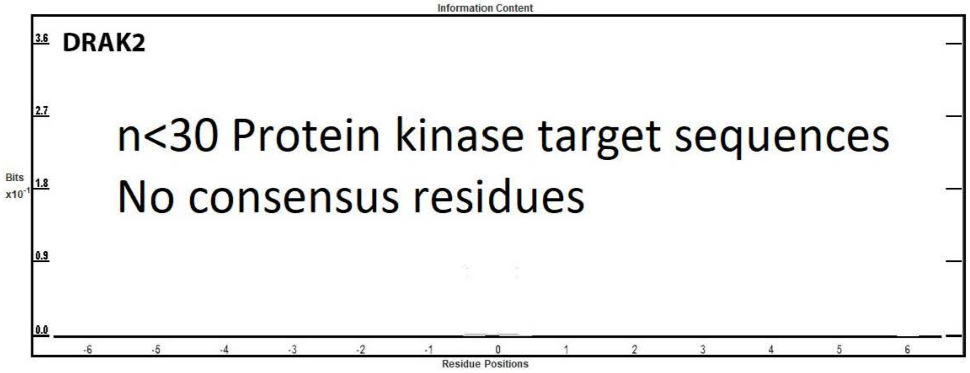
Information Content
DRAK2
n<30 Protein kinase target sequences
No consensus residues
Bits
Residue Positions

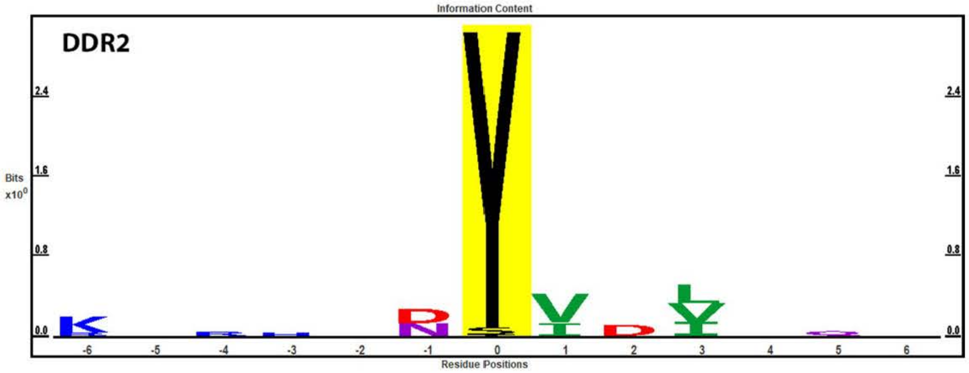
Information Content
DDR2
Bits
Residue Positions

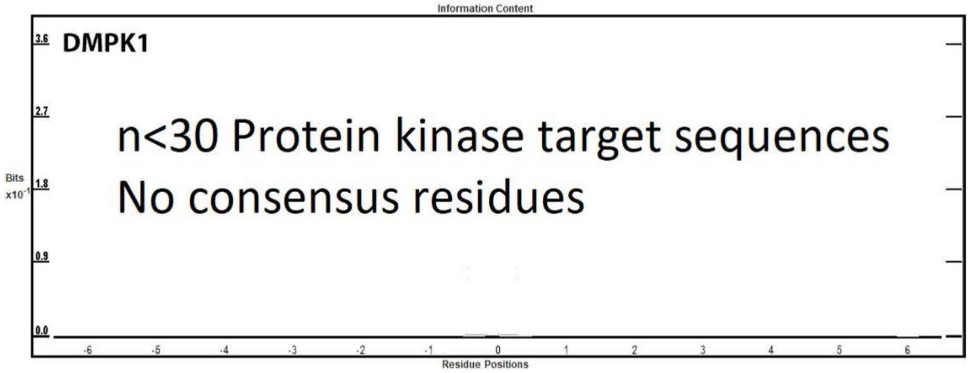
Information Content
DMPK1
n<30 Protein kinase target sequences
No consensus residues
Bits
Residue Positions

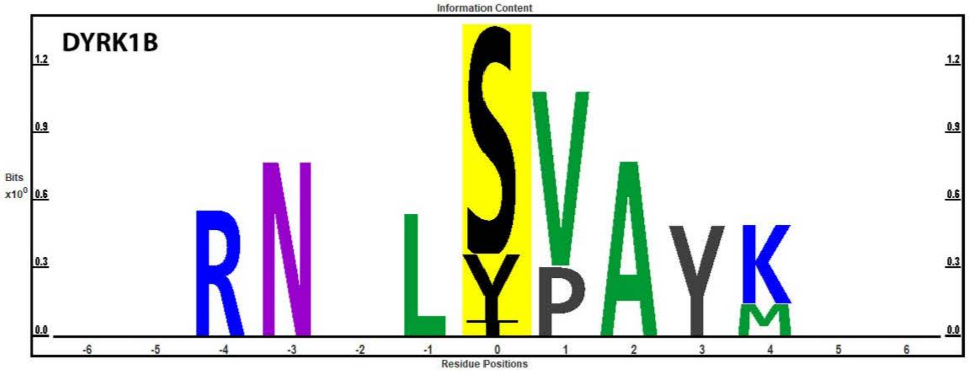
Information Content
DYRK1B
Bits
x10^0
Residue Positions

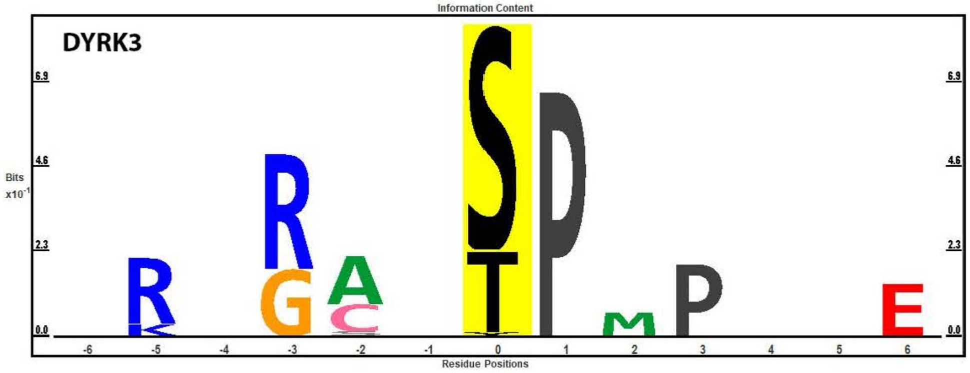
Information Content
DYRK3
Bits
x10^-1
Residue Positions

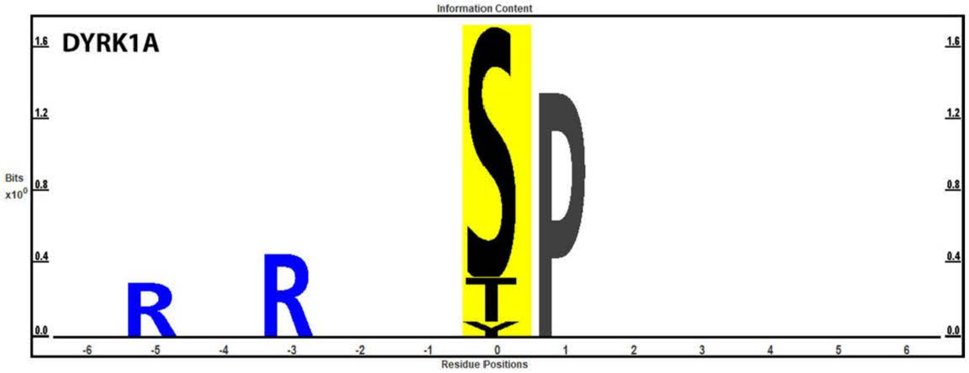
Information Content
DYRK1A
Bits
x10^0
Residue Positions

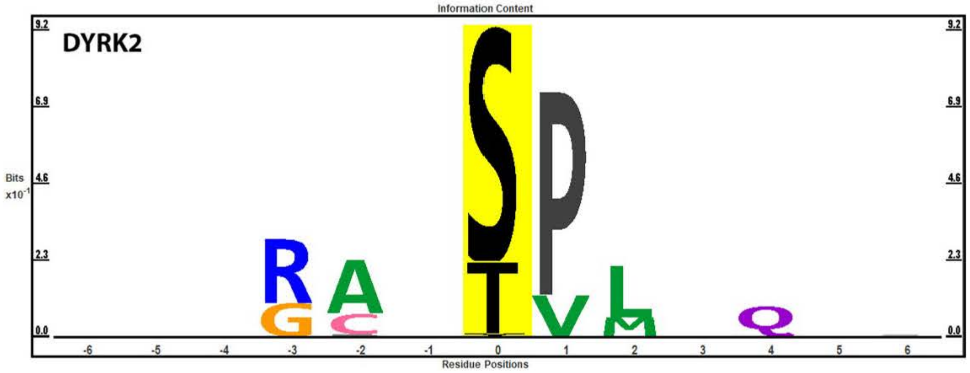
Information Content
DYRK2
Bits
x10^-1
Residue Positions

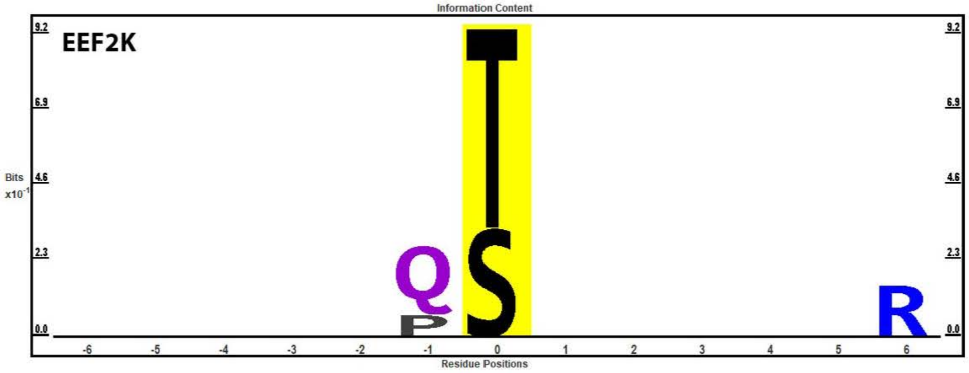
Information Content
EEF2K
Bits
x10-1
9.2
6.9
4.6
2.3
0.0
-6 -5 -4 -3 -2 -1 0 1 2 3 4 5 6
Residue Positions

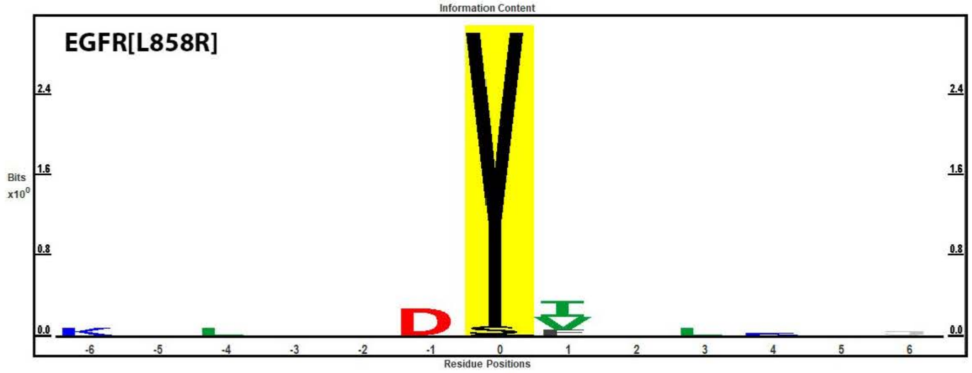
Information Content
EGFR[L858R]
Bits
x10^0
2.4
1.6
0.8
0.0
-6 -5 -4 -3 -2 -1 0 1 2 3 4 5 6
Residue Positions

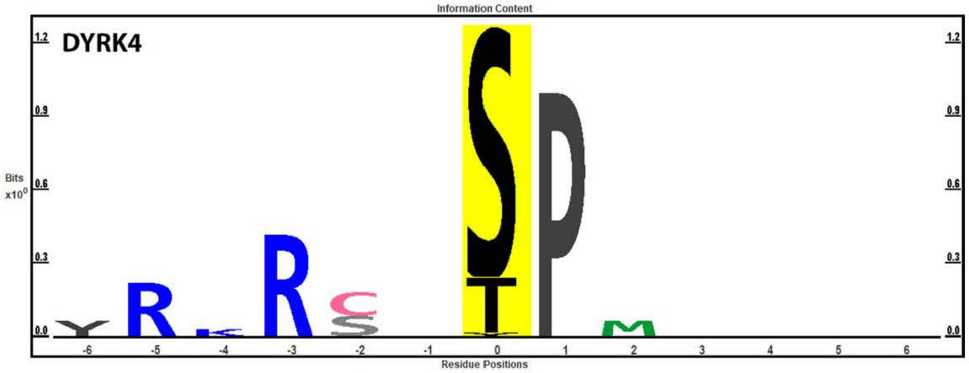
Information Content
DYRK4
Bits
x10^0
1.2
0.9
0.6
0.3
0.0
-6 -5 -4 -3 -2 -1 0 1 2 3 4 5 6
Residue Positions

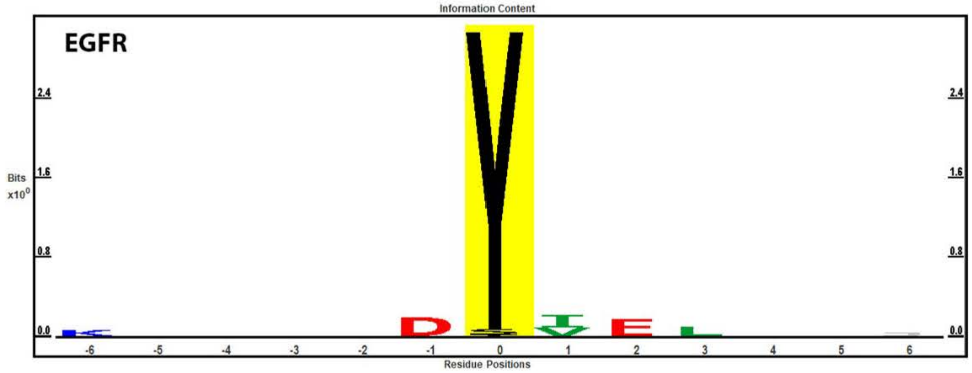
Information Content
EGFR
Bits
x10^0
2.4
1.6
0.8
0.0
-6 -5 -4 -3 -2 -1 0 1 2 3 4 5 6
Residue Positions

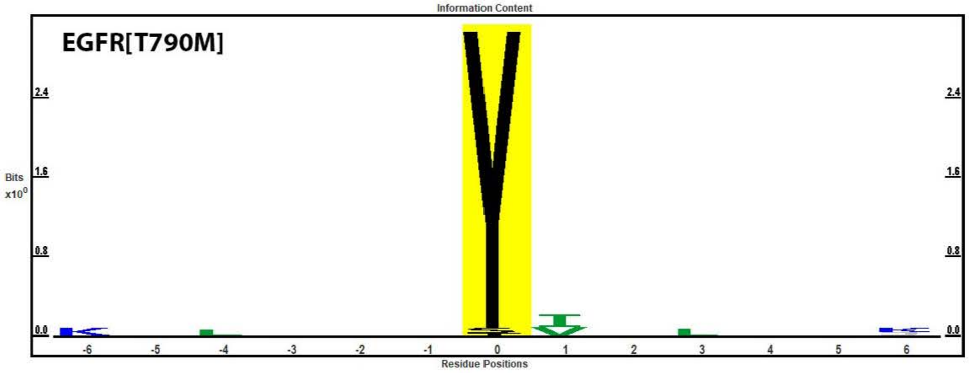
Information Content
EGFR[T790M]
Bits
x10^0
2.4
1.6
0.8
0.0
-6 -5 -4 -3 -2 -1 0 1 2 3 4 5 6
Residue Positions

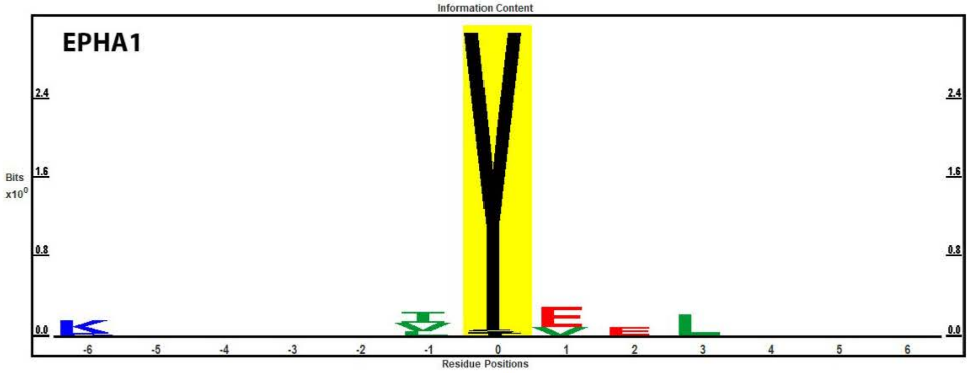
Information Content
EPHA1
Bits
x10^0
2.4
1.6
0.8
0.0
-6 -5 -4 -3 -2 -1 0 1 2 3 4 5 6
Residue Positions

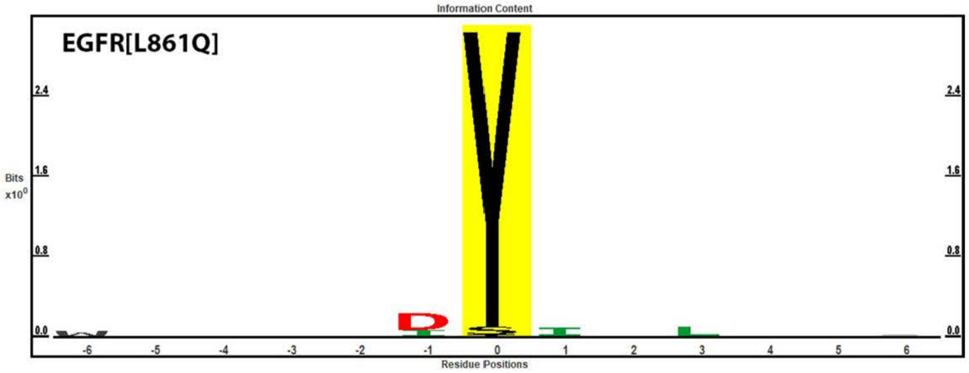
Information Content
EGFR[L861Q]
Bits
x10^0
2.4
1.6
0.8
0.0
-6 -5 -4 -3 -2 -1 0 1 2 3 4 5 6
Residue Positions

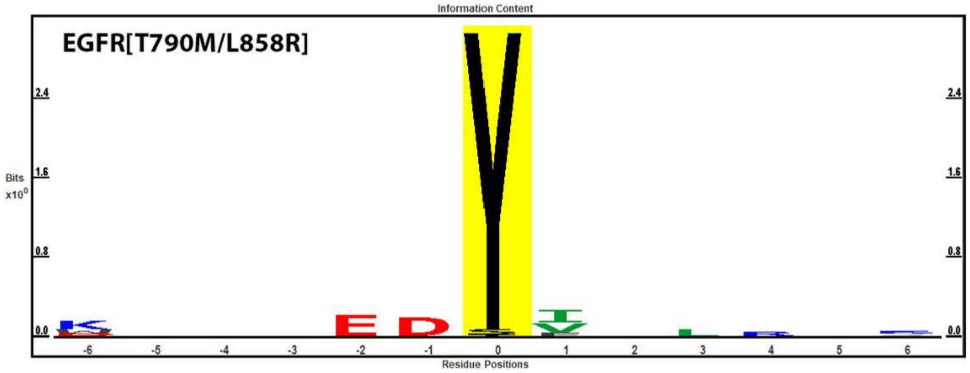
Information Content
EGFR[T790M/L858R]
Bits
x10^0
2.4
1.6
0.8
0.0
-6 -5 -4 -3 -2 -1 0 1 2 3 4 5 6
Residue Positions

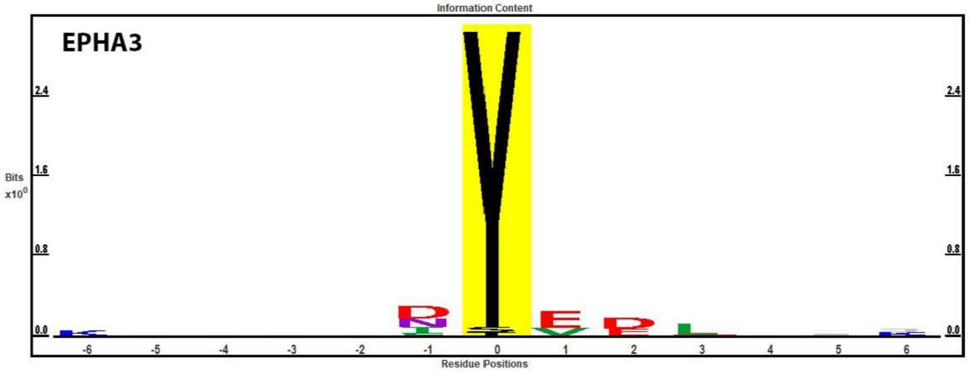
Information Content
EPHA3
Bits
x10^0
2.4
1.6
0.8
0.0
Residue Positions
-6 -5 -4 -3 -2 -1 0 1 2 3 4 5 6

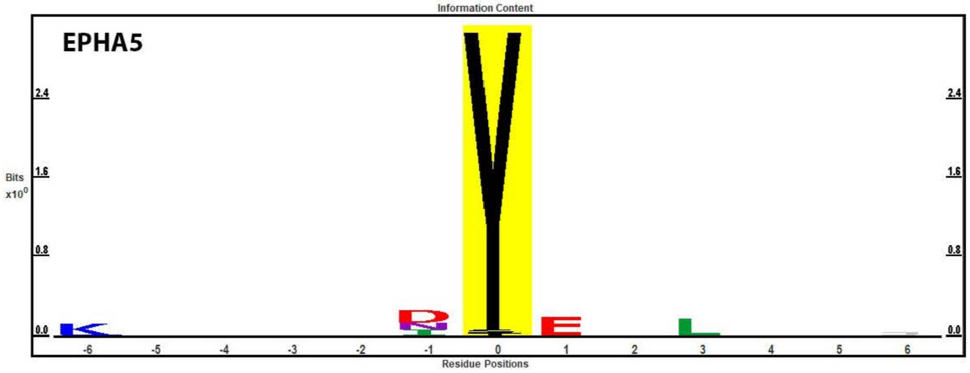
Information Content
EPHA5
Bits
x10^0
2.4
1.6
0.8
0.0
Residue Positions
-6 -5 -4 -3 -2 -1 0 1 2 3 4 5 6

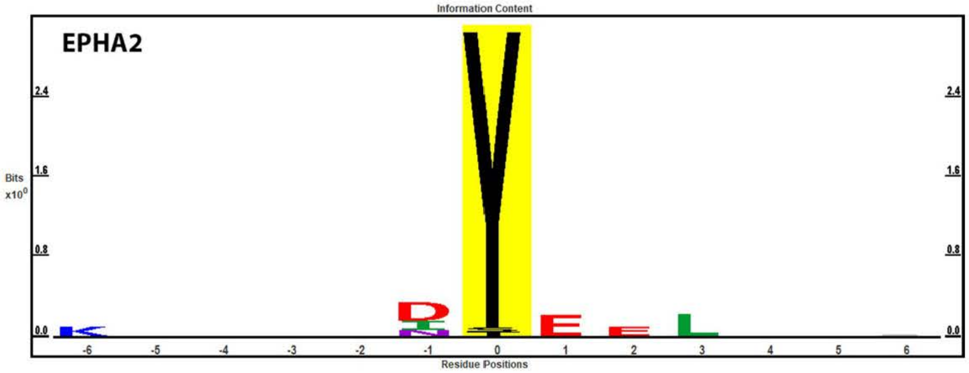
Information Content
EPHA2
Bits
x10^0
2.4
1.6
0.8
0.0
Residue Positions
-6 -5 -4 -3 -2 -1 0 1 2 3 4 5 6

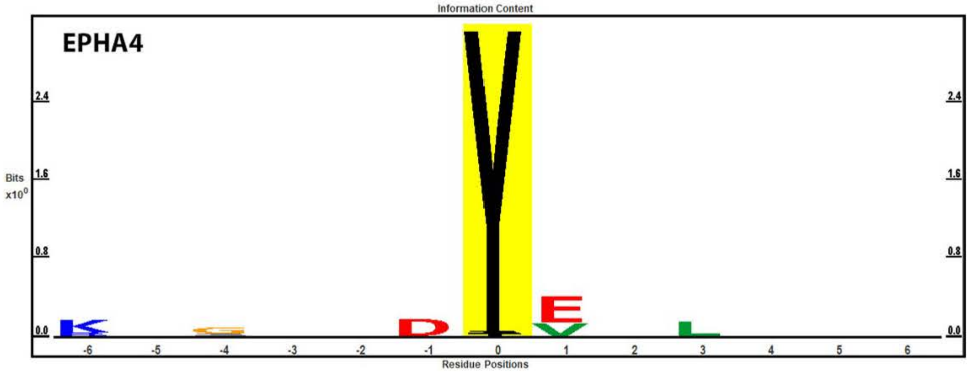
Information Content
EPHA4
Bits
x10^0
2.4
1.6
0.8
0.0
Residue Positions
-6 -5 -4 -3 -2 -1 0 1 2 3 4 5 6

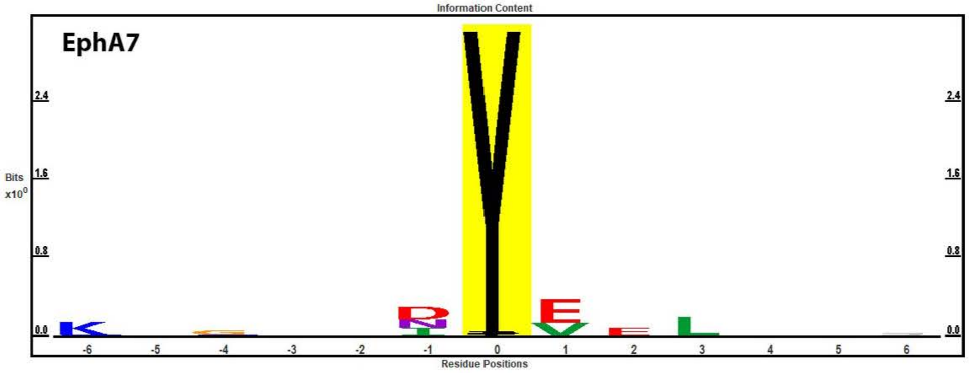
Information Content
EphA7
2.4
1.6
0.8
0.0
Bits
x10^0
-6 -5 -4 -3 -2 -1 0 1 2 3 4 5 6
Residue Positions

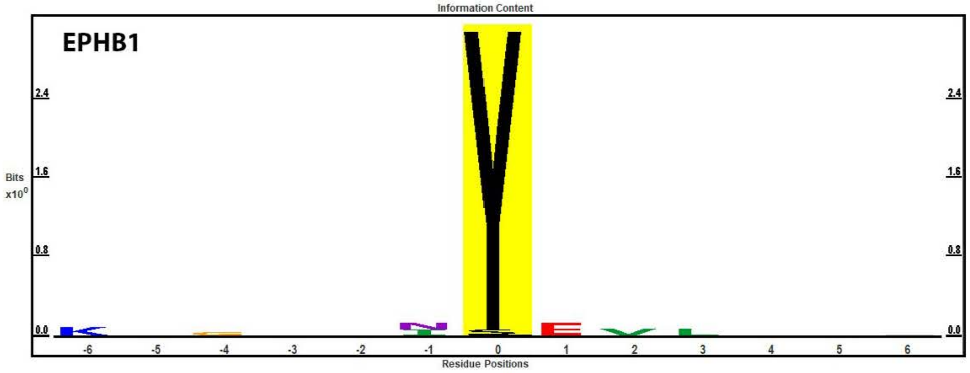
Information Content
EPHB1
2.4
1.6
0.8
0.0
Bits
x10^0
-6 -5 -4 -3 -2 -1 0 1 2 3 4 5 6
Residue Positions

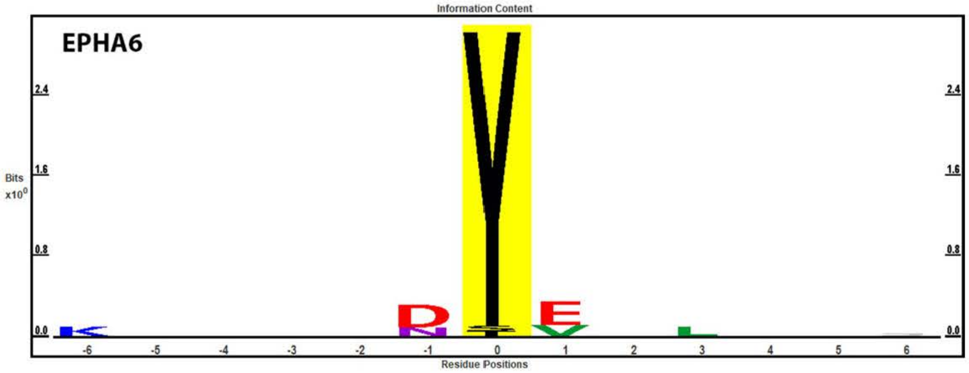
Information Content
EPHA6
2.4
1.6
0.8
0.0
Bits
x10^0
-6 -5 -4 -3 -2 -1 0 1 2 3 4 5 6
Residue Positions

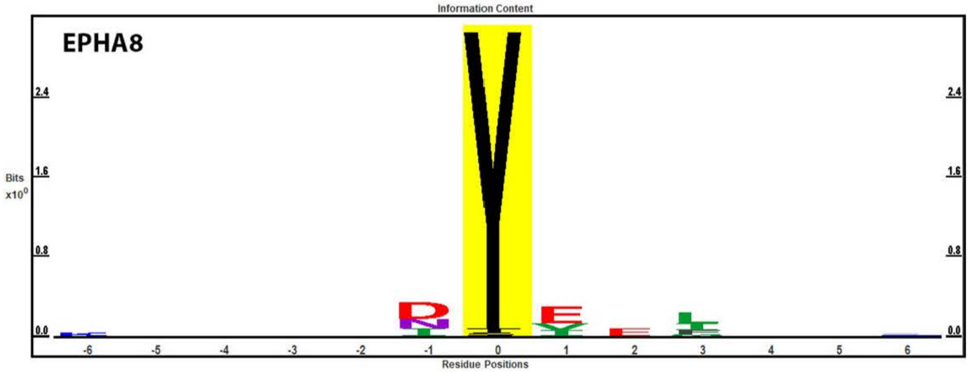
Information Content
EPHA8
2.4
1.6
0.8
0.0
Bits
x10^0
-6 -5 -4 -3 -2 -1 0 1 2 3 4 5 6
Residue Positions

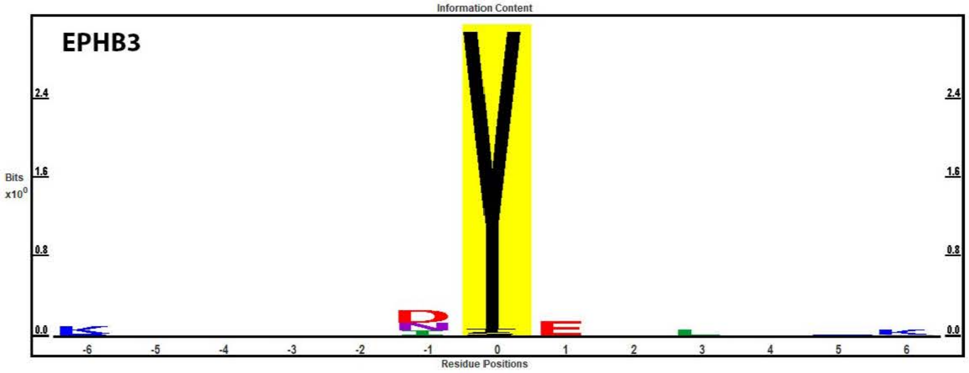
Information Content
EPHB3
Bits
x10^0
2.4
1.6
0.8
0.0
-6 -5 -4 -3 -2 -1 0 1 2 3 4 5 6
Residue Positions

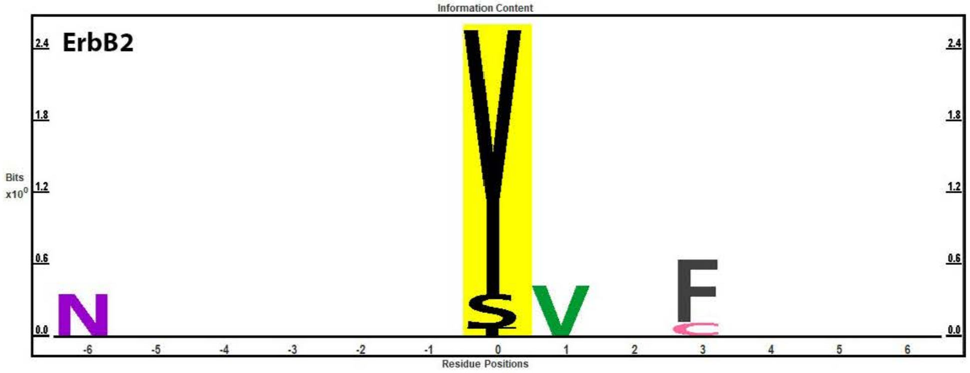
Information Content
ErbB2
Bits
x10^0
2.4
1.8
1.2
0.6
0.0
-6 -5 -4 -3 -2 -1 0 1 2 3 4 5 6
Residue Positions

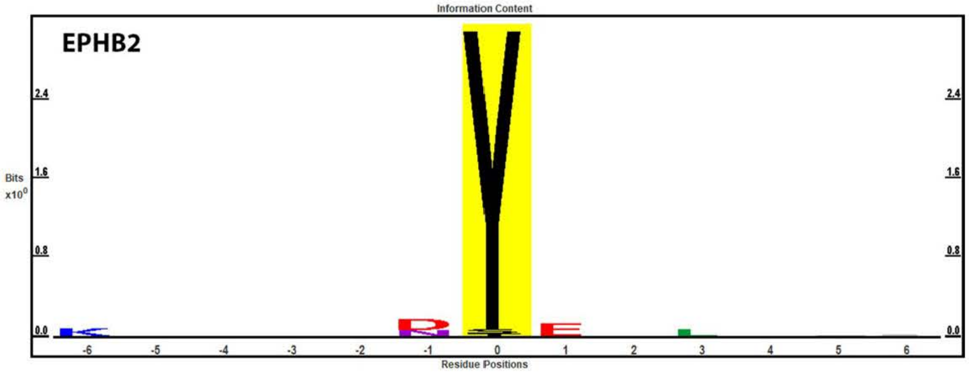
Information Content
EPHB2
Bits
x10^0
2.4
1.6
0.8
0.0
-6 -5 -4 -3 -2 -1 0 1 2 3 4 5 6
Residue Positions

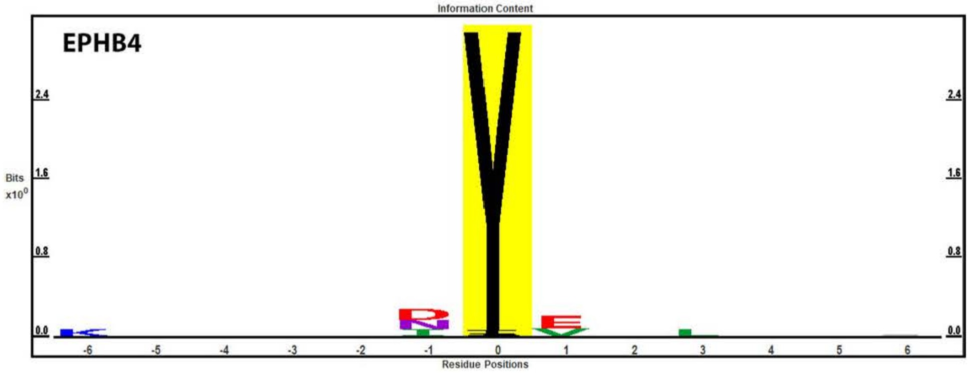
Information Content
EPHB4
Bits
x10^0
2.4
1.6
0.8
0.0
-6 -5 -4 -3 -2 -1 0 1 2 3 4 5 6
Residue Positions

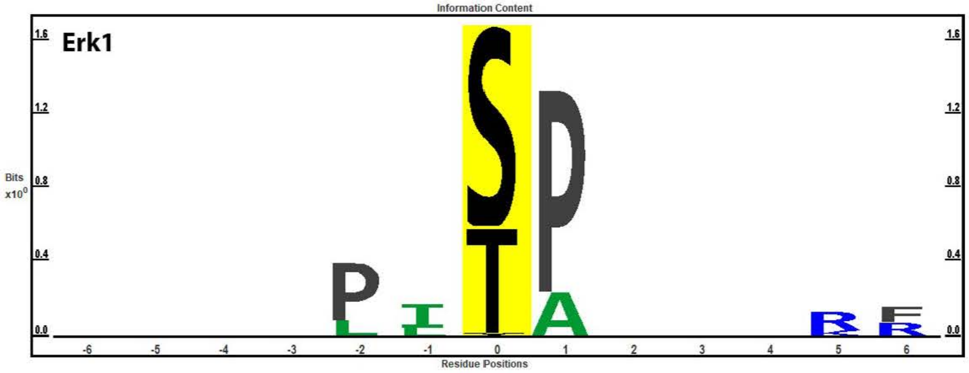
Information Content
Erk1
Bits
x10⁰
Residue Positions

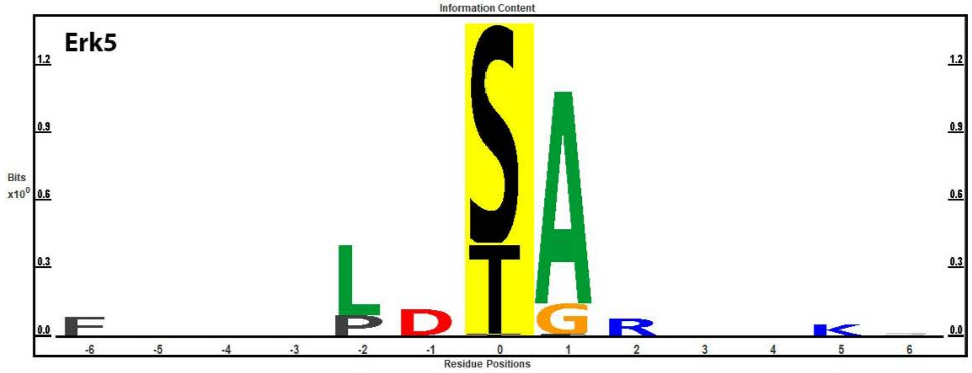
Information Content
Erk5
Bits
x10⁰
Residue Positions

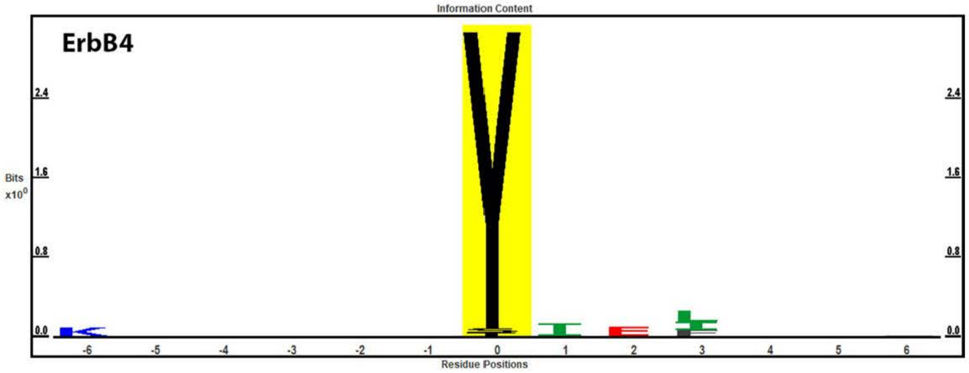
Information Content
ErbB4
Bits
x10⁰
Residue Positions

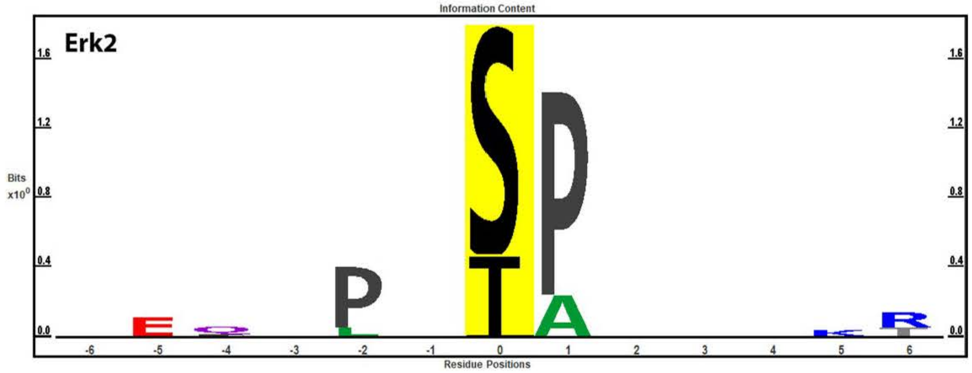
Information Content
Erk2
Bits
x10⁰
Residue Positions

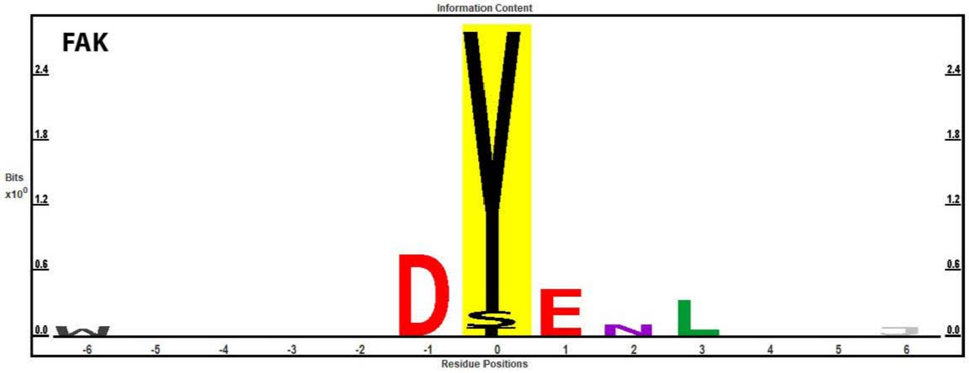
Information Content
FAK
Bits
x10^0
2.4
1.8
1.2
0.6
0.0
-6 -5 -4 -3 -2 -1 0 1 2 3 4 5 6
Residue Positions

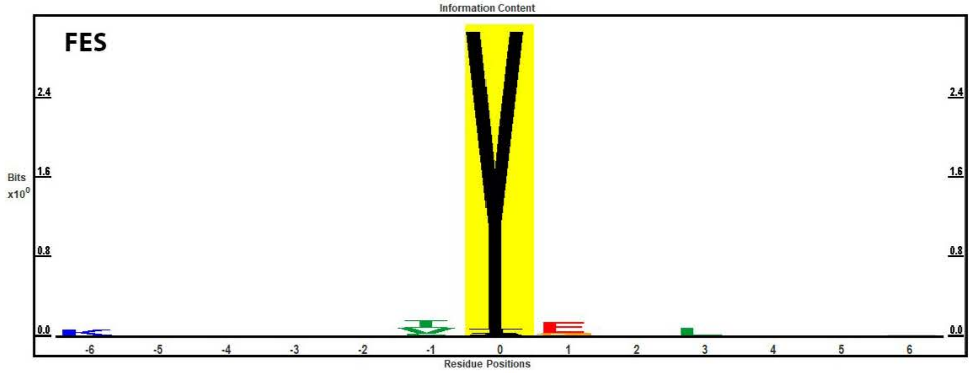
Information Content
FES
Bits
x10^0
2.4
1.6
0.8
0.0
-6 -5 -4 -3 -2 -1 0 1 2 3 4 5 6
Residue Positions

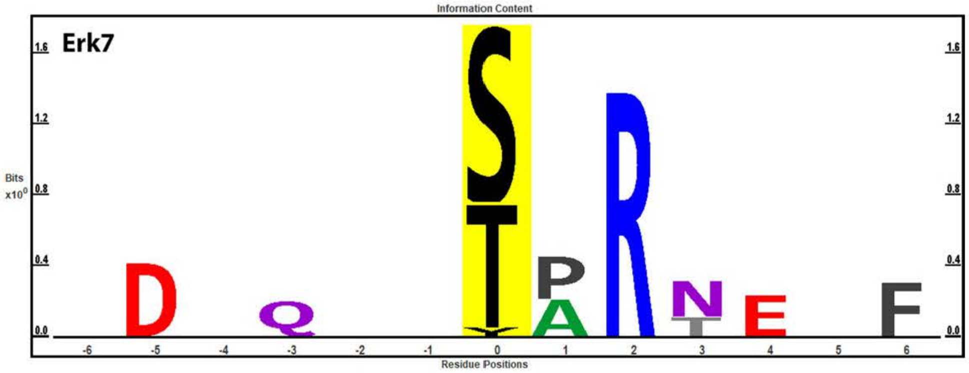
Information Content
Erk7
Bits
x10^0
1.6
1.2
0.8
0.4
0.0
-6 -5 -4 -3 -2 -1 0 1 2 3 4 5 6
Residue Positions

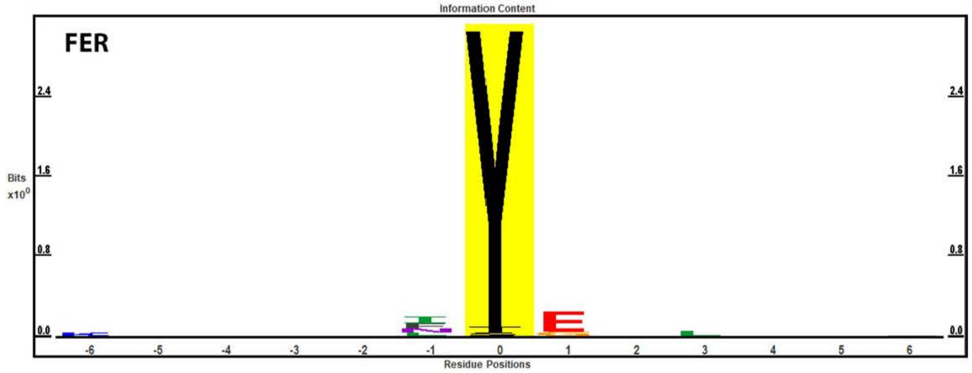
Information Content
FER
Bits
x10^0
2.4
1.6
0.8
0.0
-6 -5 -4 -3 -2 -1 0 1 2 3 4 5 6
Residue Positions

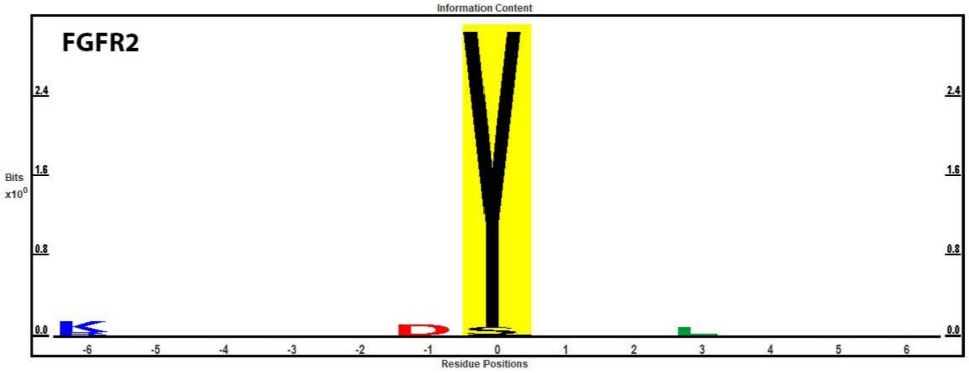
Information Content
FGFR2
Bits
x10^0
2.4
1.6
0.8
0.0
-6 -5 -4 -3 -2 -1 0 1 2 3 4 5 6
Residue Positions

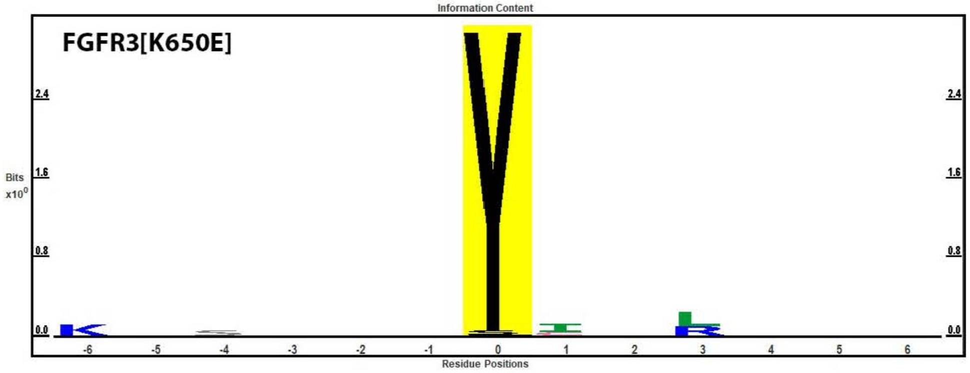
Information Content
FGFR3[K650E]
Bits
x10^0
2.4
1.6
0.8
0.0
-6 -5 -4 -3 -2 -1 0 1 2 3 4 5 6
Residue Positions

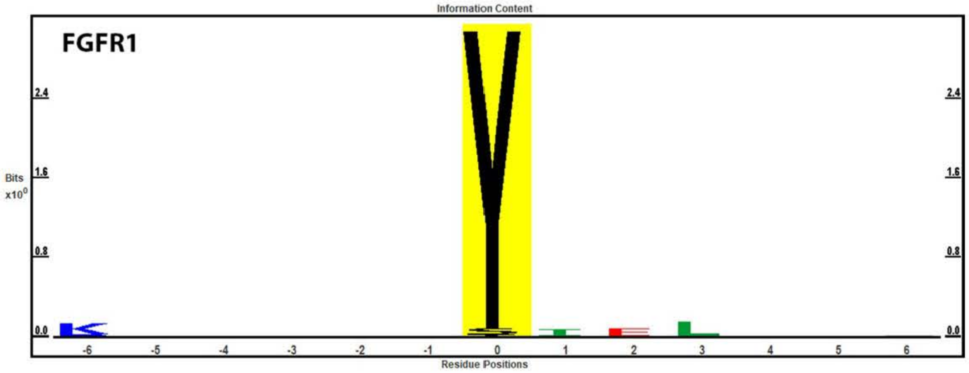
Information Content
FGFR1
Bits
x10^0
2.4
1.6
0.8
0.0
-6 -5 -4 -3 -2 -1 0 1 2 3 4 5 6
Residue Positions

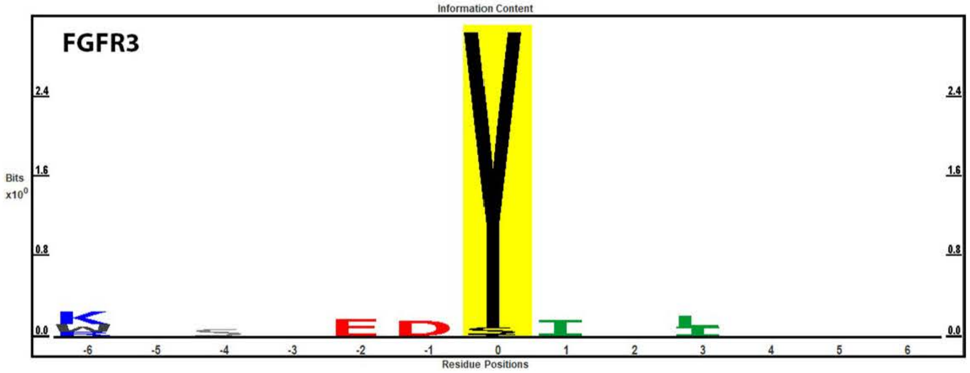
Information Content
FGFR3
Bits
x10^0
2.4
1.6
0.8
0.0
-6 -5 -4 -3 -2 -1 0 1 2 3 4 5 6
Residue Positions

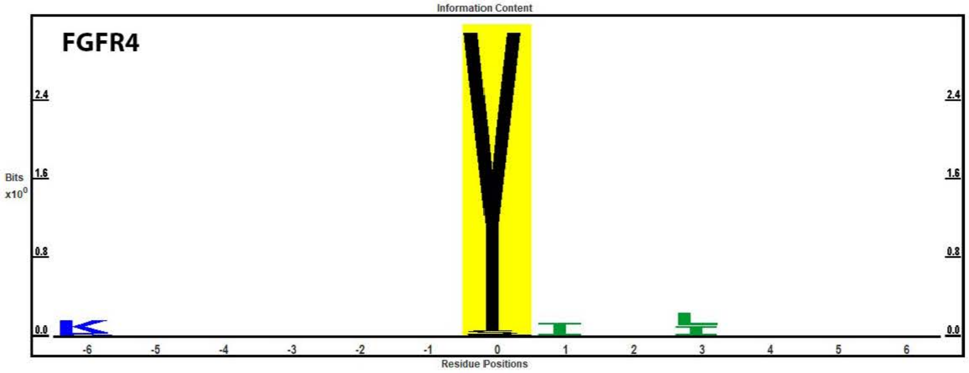
Information Content
FGFR4
Bits
x10^0
2.4
1.6
0.8
0.0
-6 -5 -4 -3 -2 -1 0 1 2 3 4 5 6
Residue Positions

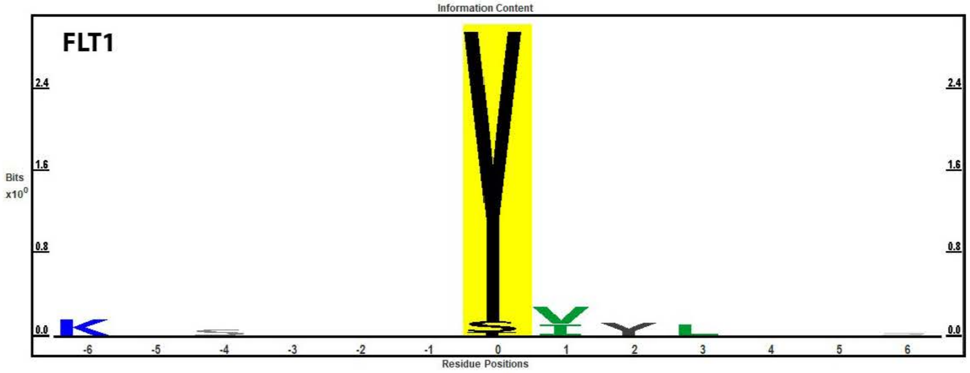
Information Content
FLT1
Bits
x10^0
2.4
1.6
0.8
0.0
-6 -5 -4 -3 -2 -1 0 1 2 3 4 5 6
Residue Positions

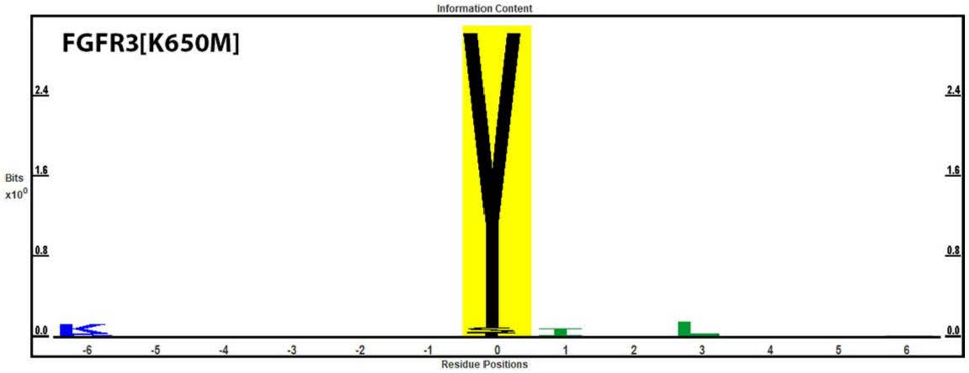
Information Content
FGFR3[K650M]
Bits
x10^0
2.4
1.6
0.8
0.0
-6 -5 -4 -3 -2 -1 0 1 2 3 4 5 6
Residue Positions

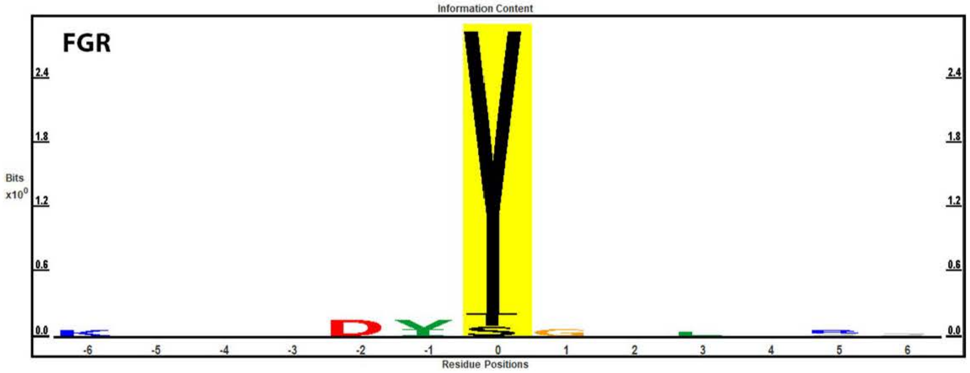
Information Content
FGR
Bits
x10^0
2.4
1.8
1.2
0.6
0.0
-6 -5 -4 -3 -2 -1 0 1 2 3 4 5 6
Residue Positions

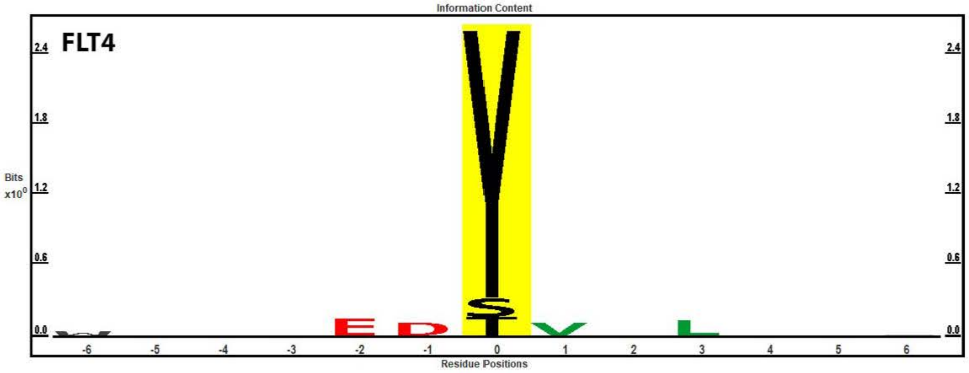
FLT4
Information Content
Bits
x10^0
Residue Positions

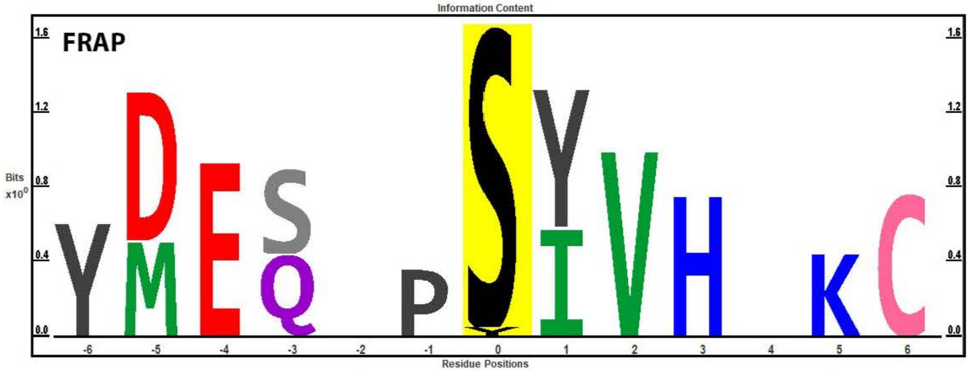
FRAP
Information Content
Bits
x10^0
Residue Positions

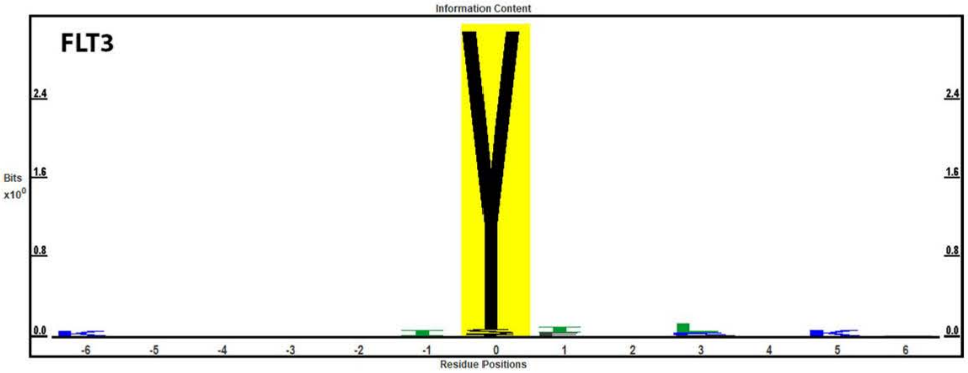
FLT3
Information Content
Bits
x10^0
Residue Positions

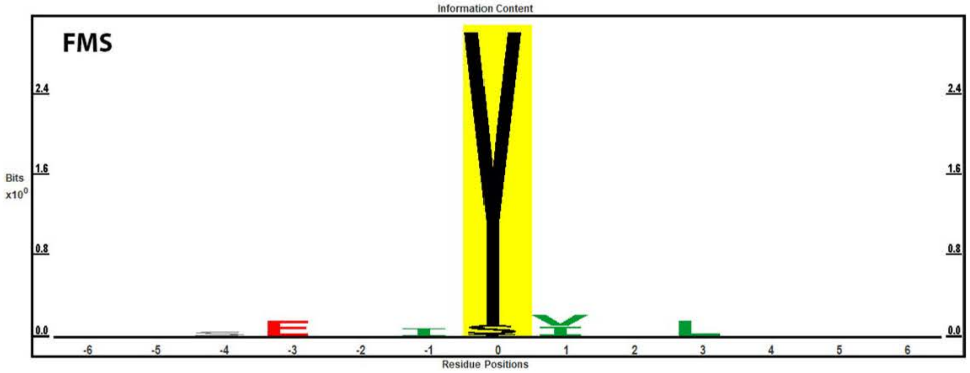
FMS
Information Content
Bits
x10^0
Residue Positions

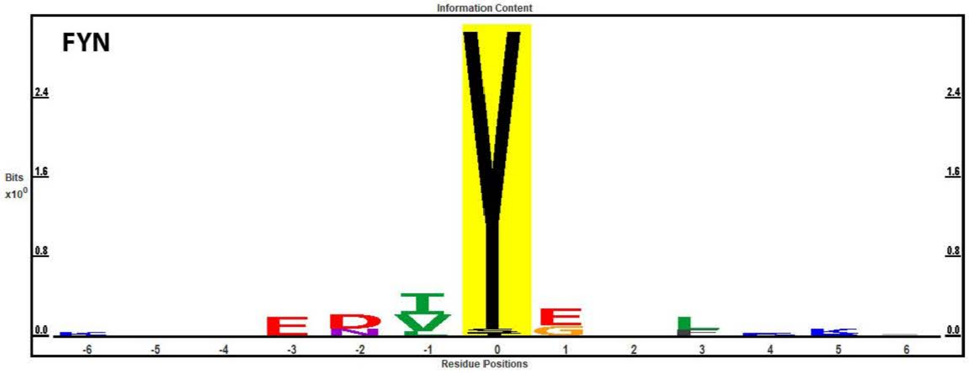
Information Content
FYN
Bits x10^0
2.4
1.6
0.8
0.0
-6 -5 -4 -3 -2 -1 0 1 2 3 4 5 6
Residue Positions

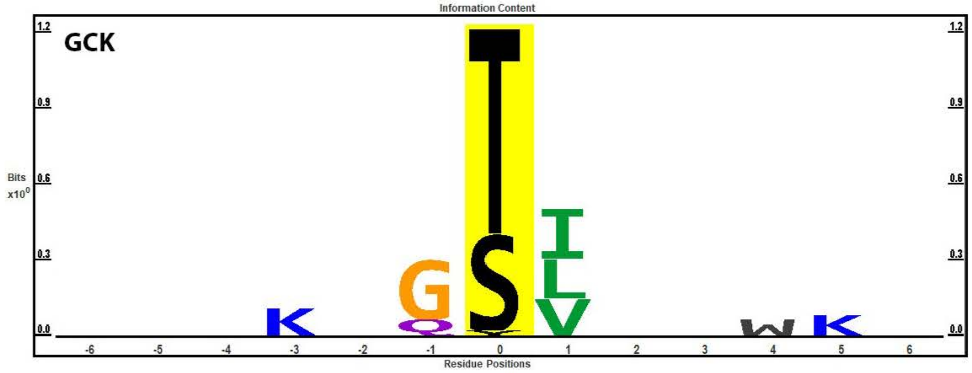
Information Content
GCK
Bits x10^0
1.2
0.9
0.6
0.3
0.0
-6 -5 -4 -3 -2 -1 0 1 2 3 4 5 6
Residue Positions

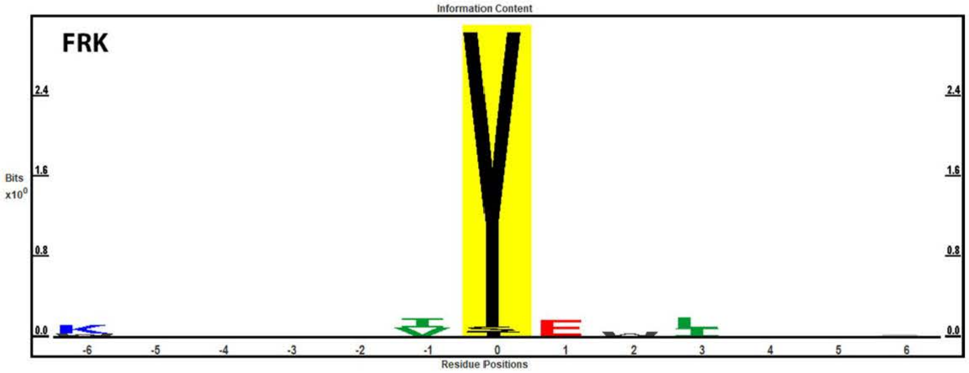
Information Content
FRK
Bits x10^0
2.4
1.6
0.8
0.0
-6 -5 -4 -3 -2 -1 0 1 2 3 4 5 6
Residue Positions

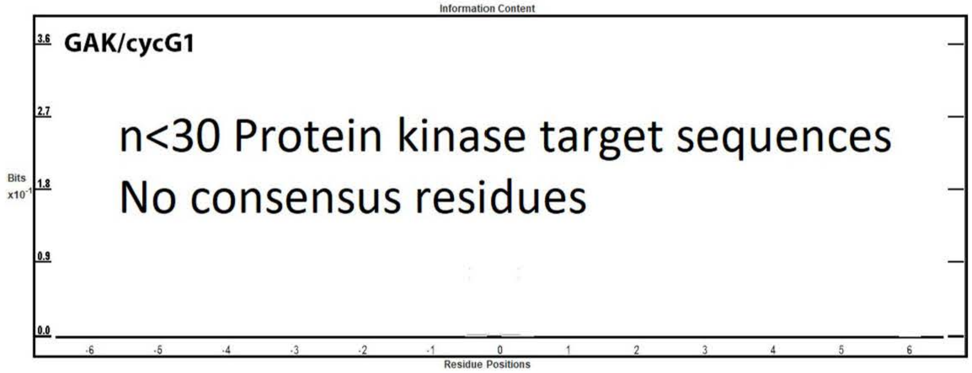
Information Content
GAK/cycG1
n<30 Protein kinase target sequences
No consensus residues
Bits x10^-1
3.6
2.7
1.8
0.9
0.0
-6 -5 -4 -3 -2 -1 0 1 2 3 4 5 6
Residue Positions

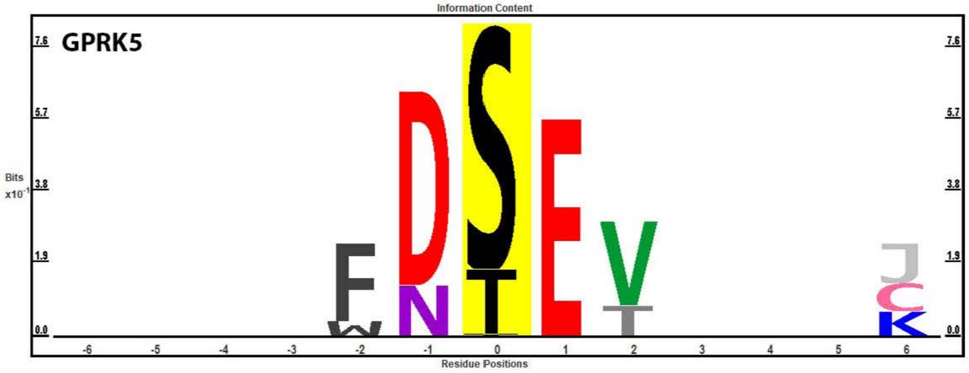
Information Content
GPRK5
Bits
x10-1
Residue Positions

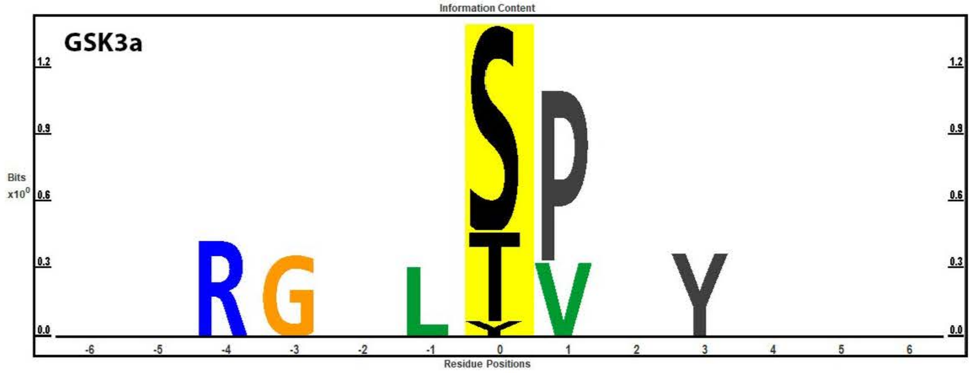
Information Content
GSK3a
Bits
x100
Residue Positions

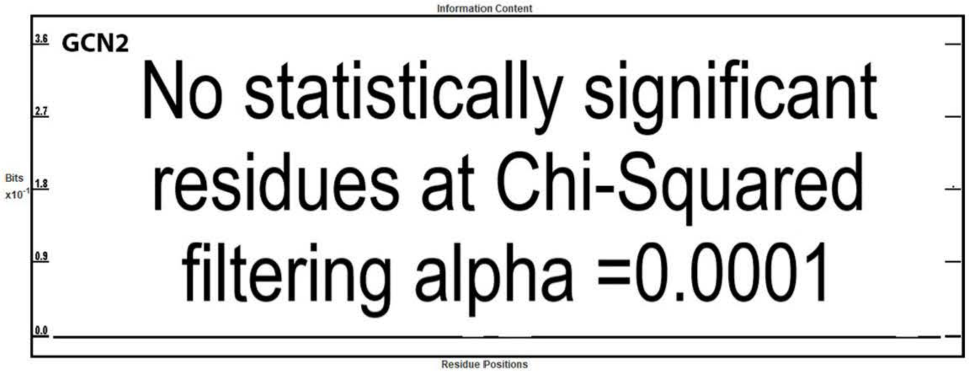
Information Content
GCN2
No statistically significant
residues at Chi-Squared
filtering alpha =0.0001
Bits
x10-1
Residue Positions

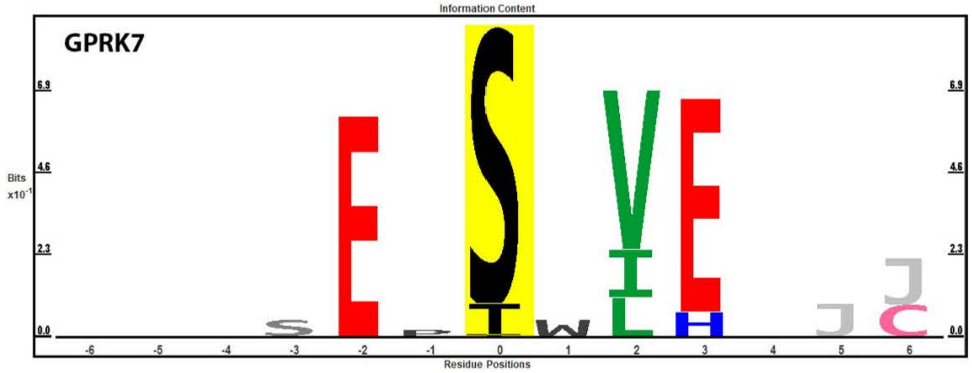
Information Content
GPRK7
Bits
x10-1
Residue Positions

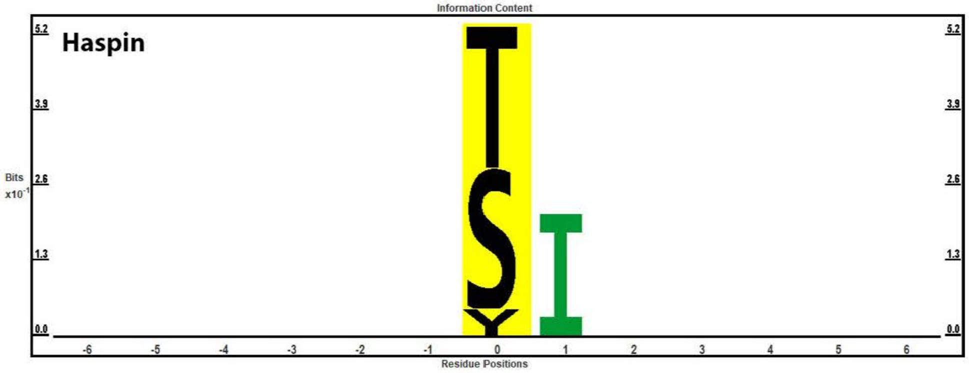
Information Content
Haspin
Bits
x10-1
5.2
3.9
2.6
1.3
0.0
-6 -5 -4 -3 -2 -1 0 1 2 3 4 5 6
Residue Positions

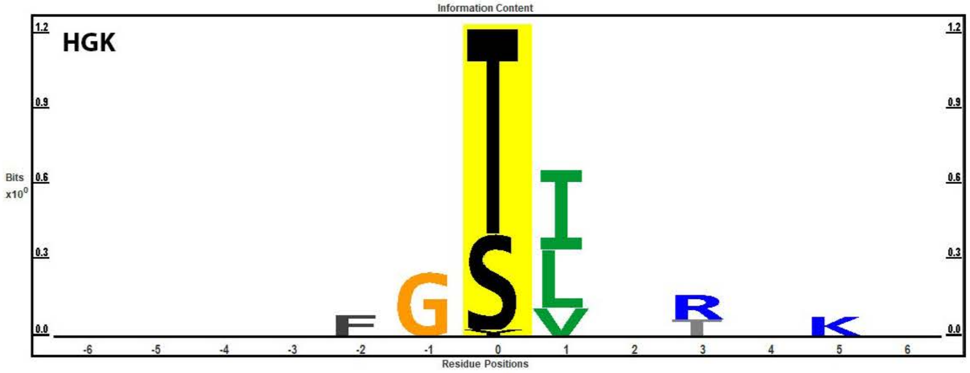
Information Content
HGK
Bits
x10^0
1.2
0.9
0.6
0.3
0.0
-6 -5 -4 -3 -2 -1 0 1 2 3 4 5 6
Residue Positions

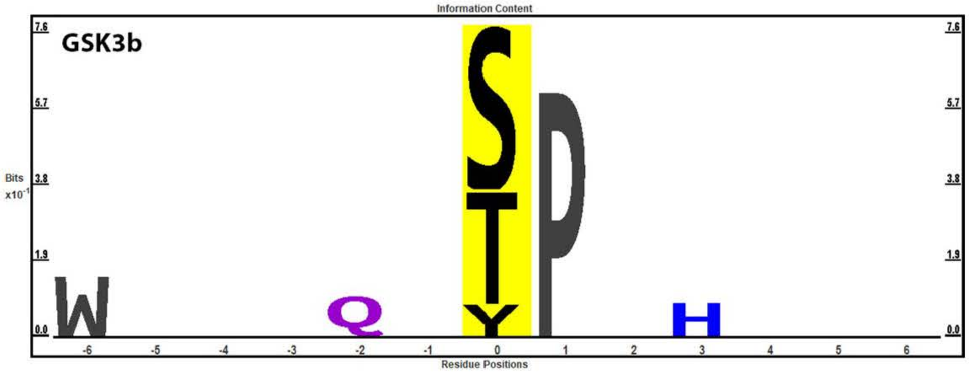
Information Content
GSK3b
Bits
x10-1
7.6
5.7
3.8
1.9
0.0
-6 -5 -4 -3 -2 -1 0 1 2 3 4 5 6
Residue Positions

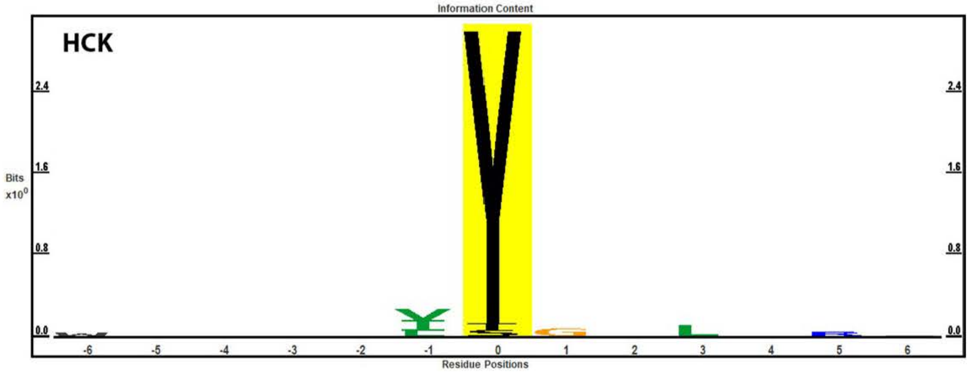
Information Content
HCK
Bits
x10^0
2.4
1.6
0.8
0.0
-6 -5 -4 -3 -2 -1 0 1 2 3 4 5 6
Residue Positions

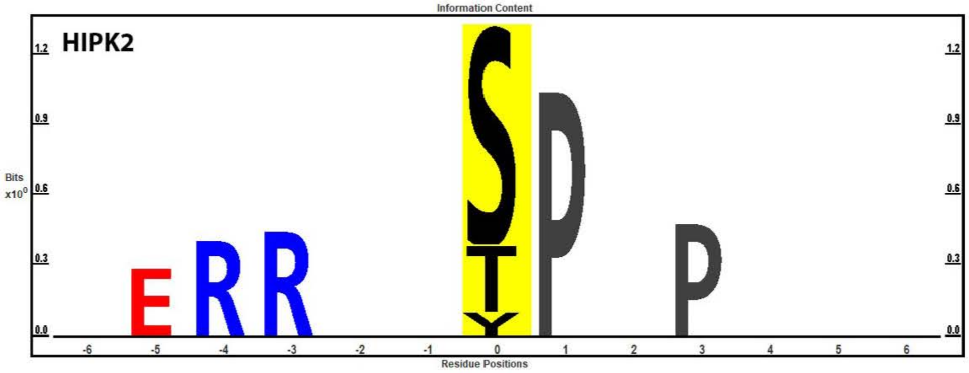
HIPK2
Information Content
Bits x10^0
Residue Positions

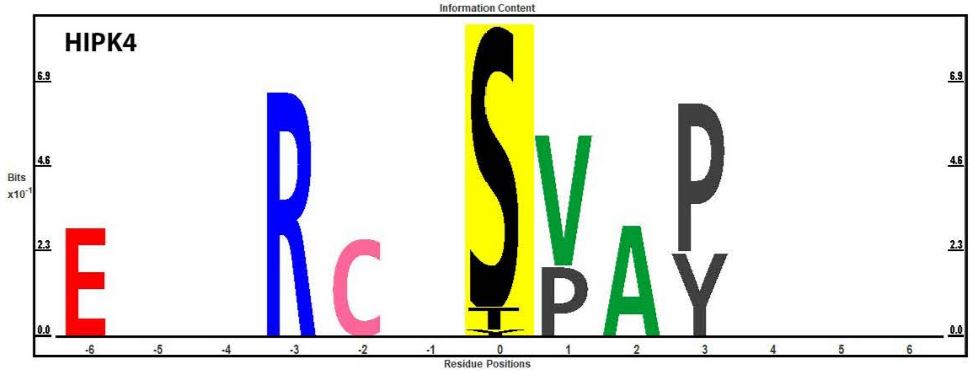
HIPK4
Information Content
Bits x10^-1
Residue Positions

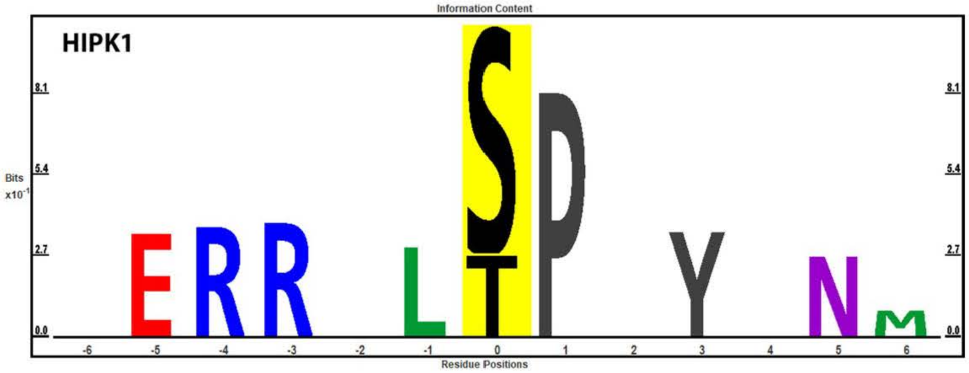
HIPK1
Information Content
Bits x10^-1
Residue Positions

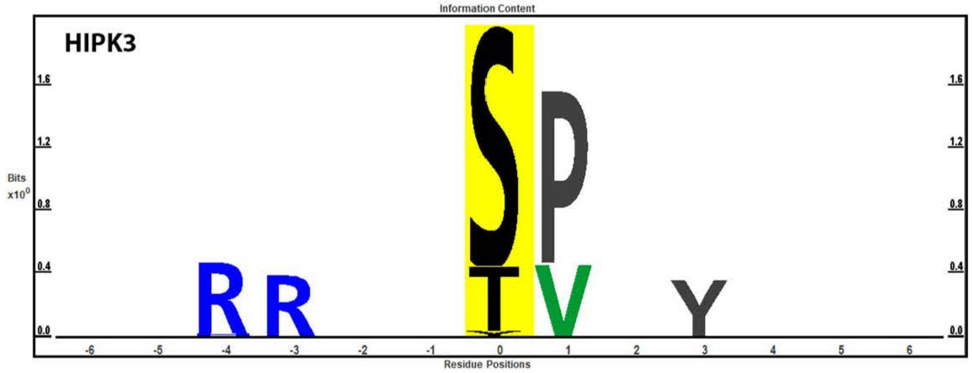
HIPK3
Information Content
Bits x10^0
Residue Positions

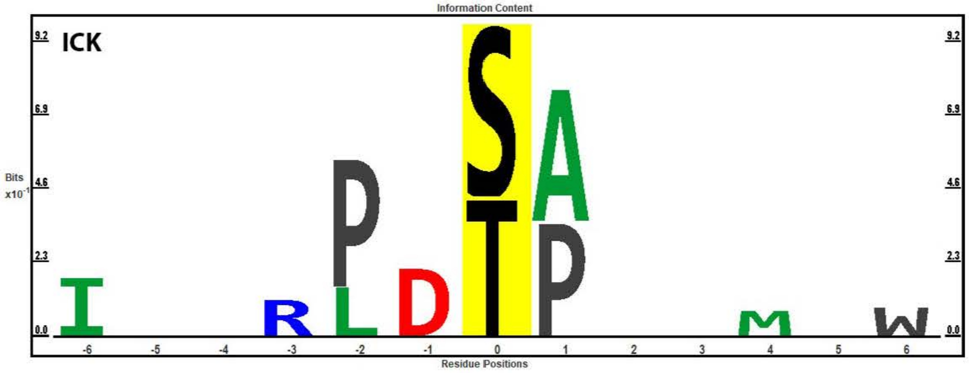
Information Content
ICK
Bits
x10-1
Residue Positions

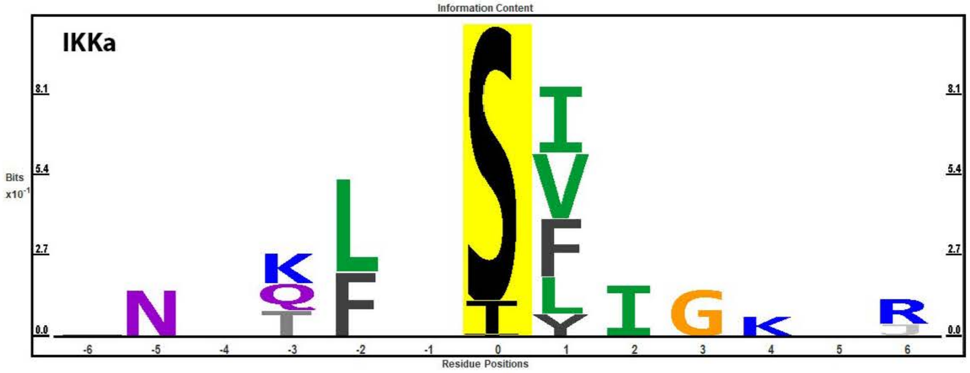
Information Content
IKKa
Bits
x10-1
Residue Positions

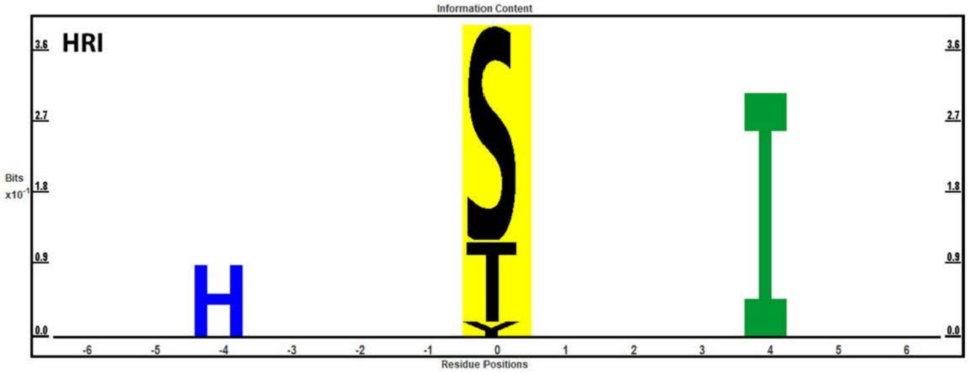
Information Content
HRI
Bits
x10-1
Residue Positions

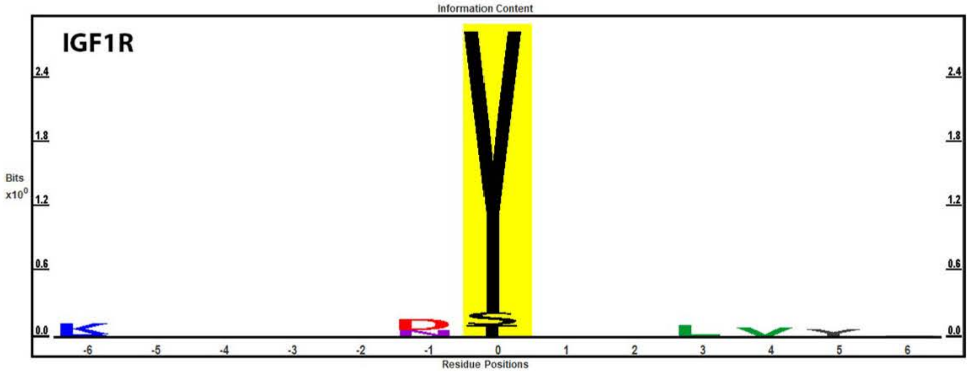
Information Content
IGF1R
Bits
x10^0
Residue Positions

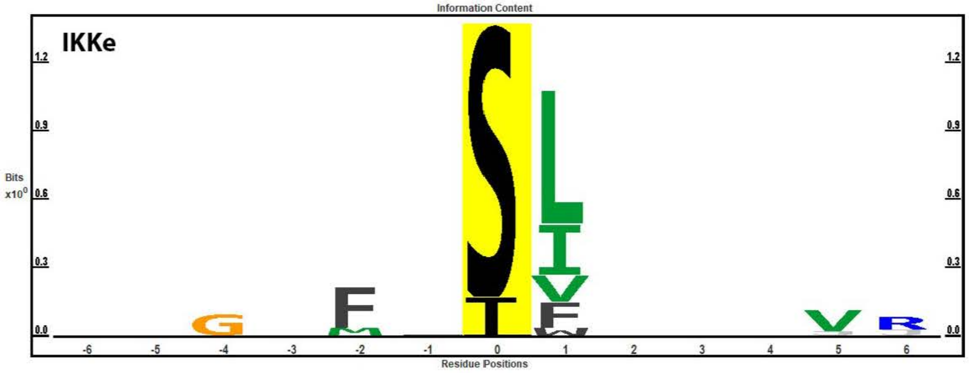
Information Content
IKKe
Bits
x10^0
Residue Positions

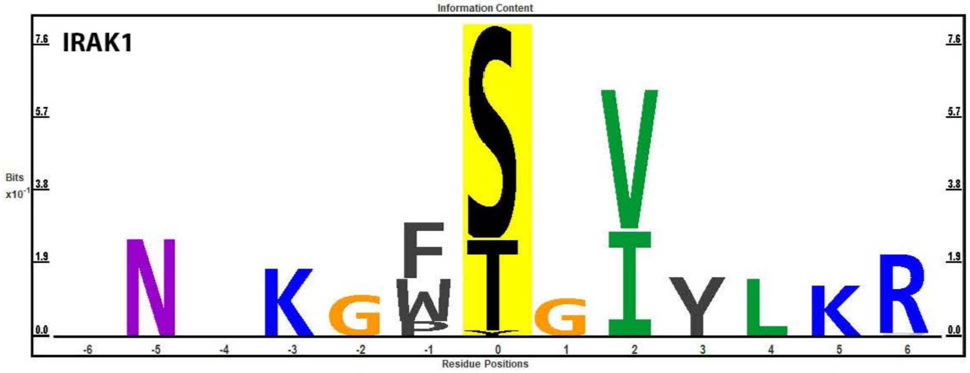
Information Content
IRAK1
Bits
x10^-1
Residue Positions

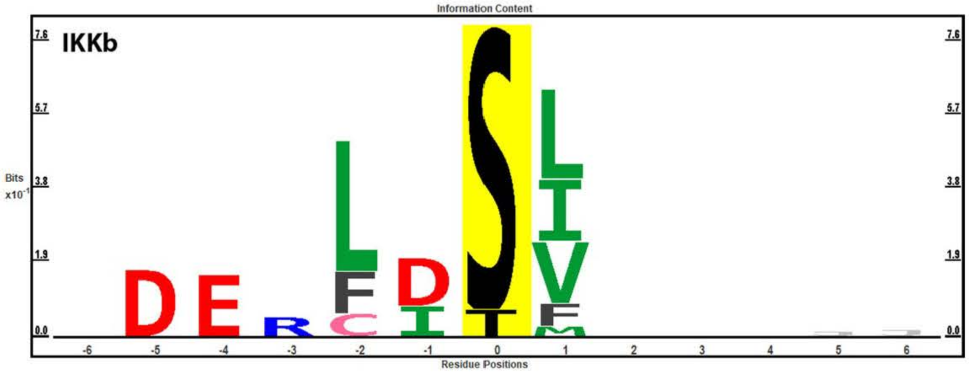
Information Content
IKKb
Bits
x10^-1
Residue Positions

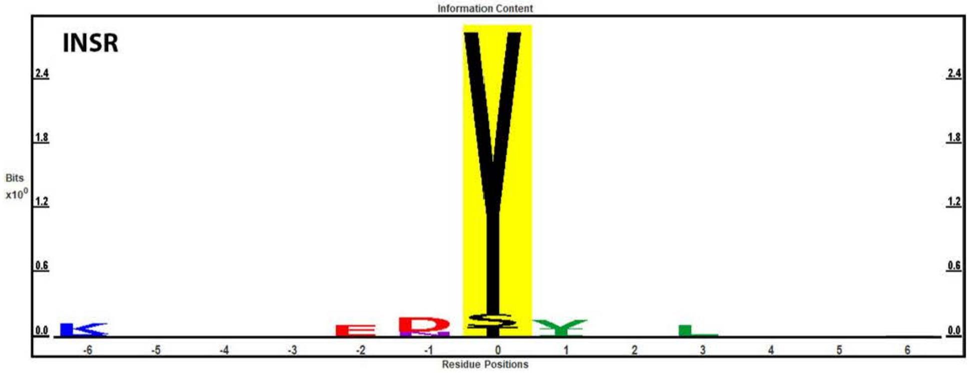
Information Content
INSR
Bits
x10^0
Residue Positions

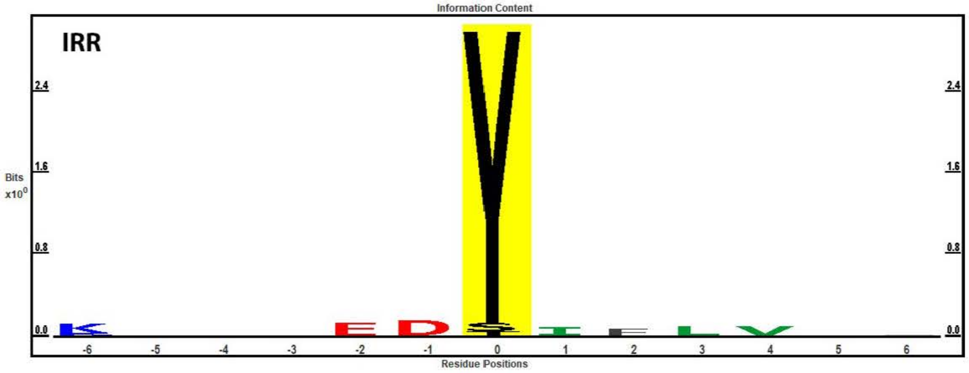
Information Content
IRR
Bits
x10^0
Residue Positions

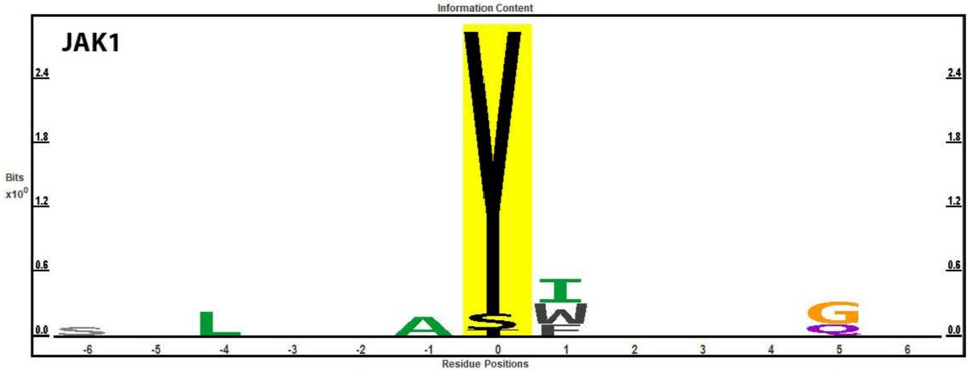
Information Content
JAK1
Bits
x10^0
Residue Positions

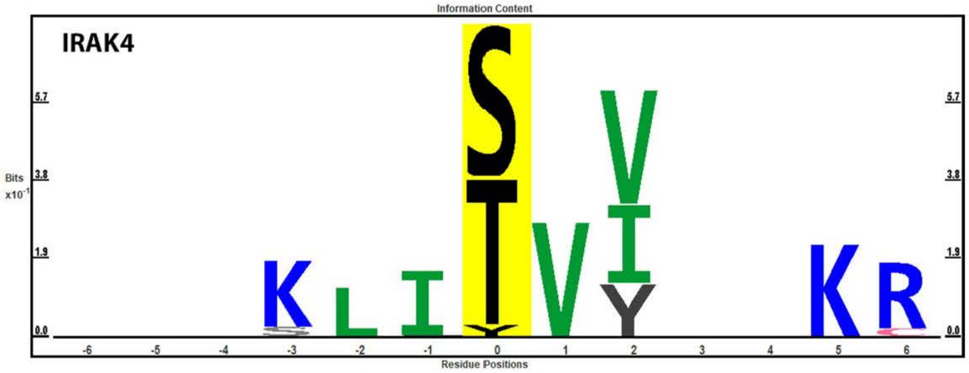
Information Content
IRAK4
Bits
x10^-1
Residue Positions

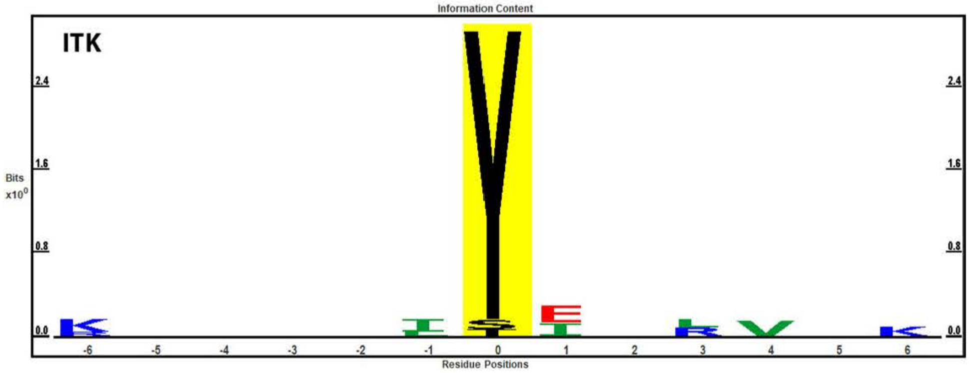
Information Content
ITK
Bits
x10^0
Residue Positions

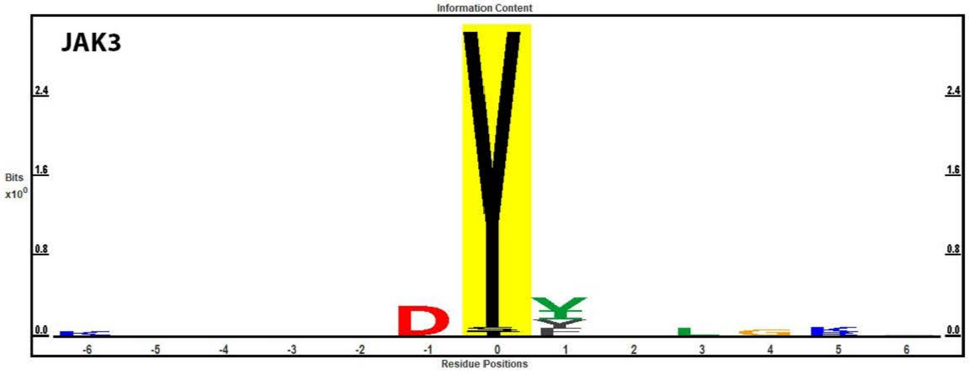
Information Content
JAK3
Bits
x10^0
0.0
0.8
1.6
2.4
-6 -5 -4 -3 -2 -1 0 1 2 3 4 5 6
Residue Positions

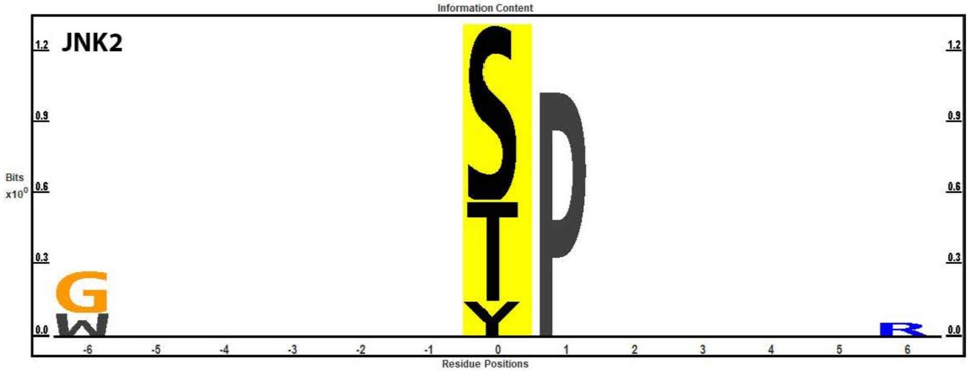
Information Content
JNK2
Bits
x10^0
0.0
0.3
0.6
0.9
1.2
-6 -5 -4 -3 -2 -1 0 1 2 3 4 5 6
Residue Positions

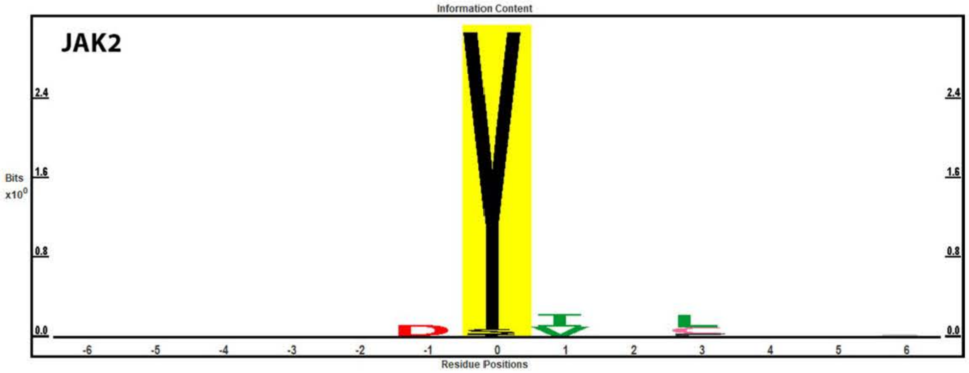
Information Content
JAK2
Bits
x10^0
0.0
0.8
1.6
2.4
-6 -5 -4 -3 -2 -1 0 1 2 3 4 5 6
Residue Positions

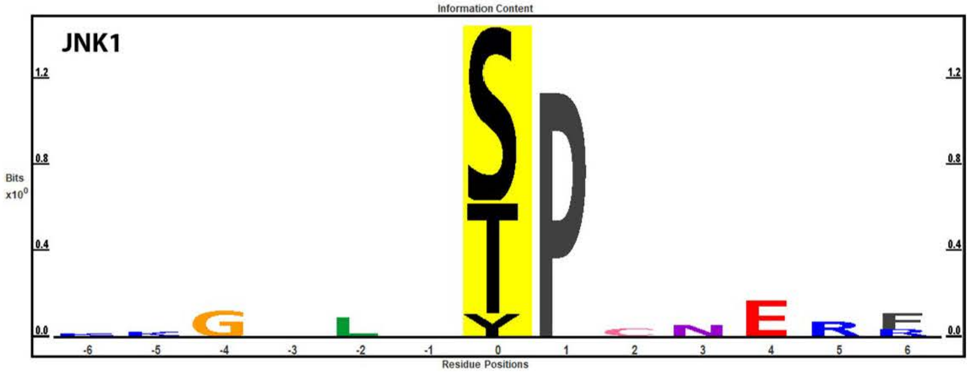
Information Content
JNK1
Bits
x10^0
0.0
0.4
0.8
1.2
-6 -5 -4 -3 -2 -1 0 1 2 3 4 5 6
Residue Positions

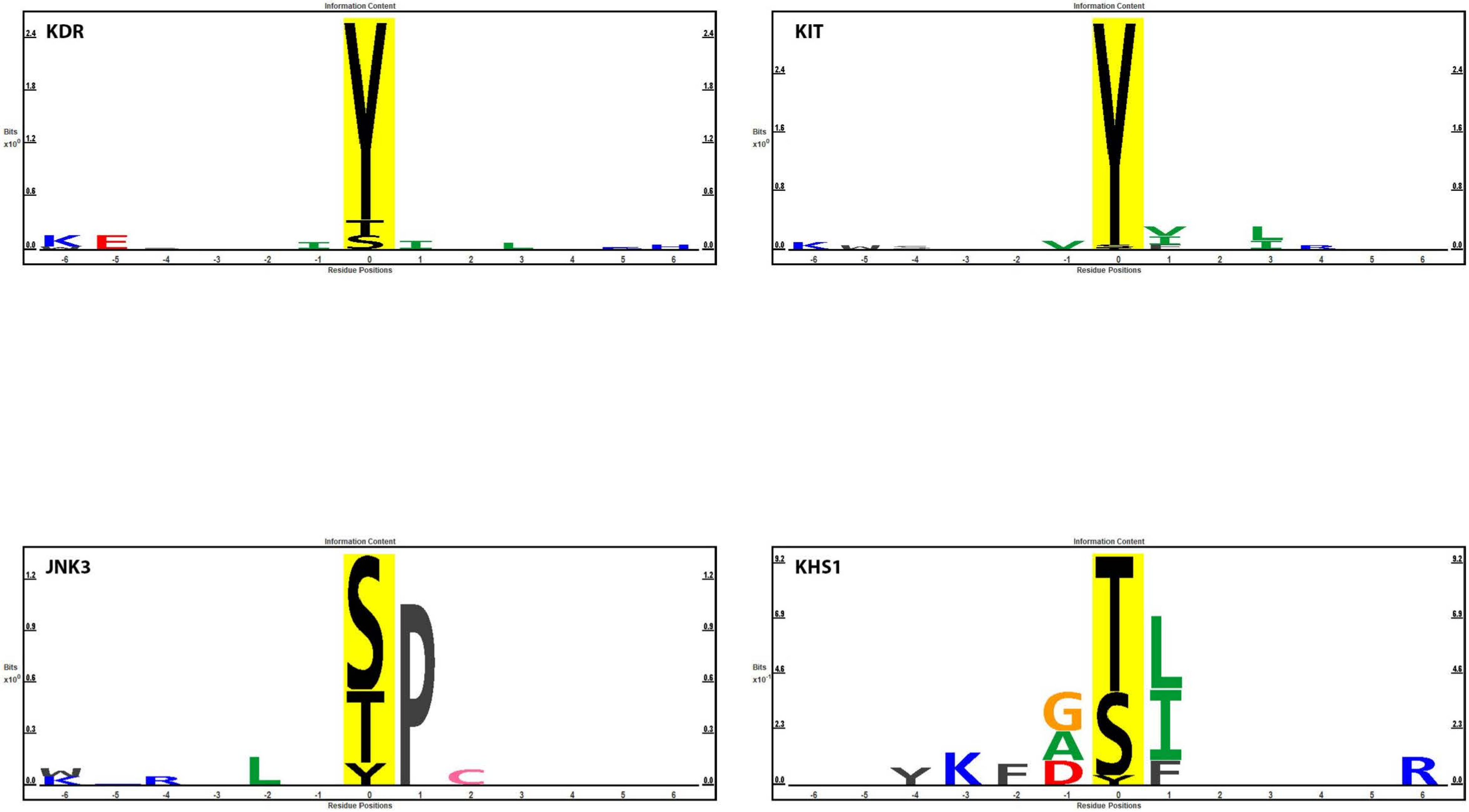
KDR
KIT
JNK3
KHS1
Information Content
Bits
Residue Positions

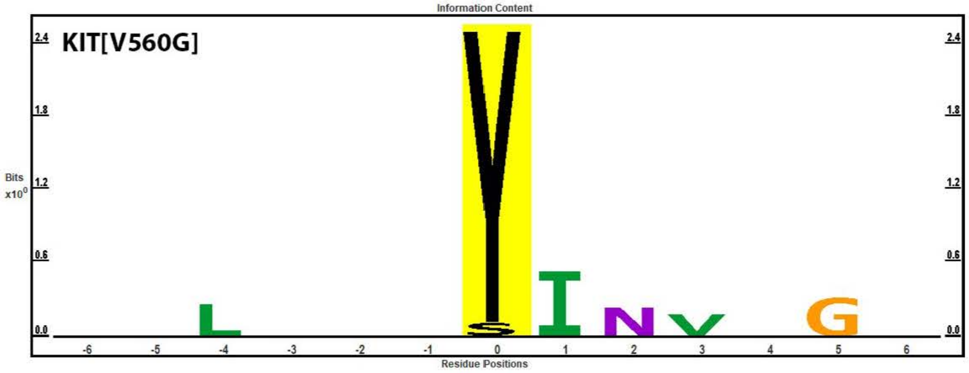
Information Content
KIT[V560G]
Bits
x10^0
2.4
1.8
1.2
0.6
0.0
-6 -5 -4 -3 -2 -1 0 1 2 3 4 5 6
Residue Positions

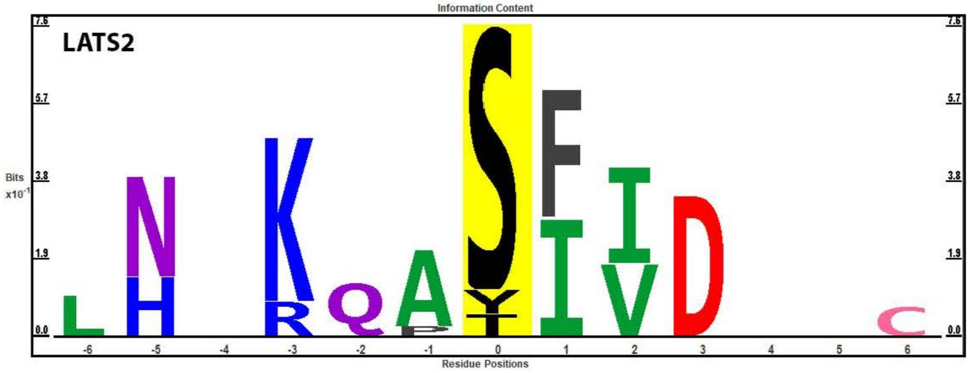
Information Content
LATS2
Bits
x10^-1
7.6
5.7
3.8
1.9
0.0
-6 -5 -4 -3 -2 -1 0 1 2 3 4 5 6
Residue Positions

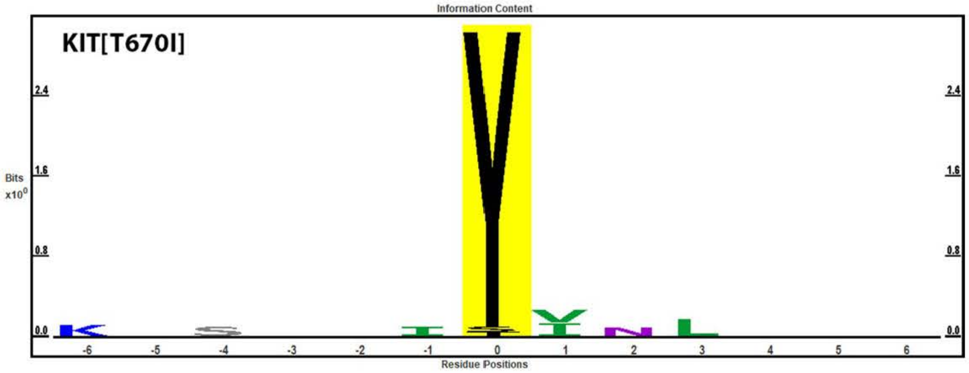
Information Content
KIT[T670I]
Bits
x10^0
2.4
1.6
0.8
0.0
-6 -5 -4 -3 -2 -1 0 1 2 3 4 5 6
Residue Positions

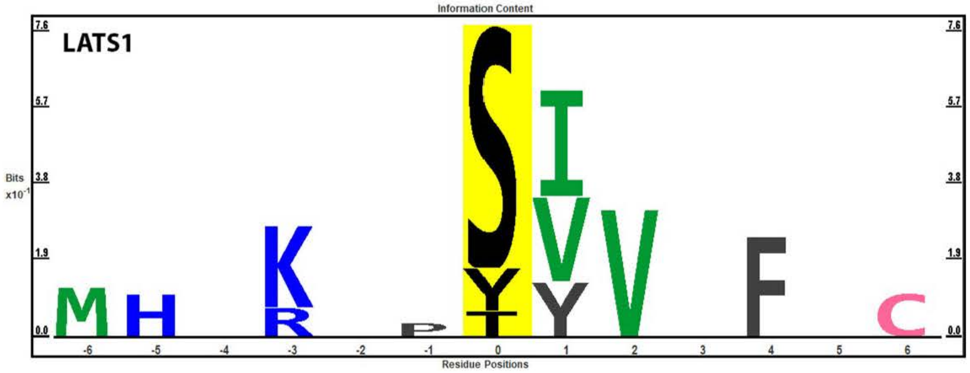
Information Content
LATS1
Bits
x10^-1
7.6
5.7
3.8
1.9
0.0
-6 -5 -4 -3 -2 -1 0 1 2 3 4 5 6
Residue Positions

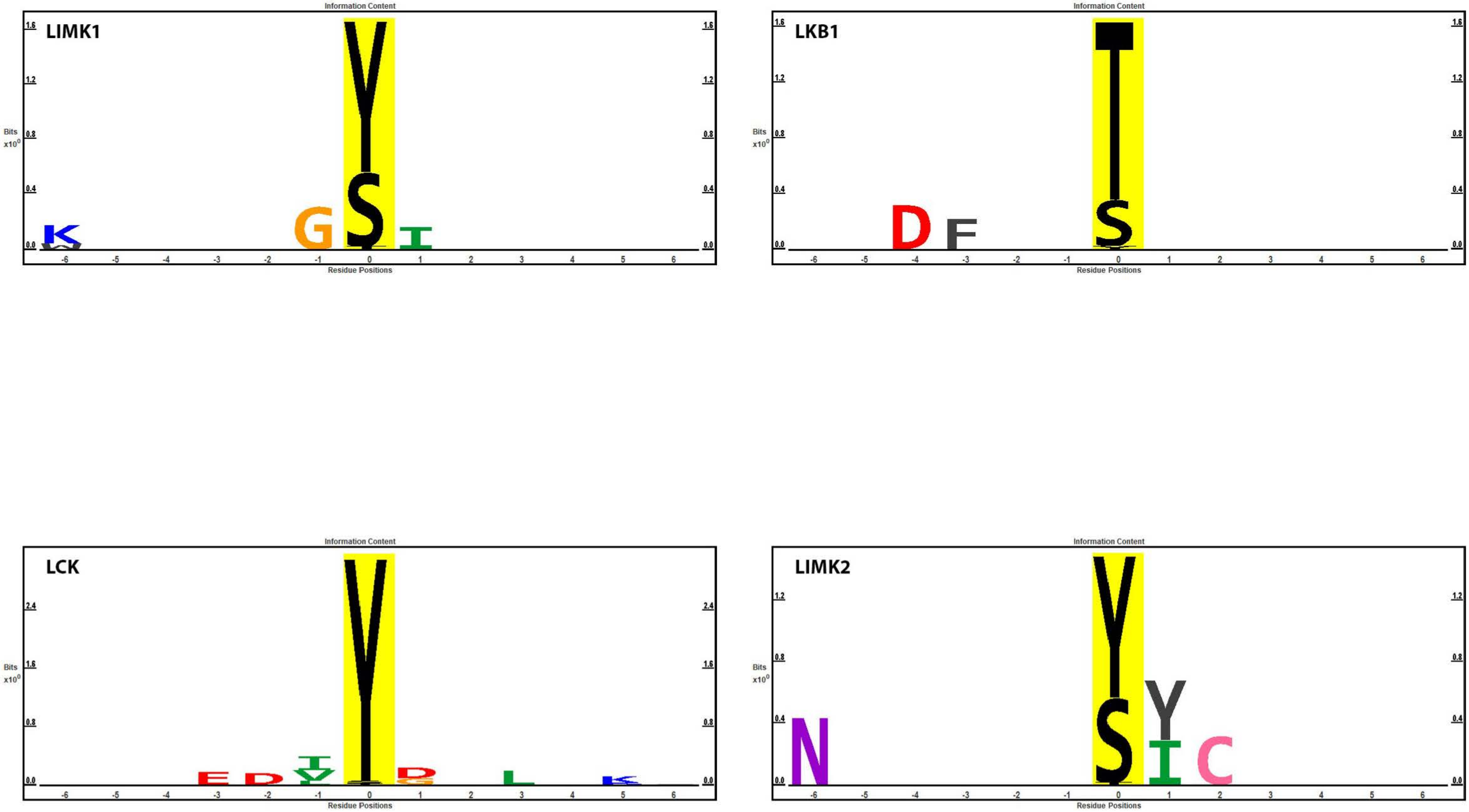
LIMK1
LKB1
LCK
LIMK2
Information Content
Bits x10^0
Residue Positions

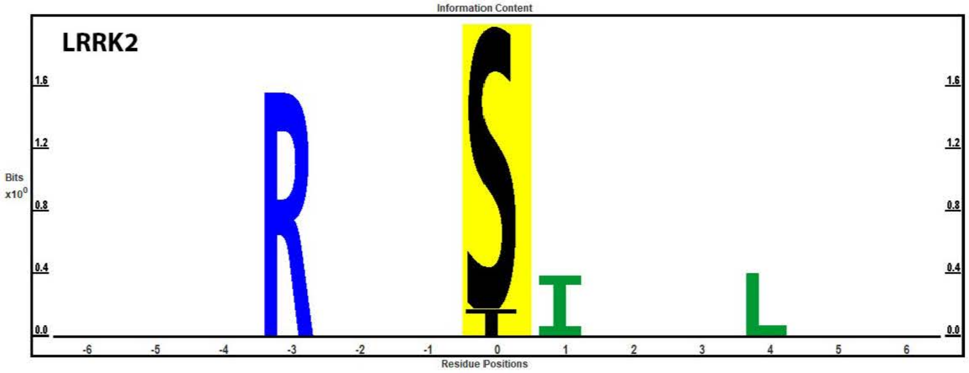
Information Content
LRRK2
Bits
x10^0
Residue Positions

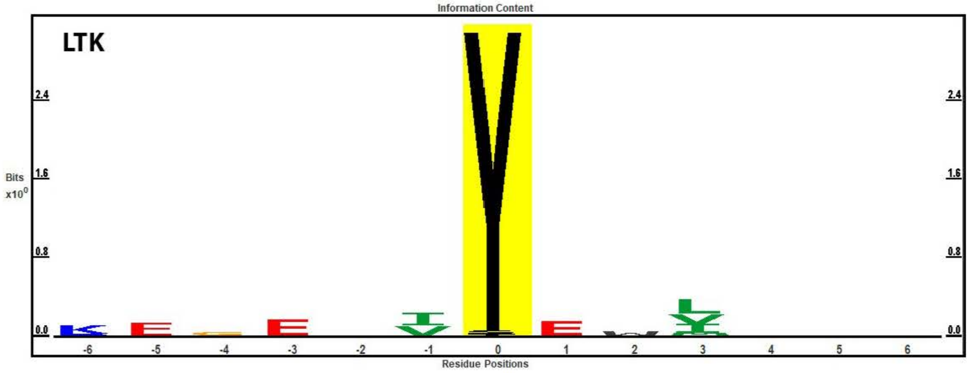
Information Content
LTK
Bits
x10^0
Residue Positions

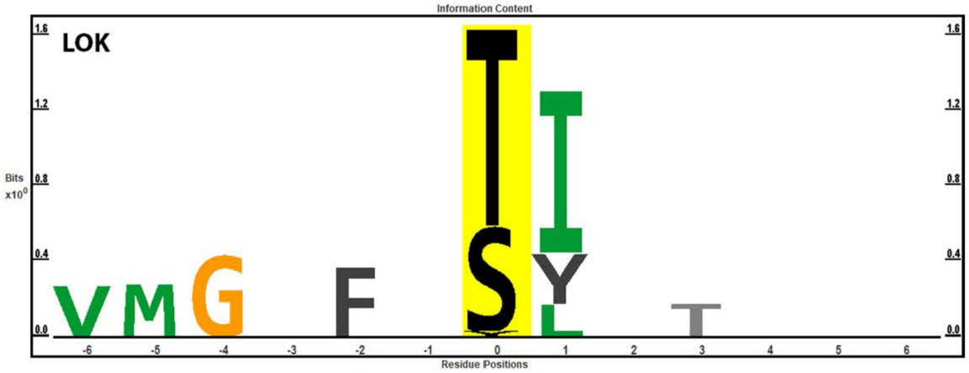
Information Content
LOK
Bits
x10^0
Residue Positions

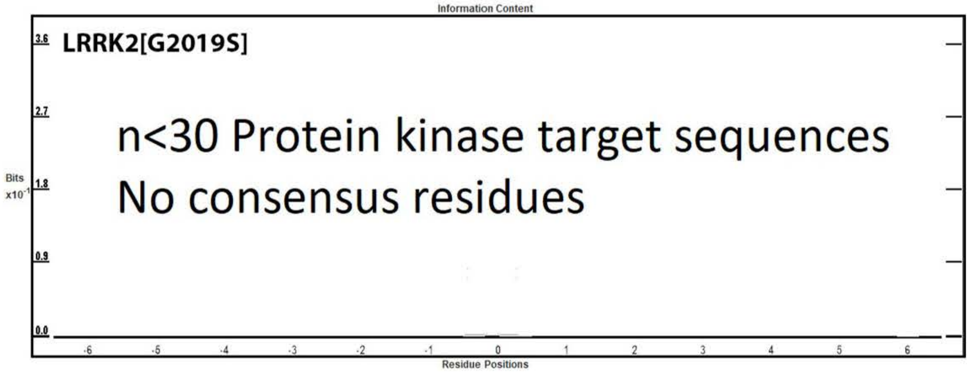
Information Content
LRRK2[G2019S]
n<30 Protein kinase target sequences
No consensus residues
Bits
x10^-1
Residue Positions

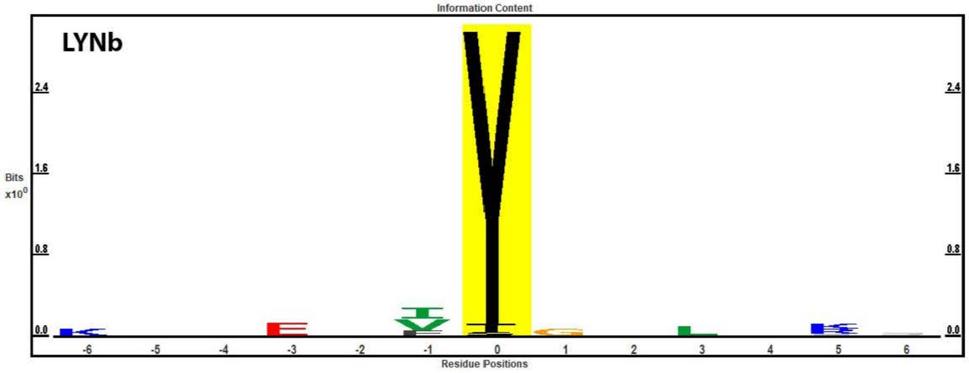
Information Content
LYNb
Bits
x10^0
2.4
1.6
0.8
0.0
-6 -5 -4 -3 -2 -1 0 1 2 3 4 5 6
Residue Positions

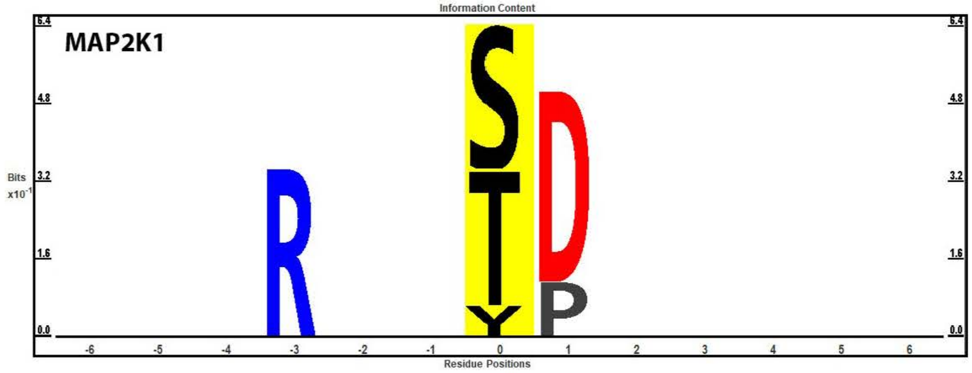
Information Content
MAP2K1
Bits
x10^-1
6.4
4.8
3.2
1.6
0.0
-6 -5 -4 -3 -2 -1 0 1 2 3 4 5 6
Residue Positions

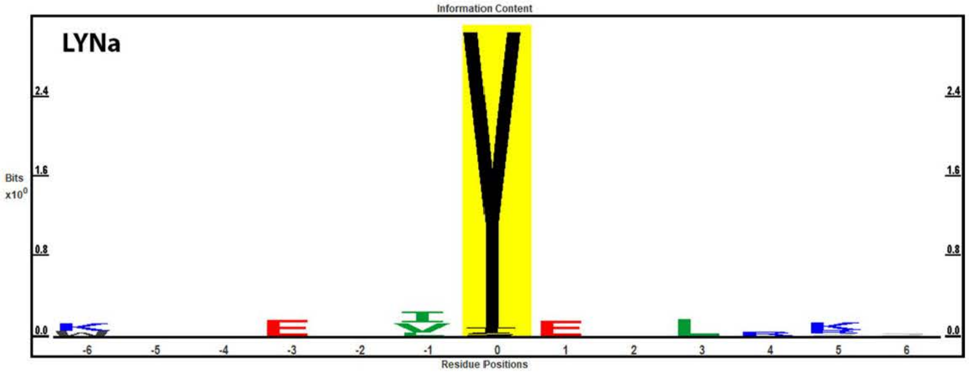
Information Content
LYNa
Bits
x10^0
2.4
1.6
0.8
0.0
-6 -5 -4 -3 -2 -1 0 1 2 3 4 5 6
Residue Positions

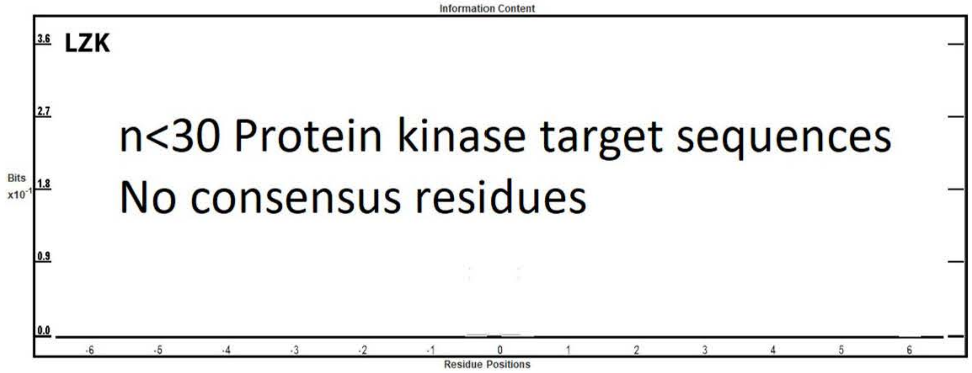
Information Content
LZK
n<30 Protein kinase target sequences
No consensus residues
Bits
x10^-1
3.6
2.7
1.8
0.9
0.0
-6 -5 -4 -3 -2 -1 0 1 2 3 4 5 6
Residue Positions

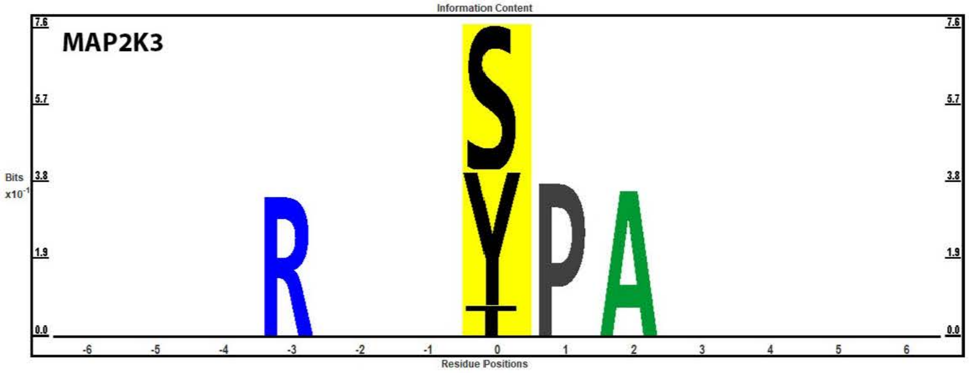
Information Content
MAP2K3
Bits x10-1
7.6
5.7
3.8
1.9
0.0
-6 -5 -4 -3 -2 -1 0 1 2 3 4 5 6
Residue Positions

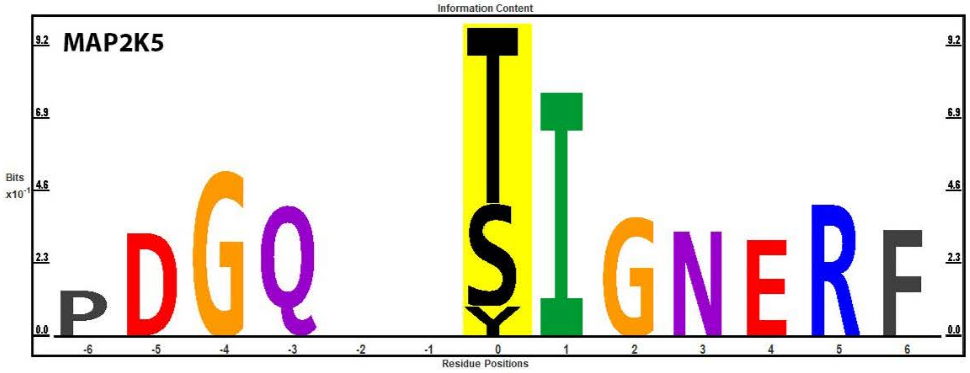
Information Content
MAP2K5
Bits x10-1
9.2
6.9
4.6
2.3
0.0
-6 -5 -4 -3 -2 -1 0 1 2 3 4 5 6
Residue Positions

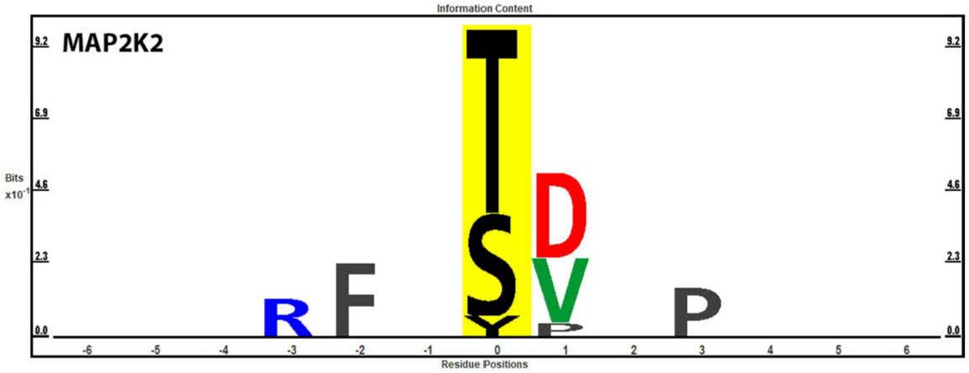
Information Content
MAP2K2
Bits x10-1
9.2
6.9
4.6
2.3
0.0
-6 -5 -4 -3 -2 -1 0 1 2 3 4 5 6
Residue Positions

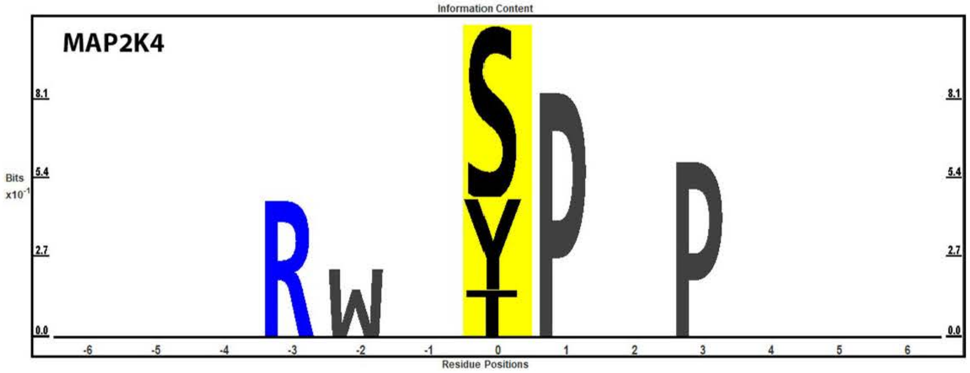
Information Content
MAP2K4
Bits x10-1
8.1
5.4
2.7
0.0
-6 -5 -4 -3 -2 -1 0 1 2 3 4 5 6
Residue Positions

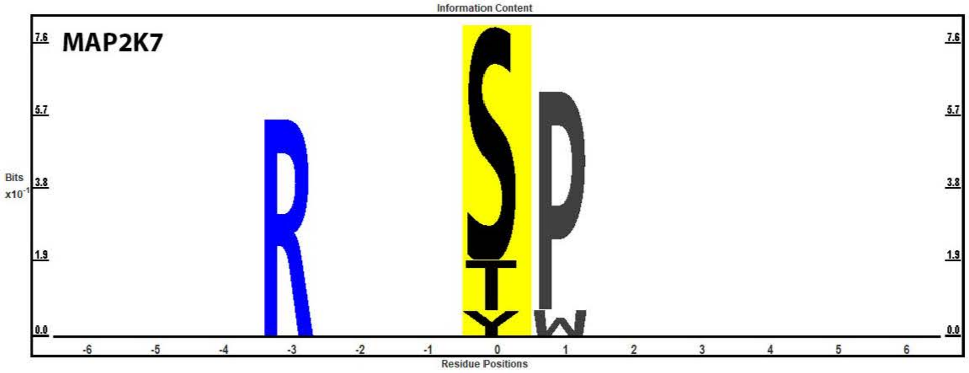
Information Content
MAP2K7
Bits
x10-1
7.6
5.7
3.8
1.9
0.0
-6 -5 -4 -3 -2 -1 0 1 2 3 4 5 6
Residue Positions

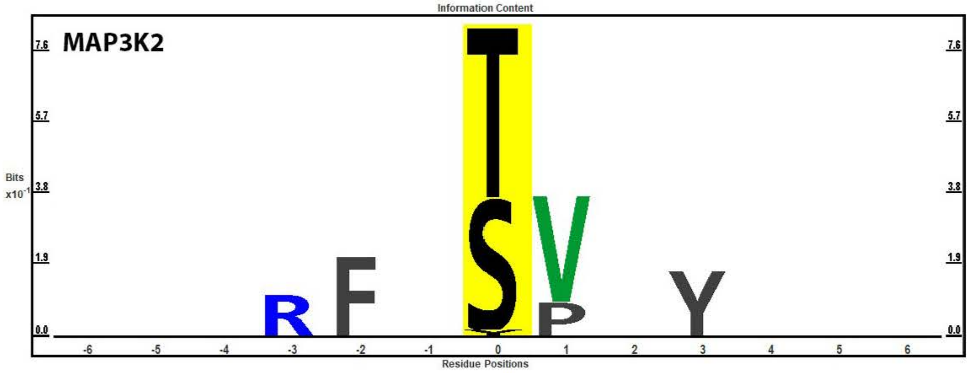
Information Content
MAP3K2
Bits
x10-1
7.6
5.7
3.8
1.9
0.0
-6 -5 -4 -3 -2 -1 0 1 2 3 4 5 6
Residue Positions

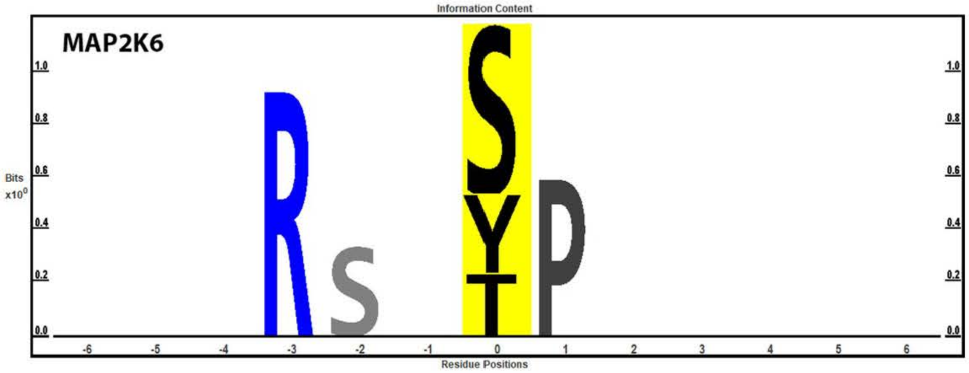
Information Content
MAP2K6
Bits
x10^0
1.0
0.8
0.6
0.4
0.2
0.0
-6 -5 -4 -3 -2 -1 0 1 2 3 4 5 6
Residue Positions

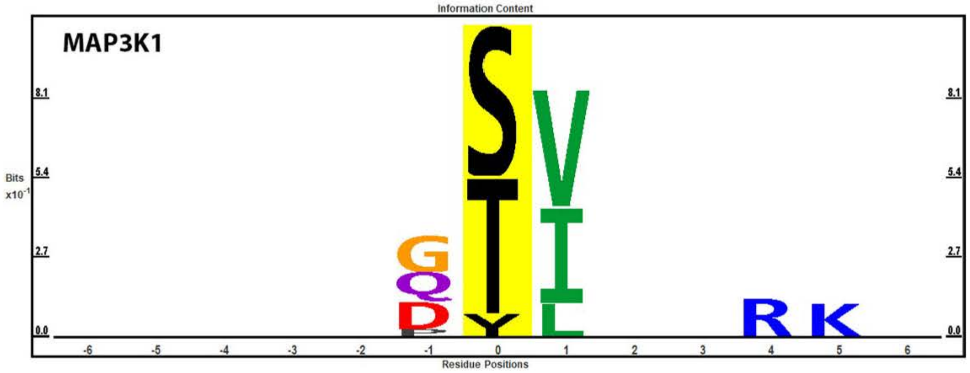
Information Content
MAP3K1
Bits
x10-1
8.1
5.4
2.7
0.0
-6 -5 -4 -3 -2 -1 0 1 2 3 4 5 6
Residue Positions

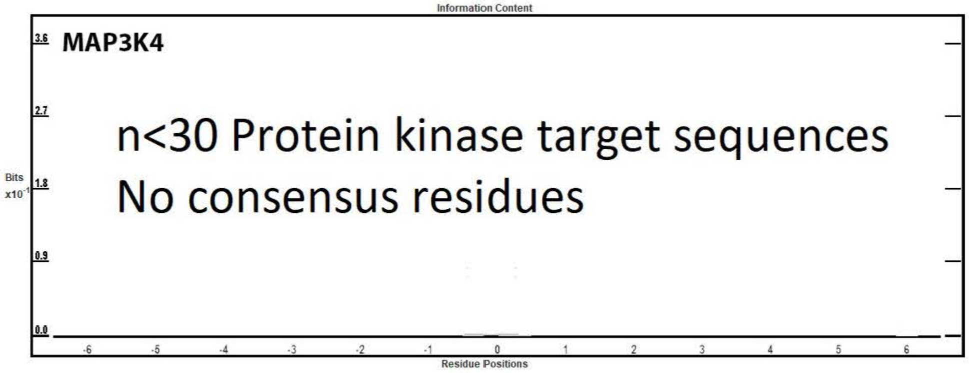
Information Content
MAP3K4
n<30 Protein kinase target sequences
No consensus residues
Bits
x10⁻¹
Residue Positions

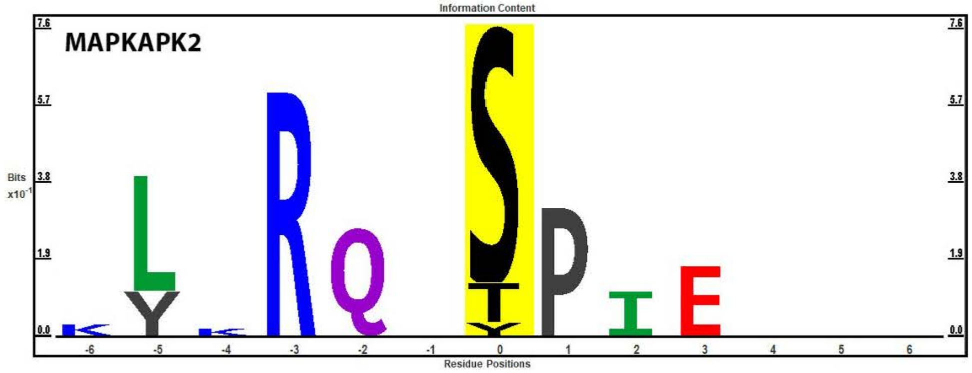
Information Content
MAPKAPK2
Bits
x10⁻¹
Residue Positions

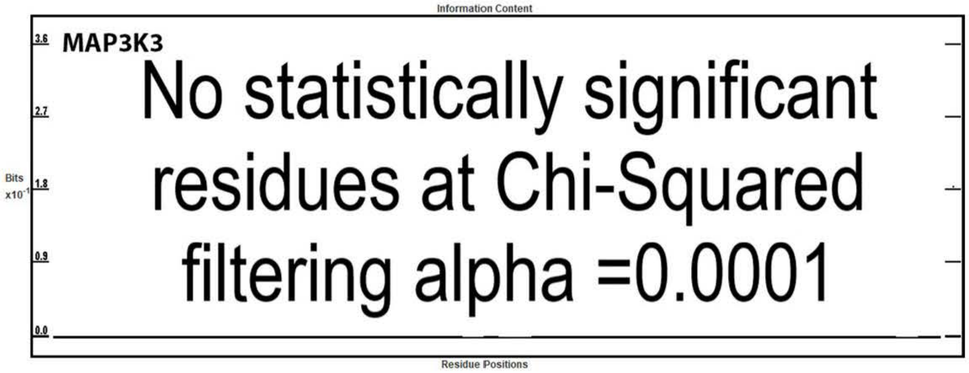
Information Content
MAP3K3
No statistically significant
residues at Chi-Squared
filtering alpha =0.0001
Bits
x10⁻¹
Residue Positions

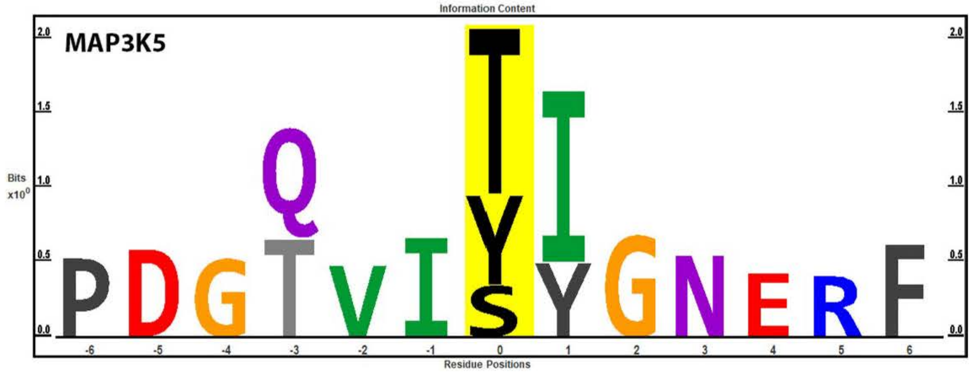
Information Content
MAP3K5
Bits
x10⁰
Residue Positions

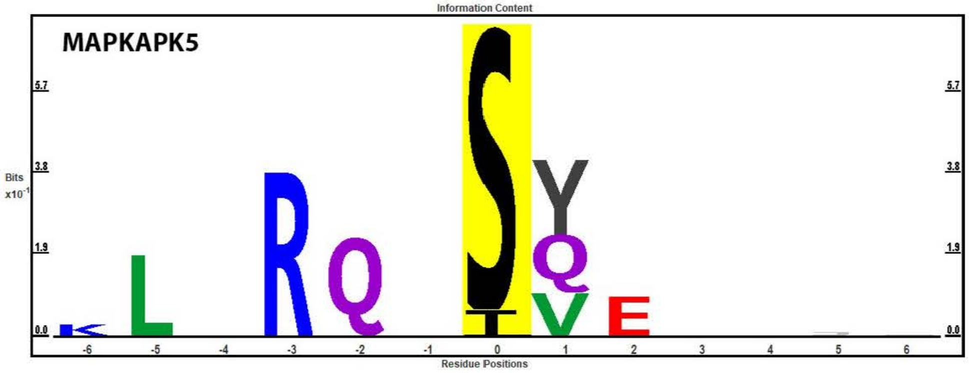
Information Content
MAPKAPK5
Bits
x10-1
Residue Positions

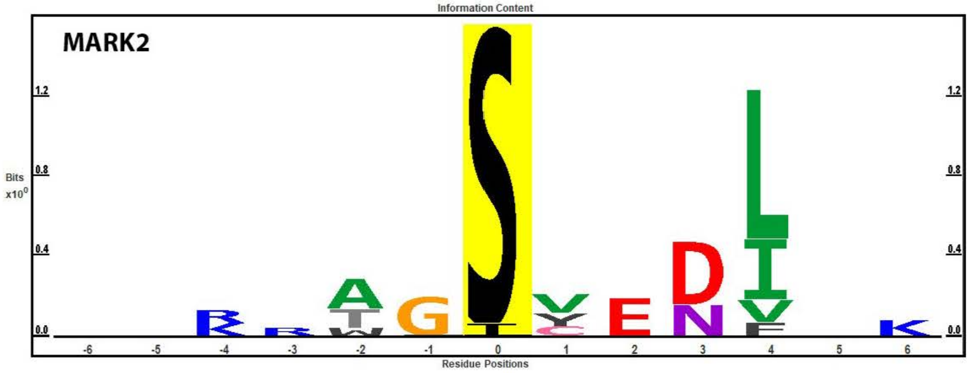
Information Content
MARK2
Bits
x100
Residue Positions

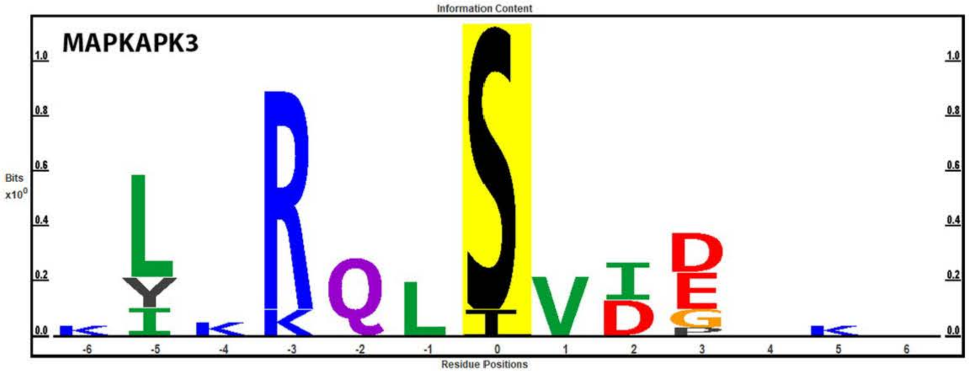
Information Content
MAPKAPK3
Bits
x100
Residue Positions

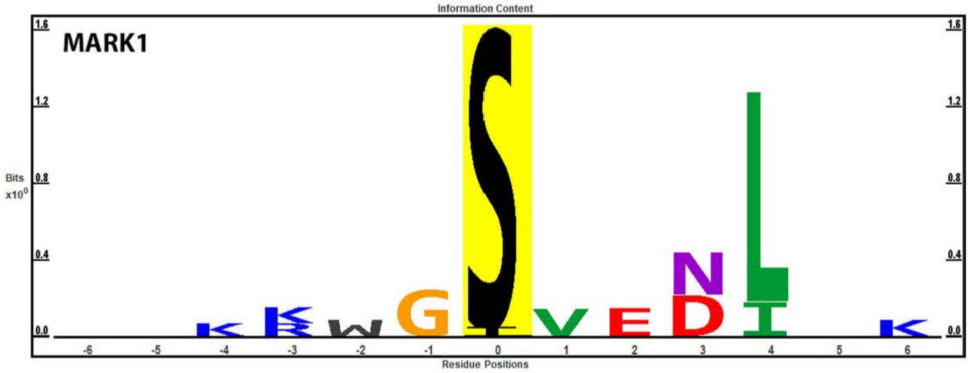
Information Content
MARK1
Bits
x100
Residue Positions

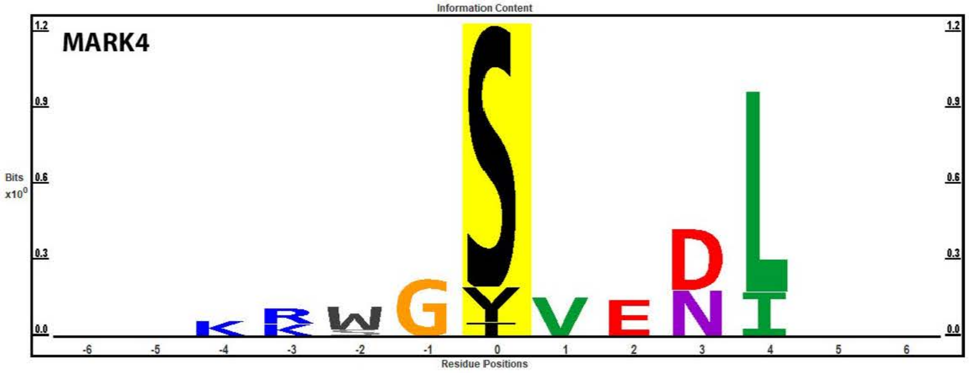
Information Content
MARK4
Bits
x10^0
Residue Positions

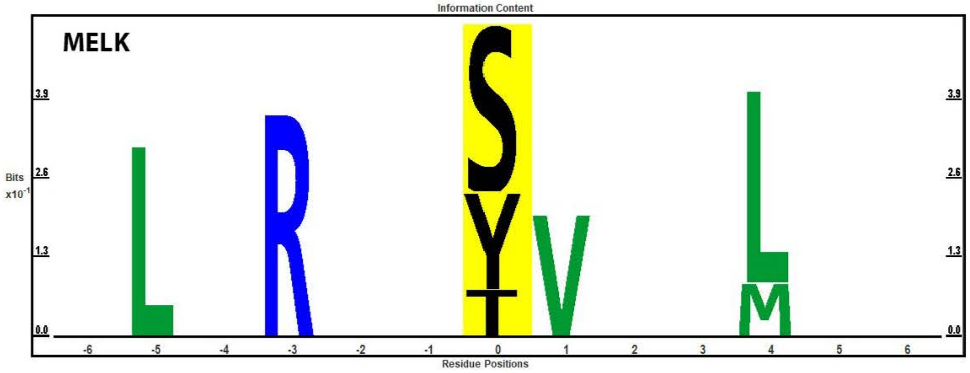
Information Content
MELK
Bits
x10^-1
Residue Positions

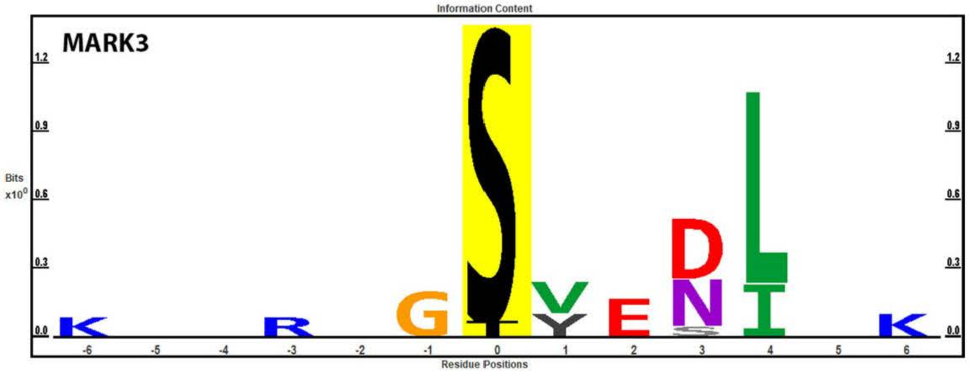
Information Content
MARK3
Bits
x10^0
Residue Positions

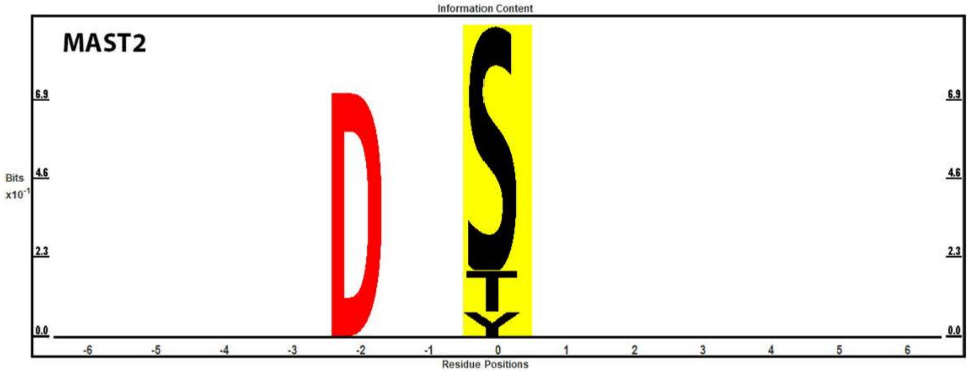
Information Content
MAST2
Bits
x10^-1
Residue Positions

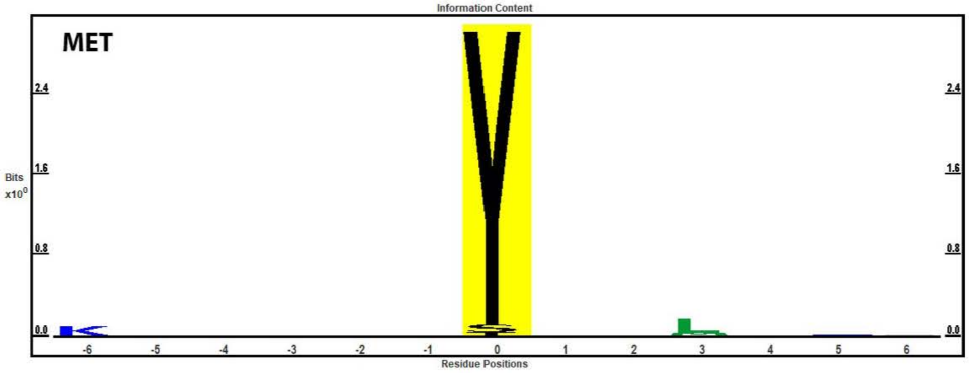
Information Content
MET
Bits
x10^0
2.4
1.6
0.8
0.0
-6 -5 -4 -3 -2 -1 0 1 2 3 4 5 6
Residue Positions

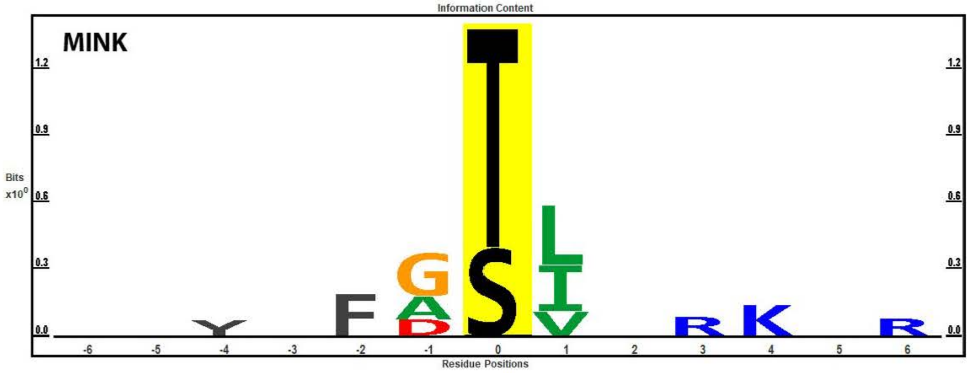
Information Content
MINK
Bits
x10^0
1.2
0.9
0.6
0.3
0.0
-6 -5 -4 -3 -2 -1 0 1 2 3 4 5 6
Residue Positions

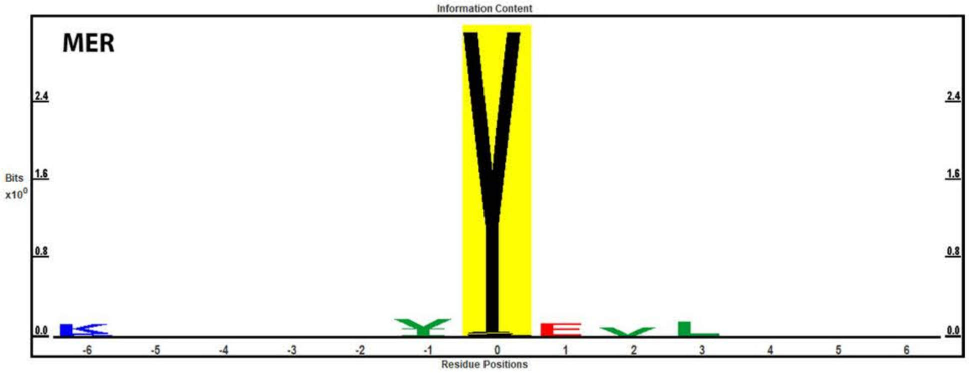
Information Content
MER
Bits
x10^0
2.4
1.6
0.8
0.0
-6 -5 -4 -3 -2 -1 0 1 2 3 4 5 6
Residue Positions

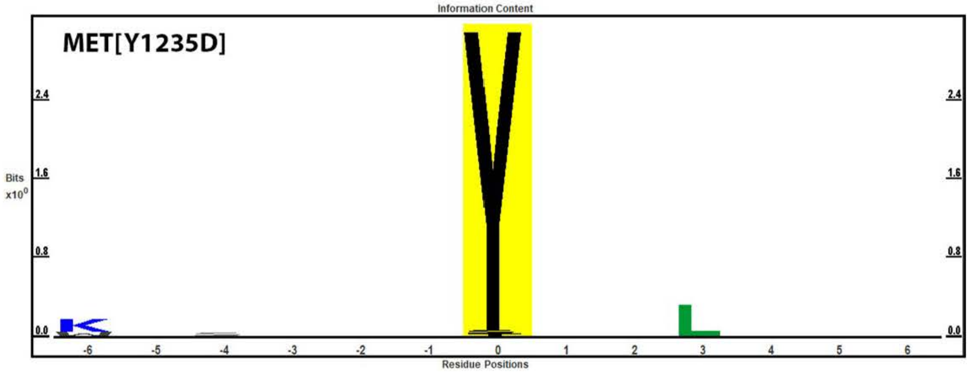
Information Content
MET[Y1235D]
Bits
x10^0
2.4
1.6
0.8
0.0
-6 -5 -4 -3 -2 -1 0 1 2 3 4 5 6
Residue Positions

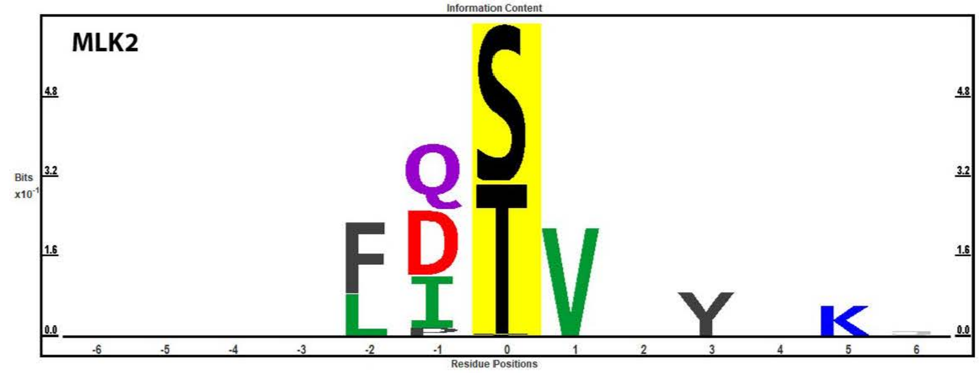
Information Content
MLK2
Bits
x10-1
Residue Positions

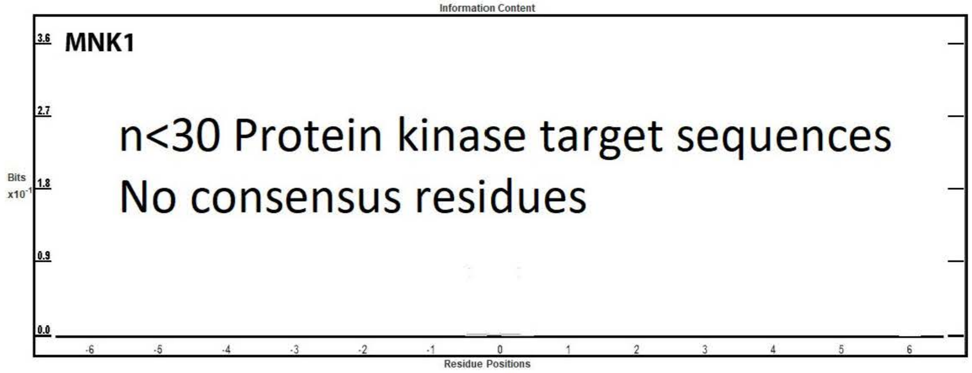
Information Content
MNK1
n<30 Protein kinase target sequences
No consensus residues
Bits
x10-1
Residue Positions

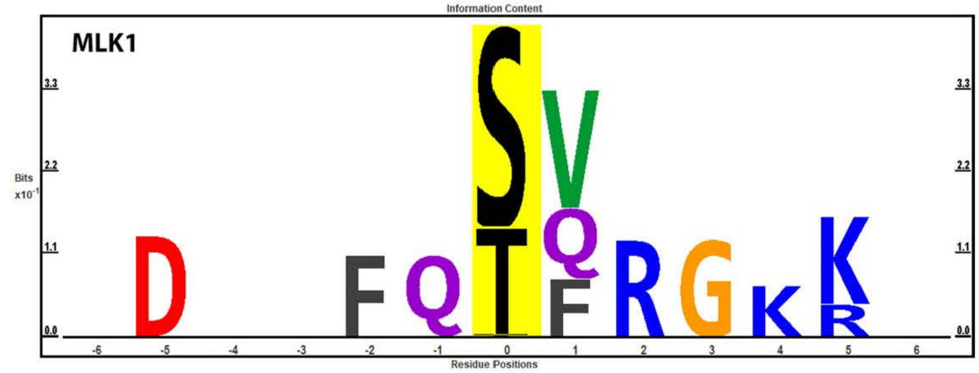
Information Content
MLK1
Bits
x10-1
Residue Positions

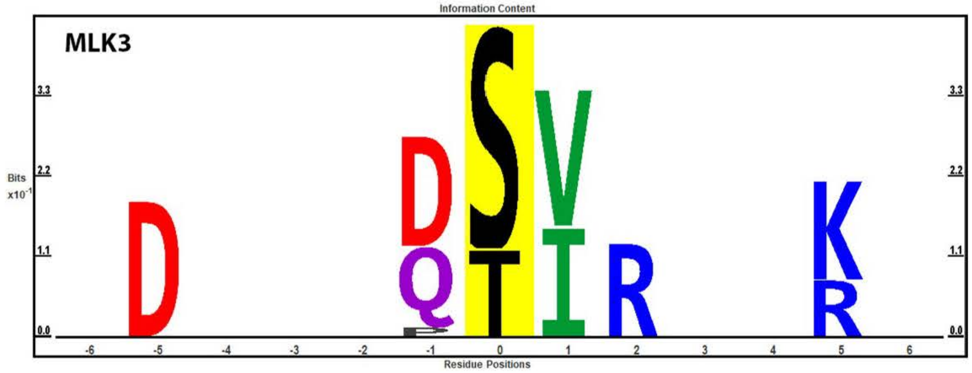
Information Content
MLK3
Bits
x10-1
Residue Positions

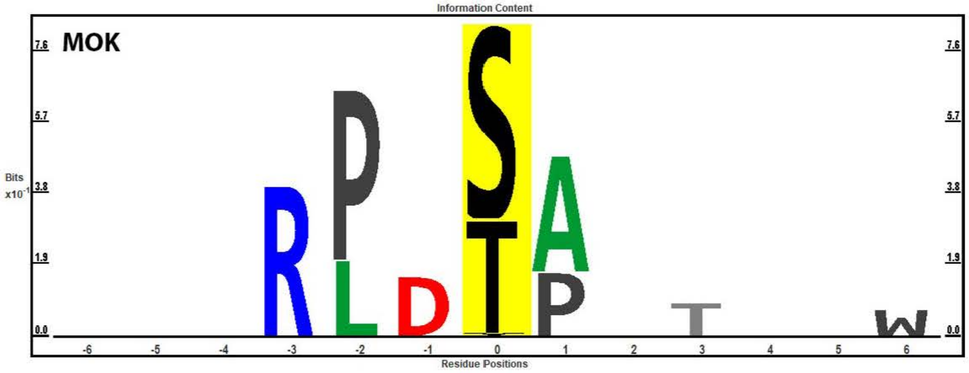
Information Content
MOK
Bits
x10-1
Residue Positions

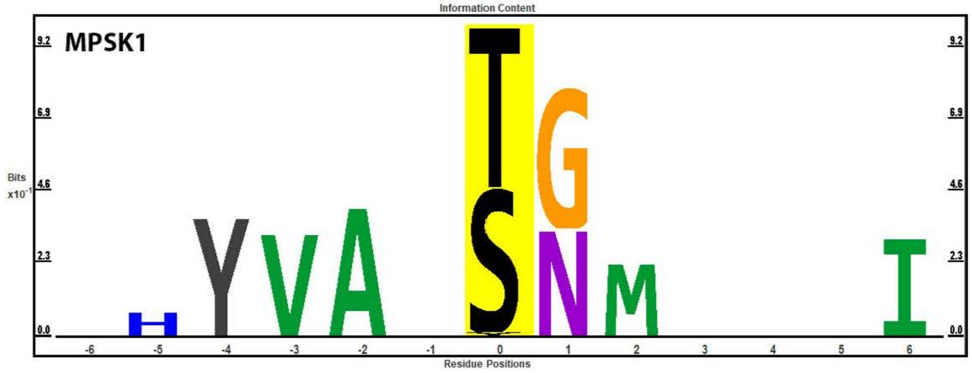
Information Content
MPSK1
Bits
x10-1
Residue Positions

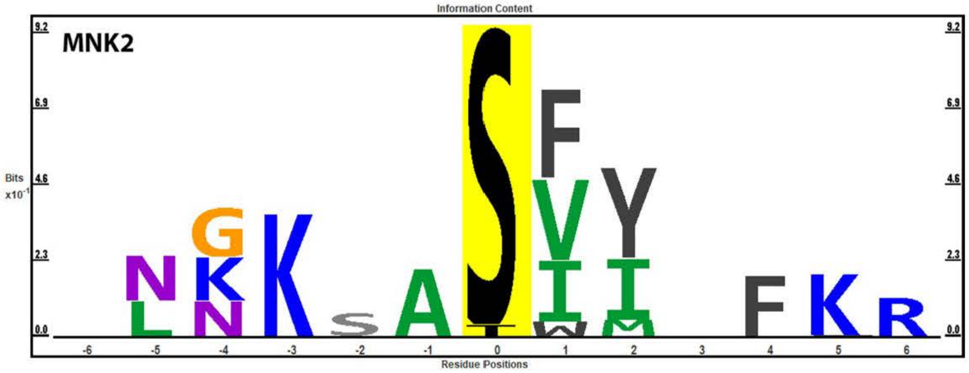
Information Content
MNK2
Bits
x10-1
Residue Positions

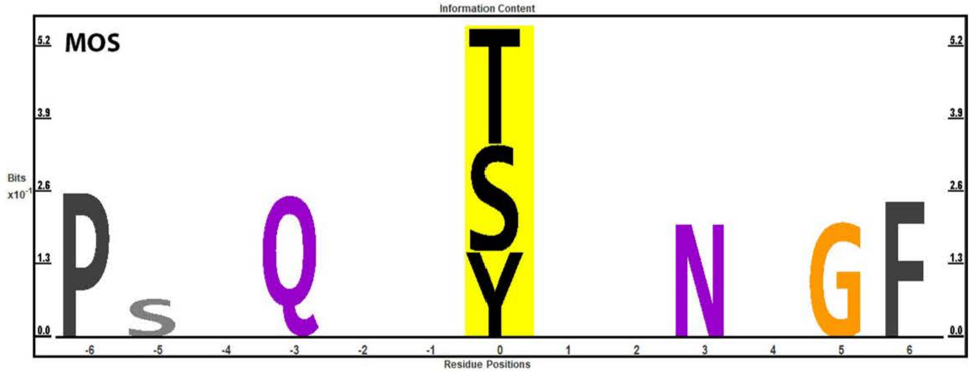
Information Content
MOS
Bits
x10-1
Residue Positions

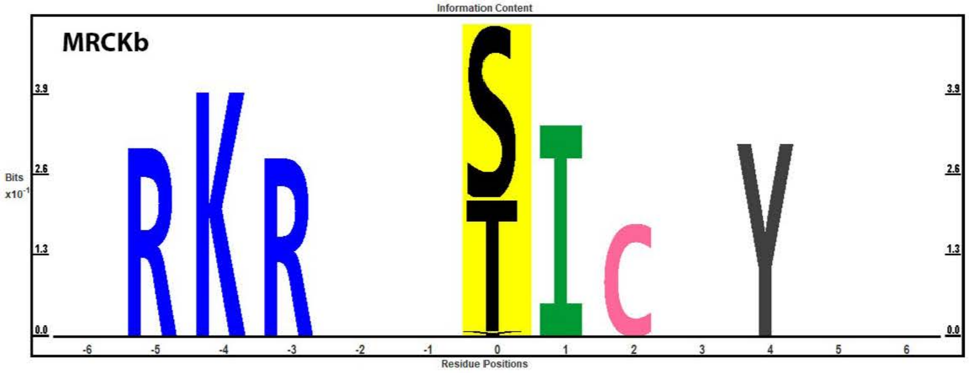
Information Content
MRCKb
Bits
x10-1
Residue Positions

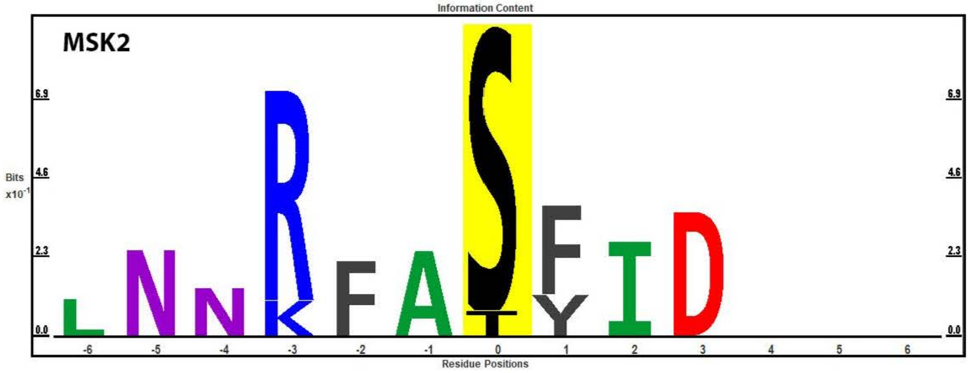
Information Content
MSK2
Bits
x10-1
Residue Positions

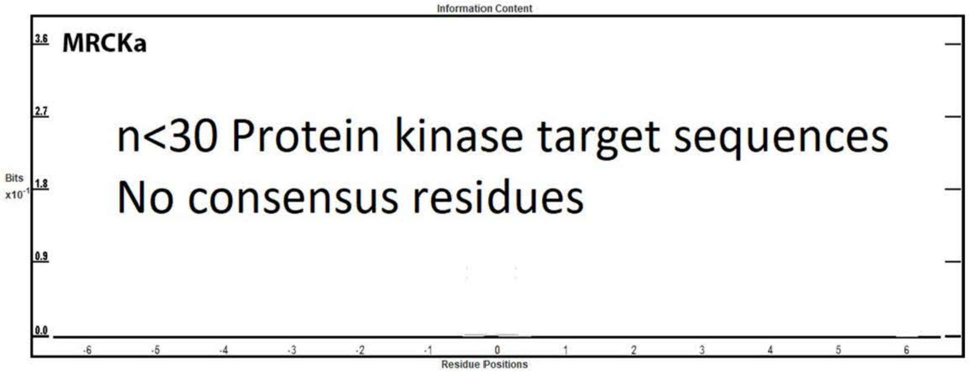
Information Content
MRCKa
n<30 Protein kinase target sequences
No consensus residues
Bits
x10-1
Residue Positions

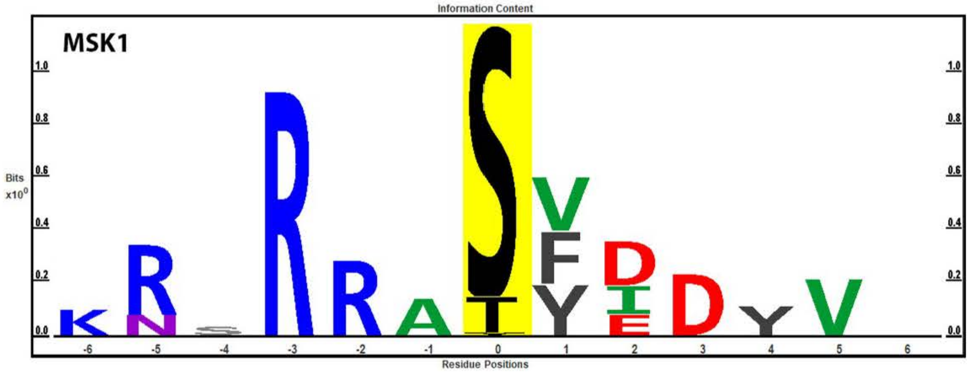
Information Content
MSK1
Bits
x10^0
Residue Positions

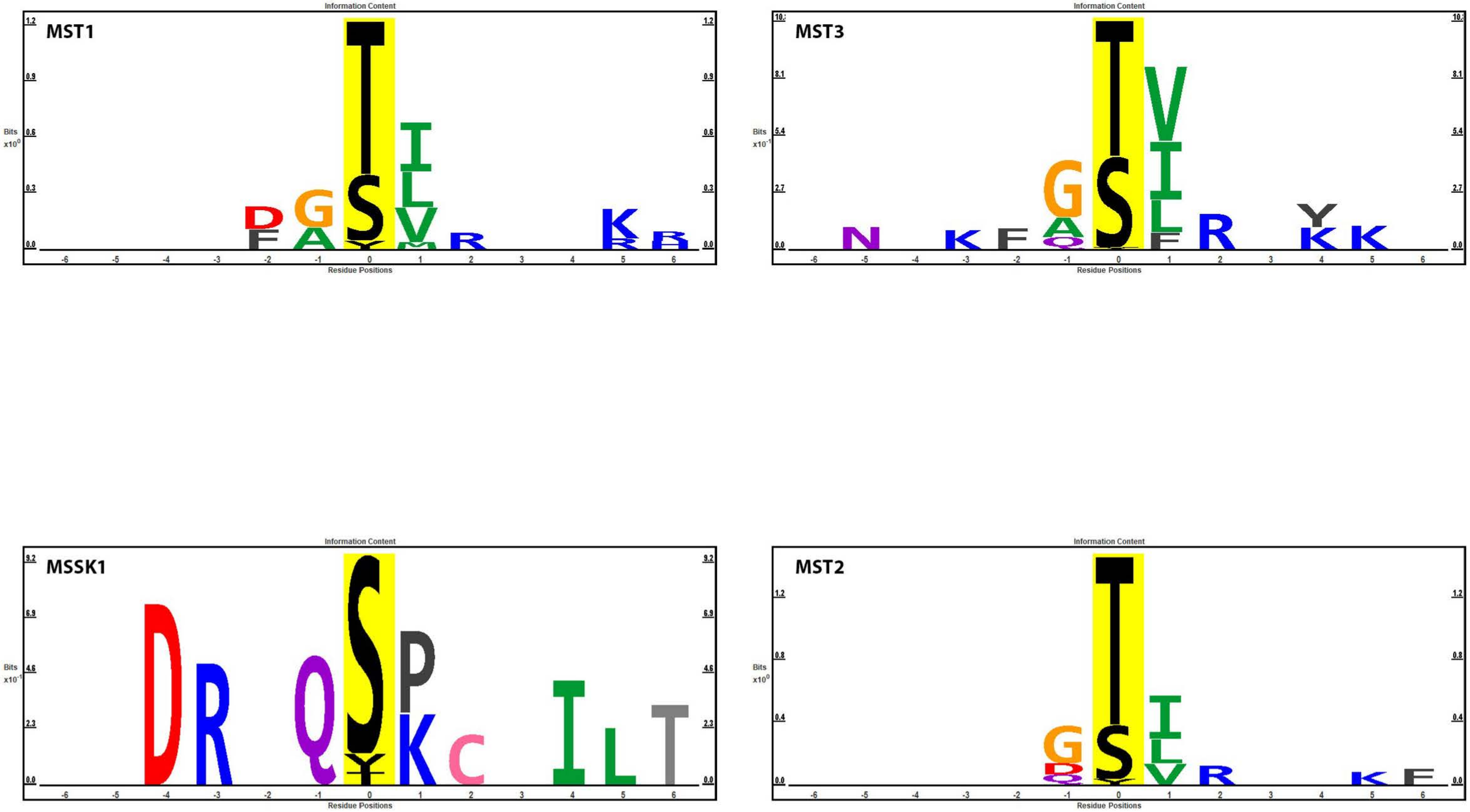
MST1
Information Content
Bits x10^0
Residue Positions
MST3
Information Content
Bits x10^-1
Residue Positions
MSSK1
Information Content
Bits x10^-1
Residue Positions
MST2
Information Content
Bits x10^0
Residue Positions

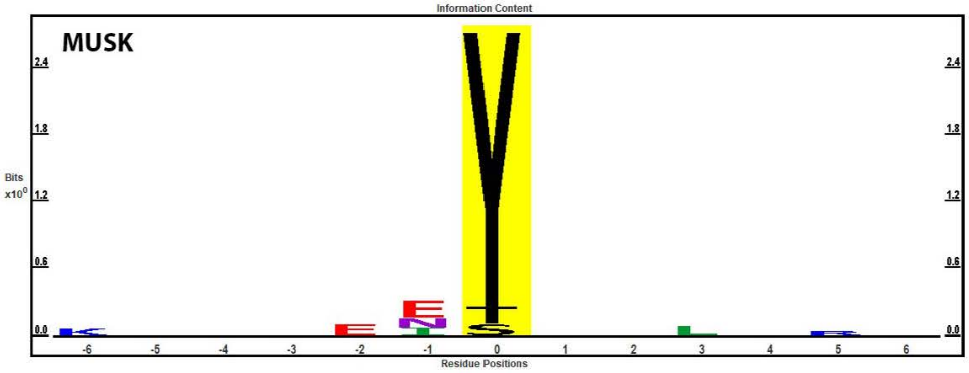
MUSK
Information Content
Bits
x10^0
Residue Positions

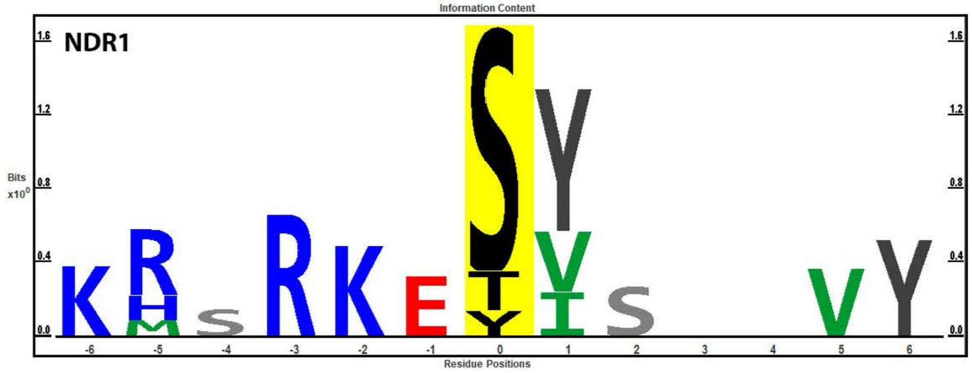
NDR1
Information Content
Bits
x10^0
Residue Positions

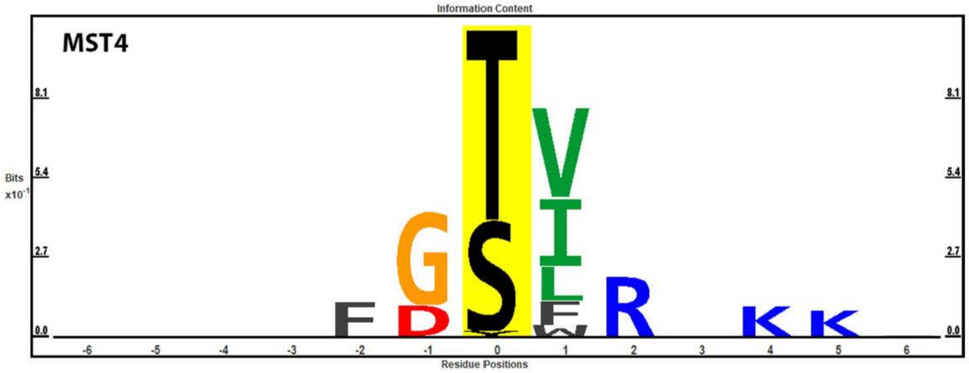
MST4
Information Content
Bits
x10^-1
Residue Positions

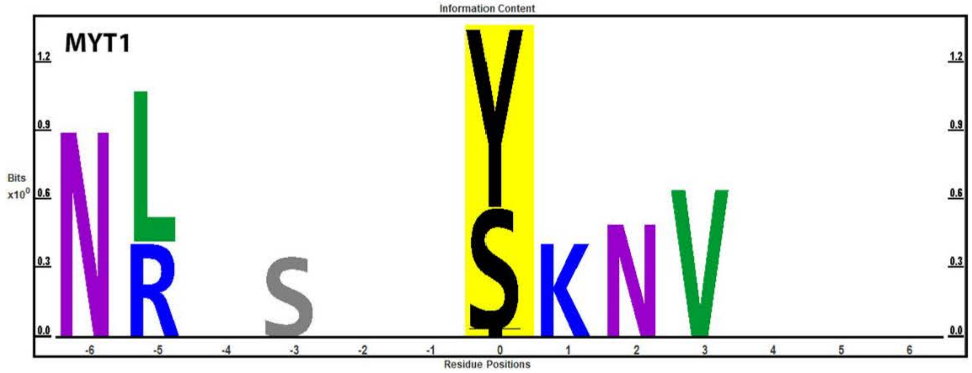
MYT1
Information Content
Bits
x10^0
Residue Positions

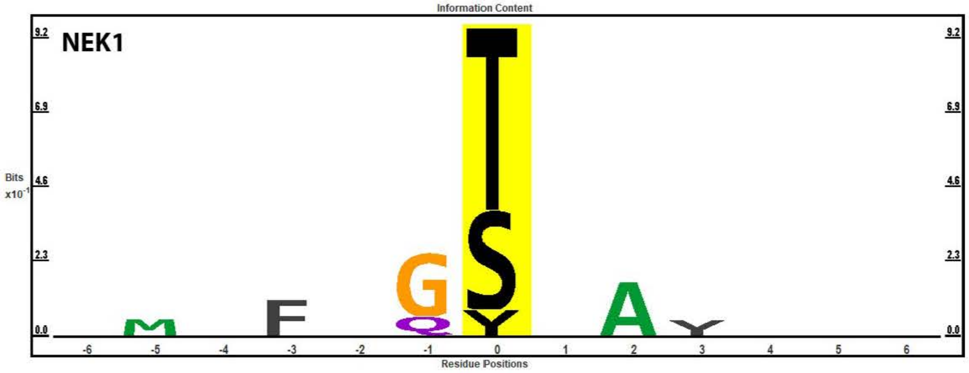
Information Content
NEK1
Bits
x10-1
9.2
6.9
4.6
2.3
0.0
-6 -5 -4 -3 -2 -1 0 1 2 3 4 5 6
Residue Positions

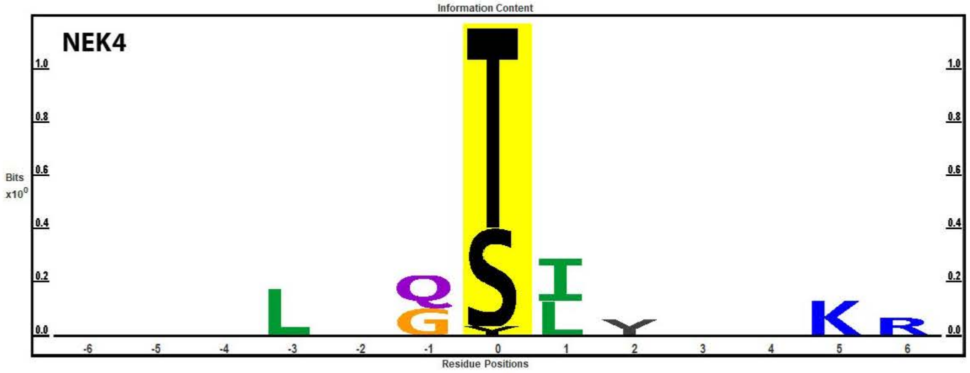
Information Content
NEK4
Bits
x100
1.0
0.8
0.6
0.4
0.2
0.0
-6 -5 -4 -3 -2 -1 0 1 2 3 4 5 6
Residue Positions

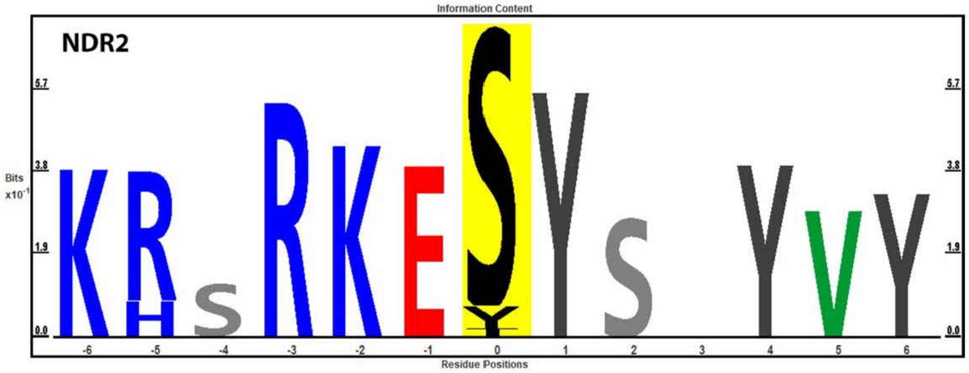
Information Content
NDR2
Bits
x10-1
5.7
3.8
1.9
0.0
-6 -5 -4 -3 -2 -1 0 1 2 3 4 5 6
Residue Positions

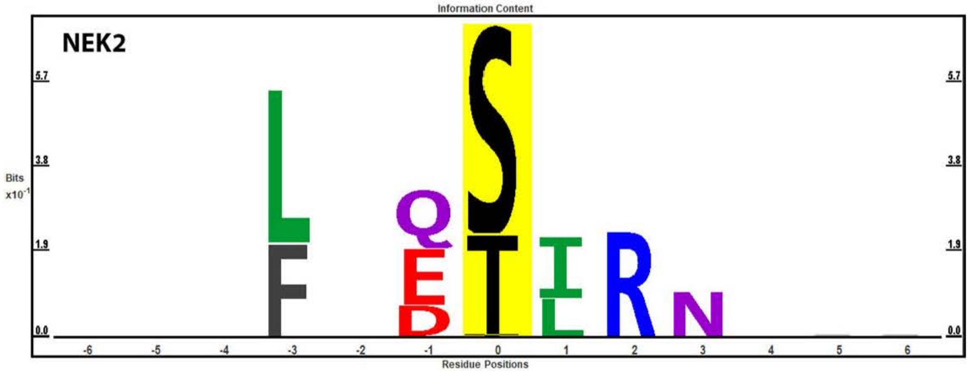
Information Content
NEK2
Bits
x10-1
5.7
3.8
1.9
0.0
-6 -5 -4 -3 -2 -1 0 1 2 3 4 5 6
Residue Positions

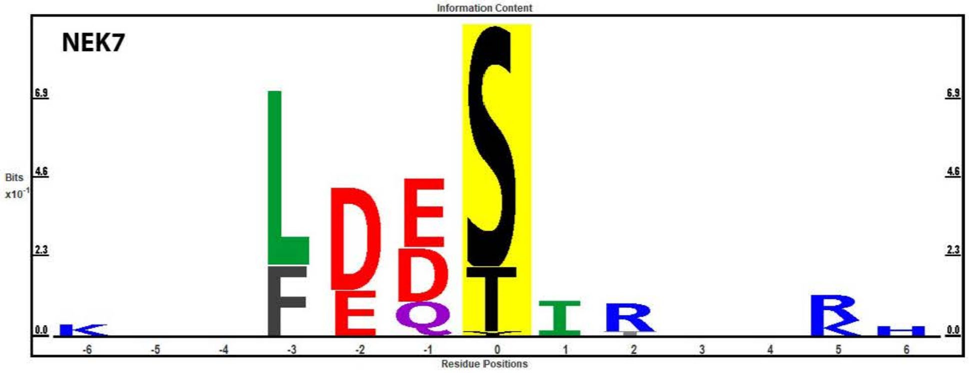
Information Content
NEK7
Bits
x10-1
0.0
2.3
4.6
6.9
-6 -5 -4 -3 -2 -1 0 1 2 3 4 5 6
Residue Positions

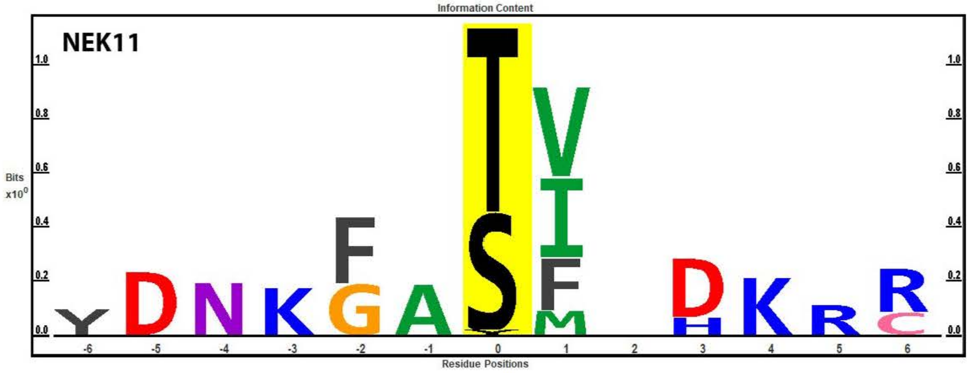
Information Content
NEK11
Bits
x100
0.0
0.2
0.4
0.6
0.8
1.0
-6 -5 -4 -3 -2 -1 0 1 2 3 4 5 6
Residue Positions

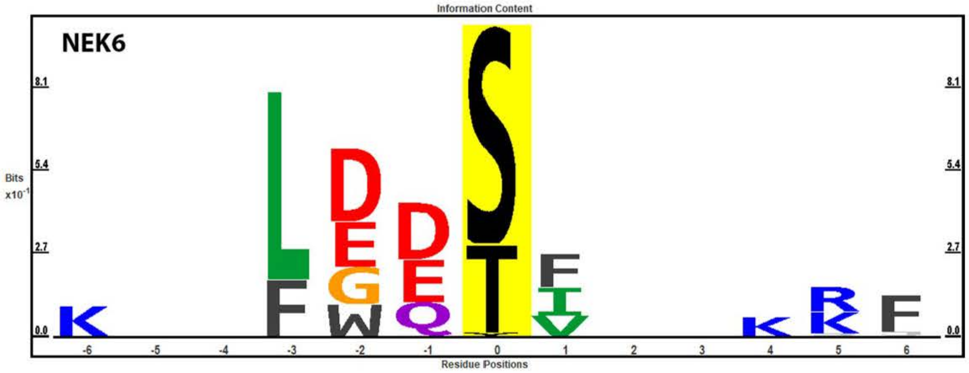
Information Content
NEK6
Bits
x10-1
0.0
2.7
5.4
8.1
-6 -5 -4 -3 -2 -1 0 1 2 3 4 5 6
Residue Positions

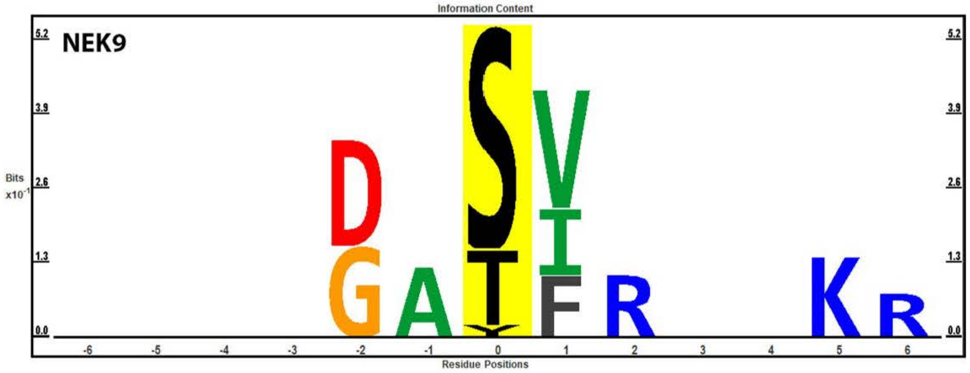
Information Content
NEK9
Bits
x10-1
0.0
1.3
2.6
3.9
5.2
-6 -5 -4 -3 -2 -1 0 1 2 3 4 5 6
Residue Positions

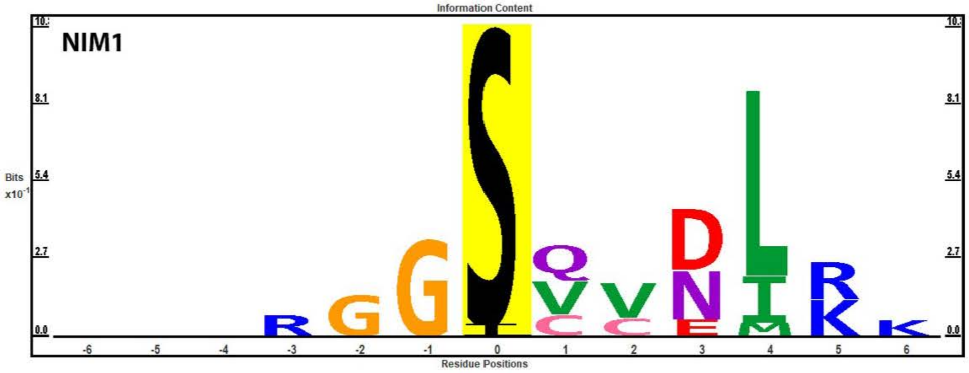
Information Content
NIM1
Bits
x10-1
Residue Positions

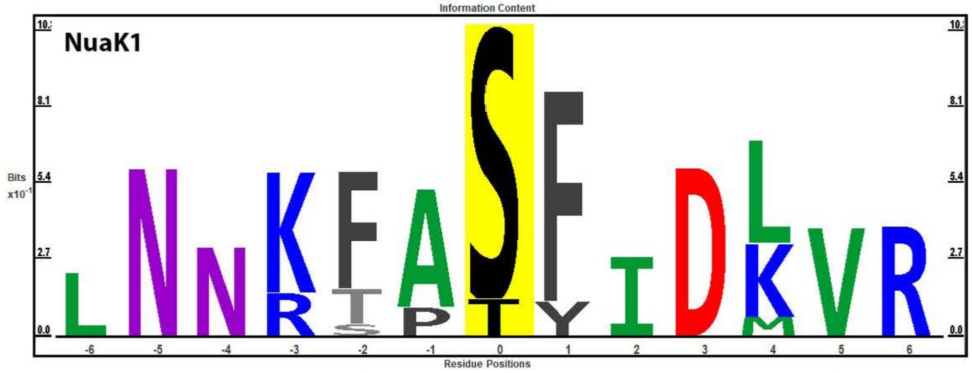
Information Content
NuaK1
Bits
x10-1
Residue Positions

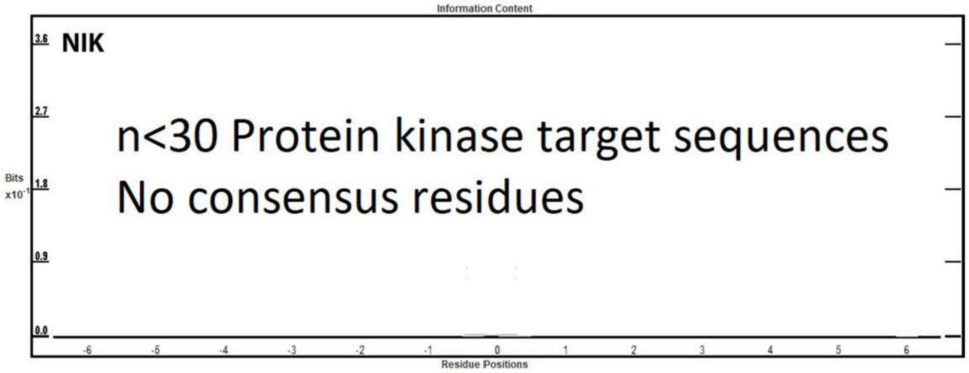
Information Content
NIK
n<30 Protein kinase target sequences
No consensus residues
Bits
x10-1
Residue Positions

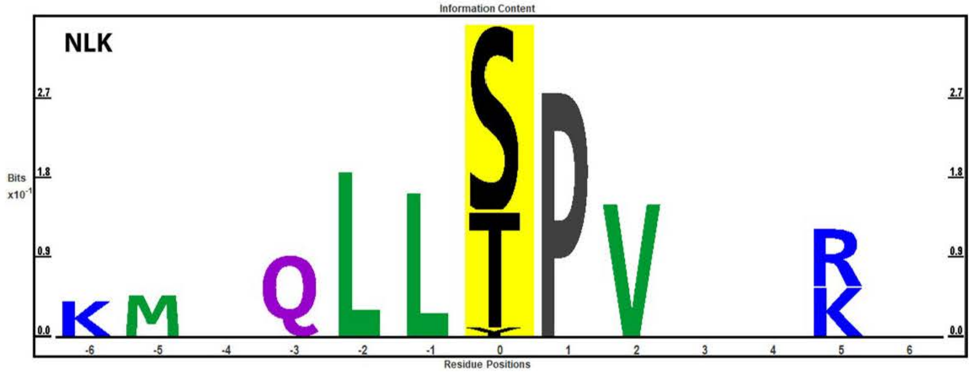
Information Content
NLK
Bits
x10-1
Residue Positions

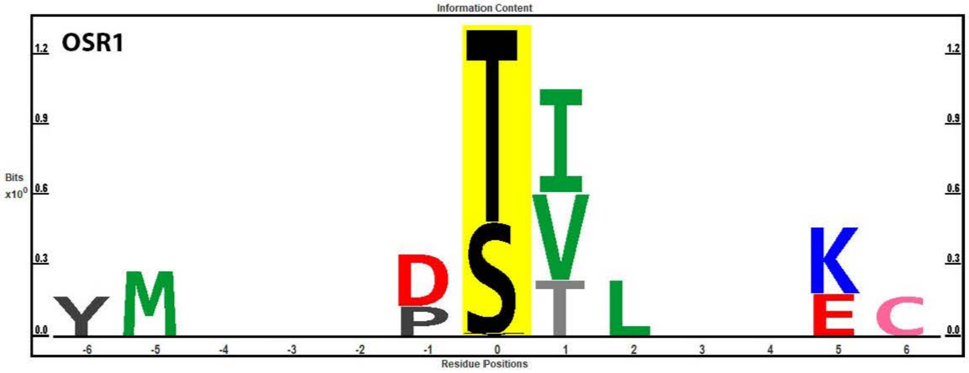
Information Content
OSR1
Bits
x10^0
Residue Positions

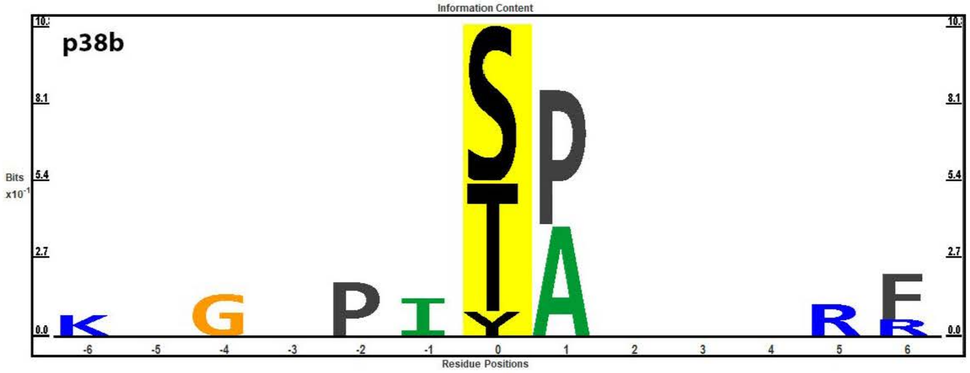
Information Content
p38b
Bits
x10^-1
Residue Positions

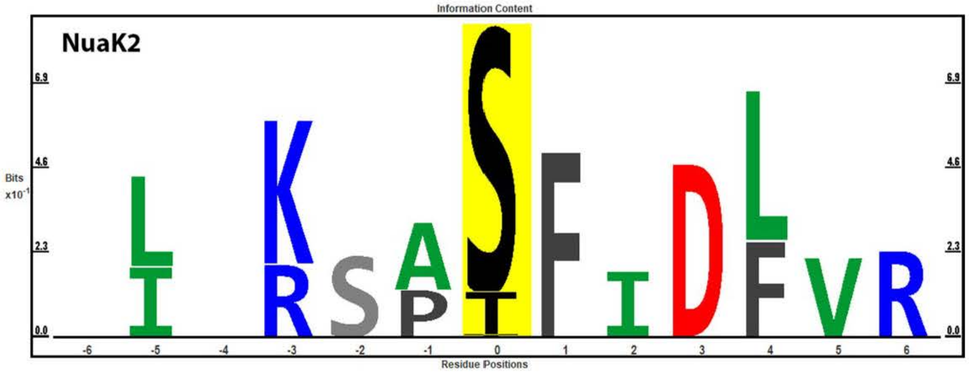
Information Content
NuaK2
Bits
x10^-1
Residue Positions

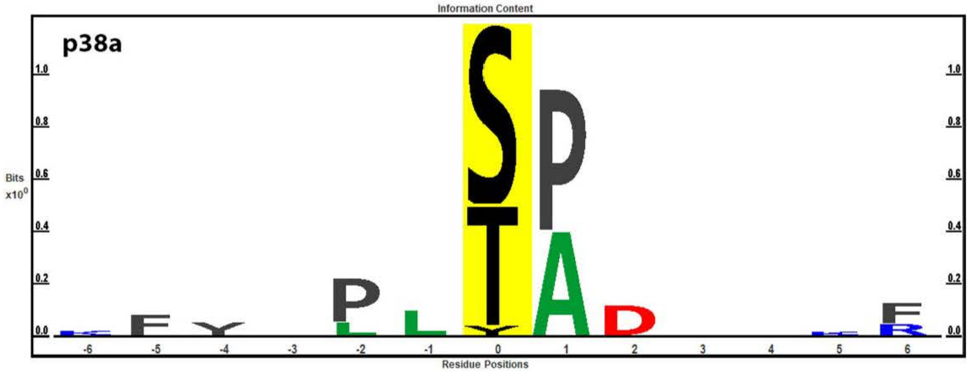
Information Content
p38a
Bits
x10^0
Residue Positions

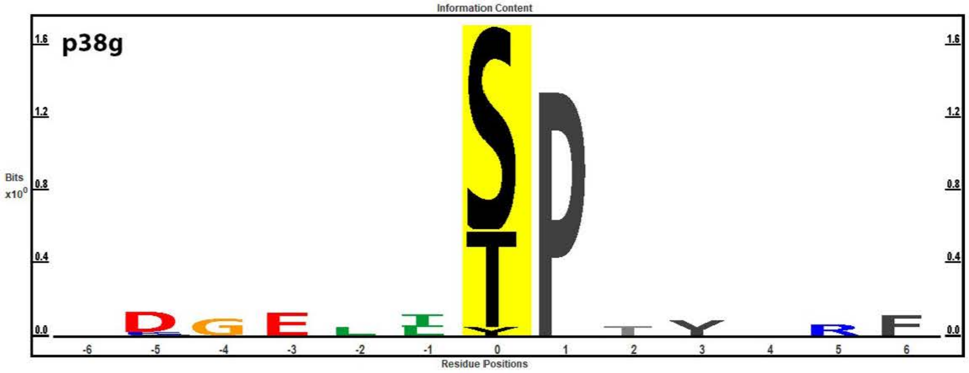
Information Content
p38g
Bits
x10^0
1.6
1.2
0.8
0.4
0.0
-6 -5 -4 -3 -2 -1 0 1 2 3 4 5 6
Residue Positions

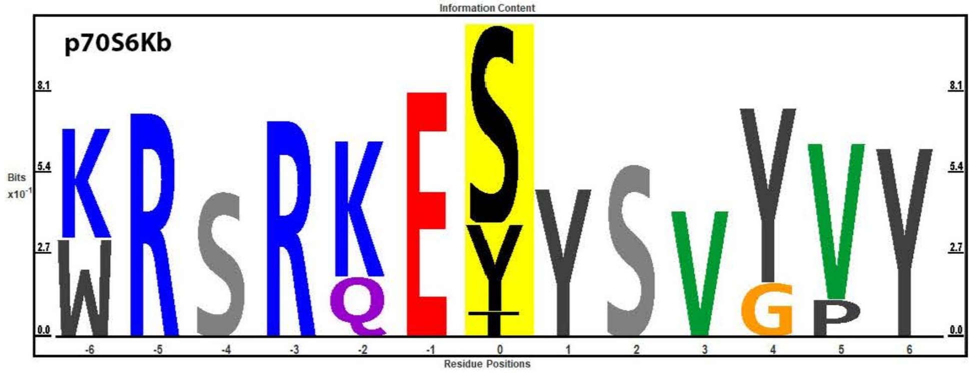
Information Content
p70S6Kb
Bits
x10^-1
8.1
5.4
2.7
0.0
-6 -5 -4 -3 -2 -1 0 1 2 3 4 5 6
Residue Positions

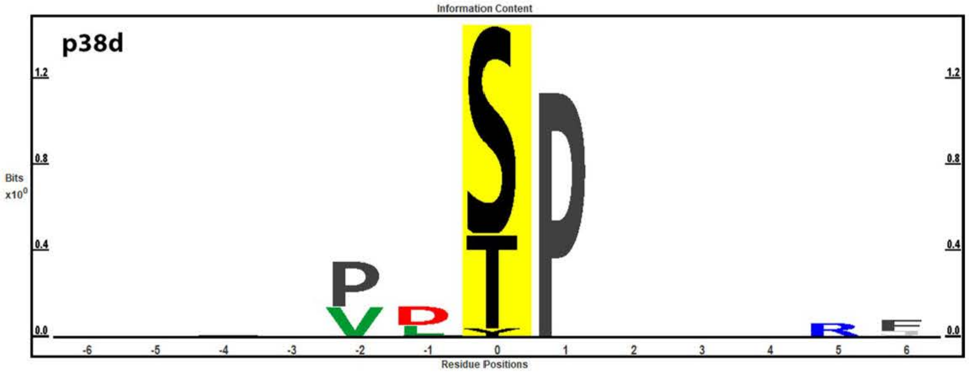
Information Content
p38d
Bits
x10^0
1.2
0.8
0.4
0.0
-6 -5 -4 -3 -2 -1 0 1 2 3 4 5 6
Residue Positions

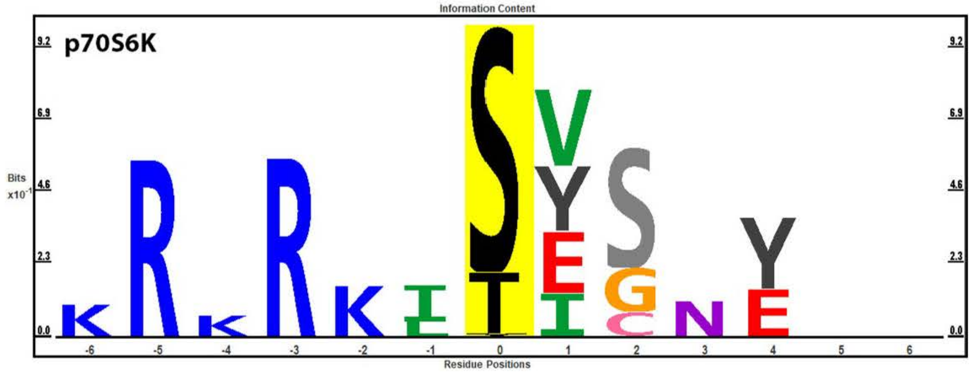
Information Content
p70S6K
Bits
x10^-1
9.2
6.9
4.6
2.3
0.0
-6 -5 -4 -3 -2 -1 0 1 2 3 4 5 6
Residue Positions

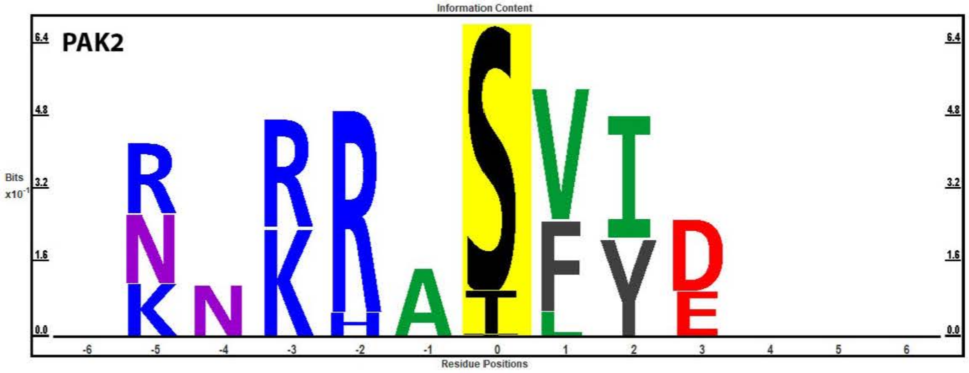
Information Content
PAK2
Bits
Residue Positions

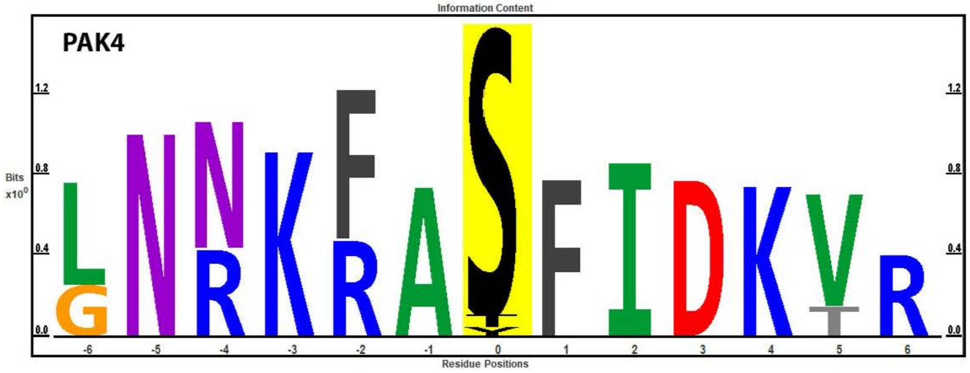
Information Content
PAK4
Bits
Residue Positions

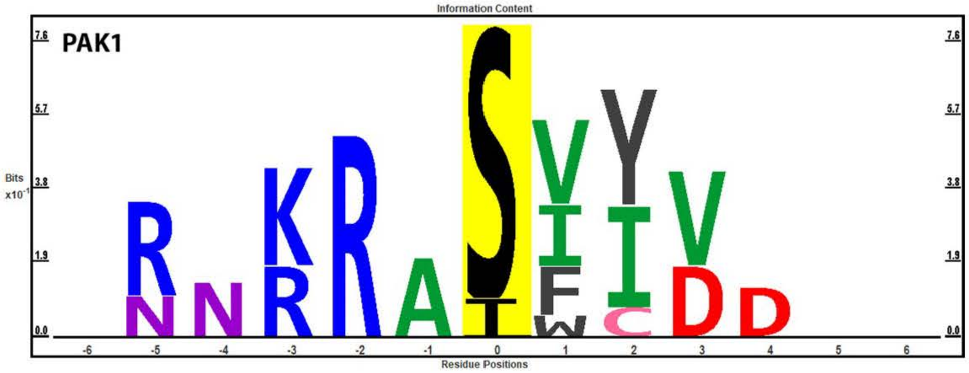
Information Content
PAK1
Bits
Residue Positions

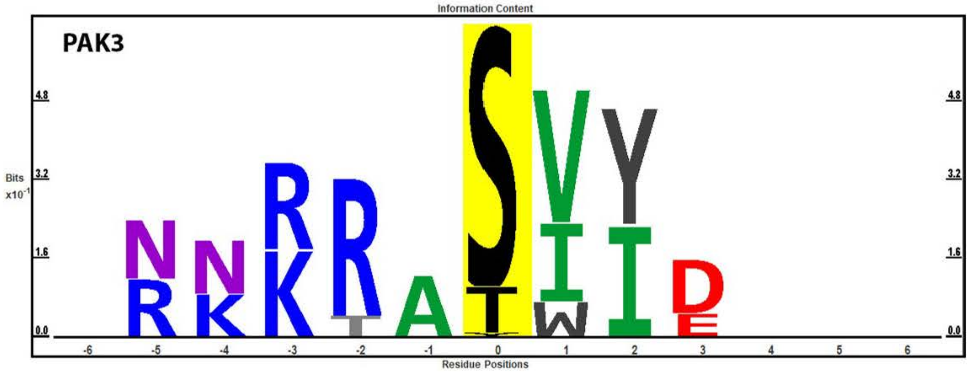
Information Content
PAK3
Bits
Residue Positions

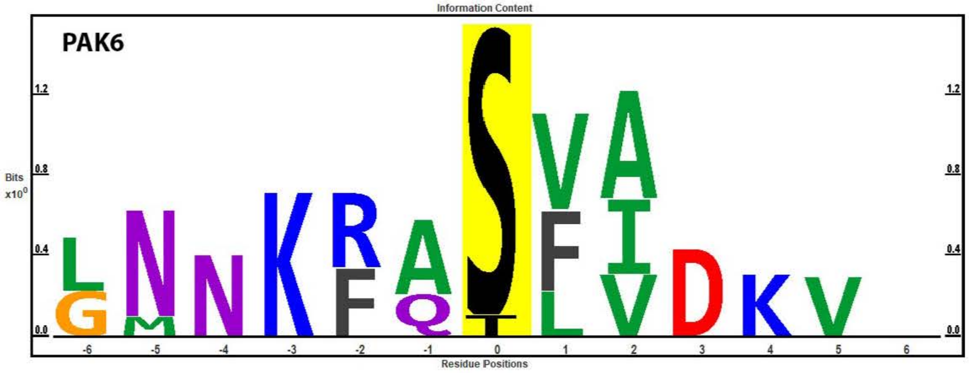
Information Content
PAK6
Bits
x10^0
Residue Positions

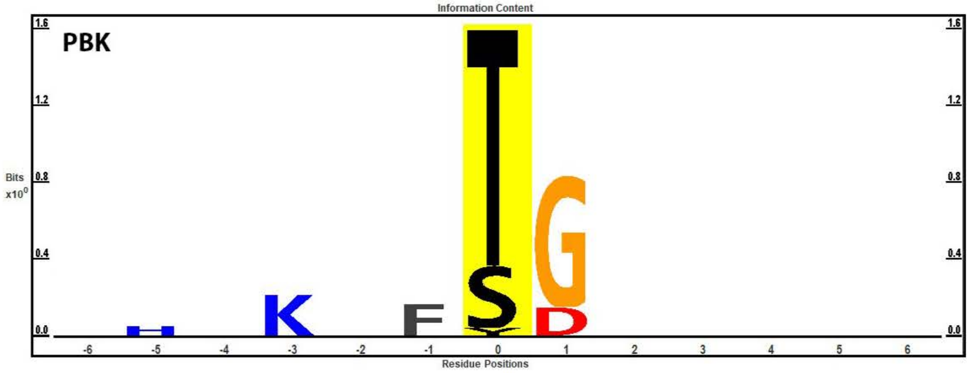
Information Content
PBK
Bits
x10^0
Residue Positions

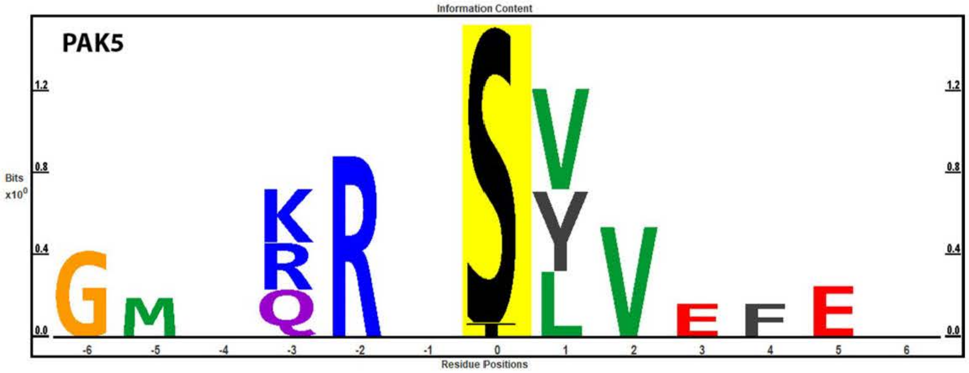
Information Content
PAK5
Bits
x10^0
Residue Positions

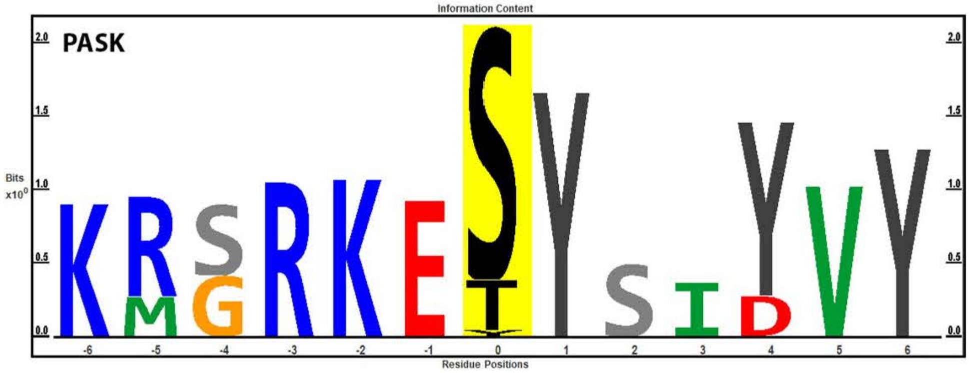
Information Content
PASK
Bits
x10^0
Residue Positions

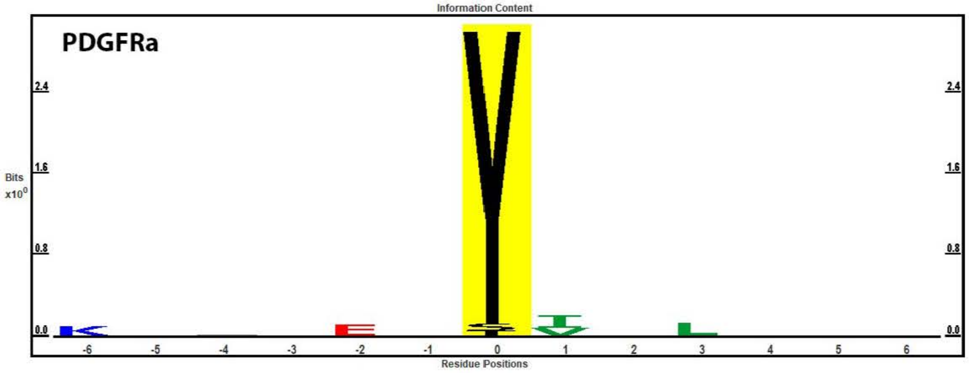
Information Content
PDGFRa
Bits
x10⁰
Residue Positions

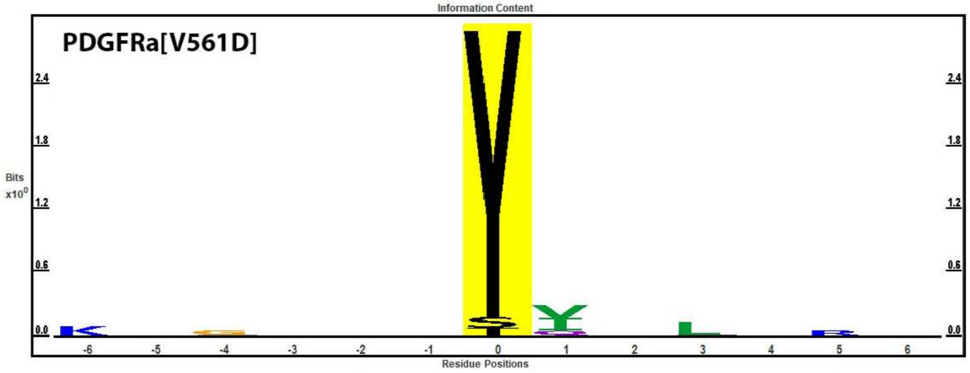
Information Content
PDGFRa[V561D]
Bits
x10⁰
Residue Positions

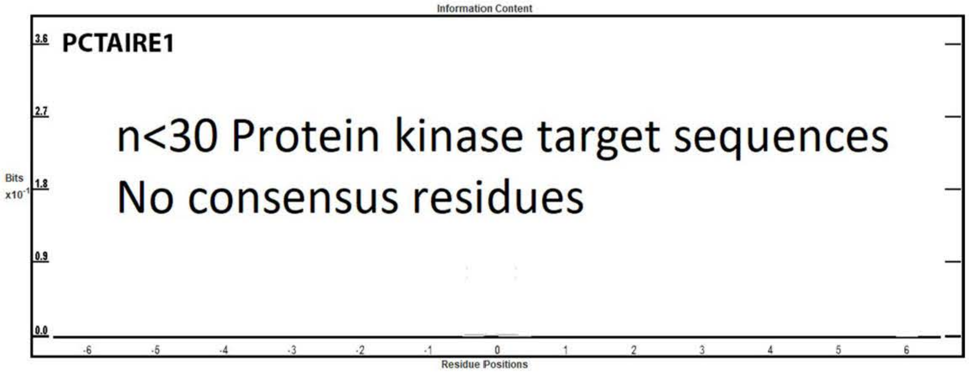
Information Content
PCTAIRE1
n<30 Protein kinase target sequences
No consensus residues
Bits
Residue Positions

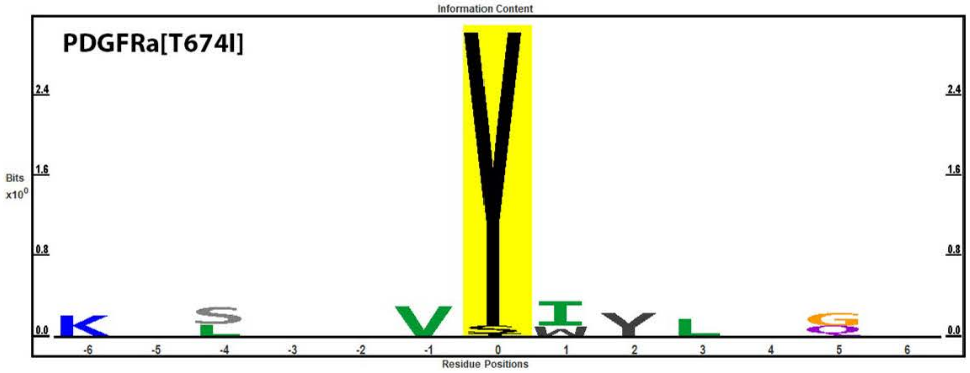
Information Content
PDGFRa[T674I]
Bits
x10⁰
Residue Positions

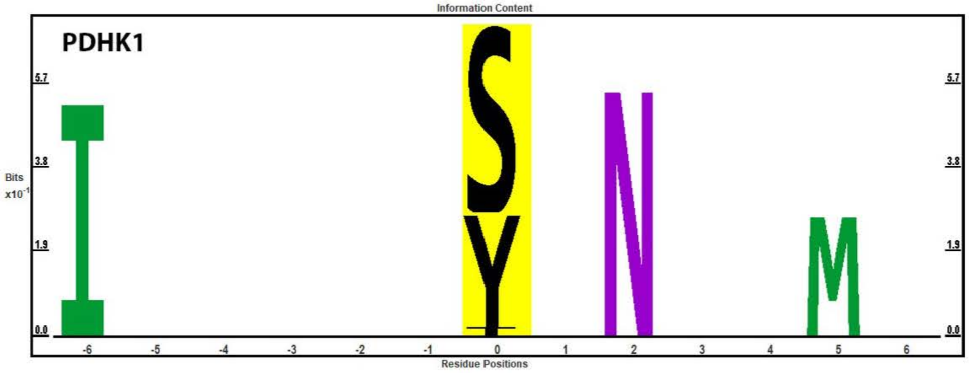
Information Content
PDHK1
Bits
x10-1
Residue Positions

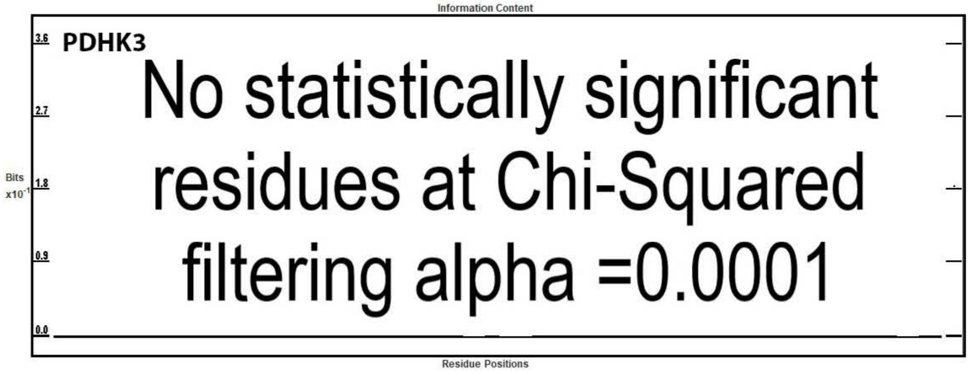
Information Content
PDHK3
No statistically significant residues at Chi-Squared filtering alpha =0.0001
Bits
x10-1
Residue Positions

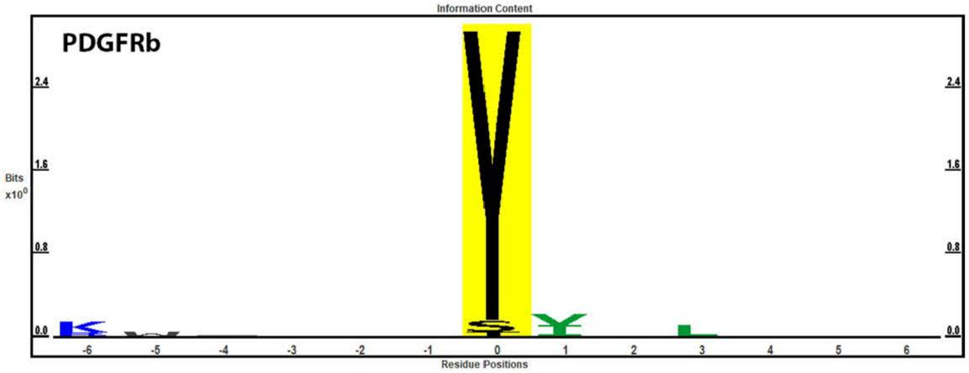
Information Content
PDGFRb
Bits
x10^0
Residue Positions

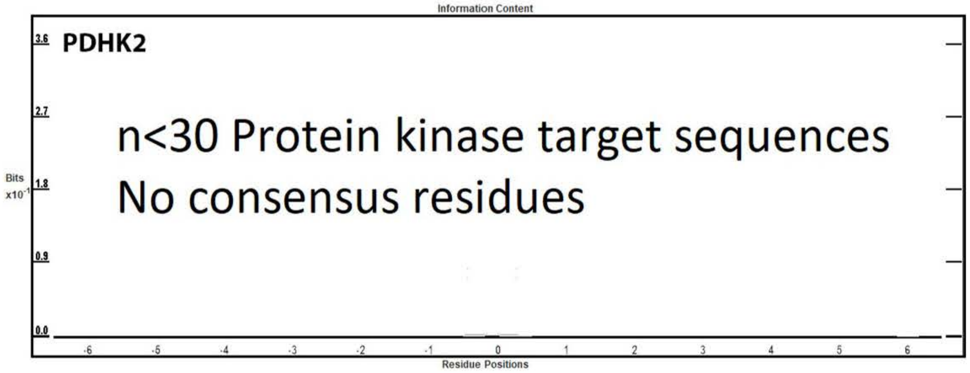
Information Content
PDHK2
n<30 Protein kinase target sequences
No consensus residues
Bits
x10-1
Residue Positions

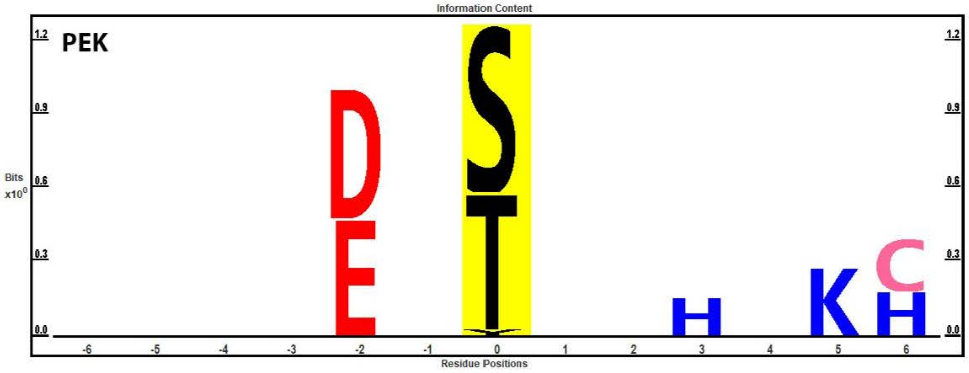
Information Content
PEK
Bits
Residue Positions

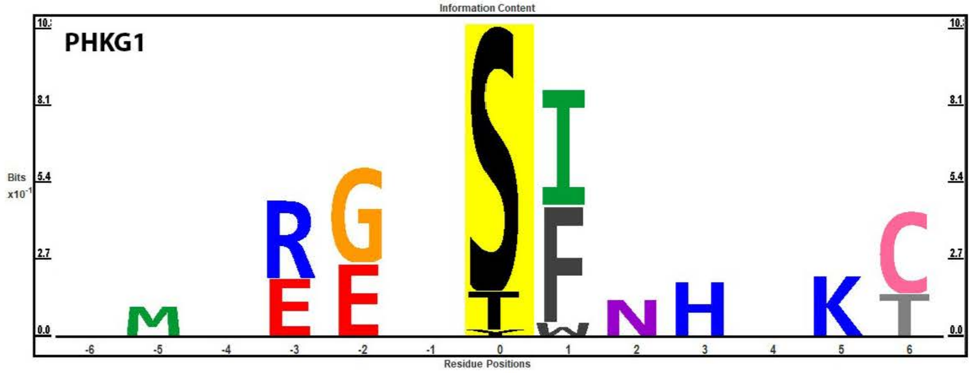
Information Content
PHKG1
Bits
Residue Positions

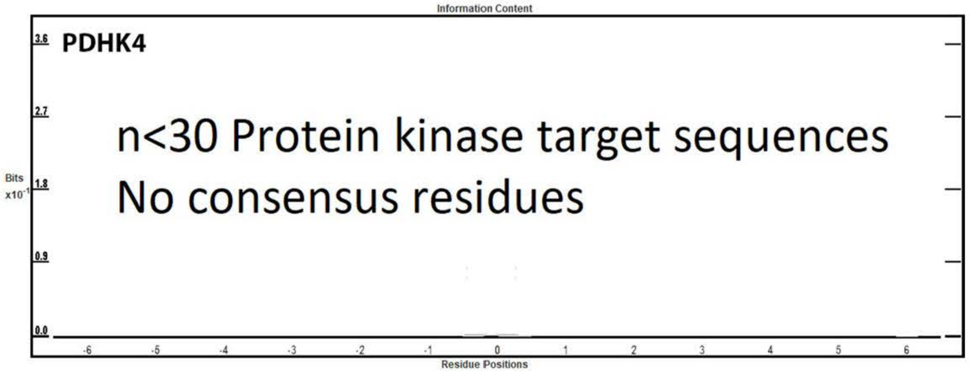
Information Content
PDHK4
n<30 Protein kinase target sequences
No consensus residues
Bits
Residue Positions

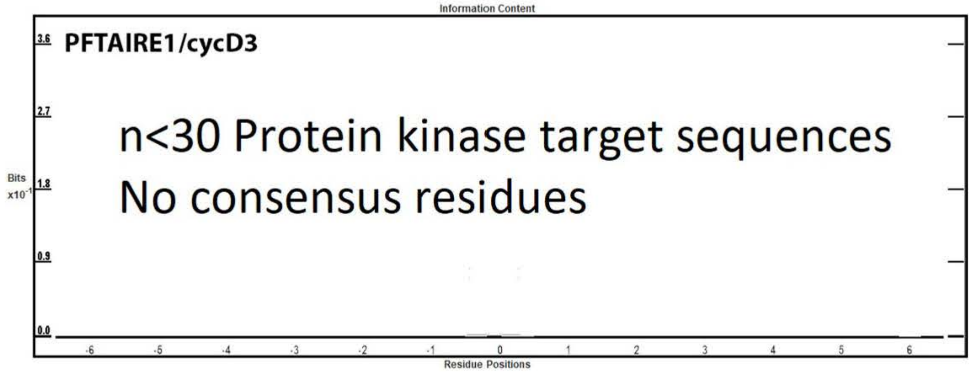
Information Content
PFTAIRE1/cycD3
n<30 Protein kinase target sequences
No consensus residues
Bits
Residue Positions

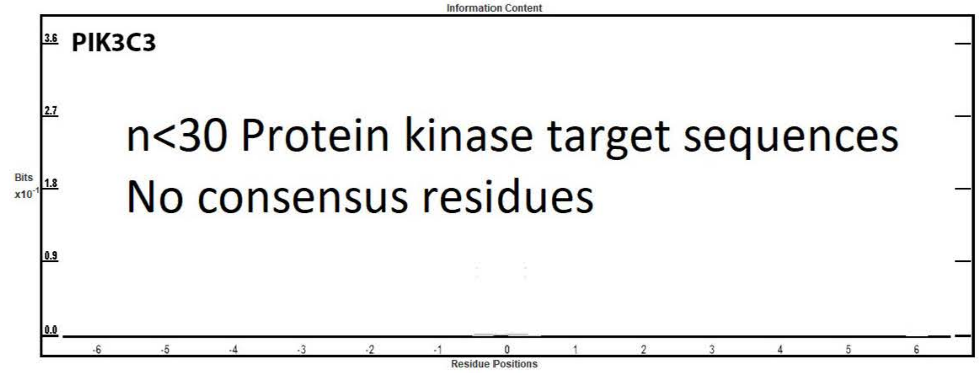
Information Content
PIK3C3
n<30 Protein kinase target sequences
No consensus residues
Bits x10⁻¹
Residue Positions

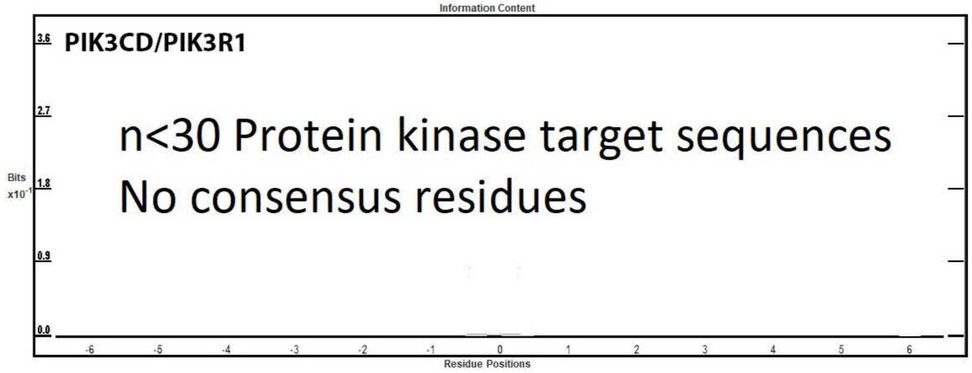
Information Content
PIK3CD/PIK3R1
n<30 Protein kinase target sequences
No consensus residues
Bits x10⁻¹
Residue Positions

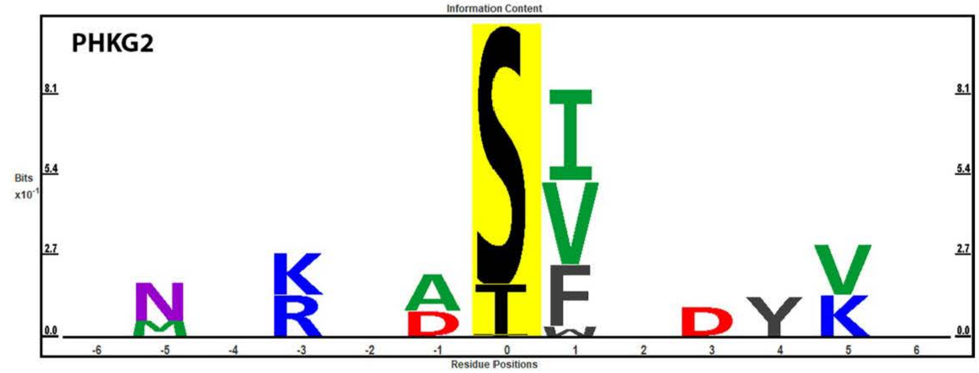
Information Content
PHKG2
Bits x10⁻¹
Residue Positions

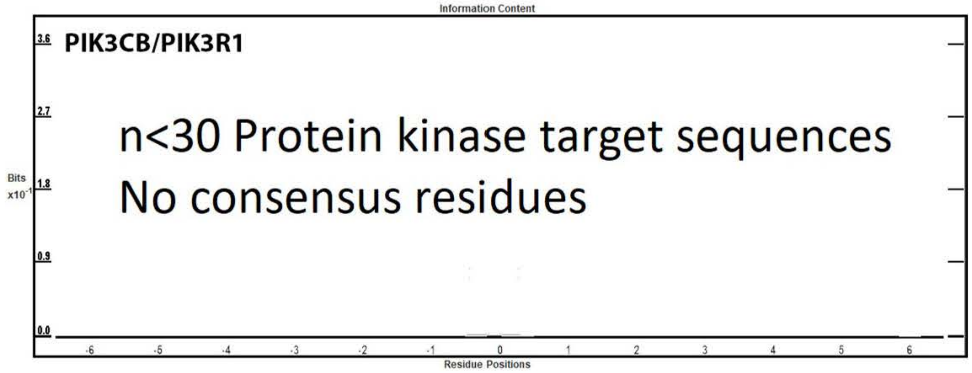
Information Content
PIK3CB/PIK3R1
n<30 Protein kinase target sequences
No consensus residues
Bits x10⁻¹
Residue Positions

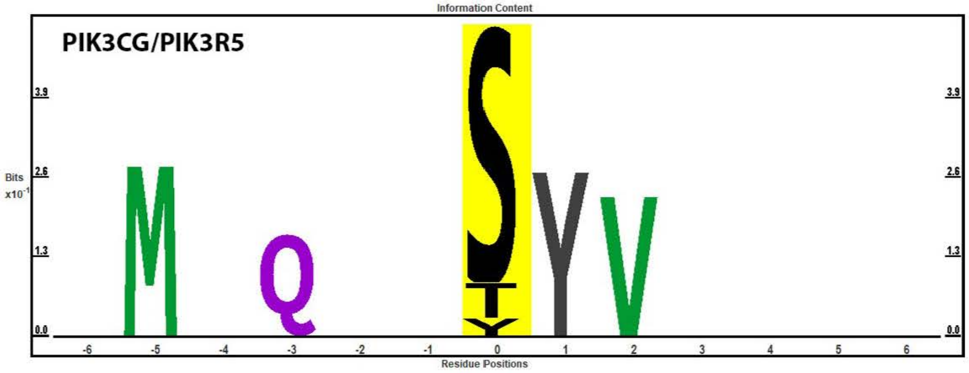
Information Content
PIK3CG/PIK3R5
Bits
x10-1
Residue Positions

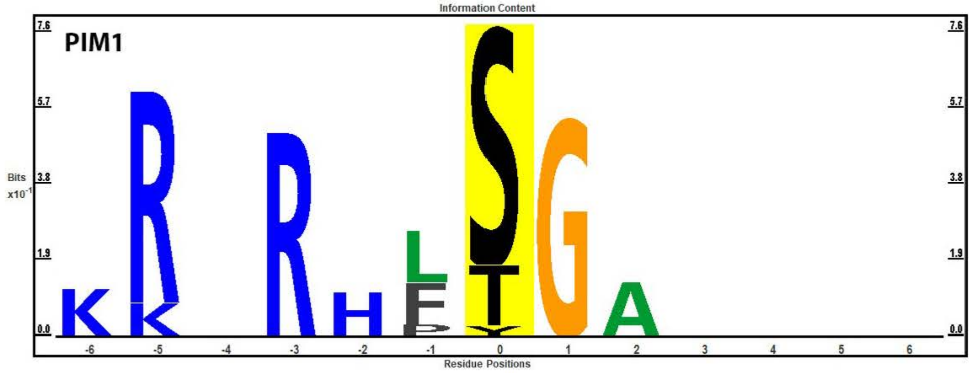
Information Content
PIM1
Bits
x10-1
Residue Positions

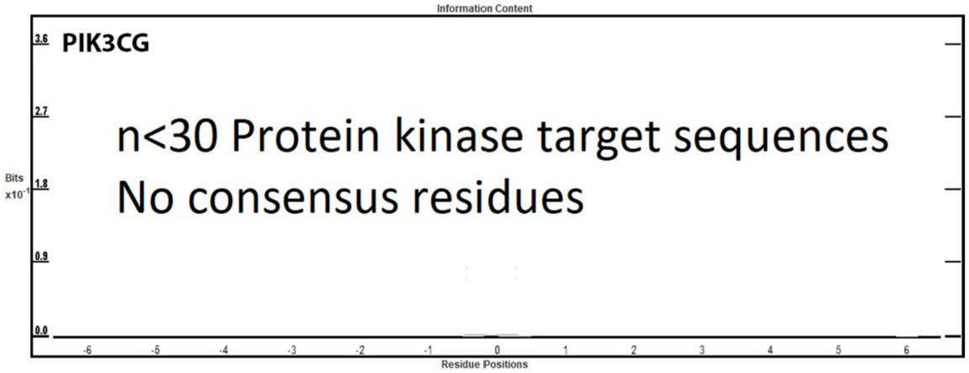
Information Content
PIK3CG
n<30 Protein kinase target sequences
No consensus residues
Bits
x10-1
Residue Positions

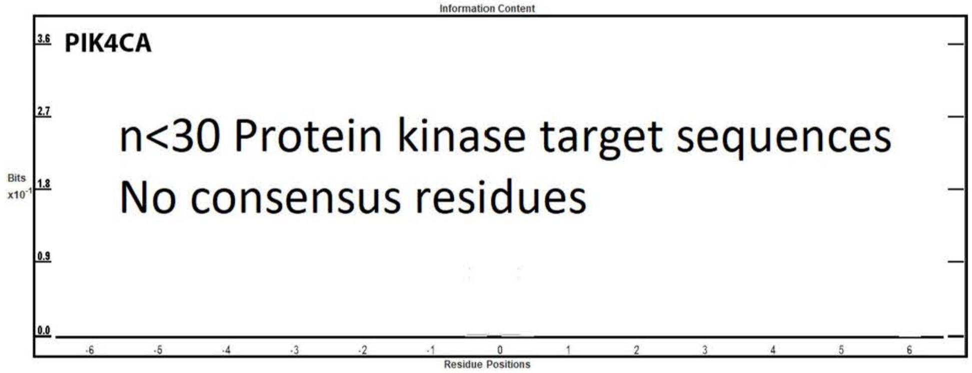
Information Content
PIK4CA
n<30 Protein kinase target sequences
No consensus residues
Bits
x10-1
Residue Positions

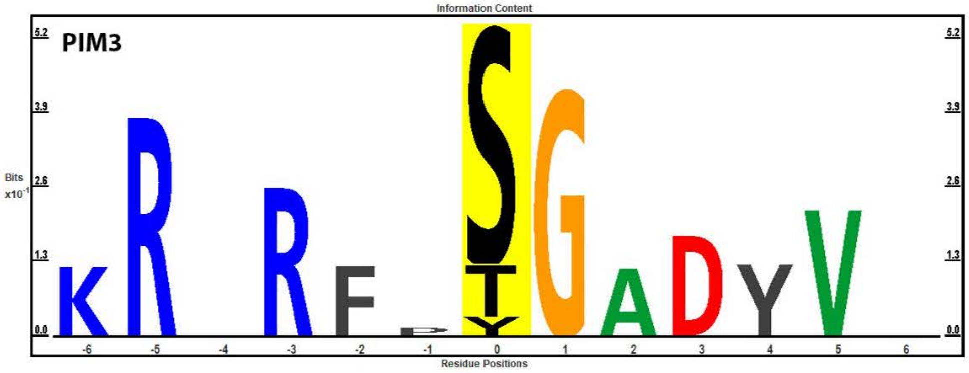
Information Content
PIM3
Bits
x10^-1
Residue Positions

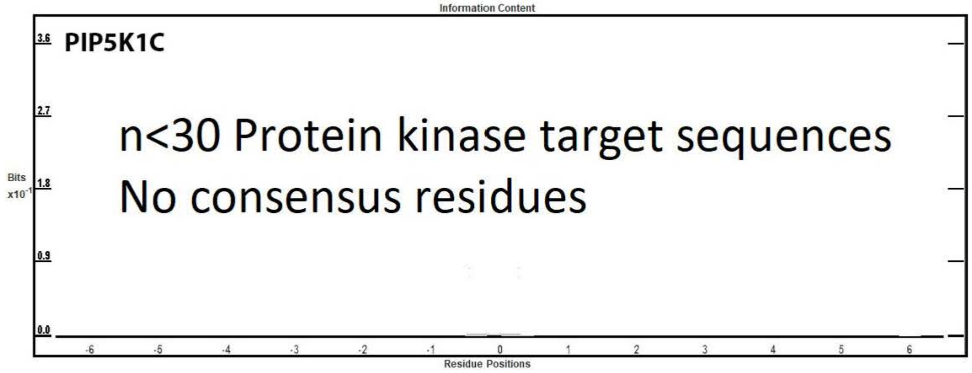
Information Content
PIP5K1C
n<30 Protein kinase target sequences
No consensus residues
Bits
x10^-1
Residue Positions

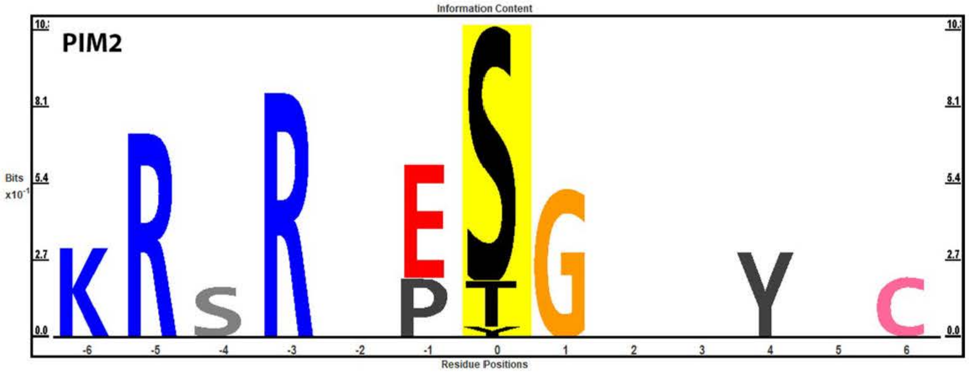
Information Content
PIM2
Bits
x10^-1
Residue Positions

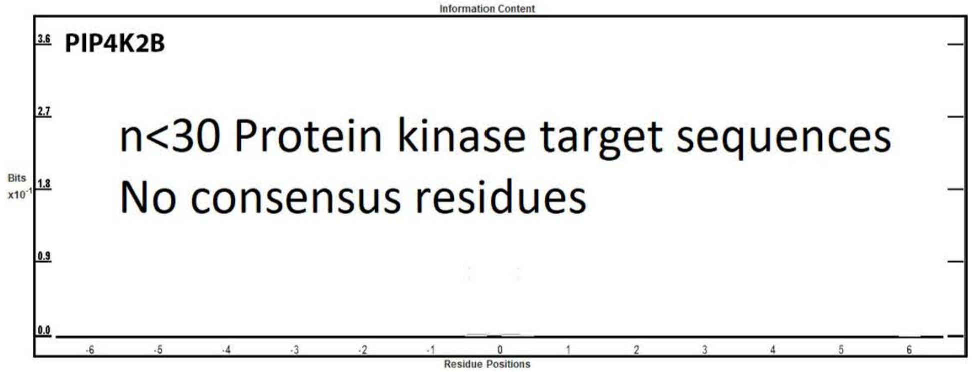
Information Content
PIP4K2B
n<30 Protein kinase target sequences
No consensus residues
Bits
x10^-1
Residue Positions

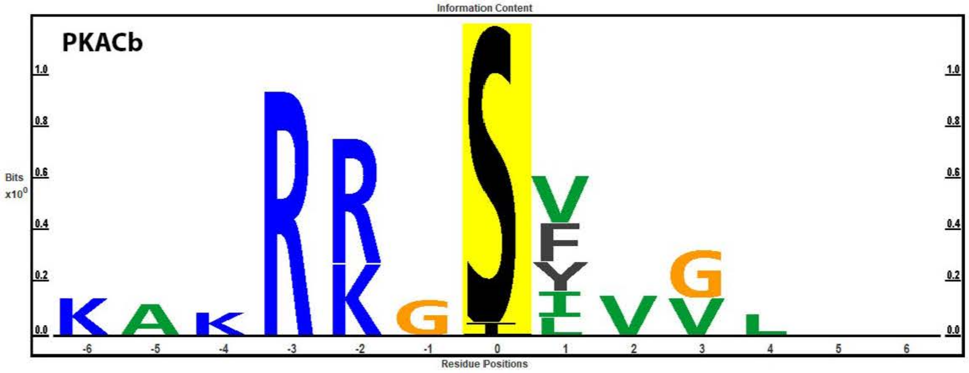
Information Content
PKACb
Bits
x10^0
Residue Positions

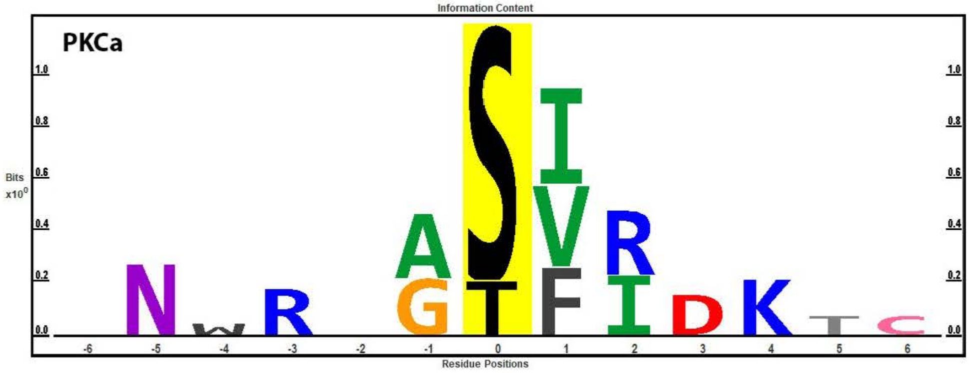
Information Content
PKCa
Bits
x10^0
Residue Positions

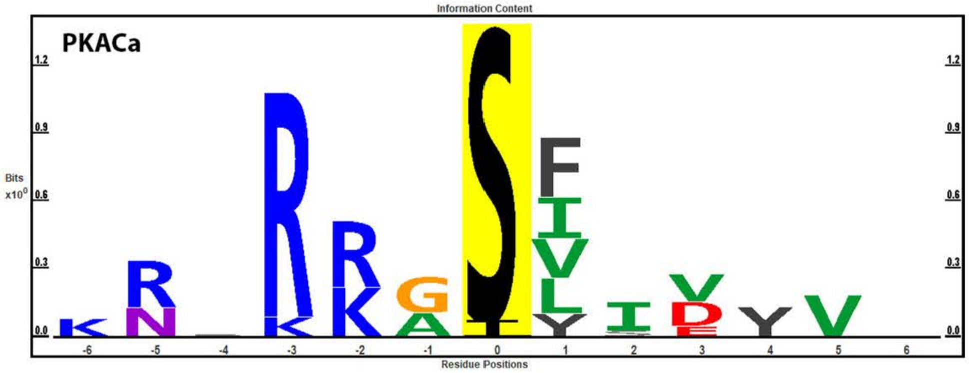
Information Content
PKACa
Bits
x10^0
Residue Positions

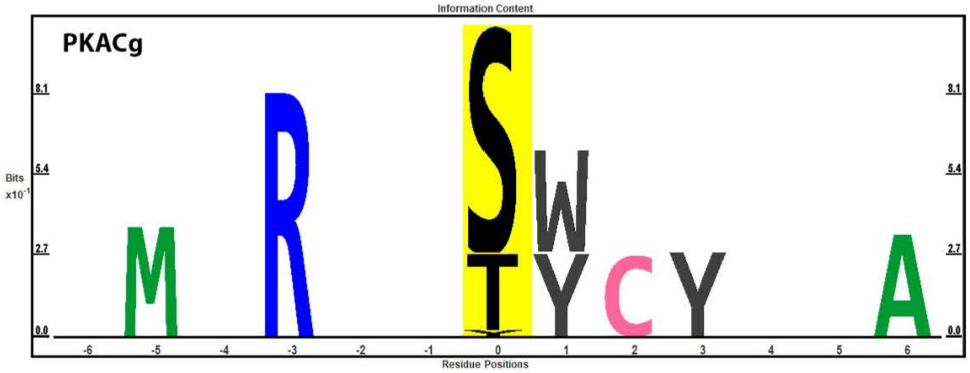
Information Content
PKACg
Bits
x10^-1
Residue Positions

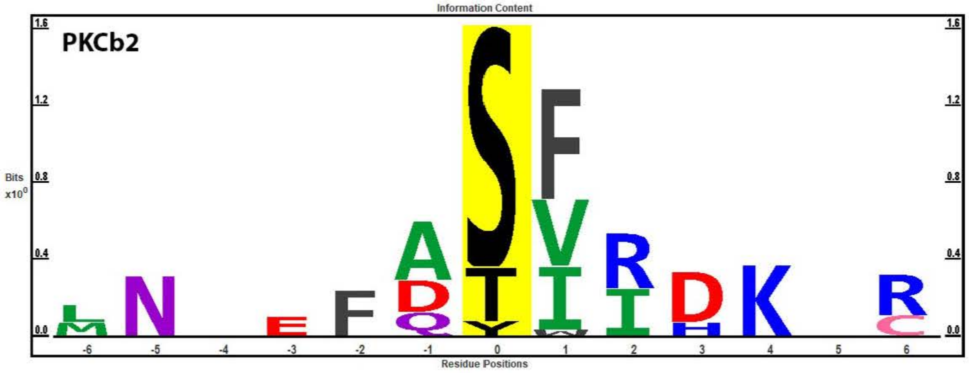
Information Content
PKCb2
Bits
x10^0
Residue Positions

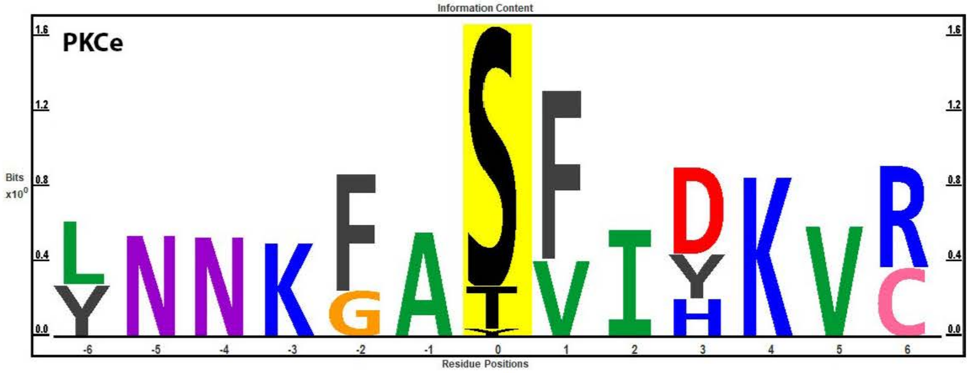
Information Content
PKCe
Bits
x10^0
Residue Positions

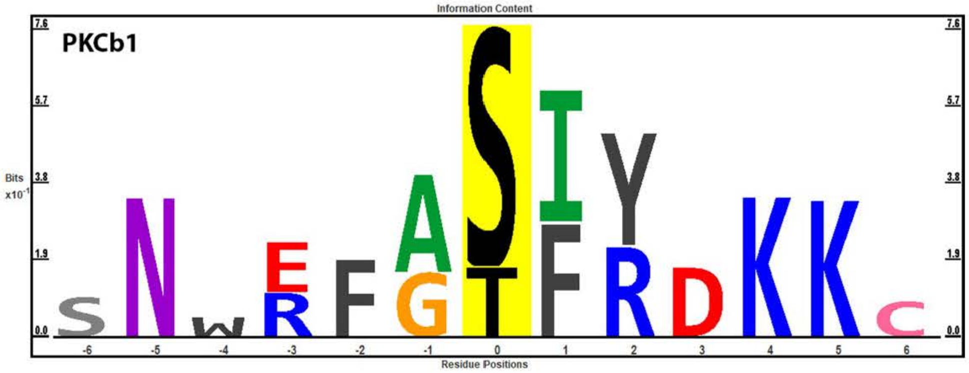
Information Content
PKCb1
Bits
x10^-1
Residue Positions

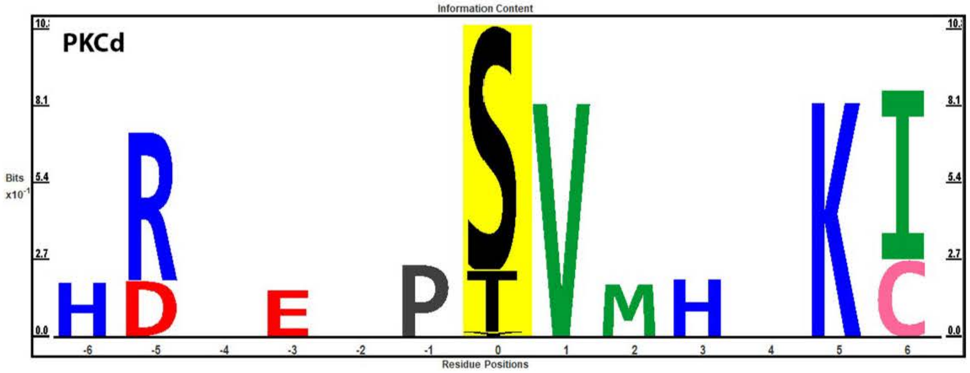
Information Content
PKCd
Bits
x10^-1
Residue Positions

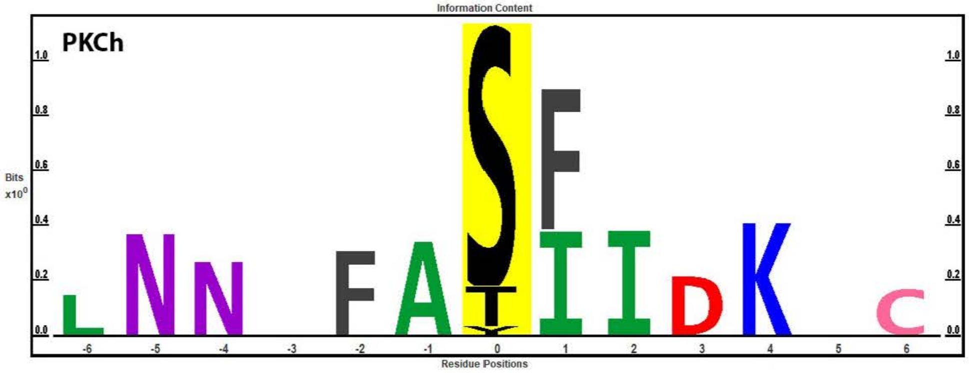
Information Content
PKCh
Bits
x10^0
1.0
0.8
0.6
0.4
0.2
0.0
-6 -5 -4 -3 -2 -1 0 1 2 3 4 5 6
Residue Positions

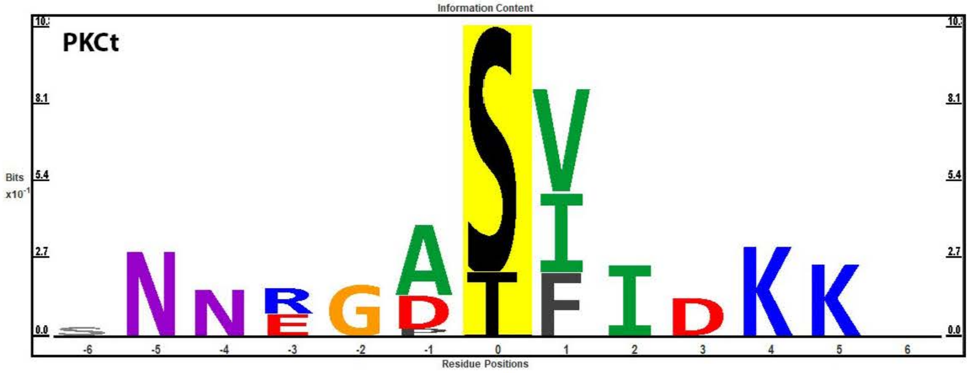
Information Content
PKCt
Bits
x10^-1
8.1
5.4
2.7
0.0
-6 -5 -4 -3 -2 -1 0 1 2 3 4 5 6
Residue Positions

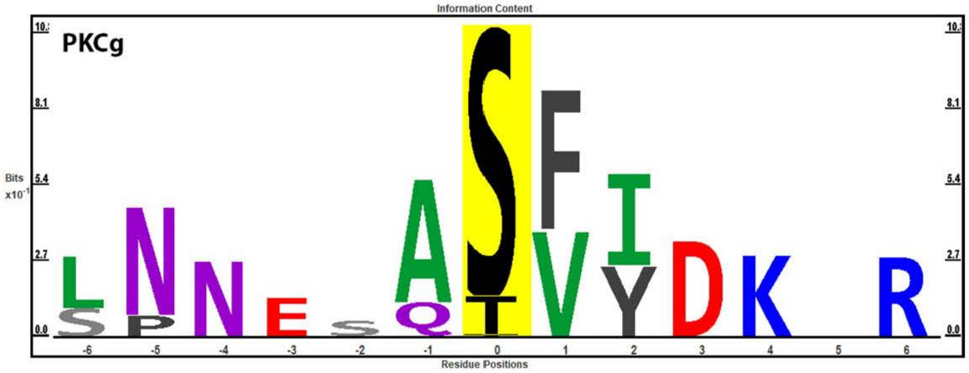
Information Content
PKCg
Bits
x10^-1
8.1
5.4
2.7
0.0
-6 -5 -4 -3 -2 -1 0 1 2 3 4 5 6
Residue Positions

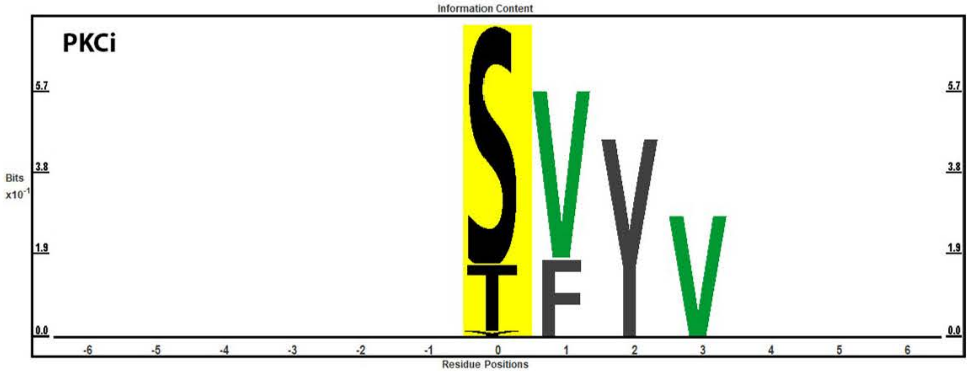
Information Content
PKCi
Bits
x10^-1
5.7
3.8
1.9
0.0
-6 -5 -4 -3 -2 -1 0 1 2 3 4 5 6
Residue Positions

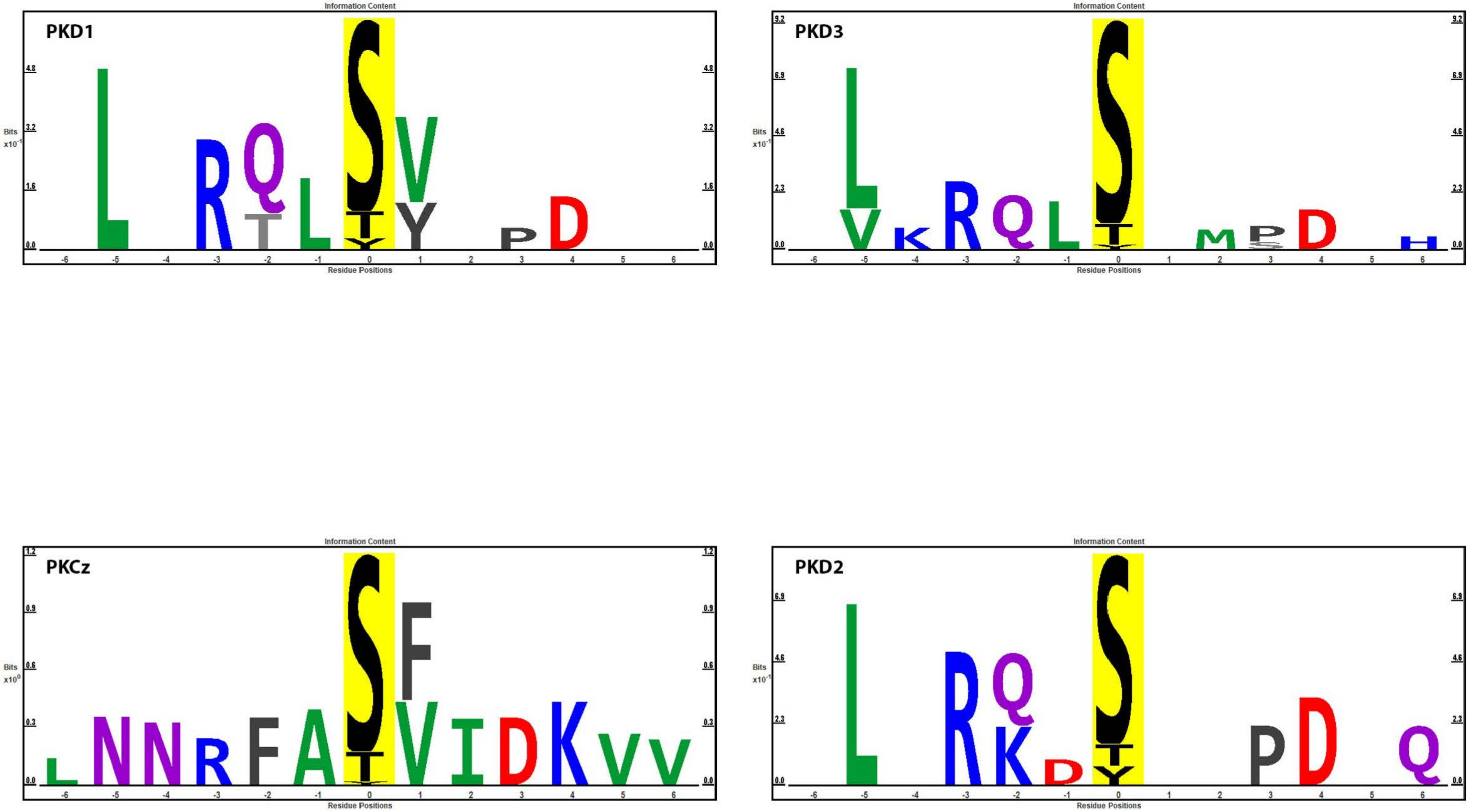

PKD1
PKD3
PKCz
PKD2
Information Content
Bits
Residue Positions

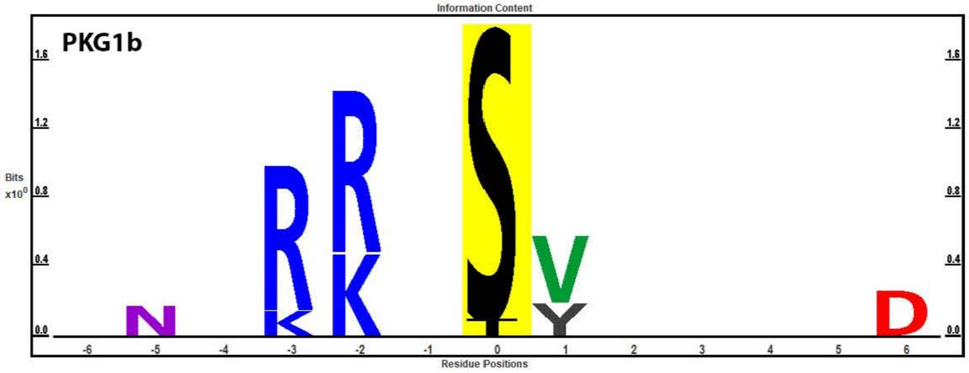
Information Content
PKG1b
Bits
x10⁰
Residue Positions

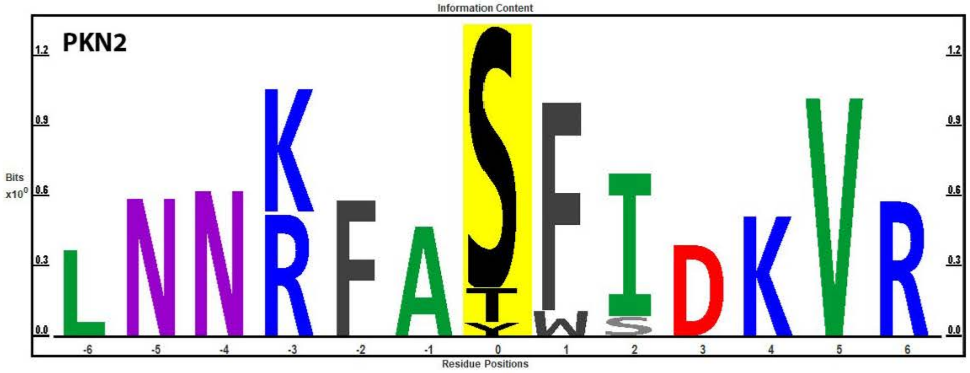
Information Content
PKN2
Bits
x10⁰
Residue Positions

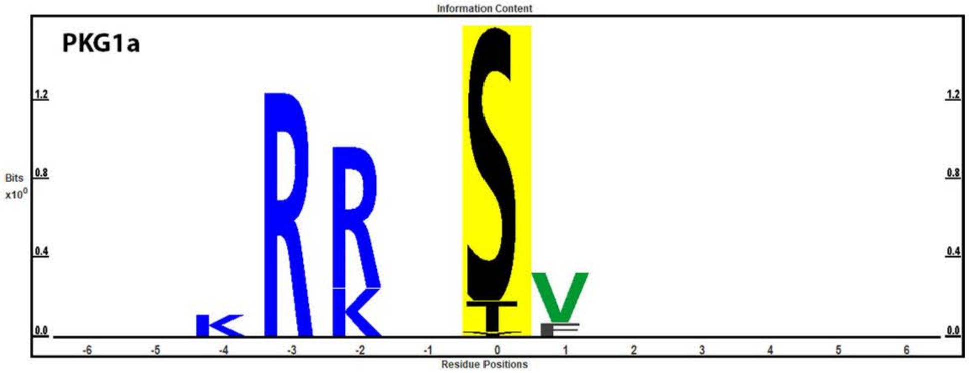
Information Content
PKG1a
Bits
x10⁰
Residue Positions

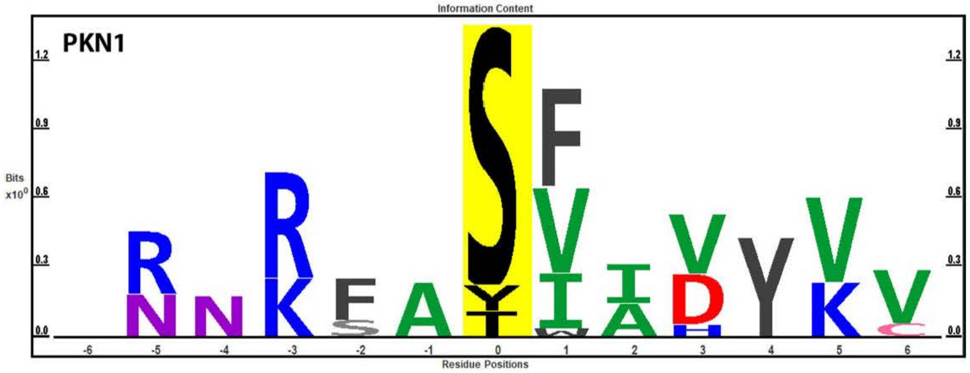
Information Content
PKN1
Bits
x10⁰
Residue Positions

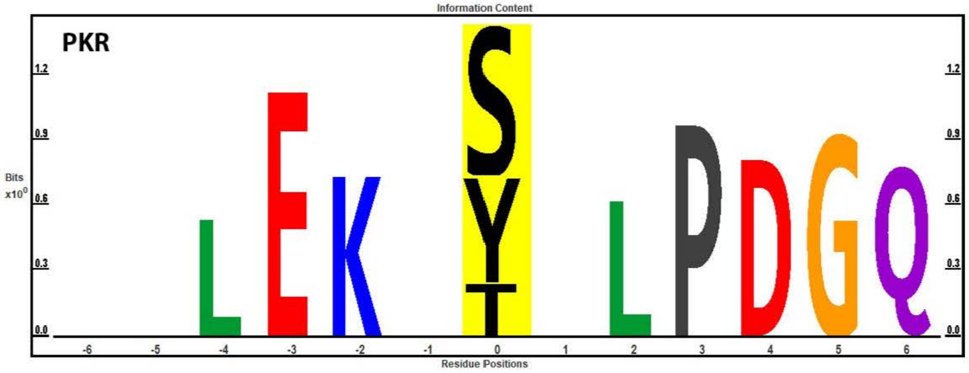
Information Content
PKR
Bits
x10^0
Residue Positions

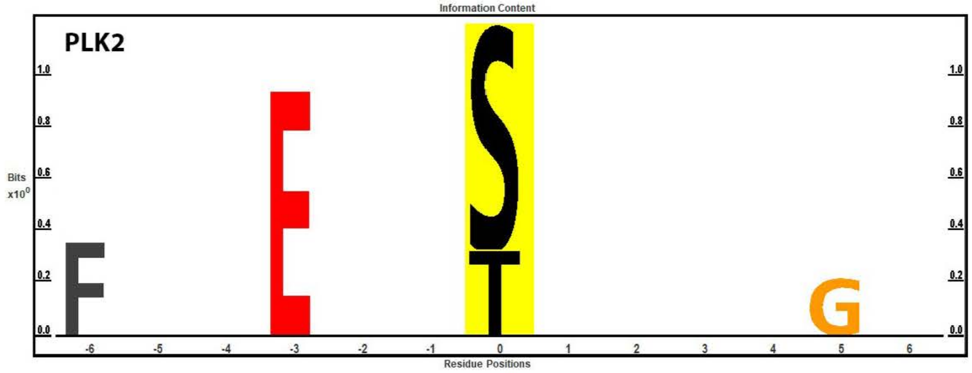
Information Content
PLK2
Bits
x10^0
Residue Positions

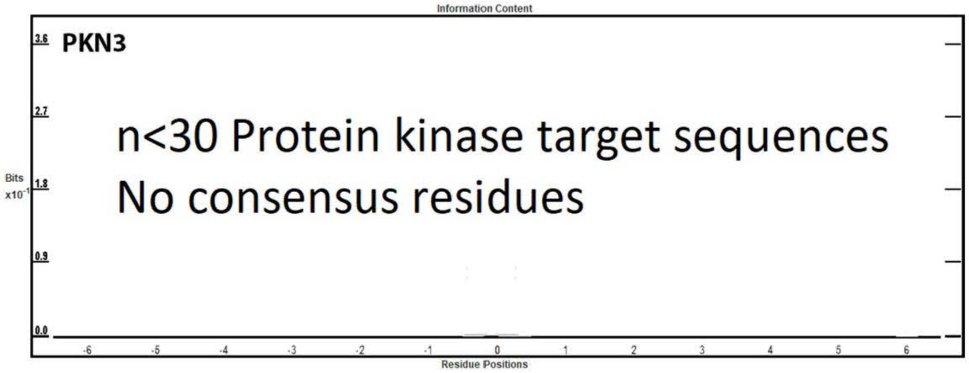
Information Content
PKN3
n<30 Protein kinase target sequences
No consensus residues
Bits
x10^-1
Residue Positions

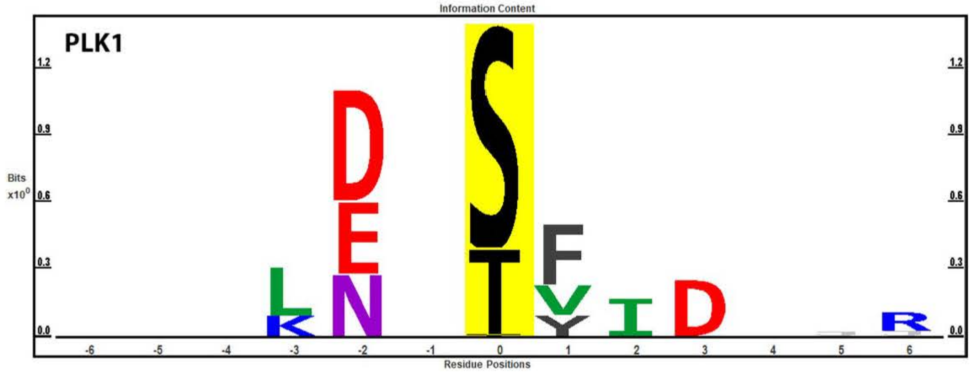
Information Content
PLK1
Bits
x10^0
Residue Positions

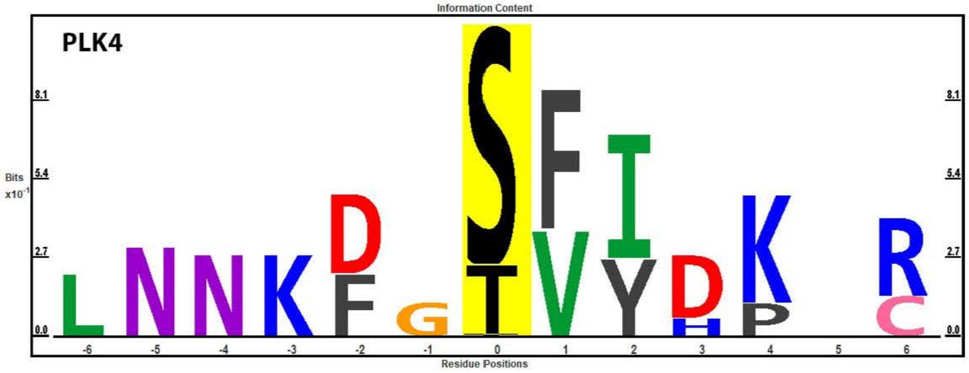
Information Content
PLK4
Bits
x10-1
8.1
5.4
2.7
0.0
-6 -5 -4 -3 -2 -1 0 1 2 3 4 5 6
Residue Positions

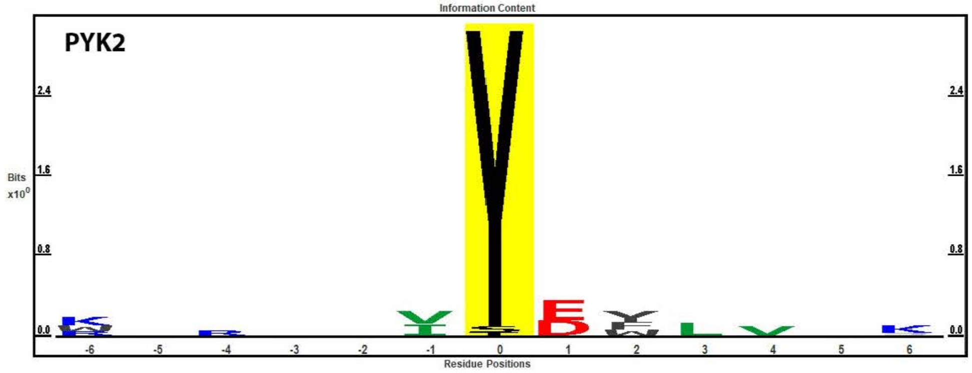
Information Content
PYK2
Bits
x10^0
2.4
1.6
0.8
0.0
-6 -5 -4 -3 -2 -1 0 1 2 3 4 5 6
Residue Positions

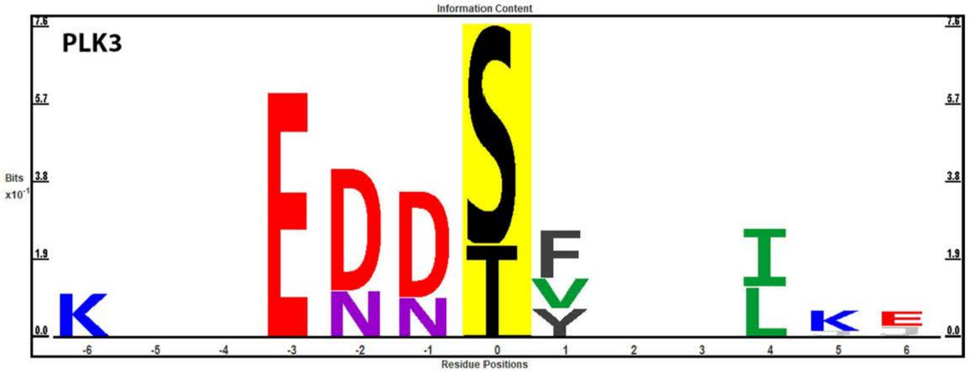
Information Content
PLK3
Bits
x10-1
7.6
5.7
3.8
1.9
0.0
-6 -5 -4 -3 -2 -1 0 1 2 3 4 5 6
Residue Positions

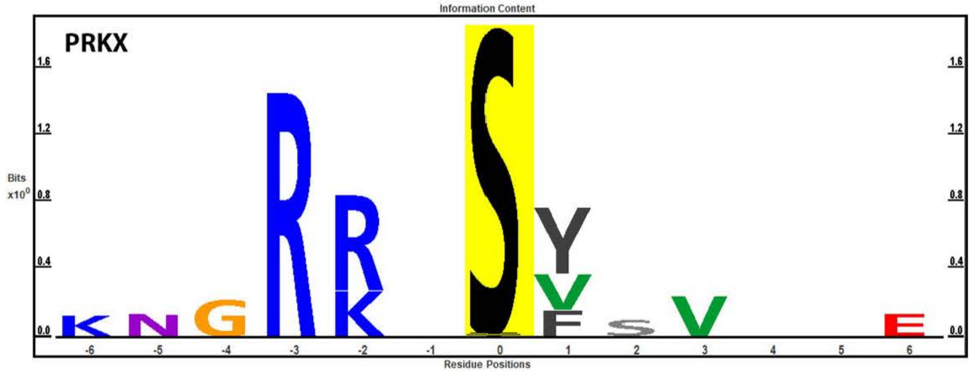
Information Content
PRKX
Bits
x10^0
1.6
1.2
0.8
0.4
0.0
-6 -5 -4 -3 -2 -1 0 1 2 3 4 5 6
Residue Positions

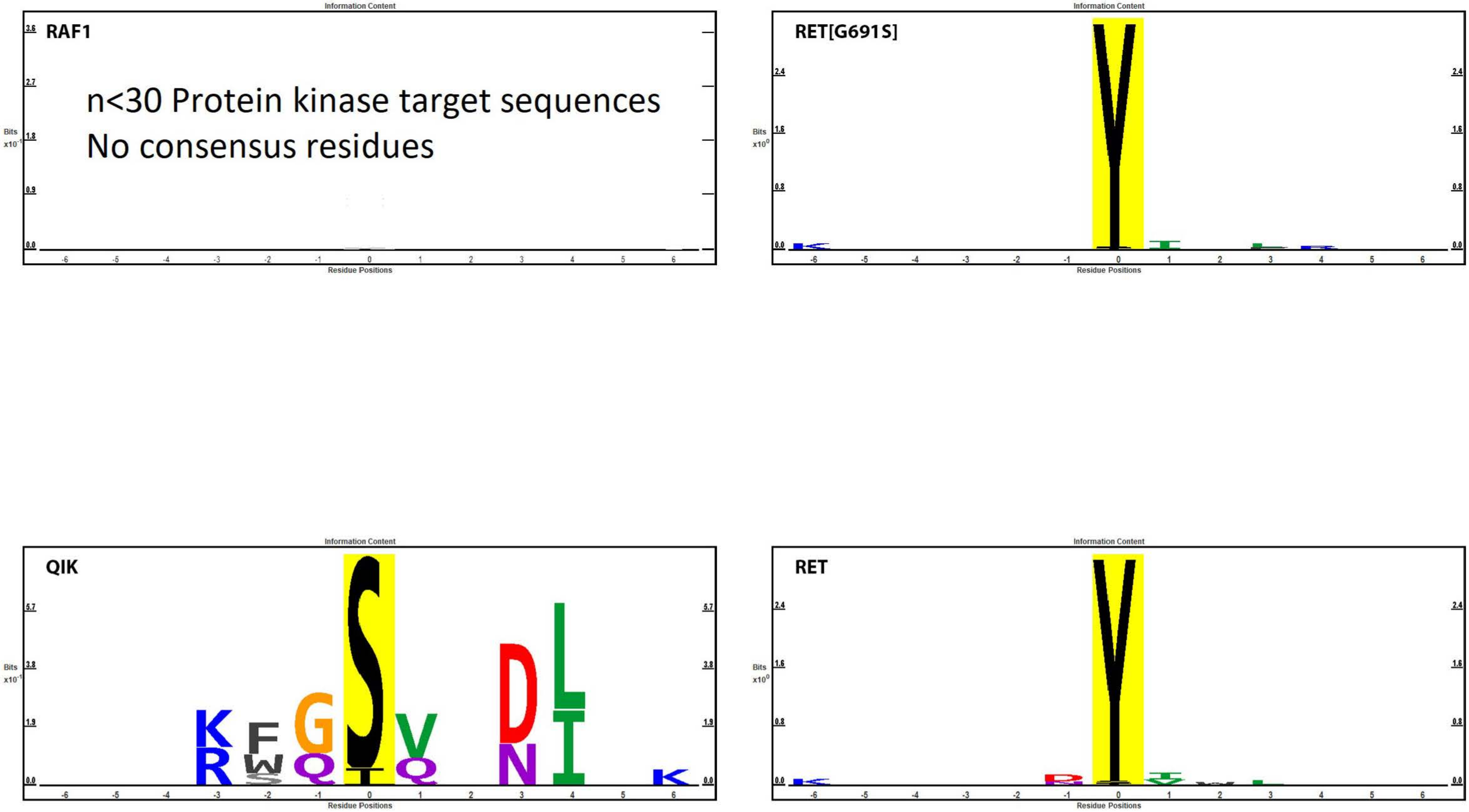

Information Content
RAF1
n<30 Protein kinase target sequences
No consensus residues
Bits
x10⁻¹
Residue Positions
RET[G691S]
Bits
x10⁰
Residue Positions
QIK
Bits
x10⁻¹
Residue Positions
RET
Bits
x10⁰
Residue Positions

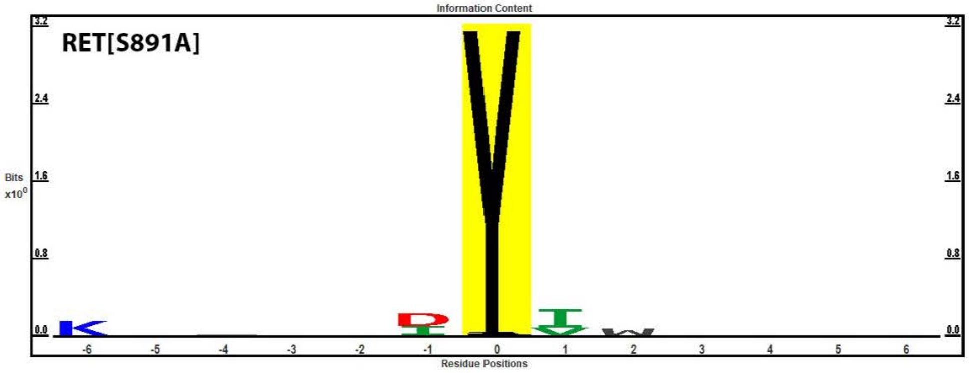
Information Content
RET[S891A]
Bits
x10^0
Residue Positions

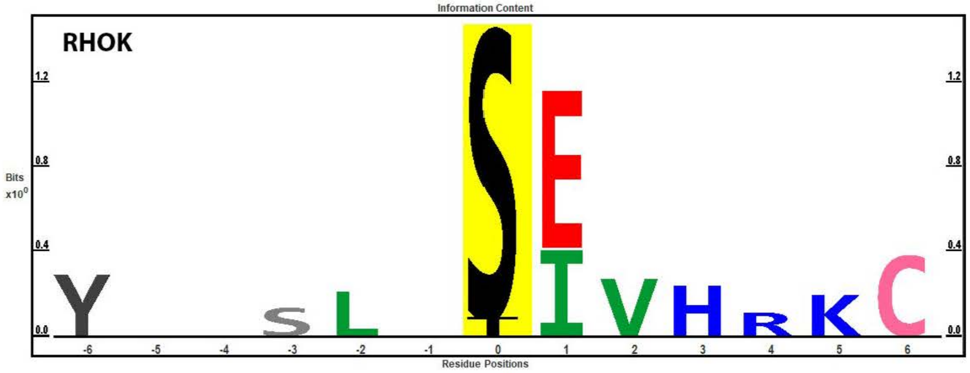
Information Content
RHOK
Bits
x10^0
Residue Positions

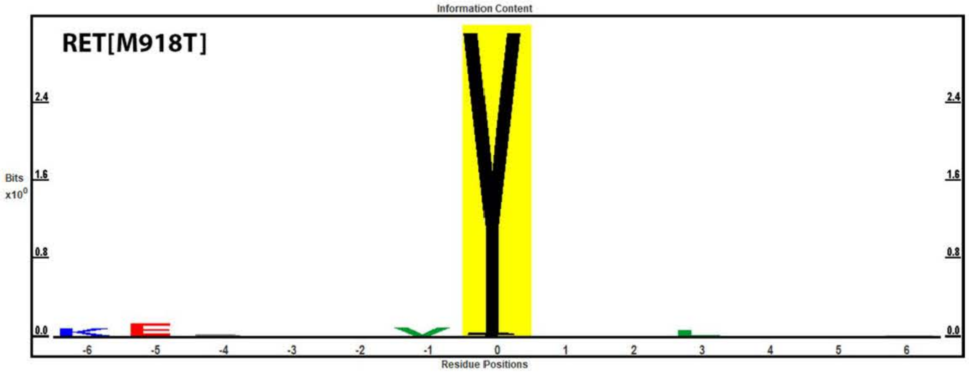
Information Content
RET[M918T]
Bits
x10^0
Residue Positions

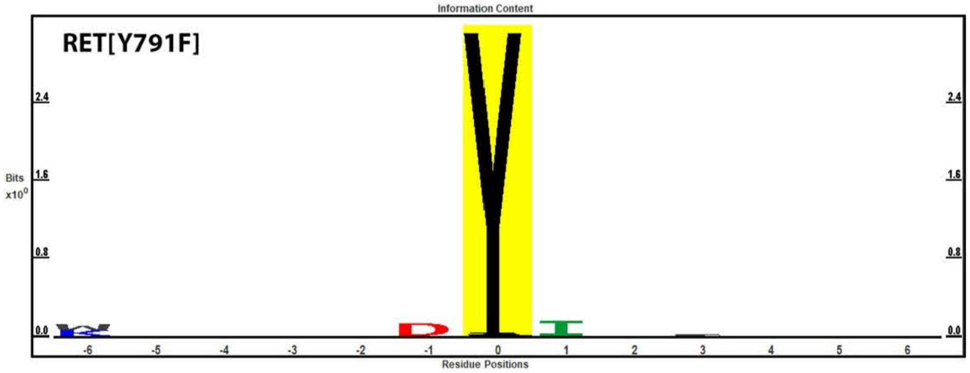
Information Content
RET[Y791F]
Bits
x10^0
Residue Positions

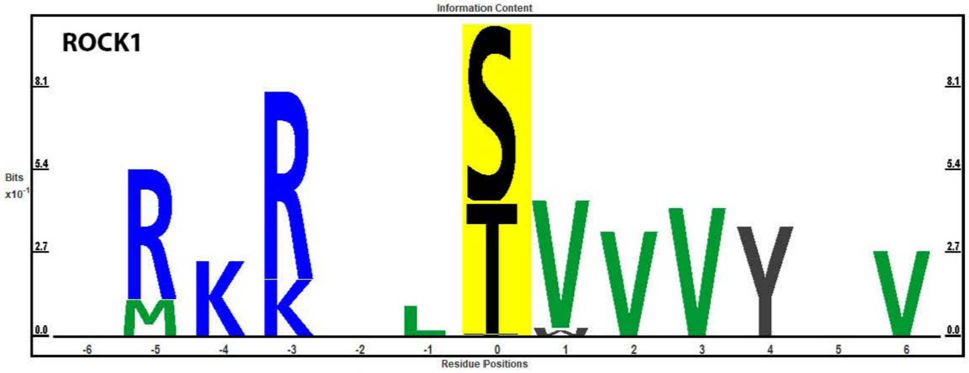
ROCK1
Information Content
Bits x10^-1
Residue Positions

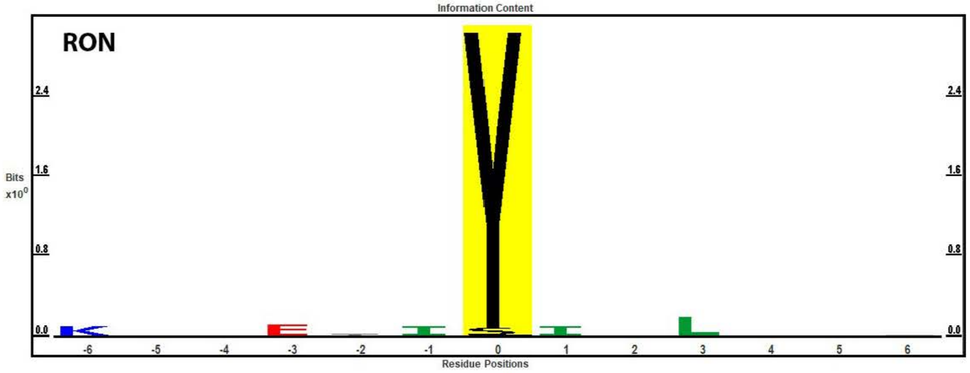
RON
Information Content
Bits x10^0
Residue Positions

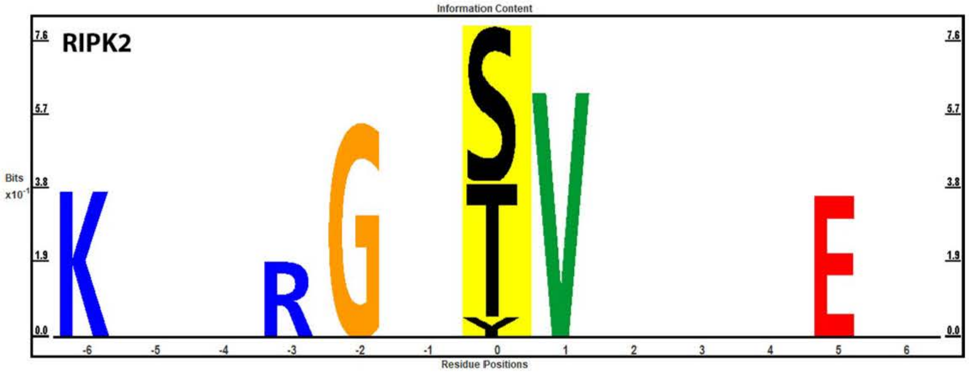
RIPK2
Information Content
Bits x10^-1
Residue Positions

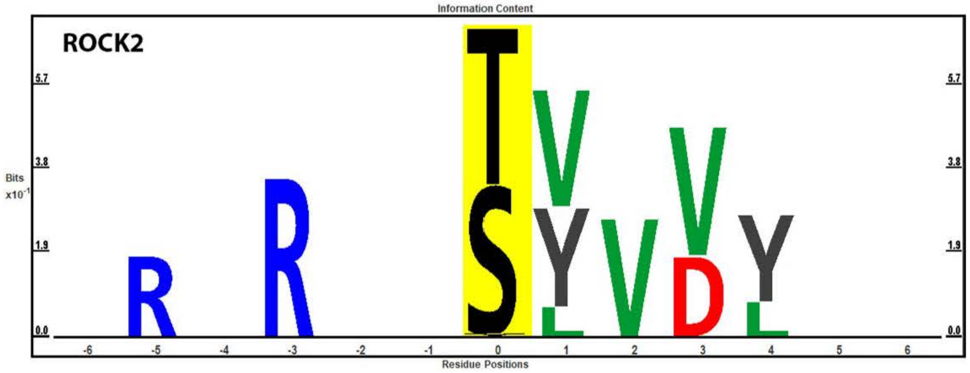
ROCK2
Information Content
Bits x10^-1
Residue Positions

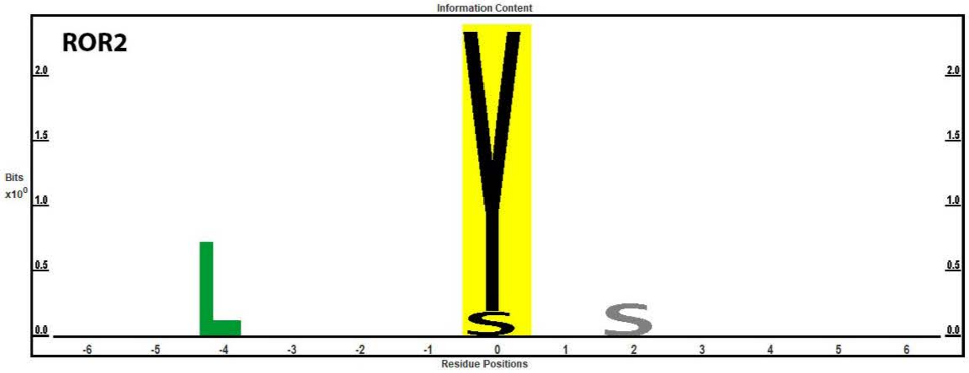
Information Content
ROR2
Bits
Residue Positions

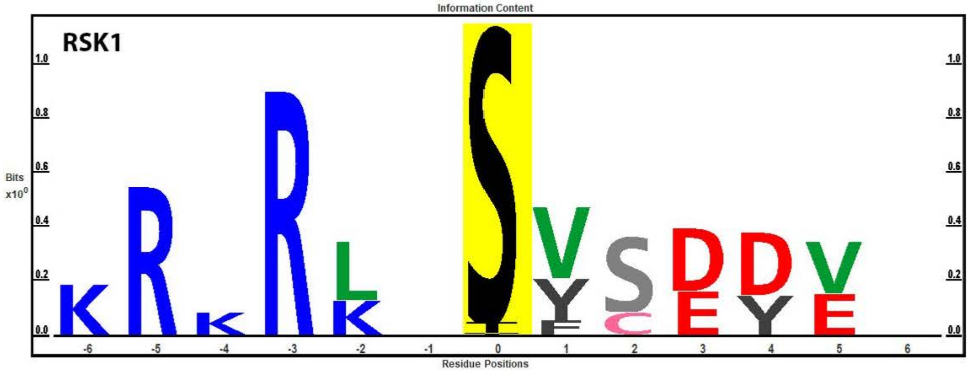
Information Content
RSK1
Bits
Residue Positions

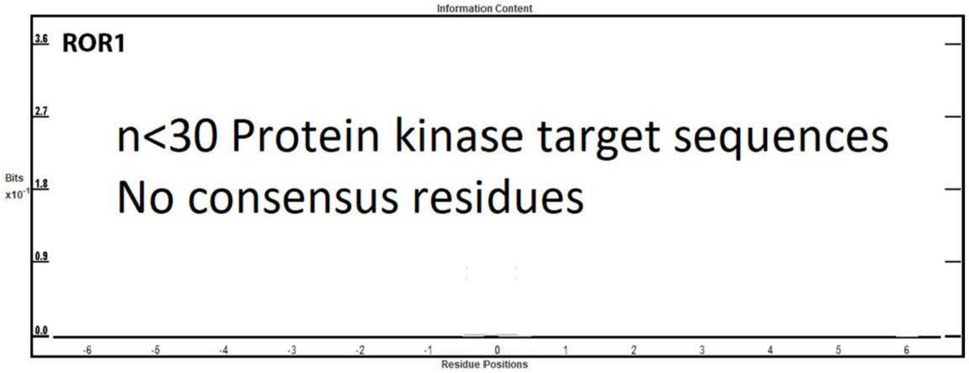
Information Content
ROR1
n<30 Protein kinase target sequences
No consensus residues
Bits
Residue Positions

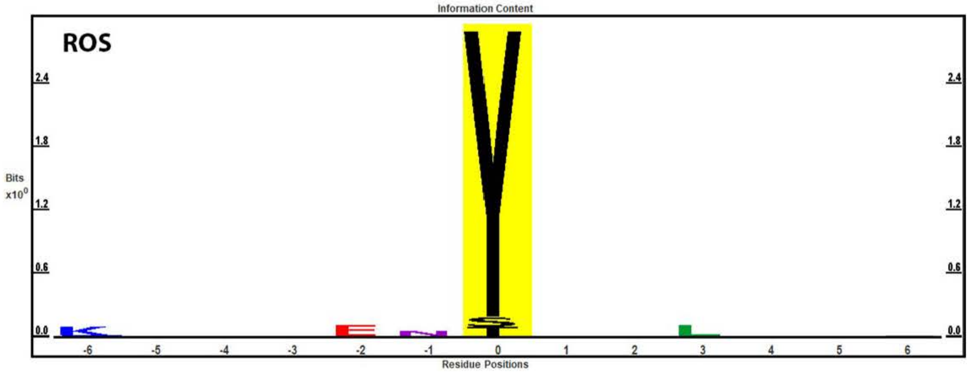
Information Content
ROS
Bits
Residue Positions

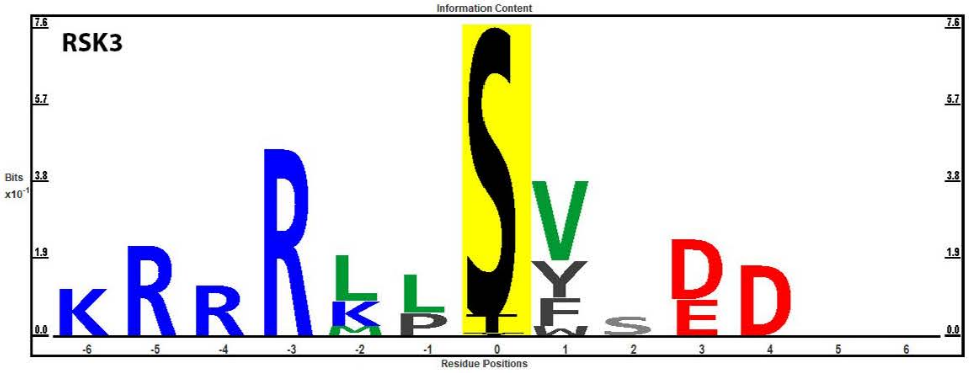
RSK3
Information Content
Bits
x10-1
7.6
5.7
3.8
1.9
0.0
-6 -5 -4 -3 -2 -1 0 1 2 3 4 5 6
Residue Positions

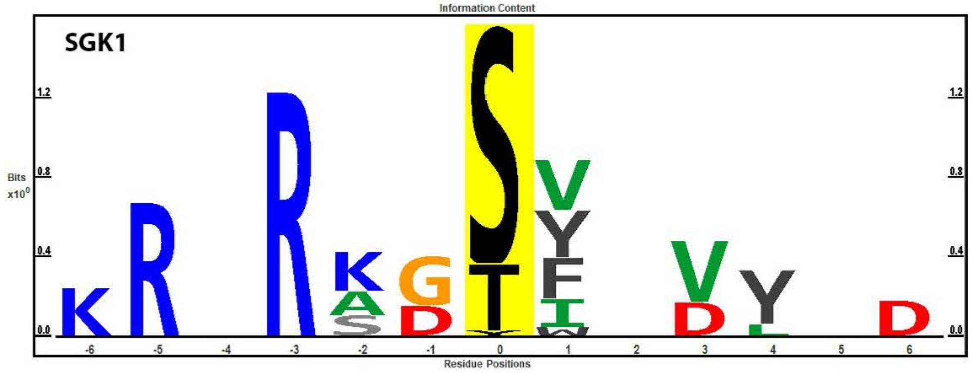
SGK1
Information Content
Bits
x10^0
1.2
0.8
0.4
0.0
-6 -5 -4 -3 -2 -1 0 1 2 3 4 5 6
Residue Positions

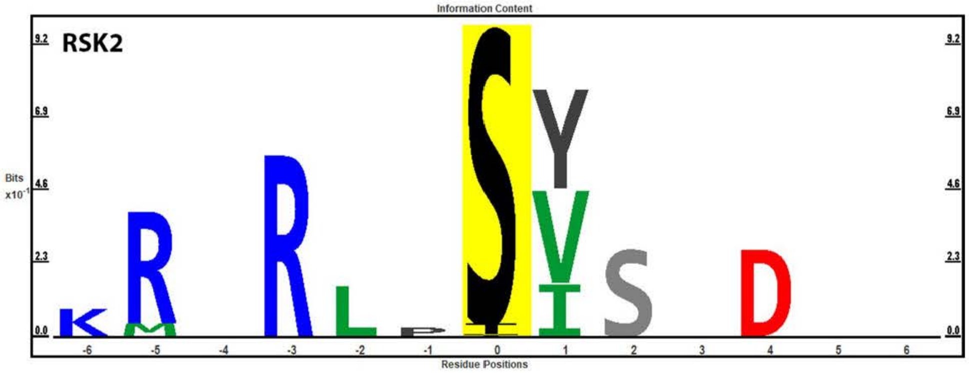
RSK2
Information Content
Bits
x10-1
9.2
6.9
4.6
2.3
0.0
-6 -5 -4 -3 -2 -1 0 1 2 3 4 5 6
Residue Positions

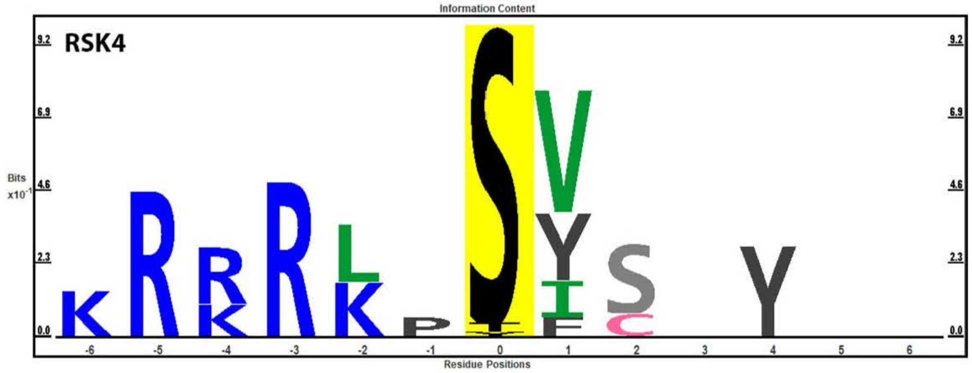
RSK4
Information Content
Bits
x10-1
9.2
6.9
4.6
2.3
0.0
-6 -5 -4 -3 -2 -1 0 1 2 3 4 5 6
Residue Positions

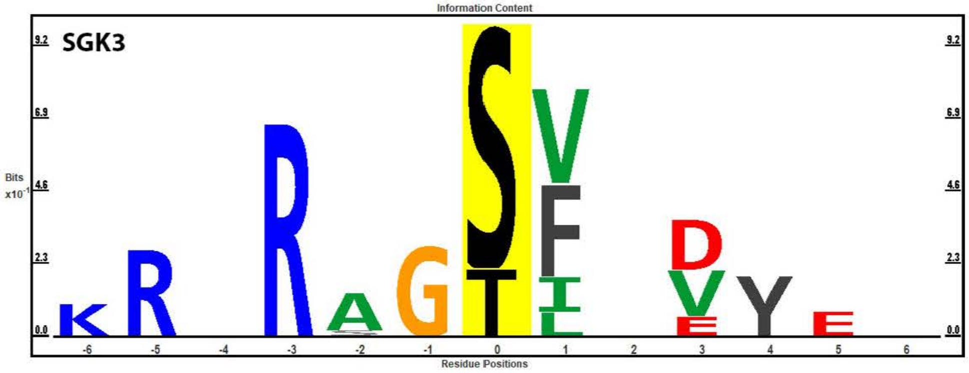
Information Content
SGK3
Bits
Residue Positions

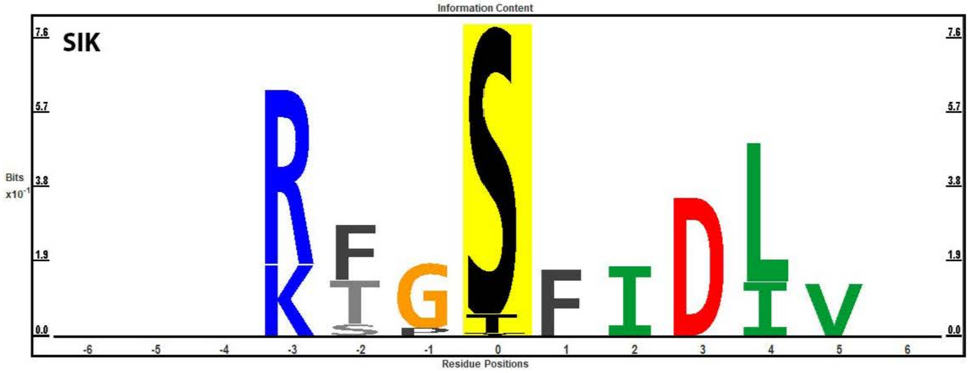
Information Content
SIK
Bits
Residue Positions

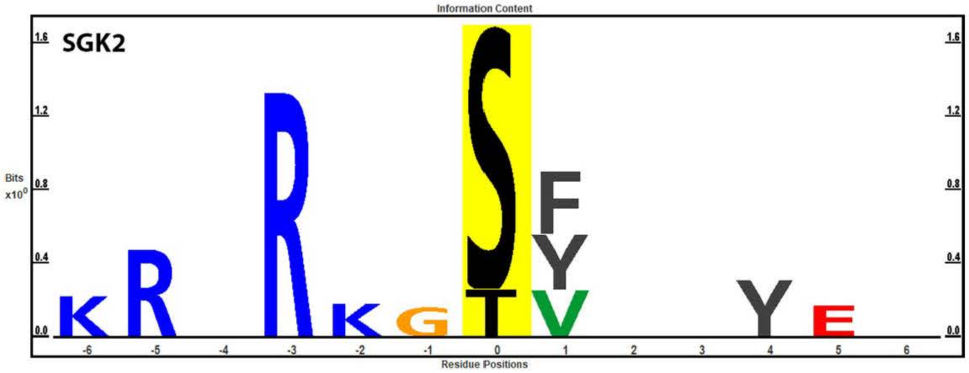
Information Content
SGK2
Bits
Residue Positions

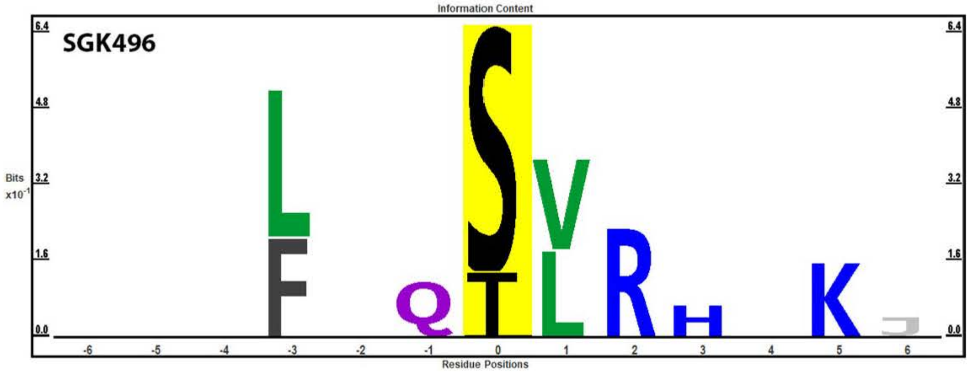
Information Content
SGK496
Bits
Residue Positions

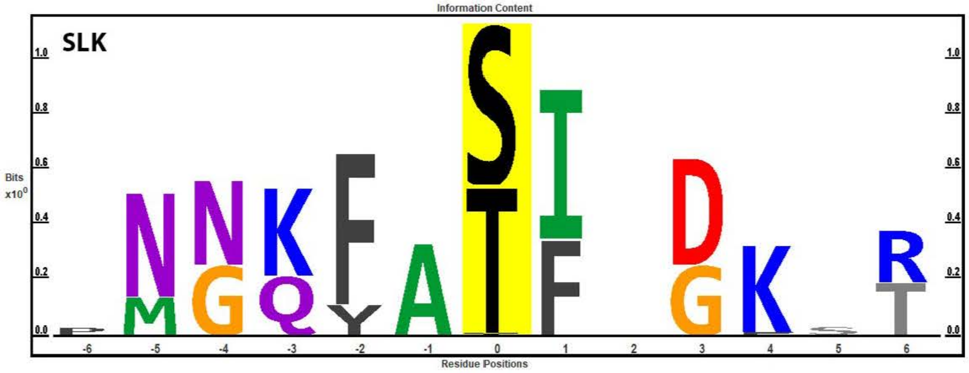
Information Content
SLK
Bits
x10^0
Residue Positions

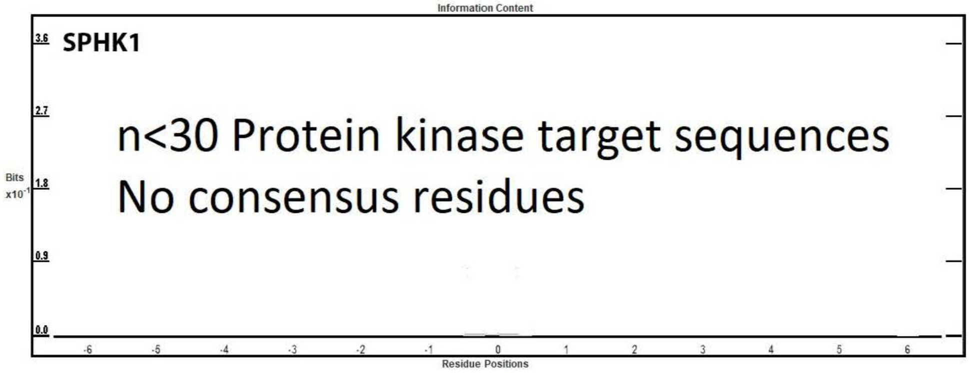
Information Content
SPHK1
n<30 Protein kinase target sequences
No consensus residues
Bits
x10^-1
Residue Positions

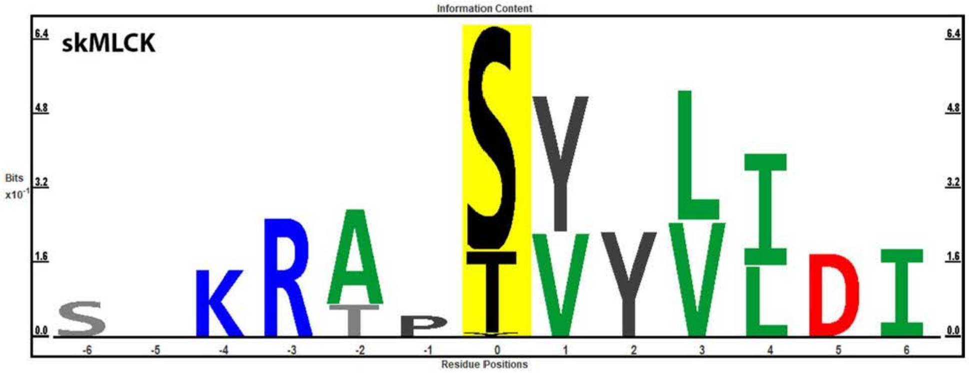
Information Content
skMLCK
Bits
x10^-1
Residue Positions

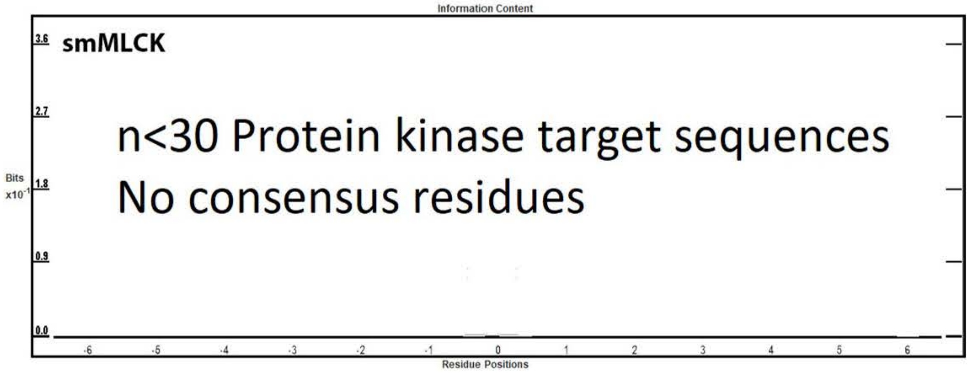
Information Content
smMLCK
n<30 Protein kinase target sequences
No consensus residues
Bits
x10^-1
Residue Positions

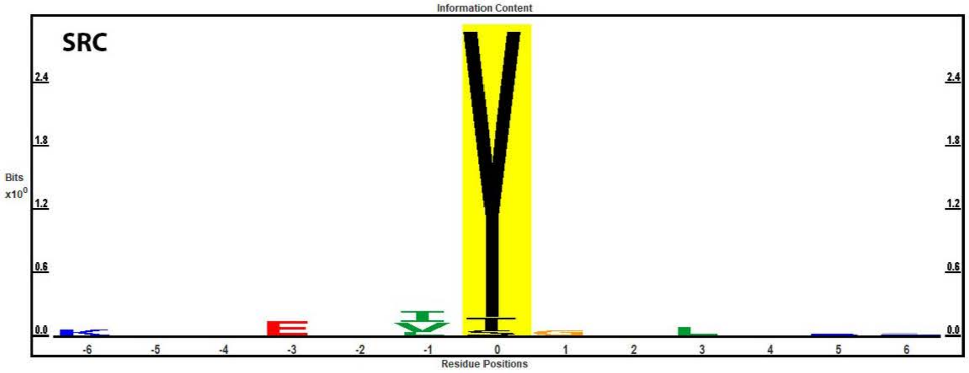
Information Content
SRC
Bits x10^0
2.4
1.8
1.2
0.6
0.0
-6 -5 -4 -3 -2 -1 0 1 2 3 4 5 6
Residue Positions

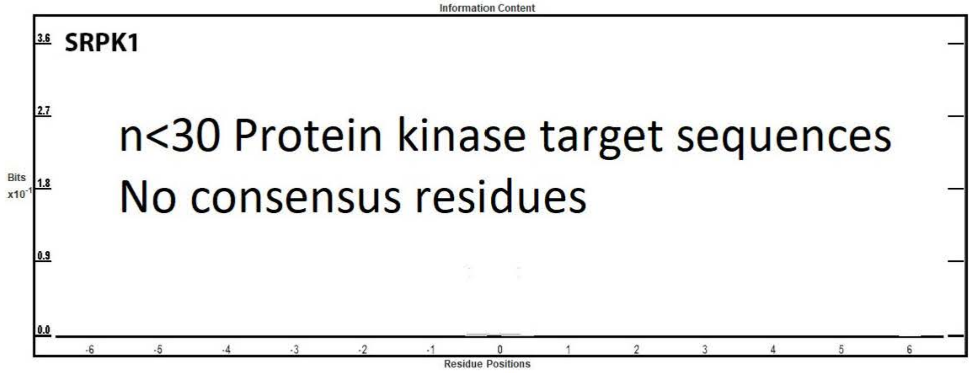
Information Content
SRPK1
3.6
2.7
1.8
0.9
0.0
Bits x10^-1
n<30 Protein kinase target sequences
No consensus residues
-6 -5 -4 -3 -2 -1 0 1 2 3 4 5 6
Residue Positions

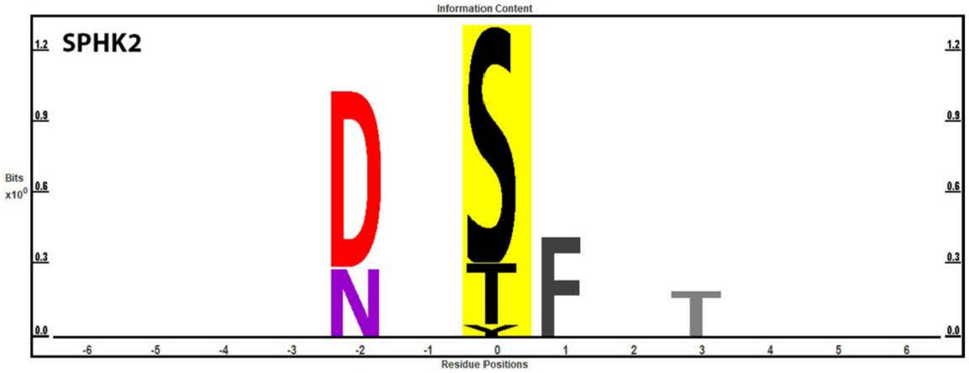
Information Content
SPHK2
Bits x10^0
1.2
0.9
0.6
0.3
0.0
-6 -5 -4 -3 -2 -1 0 1 2 3 4 5 6
Residue Positions

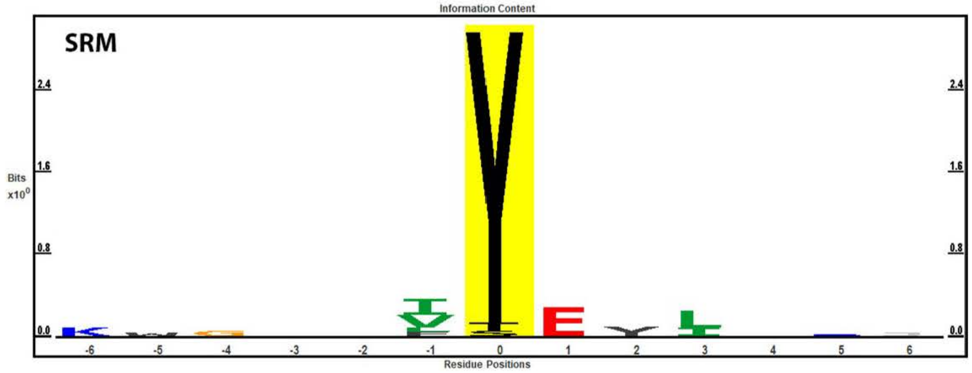
Information Content
SRM
Bits x10^0
2.4
1.6
0.8
0.0
-6 -5 -4 -3 -2 -1 0 1 2 3 4 5 6
Residue Positions

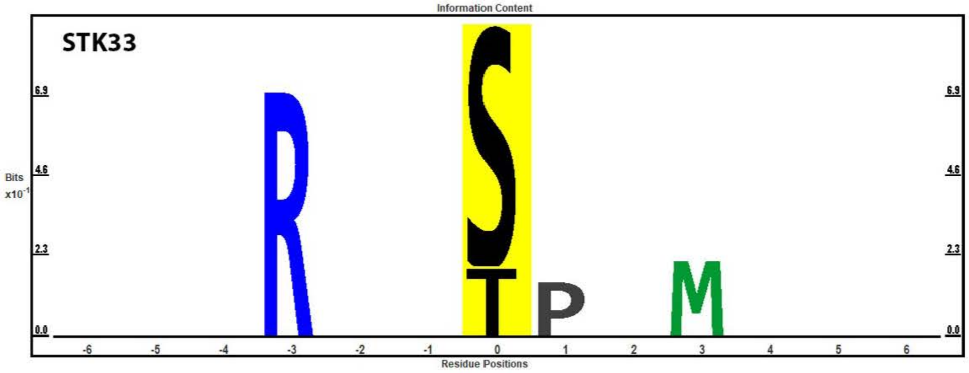
Information Content
STK33
Bits
x10-1
6.9
4.6
2.3
0.0
-6 -5 -4 -3 -2 -1 0 1 2 3 4 5 6
Residue Positions

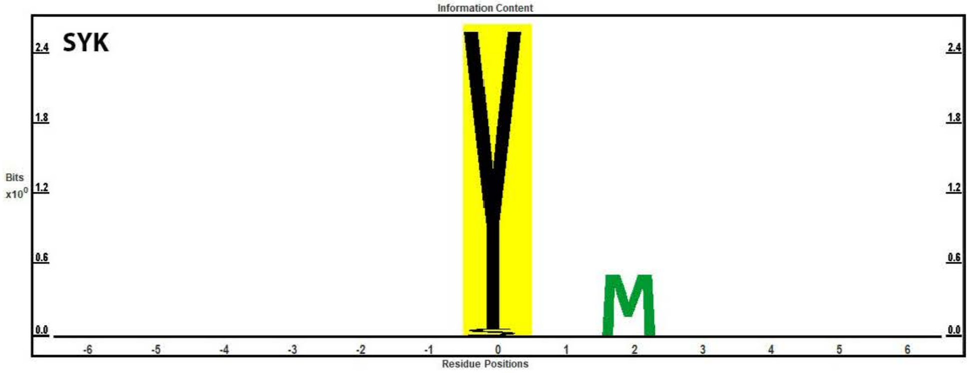
Information Content
SYK
Bits
x10^0
2.4
1.8
1.2
0.6
0.0
-6 -5 -4 -3 -2 -1 0 1 2 3 4 5 6
Residue Positions

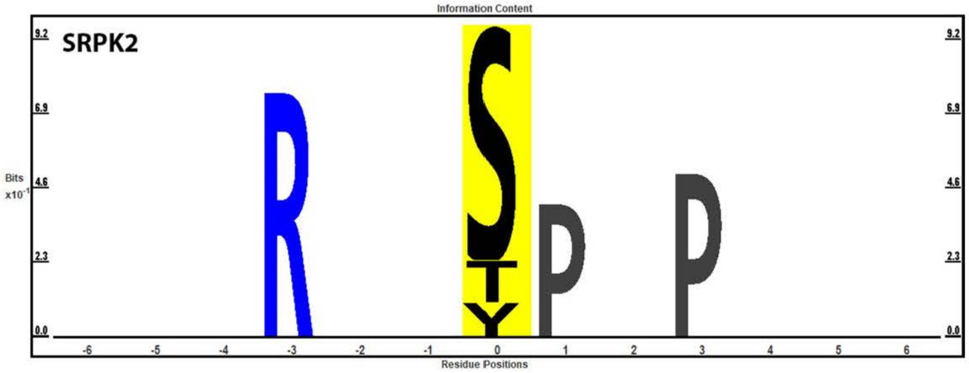
Information Content
SRPK2
Bits
x10-1
9.2
6.9
4.6
2.3
0.0
-6 -5 -4 -3 -2 -1 0 1 2 3 4 5 6
Residue Positions

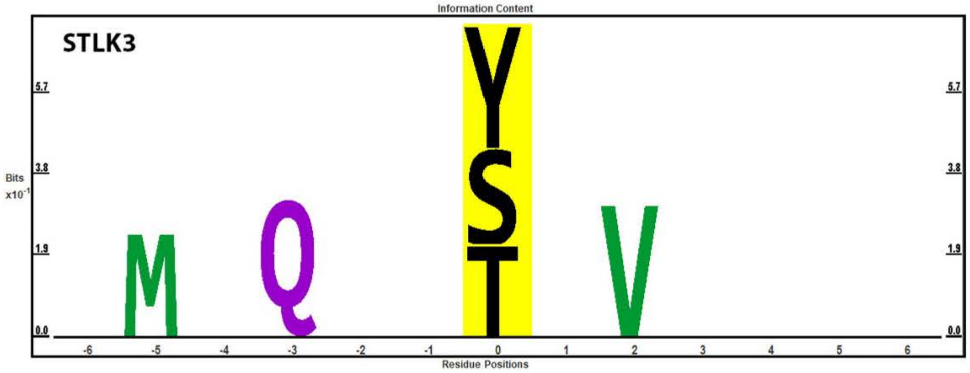
Information Content
STLK3
Bits
x10-1
5.7
3.8
1.9
0.0
-6 -5 -4 -3 -2 -1 0 1 2 3 4 5 6
Residue Positions

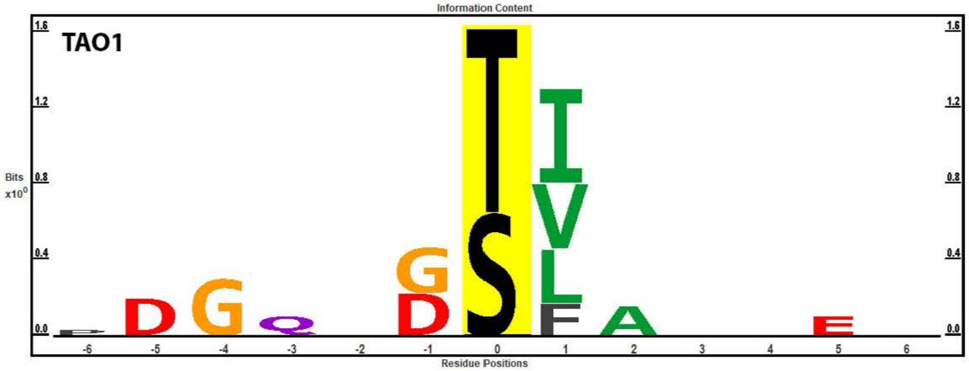
Information Content
TAO1
Bits
Residue Positions

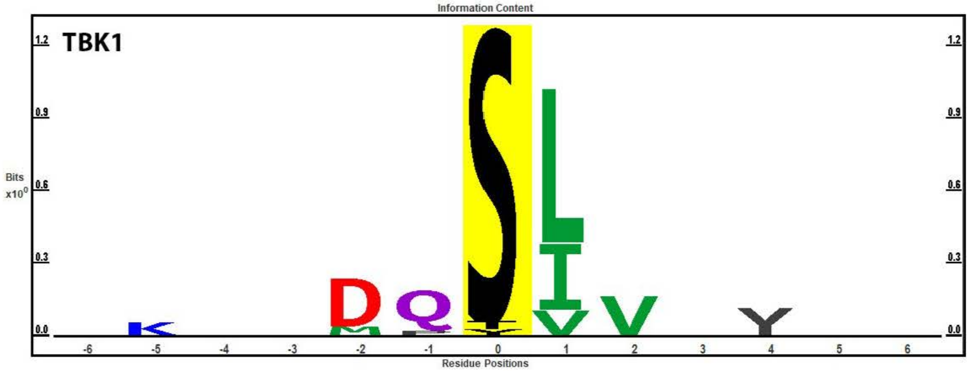
Information Content
TBK1
Bits
Residue Positions

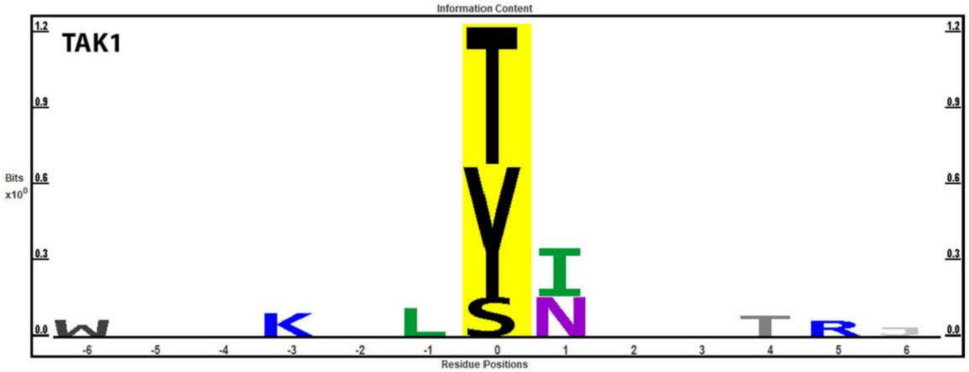
Information Content
TAK1
Bits
Residue Positions

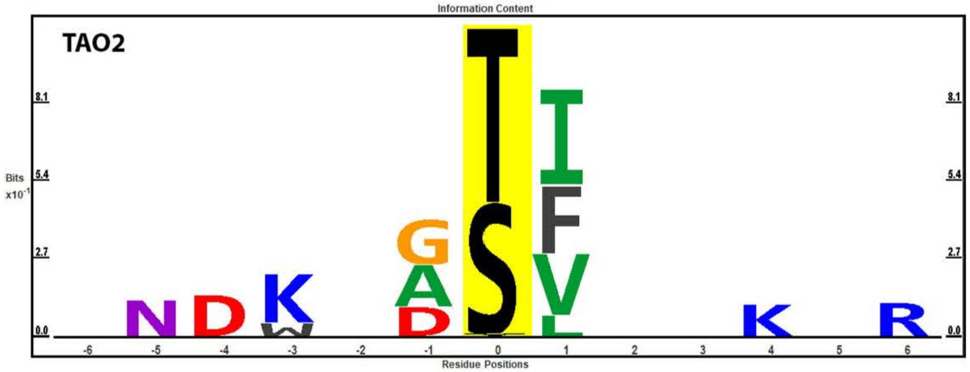
Information Content
TAO2
Bits
Residue Positions

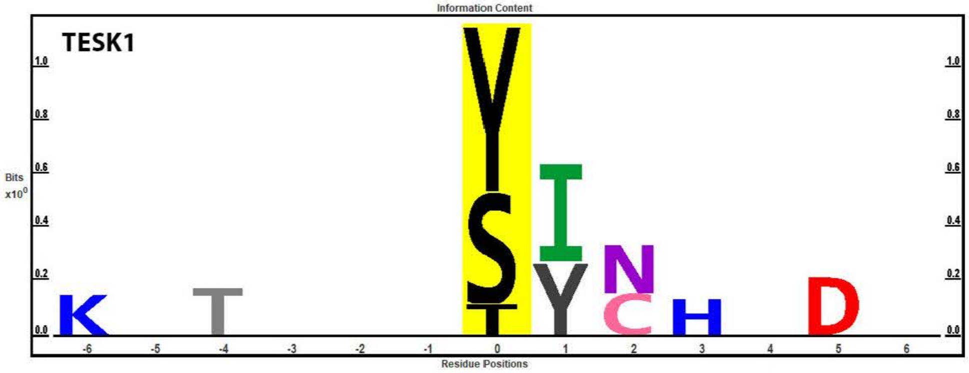
Information Content
TESK1
Bits
x10^0
Residue Positions

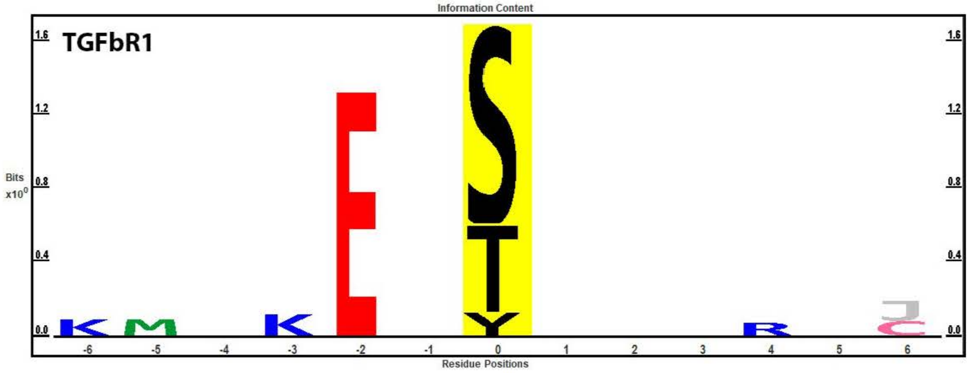
Information Content
TGFbR1
Bits
x10^0
Residue Positions

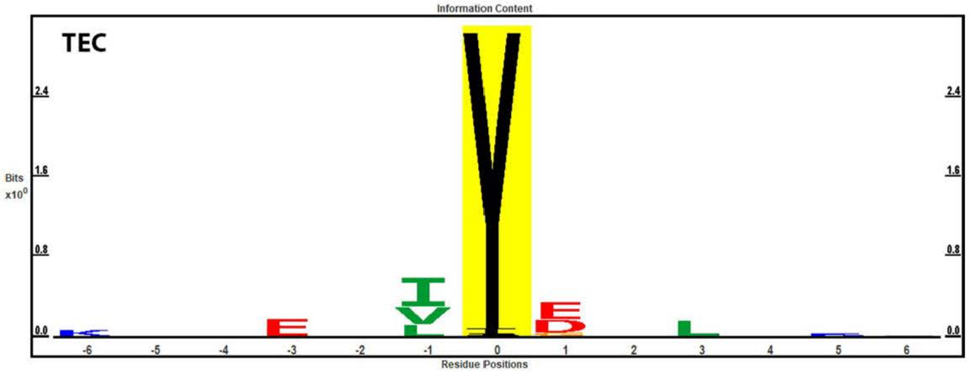
Information Content
TEC
Bits
x10^0
Residue Positions

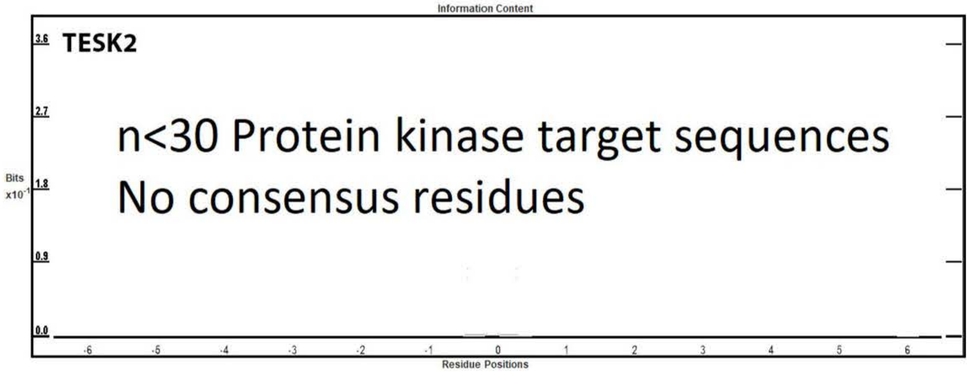
Information Content
TESK2
n<30 Protein kinase target sequences
No consensus residues
Bits
x10^-1
Residue Positions

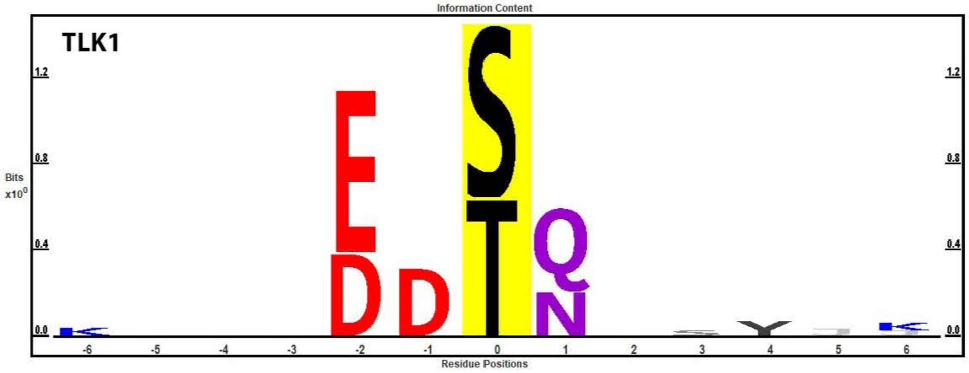
Information Content
TLK1
Bits
x10^0
1.2
0.8
0.4
0.0
-6 -5 -4 -3 -2 -1 0 1 2 3 4 5 6
Residue Positions

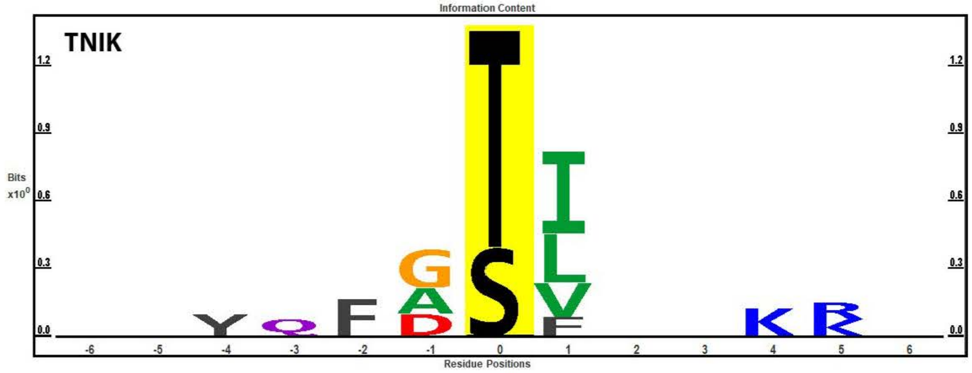
Information Content
TNIK
Bits
x10^0
1.2
0.9
0.6
0.3
0.0
-6 -5 -4 -3 -2 -1 0 1 2 3 4 5 6
Residue Positions

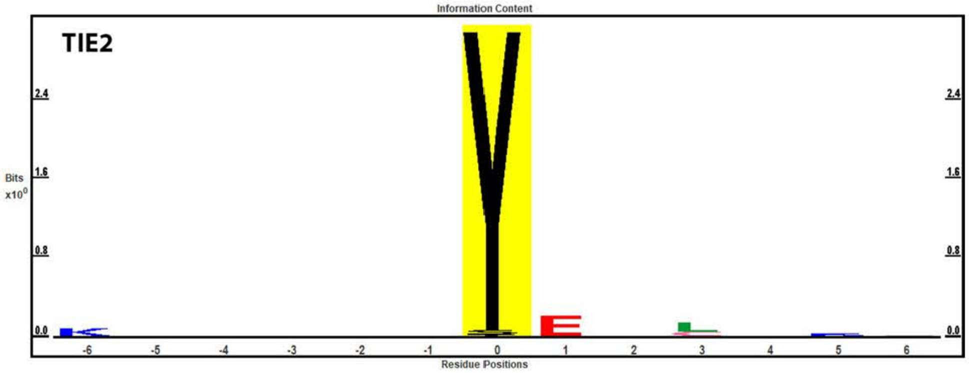
Information Content
TIE2
Bits
x10^0
2.4
1.6
0.8
0.0
-6 -5 -4 -3 -2 -1 0 1 2 3 4 5 6
Residue Positions

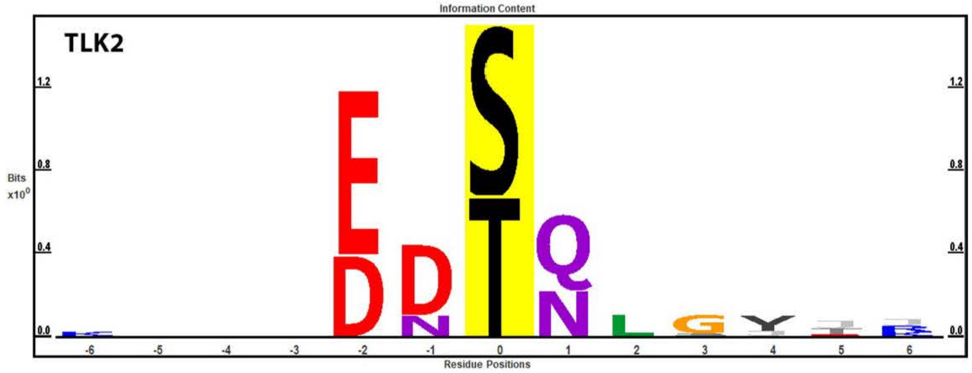
Information Content
TLK2
Bits
x10^0
1.2
0.8
0.4
0.0
-6 -5 -4 -3 -2 -1 0 1 2 3 4 5 6
Residue Positions

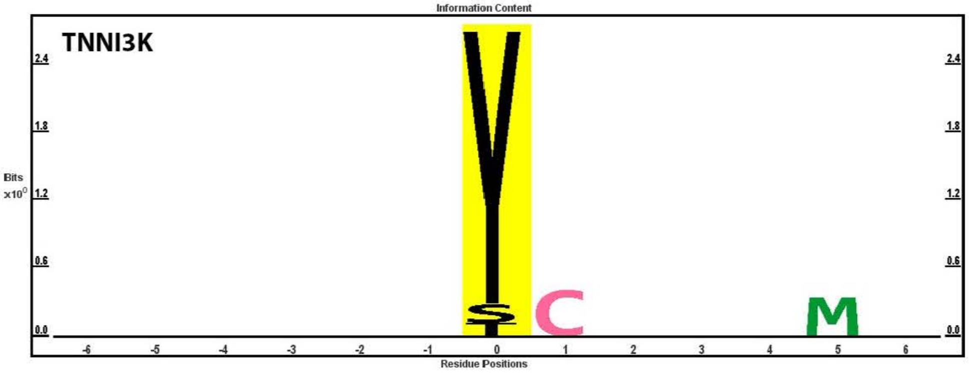
Information Content
TNNI3K
Bits
x10^0
2.4
1.8
1.2
0.6
0.0
-6 -5 -4 -3 -2 -1 0 1 2 3 4 5 6
Residue Positions

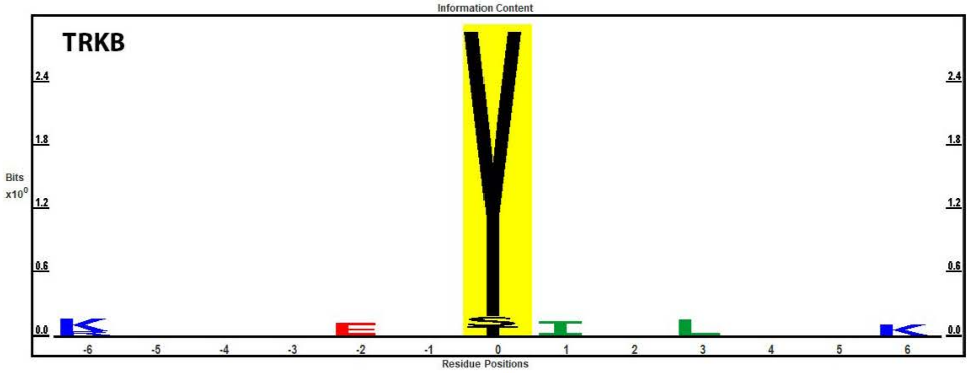
Information Content
TRKB
Bits
x10^0
2.4
1.8
1.2
0.6
0.0
-6 -5 -4 -3 -2 -1 0 1 2 3 4 5 6
Residue Positions

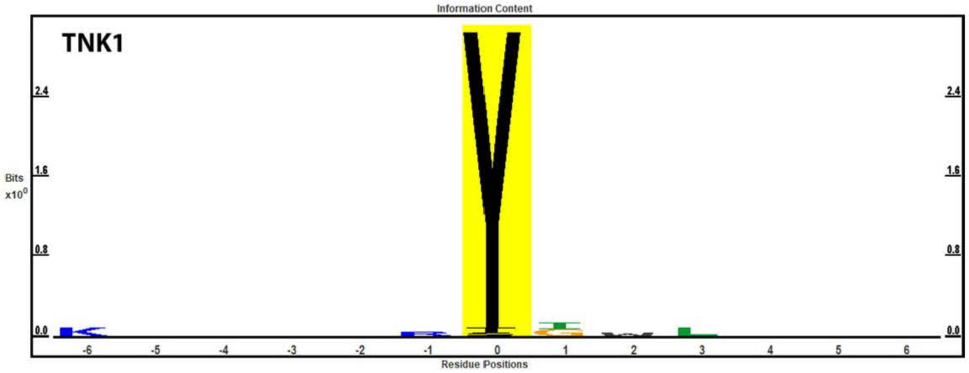
Information Content
TNK1
Bits
x10^0
2.4
1.6
0.8
0.0
-6 -5 -4 -3 -2 -1 0 1 2 3 4 5 6
Residue Positions

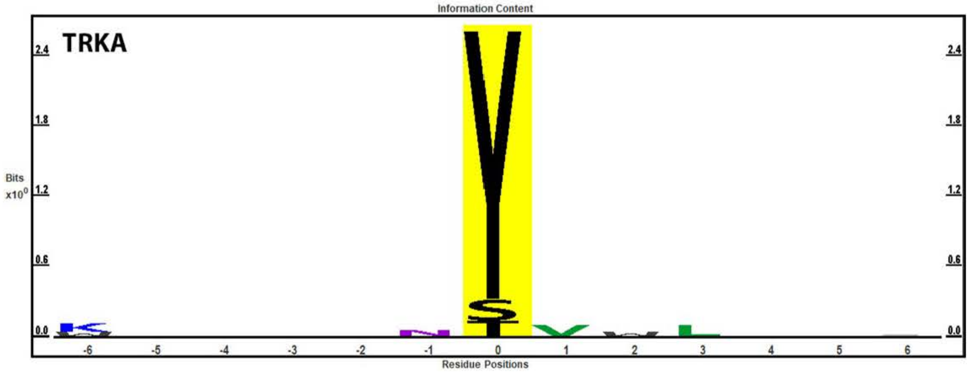
Information Content
TRKA
Bits
x10^0
2.4
1.8
1.2
0.6
0.0
-6 -5 -4 -3 -2 -1 0 1 2 3 4 5 6
Residue Positions

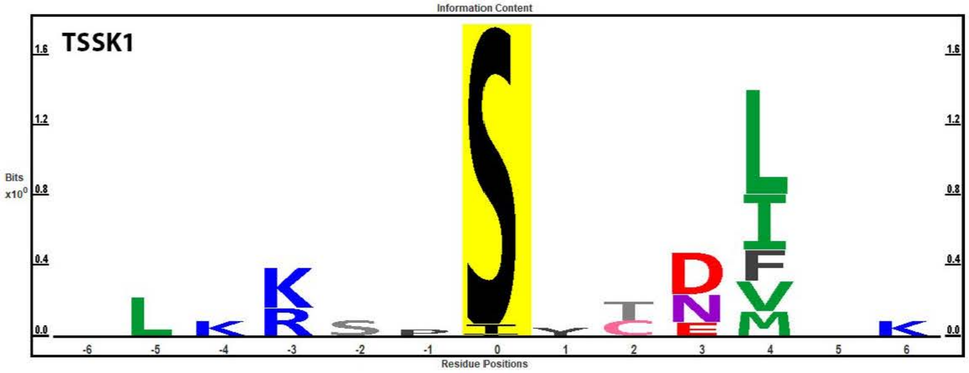
Information Content
TSSK1
Bits
x10^0
Residue Positions

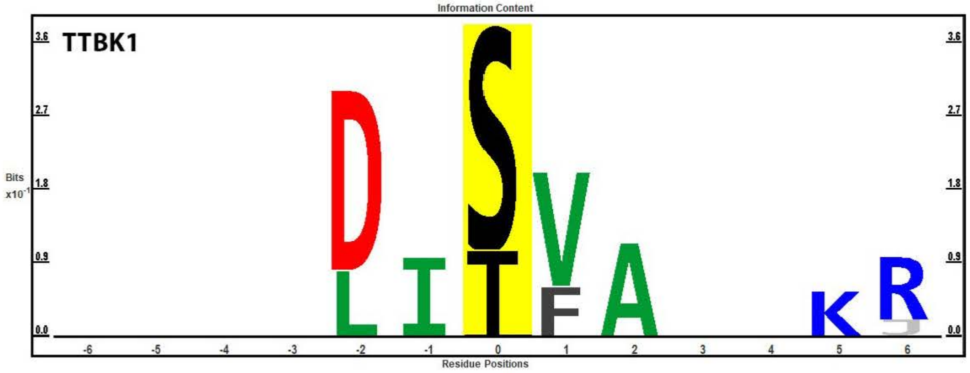
Information Content
TTBK1
Bits
x10^-1
Residue Positions

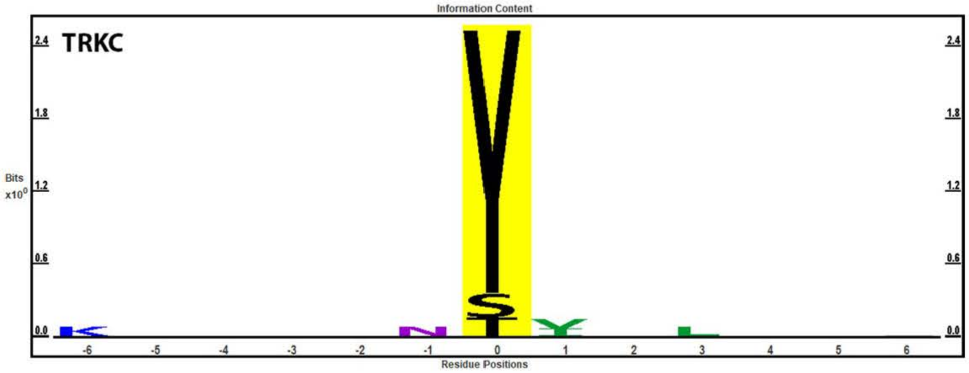
Information Content
TRKC
Bits
x10^0
Residue Positions

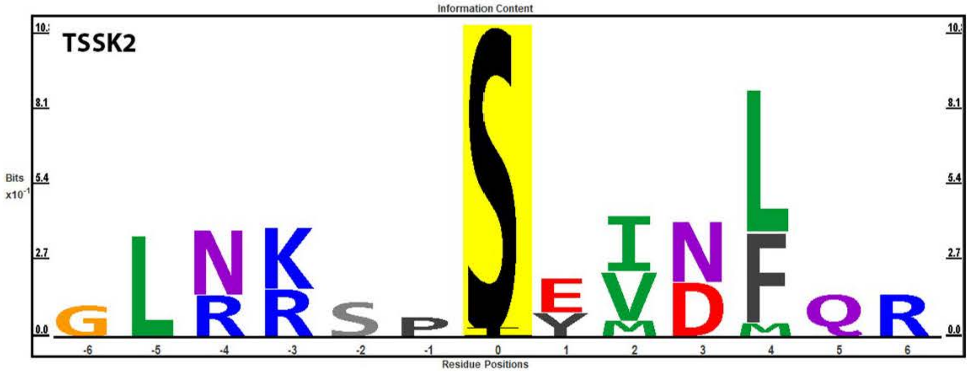
Information Content
TSSK2
Bits
x10^-1
Residue Positions

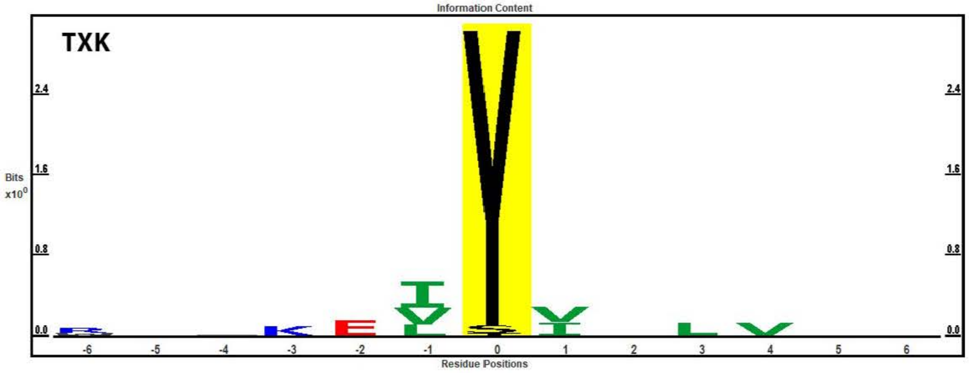
Information Content
TXK
Bits
x10^0
2.4
1.6
0.8
0.0
-6 -5 -4 -3 -2 -1 0 1 2 3 4 5 6
Residue Positions

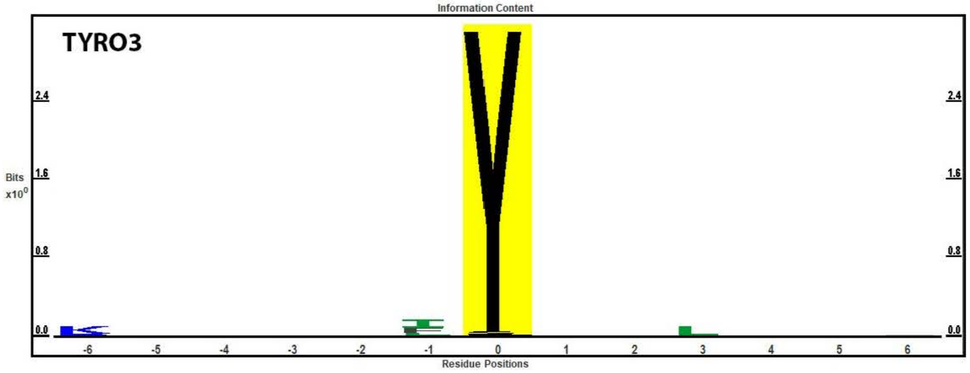
Information Content
TYRO3
Bits
x10^0
2.4
1.6
0.8
0.0
-6 -5 -4 -3 -2 -1 0 1 2 3 4 5 6
Residue Positions

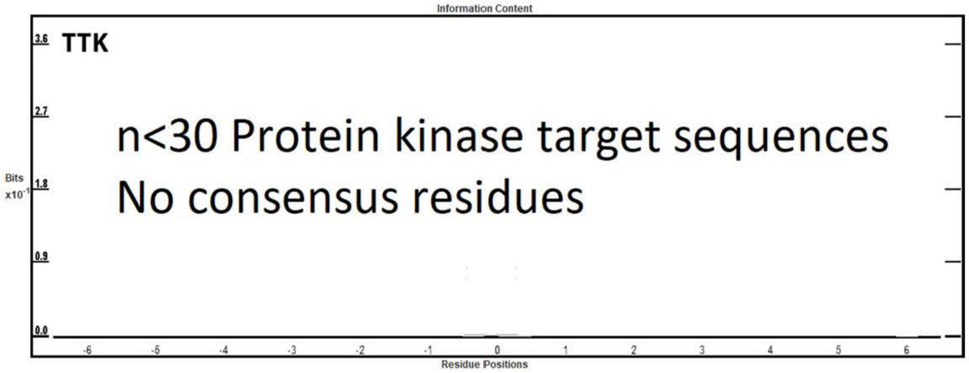
Information Content
TTK
n<30 Protein kinase target sequences
No consensus residues
Bits
x10^-1
3.6
2.7
1.8
0.9
0.0
-6 -5 -4 -3 -2 -1 0 1 2 3 4 5 6
Residue Positions

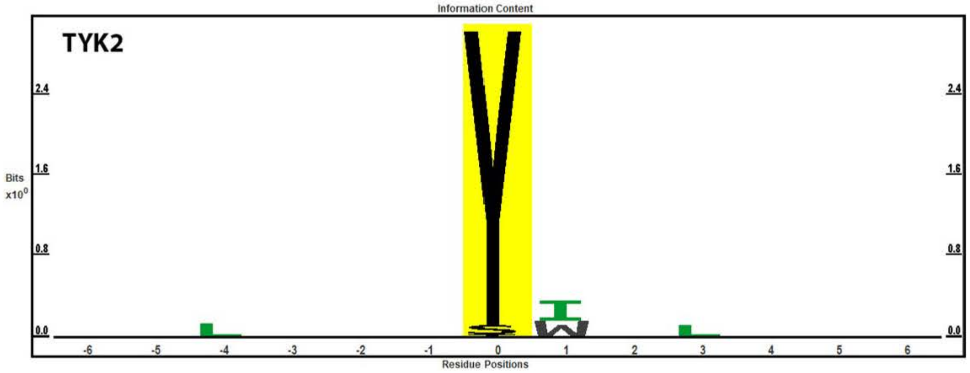
Information Content
TYK2
Bits
x10^0
2.4
1.6
0.8
0.0
-6 -5 -4 -3 -2 -1 0 1 2 3 4 5 6
Residue Positions

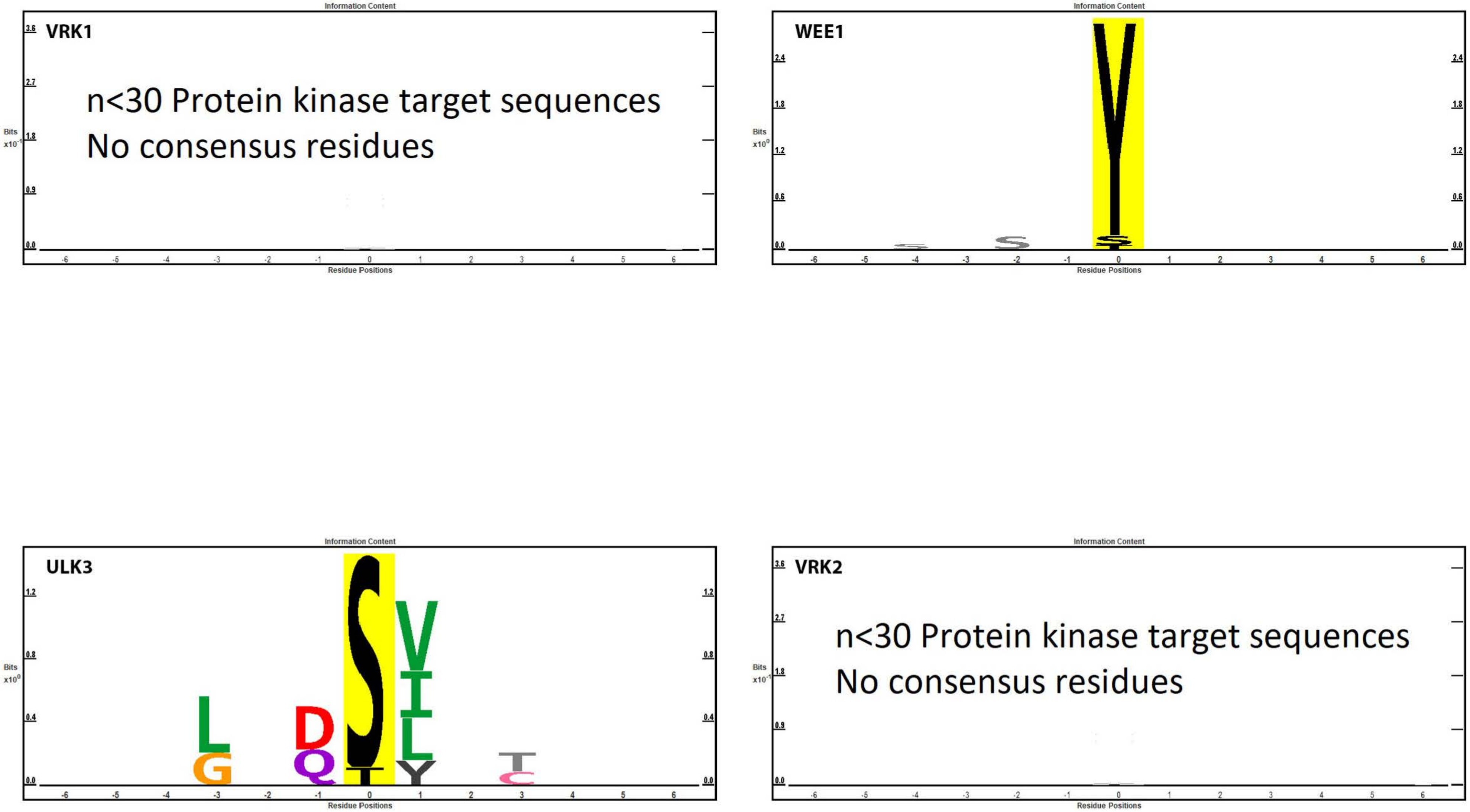
Information Content
VRK1
n<30 Protein kinase target sequences
No consensus residues
Bits
x10⁻¹
Residue Positions
Information Content
WEE1
Bits
x10⁰
Residue Positions
Information Content
ULK3
Bits
x10⁰
Residue Positions
Information Content
VRK2
n<30 Protein kinase target sequences
No consensus residues
Bits
x10⁻¹
Residue Positions

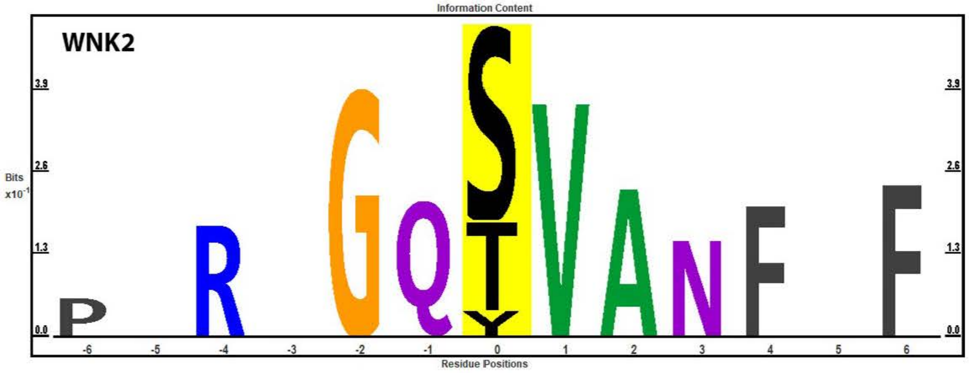
Information Content
WNK2
Bits
x10⁻¹
Residue Positions

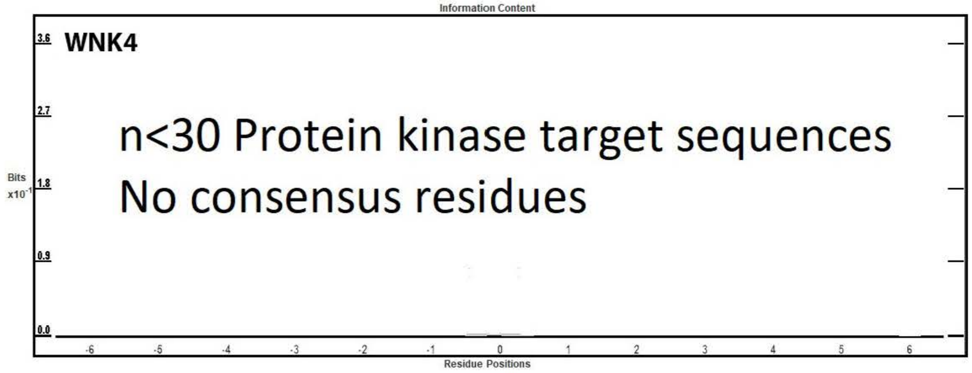
Information Content
WNK4
n<30 Protein kinase target sequences
No consensus residues
Bits
x10⁻¹
Residue Positions

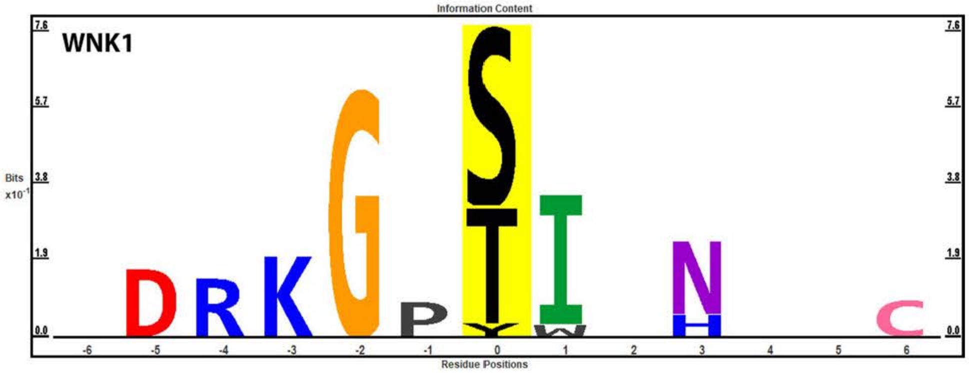
Information Content
WNK1
Bits
x10⁻¹
Residue Positions

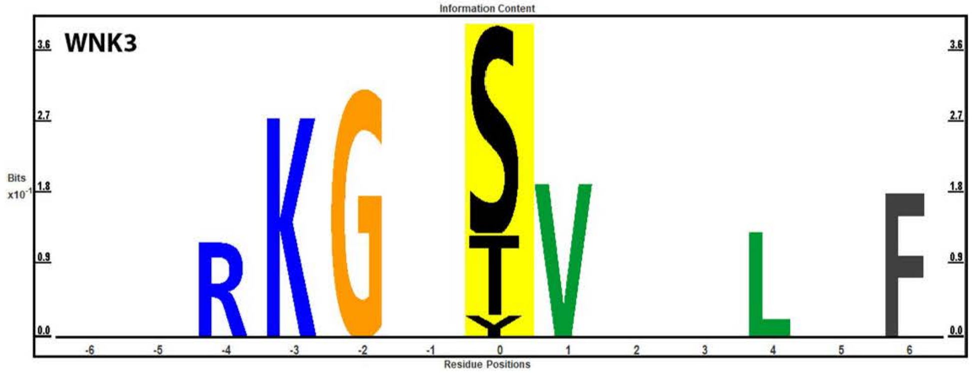
Information Content
WNK3
Bits
x10⁻¹
Residue Positions

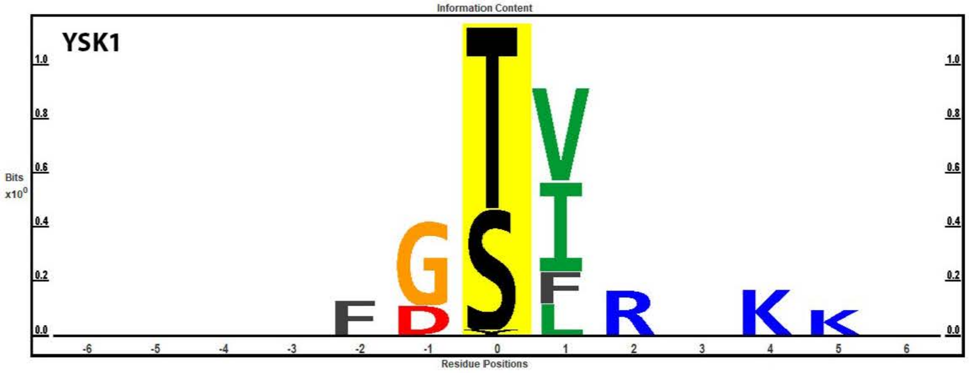
Information Content
YSK1
Bits
x10^0
1.0
0.8
0.6
0.4
0.2
0.0
-6 -5 -4 -3 -2 -1 0 1 2 3 4 5 6
Residue Positions

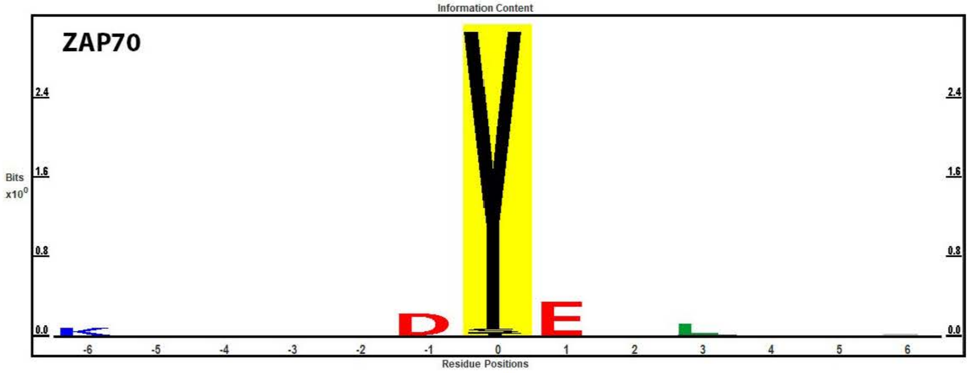
Information Content
ZAP70
Bits
x10^0
2.4
1.6
0.8
0.0
-6 -5 -4 -3 -2 -1 0 1 2 3 4 5 6
Residue Positions

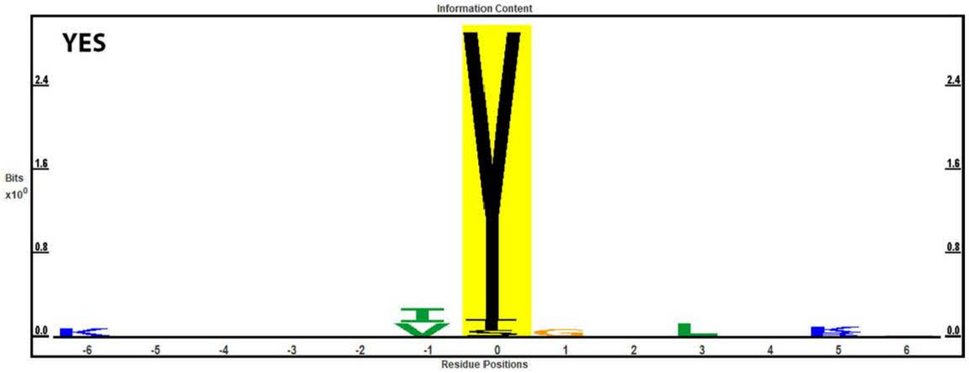
Information Content
YES
Bits
x10^0
2.4
1.6
0.8
0.0
-6 -5 -4 -3 -2 -1 0 1 2 3 4 5 6
Residue Positions

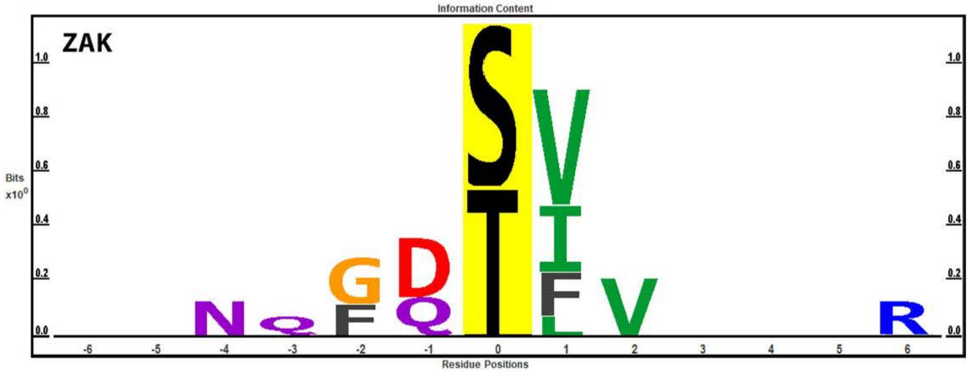
Information Content
ZAK
Bits
x10^0
1.0
0.8
0.6
0.4
0.2
0.0
-6 -5 -4 -3 -2 -1 0 1 2 3 4 5 6
Residue Positions
